# Structure activity relationship studies on rhodanines and derived enethiol inhibitors of metallo-β-lactamases

**DOI:** 10.1016/j.bmc.2018.02.043

**Published:** 2018-07-15

**Authors:** Dong Zhang, Marios S. Markoulides, Dmitrijs Stepanovs, Anna M. Rydzik, Ahmed El-Hussein, Corentin Bon, Jos J.A.G. Kamps, Klaus-Daniel Umland, Patrick M. Collins, Samuel T. Cahill, David Y. Wang, Frank von Delft, Jürgen Brem, Michael A. McDonough, Christopher J. Schofield

**Affiliations:** a Department of Chemistry, University of Oxford, Chemistry Research Laboratory, 12 Mansfield Road, Oxford OX1 3TA, United Kingdom; b Diamond Light Source, Harwell Science and Innovation Campus, Didcot OX11 0DE, United Kingdom; c The National Institute of Laser Enhanced Science, Cairo University, Egypt; d Structural Genomics Consortium (SGC), University of Oxford, Oxford, OX3 7DQ, UK; ^e^Department of Biochemistry, University of Johannesburg, Auckland Park, 2006, South Africa

**Keywords:** Metallo β-lactamase, Antibiotic resistance, Carbapenemase, Inhibitors, Structure activity relationships

## Abstract

Metallo-β-lactamases (MBLs) enable bacterial resistance to almost all classes of β-lactam antibiotics. We report studies on enethiol containing MBL inhibitors, which were prepared by rhodanine hydrolysis. The enethiols inhibit MBLs from different subclasses. Crystallographic analyses reveal that the enethiol sulphur displaces the di-Zn(II) ion bridging ‘hydrolytic’ water. In some, but not all, cases biophysical analyses provide evidence that rhodanine/enethiol inhibition involves formation of a ternary MBL enethiol rhodanine complex. The results demonstrate how low molecular weight active site Zn(II) chelating compounds can inhibit a range of clinically relevant MBLs and provide additional evidence for the potential of rhodanines to be hydrolysed to potent inhibitors of MBL protein fold and, maybe, other metallo-enzymes, perhaps contributing to the complex biological effects of rhodanines. The results imply that any medicinal chemistry studies employing rhodanines (and related scaffolds) as inhibitors should as a matter of course include testing of their hydrolysis products.

## Graphical abstract

**Figure:**
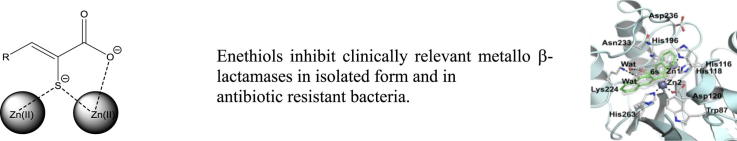

## 1 Introduction

**Fig. 1:**
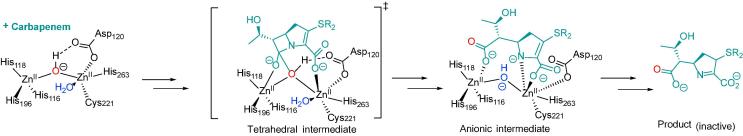
Outlined mechanism for B1 MBL catalysed β-lactam hydrolysis as exemplified by hydrolysis of a carbapenem. The anionic intermediate, but not the tetrahedral intermediate^‡,^ has been observed spectroscopically^25^.

Following the clinical introduction of the penicillins in the 1940s, β-lactam antibiotics came to be, and remain, amongst the most important medicines in use.^1^ The remarkable longevity and the widespread ability of β-lactams to act as antibiotics has been achieved in the face of multiple resistance mechanisms,^2^, ^3^, ^4^ the most prevalent of which is mediated by β-lactamases which catalyse the hydrolysis of β-lactams.^2^, ^5^, ^6^, ^7^, ^8^, ^9^, ^10^ There are two mechanistic types of β-lactamase – the serine β-lactamases (SBLs), which employ a nucleophilic serine (Ambler classes A, C, D) and the metallo-β-lactamases (MBLs), which utilise a Zn(II) bound hydroxide in β-lactam hydrolysis (class B).^6^, ^9^ Inhibitors of the class A and C SBLs have been used successfully in combination with β-lactams.^11^ More recently, a broad spectrum inhibitor of class A, C, and some D SBLs,^12^ Avibactam, has been introduced for clinical use in combination with a cephalosporin.^13^, ^14^ However, no clinically useful MBL inhibitors are currently available,^15^, ^16^ and most SBL inhibitors are susceptible to MBL catalysed hydrolysis (Fig. 1).^17^

The class B MBLs all utilise one (subclass B2 and some B3) or two (subclasses B1 and some B3) Zn(II) ions at their active site.^18^ The B1 MBLs are the most important MBLs from a clinical perspective.^19^ Developing MBL inhibitors with the breadth of activity required for clinical application is challenging, because of variations in the mobile regions surrounding the active sites of B1 MBLs.^20^ Various types of MBL inhibitor have been developed,^21^ most of which chelate to the active site Zn(II) ion(s); however, few if any, of the reported inhibitors have the required breadth of potency against the three major B1 MBL families that are clinically widespread (i.e. the New Delhi MBL (NDM), Verona integron-encoded MBL (VIM), and Imipenemase (IMP) MBLs).

**Scheme 1:**
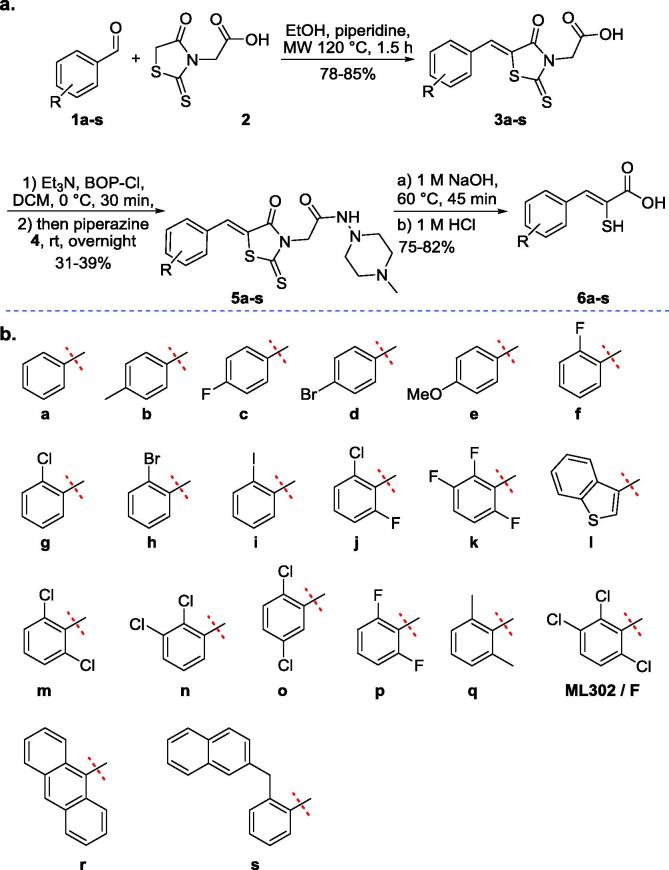
Synthesis of enethiol based β-lactamase inhibitors. (a) Route for preparation of **ML302 5a-q** analogues and **ML302F 6a-q** analogues.^24^ (b) R groups for **5a-q** and **6a-q**. MW: microwave irradiation.

We have developed an assay platform for MBLs employing a fluorogenic cephalosporin substrate,^22^ which we used to screen potential β-lactamase inhibitors. As part of this work we tested the potency of the rhodanine **ML302** (Scheme 1), which was identified following a high-throughput screen, as inhibitor of VIM-2 and IMP-1.^23^ Unexpectedly, we found that **ML302** undergoes hydrolysis to give the enethiol fragment, **ML302F** (Scheme 1), which inhibits MBLs *via* active site Zn(II) ion chelation; in the case of VIM-2 we observed the unusual formation of a ternary complex between the enzyme and two different ligands, **ML302** and **ML302F**.^24^ We now describe structure activity relationship and biophysical studies on the rhodanine derived enethiol MBL inhibitors.^25^

## 2 Materials and methods

### 2.1 Protein production and purification

Recombinant forms of NDM-1, VIM-2, SPM-1, IMP-1, BcII and CphA MBLs and TEM-1, CTX-M-15, AmpC and OXA-10 SBLs were produced as described previously. 22, 23, 26

### 2.2 Experimental procedures for synthesis

**Scheme 2:**
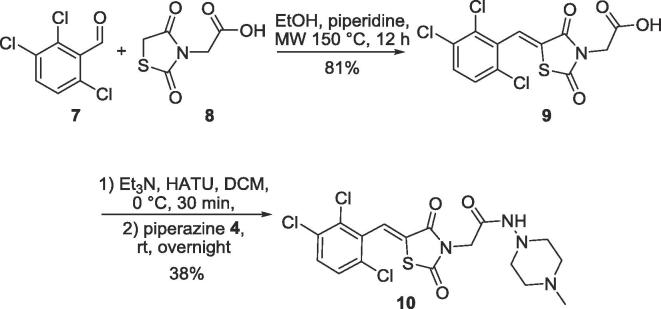
Synthesis of the 2,4-dione derivative **10** of **ML302**.

The syntheses of **3a-s**, **ML302** analogues **5a-s**, and **ML302F** analogues **6a-s** were performed as previously described^24^. Following the procedure of Brem et al.,^24^ **10** was prepared in two steps from the corresponding aldehyde *via* Knoevenagel condensation with rhodanine followed by amide coupling (Scheme 2).

**Scheme 3:**
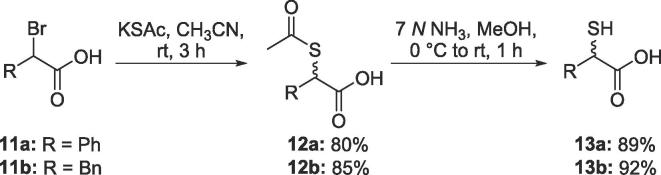
Synthesis of racemic α-mercaptocarboxylic acids 13a and 13b^26^.

Following the procedure of Shaffer et al.,^27^ α-mercaptocarboxylic acids **13a** and **13b** were prepared in two steps from the corresponding α-bromocarboxylic acids via nucleophilic substitution with potassium thioacetate followed by basic hydrolysis (Scheme 3).

**Scheme 4:**
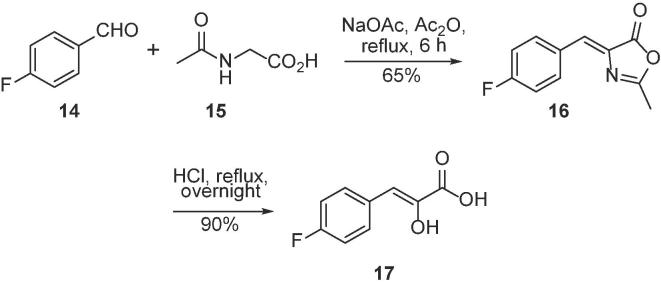
Synthesis of α-hydroxycinnamic acid **17**.^27^

Following the procedure of Braña et al.,^28^ α-hydroxycinnamic acid **7** was prepared in two steps from the corresponding aldehyde via Erlenmeyer azlactone synthesis of **6** followed by acid hydrolysis (Scheme 4).

**Scheme 5:**
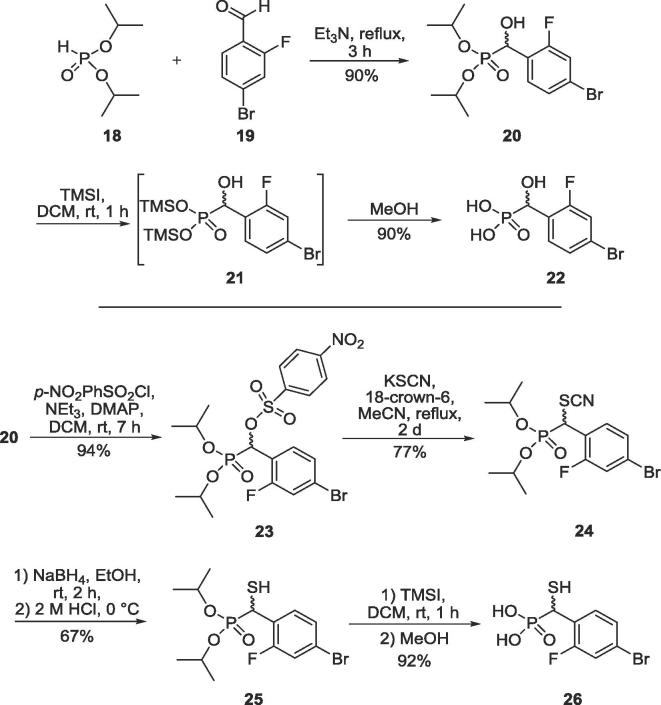
Synthesis of α-hydroxy phosphonic acid **22** and α-sulfanyl phosphonic acid **26**^41^, ^42^, ^43^, ^44^, ^45^, ^46^, ^47^, ^48^, ^49^, ^50^.

α-Hydroxy phosphonic acid **22** and α-sulfanyl phosphonic acid **26** were synthesised according to the procedure of Bebrone et al.^29^ (Scheme 5). Compounds were characterised as detailed in Supplemental information.

### 2.3 Inhibition analyses

Inhibition analyses against bacterial MBLs and SBLs were performed as described previously.^22^, ^24^, ^26^ Residual enzyme activities were determined for a range of inhibitor concentrations. Non-linear regression fitting of IC_50_ curves was carried out using a three-parameter dose–response curve in GraphPad Prism. Errors in IC_50_ values are expressed as:

$$\frac{\sigma (\log{\mathrm{IC}}_{50})}{\log{\mathrm{IC}}_{50}}\times {\mathrm{IC}}_{50}$$

Additional data are presented in Supplemental Information (Table S1 and Figs. S1–S6).

### 2.4 NMR time course experiments

NMR experiments were carried out using a Bruker Avance III 700 MHz machine equipped with a TCI inverse cryoprobe or a Bruker Avance III HD 600 MHz spectrometer equipped with a Prodigy cryoprobe at 298 K. Data were analysed using Bruker Topspin 3.5. Processing of spectra was done with a Lorentzian line broadening of 0.3 Hz. Chemical shifts (δ) are given as parts per million (ppm) relative to residual HDO (δH 4.70 ppm for ^1^H NMR).

For rhodanine stability studies solutions were buffered in either freshly prepared NH_4_HCO_3_ (50 mM, pH 7.50) or Tris-d_11_ (50 mM, pH 7.50) both with NaCl (100 mM) in D_2_O. **ML302** stock solution (50 mM in DMSO‑*d*_6_) was added to a sample to give a final concentration of 200 µM. When specified, NDM-1 was added to give a final concentration of 1 µM. The reaction was followed at 1 h intervals over 18 h (Figs. S7 and S8).

### 2.5 Crystallography

BcII crystals were prepared using the sitting drop vapour diffusion method (293 K, 200 mM ammonium sulfate, 100 mM bis-Tris buffer pH 5.5, 25% ^w^/_v_ polyethylene glycol 3350, and 5 mM inhibitor). The crystals were cryoprotected using well solution diluted to 25% ^v^/_v_ glycerol before being flash cooled in liquid nitrogen. All data sets were collected at 100 K. All data were autoprocessed at the beamline using xia2.^30^ The structures were solved using molecular replacement (using PDB ID 4TYT as a search model^24^) within PHASER. The structures were then fitted to the electron density and refined using COOT^31^ and PHENIX^32^ until R_work_ and R_free_ no longer converged.

For VIM-2, crystals were grown as reported^25^; crystal soaking was performed by directly adding 32.5 nL of a 100 mM stock of **ML302F** in DMSO to 300 nL crystal drops using a Labcyte Echo 550 acoustic drop dispenser, which is part of the XChem pipeline at Diamond Light Source.^33^ Crystals were soaked with the ligand for 135 min before the addition of 300 nL of 50% ^v^/_v_ glycerol and flash cooling. X-ray diffraction data were collected at Diamond Light Source beamline I04-1, and processed with Diamond’s automated processing pipelines, using xia2^28^ and XDS,^34^ with XChemExplorer^35^ and Dimple used for electron density generation. Initial ligand bound electron density was identified using PanDDA.^36^ Grade^37^ was used for ligand restraint generation. Final model preparation was performed by iterative cycles of refinement using REFMAC^38^ and model building in Coot.^31^ Data collection, PDB codes, and refinement statistics for all structures are given in **Table S2**.

## 3 Results and discussion

### 3.1 Synthetic routes to the enethiols and related compounds

The synthetic route used for the preparation of **ML302** and **ML302F** analogues (**5a-q** and **6a-q**, respectively) is shown in Scheme 1.^24^ **ML302** analogues **5a-q** were prepared in two steps, by Knoevenagel-type condensation of rhodanine-3-acetic acid **2** and the appropriate aldehyde **1a–q** to provide, predominantly, the *Z*-isomer of the benzylidene-4-oxo-2-thioxo-thiazolidin-3-yl)-acetic acid derivatives (**3a-3q**),^39^, ^40^ which were coupled with amino-4-methyl-piperazine **4** to give the desired **ML302** analogues (**5a-q**). Base mediated hydrolysis of the **ML302** derivatives **5a-q** then provided **ML302F** and its analogues **6a-q** (Scheme 1).

Consistent with a literature report,^41^ we observed decomposition of the **ML302F** analogues **6a-q** in DMSO. Thus, biochemical assays were performed by using the corresponding sodium salts (conversion with 100 mM sodium bicarbonate immediately prior to assay), and characterizations were performed in MeOD (see Section 3 in Supplementary Information). The (**ML302F**) **6** analogues showed good stability as crystalline solids in their acid form, after purification by re-crystallisation from toluene.

To investigate the effects of changing the electronic properties of the thiazolidine ring on MBL inhibition, the 2,4-dione derivative **10** of **ML302** was prepared from **8** in a similar manner to that used for the synthesis of the **ML302 5a-q** and **ML302F 6a-q** analogues (Scheme 2). The 2,4-dioxo thiazolidineacetic acid **8** was found to be substantially less reactive towards Knoevenagel condensation than the analogous 4-oxo-2-thioxo 2 ring system. This reduced reactivity coupled with the broad spectrum biological importance of these types of ring systems has led to the development of catalytic protocols for their synthesis (e.g. pyridine/EtOH, piperidine/EtOH/AcOH, CH_3_COONa/AcOH, NH_4_OAc/AcOH, NH_4_OAc, NH_4_OAc/toluene etc.).^42^, ^43^, ^44^, ^45^, ^46^, ^47^, ^48^, ^49^, ^50^, ^51^ However, these conditions proved to be low yielding in our hands. Applying longer reaction times (12 h under microwave conditions), using the EtOH/piperidine system provided the best result (80%) for preparation of 2,4-dioxo thiazolidineacetic acid **9**.

For comparison with the enethiols, α-mercaptocarboxylic acids **13a** and **13b** were prepared in two steps from the corresponding α-bromocarboxylic acids (**11a** and **11b**, respectively), following the procedure of Shaffer et al.^27^ *via* nucleophilic substitution with potassium thioacetate followed by basic hydrolysis (Scheme 3). The α-hydroxycinnamic acid enol analogue **17** of **ML302F** was prepared from the corresponding aldehyde *via* Erlenmeyer azlactone synthesis of **16** followed by acid mediated hydrolysis (Scheme 4).^28^

To investigate the importance of the carboxylate^52^ in MBL inhibition, α-sulfanyl phosphonic acid **26** was synthesised in five steps in an overall yield of 40% from diisopropyl phosphite (Scheme 5). Thus, 1,2-addition of diisopropyl phosphite **18** to 4-bromo-2-fluoro-benzaldehyde **19** afforded the α-hydroxy phosphonate **20**, which was converted to the *para*-nitrobenzenesulfonate derivative **23**. Nucleophilic substitution using potassium thiocyanate gave the thiocyanate derivative **24** which was reduced with sodium borohydride to afford α-sulfanyl phosphonate **25**. Treatment of α-sulfanyl phosphonate **25** with trimethylsilyliodide, followed by methanolysis afforded the α-sulfanyl phosphonic acid **26**. α-Hydroxy phosphonic acid **22** was prepared in a similar way, by hydrolysis of the α-hydroxy phosphonate **20**.

### 3.2 Inhibition assays

We first screened the inhibitors against a representative set of presently clinically important and other MBLs^22^, comprising both Class B1 enzymes (NDM-1, New Delhi MBL-1; VIM-2, Verona integron–encoded MBL-2; BcII, *Bacillus cereus* II MBL; SPM-1, São Paulo MBL-1; IMP-1, imipenemase MBL-1) and the Class B2 MBL CphA (carbapenem hydrolysing MBL from *Aeromonas hydrophila*).^22^ The latter MBL (CphA) only utilises one active site Zn(II) ion in catalysis, whilst the others normally use two Zn(II) ions. We also screened the inhibitors against a representative panel of SBLs from different classes (TEM-1, Temoneira β-lactamase-1, class A; CTX-M-15, cefotaxime hydrolysing β-lactamase from Munich 15, class A, extended spectrum β-lactamase (ESBL); AmpC *E. coli*, class C, and OXA-10, oxacillinase-10, class D).

A number of trends for the rhodanine derived inhibitors (compound series, **3a-q**, **5a-q**, and **6a-q**) are apparent from the results (Table 1). In all cases the enethiols (**6a-q**) were the most potent inhibitors within a given set of rhodanine/enethiol derivatives, implying that the enethiols are the prime source of inhibition. With a few exceptions (mostly in the case of VIM-2 and the atypical subclass B1 MBL, SPM-1), the rhodanine-3-acetic acids (**3a-q**) were either inactive (at 50 µM) or only weakly active compared to the enethiols (**6a-q**). This is also the case for the amides (**ML302**, **5a-5q**) against certain MBLs, though there were more exceptions (e.g. **5i-q)**. It is likely that, at least to some extent, the activities for the rhodanine-3-acetic acids (**3a-q**) and amides (**ML302**, **5a-5q**) result from (partial) hydrolysis of the compounds to give their corresponding enethiols (**6a-q**). The differences in activities between rhodanine-3-acetic acids (**3a-q**) or amides (**ML302**, **5a-5q**) may in part reflect the extent of hydrolysis. Whether or not such hydrolysis is enzyme catalysed is difficult to (partial) judge given the potency of enethiol (**6a-q**) inhibition. The proposal of enzyme mediated hydrolysis is supported by the different results observed for analogous amides and enethiols. Thus, amide **ML302** manifests similar inhibition compared to the enethiol **ML302F** for two of the tested B1 subclass MBLs (IMP-1 and VIM-2), whereas for SPM-1, BcII and NDM-1, **ML302** was ∼5, ∼4 and ∼15-fold less active than **ML302F**. For the subclass B2 MBL CphA, **ML302** (IC_50_ value > 50 µM) was also significantly less active than **ML302F** (IC_50_ value 200 nM). Thus, **ML302** may be hydrolysed at different rates by different MBLs.

**Table 1:** Screening results for the inhibition of MBLs by rhodanine derived inhibitors.

**Figure:**
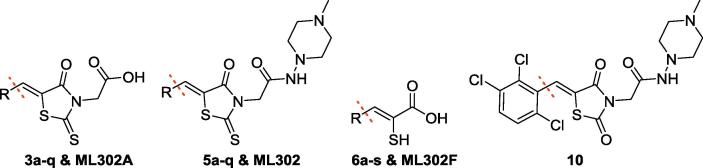

**Compound**	R	IC_50_ (µM)				
		SPM-1	IMP-1	BcII	VIM-2	NDM-1
**3a** **5a** **6a**		>50 >50 0.3 ± 7×10^−3^	>50 22.7 ± 0.3 0.3 ± 0.03	>50 >50 0.7 ± 0.2	36.8 ± 0.3 16.4 ± 0.1 0.4 ± 0.02	>50 26.7 ± 1.0 7.9 ± 0.1
**3b** **5b** **6b**		>50 >50 0.3 ± 7×10^−3^	>50 >50 0.2 ± 5×10^−3^	>50 >50 0.7 ± 0.05	32.0 ± 0.7 >50 0.4 ± 0.01	>50 28.0 ± 1.0 3.7 ± 0.1
**3c** **5c** **6c**		>50 >50 4.1 ± 0.4	>50 >50 2.7 ± 0.1	>50 >50 8.2 ± 0.1	34.5 ± 0.6 >50 9.7 ± 0.3	>50 >50 5.0 ± 0.4
**3d** **5d** **6d**		39.7 ± 0.8 >50 2.3 ± 0.3	>50 28.7 ± 1.2 3.3 ± 0.4	>50 >50 9.9 ± 0.2	>50 44.0 ± 0.4 9.4 ± 0.2	>50 >50 6.3 ± 0.2
**3e** **5e** **6e**		>50 >50 0.6 ± 0.1	>50 31.5 ± 3.2 0.8 ± 5×10^−3^	>50 >50 2.4 ± 0.1	>50 >50 0.4 ± 0.02	>50 48.9 ± 0.7 8.2 ± 0.3
**3f** **5f** **6f**		>50 42.7 ± 0.8 0.3 ± 7×10^−3^	>50 22.6 ± 0.3 1.4 ± 1.0	>50 26.3 ± 1.5 4.0 ± 0.1	17.6 ± 0.7 6.2 ± 0.2 3.3 ± 0.2	>50 >50 12.9 ± 0.4
**3g** **5g** **6g**		25.1 ± 1.5 9.8 ± 0.4 0.1 ± 3×10^−3^	43.2 ± 2.1 7.6 ± 0.1 0.4 ± 0.01	>50 >50 0.9 ± 0.1	6.7 ± 0.2 2.0 ± 0.2 0.4 ± 0.02	>50 >50 5.1 ± 0.5
**3h** **5h** **6h**		36.6 ± 0.8 8.1 ± 0.3 3.5 ± 0.3	32.5 ± 1.3 8.3 ± 0.2 0.8 ± 0.06	>50 14.6 ± 0.8 5.8 ± 0.1	>50 11.4 ± 1.0 2.7 ± 0.1	>50 >50 16.2 ± 0.3
**3i** **5i** **6i**		2.0 ± 0.5 8.8 ± 0.3 1.4 ± 0.4	22.4 ± 0.4 37.1 ± 2.1 0.7 ± 0.02	31.7 ± 1.0 >50 6.3 ± 0.1	5.9 ± 0.1 37.6 ± 1.0 3.5 ± 0.1	>50 >50 5.0 ± 0.2
**3j** **5j** **6j**		1.2 ± 0.3 0.1 ± 4×10^−3^ 4.3 × 10^−3^ ± 3×10^−4^	43.7 ± 0.5 0.3 ± 0.01 0.03 ± 3×10^−4^	>50 2.0 ± 0.2 0.06 ± 2×10^−3^	9.8 ± 2.8 0.8 ± 0.1 0.05 ± 1×10^−3^	>50 >50 4.4 ± 0.2
**3k** **5k** **6k**		8.8 ± 0.3 1.5 ± 0.2 0.1 ± 2×10^−3^	46.5 ± 0.7 1.6 ± 0.4 0.4 ± 0.02	27.0 ± 2.2 3.5 ± 0.2 0.3 ± 0.01	47.6 ± 0.4 2.6 ± 0.1 0.3 ± 0.01	>50 40.1 ± 2.1 3.3 ± 0.1
**3L** **5L** **6L**		13.9 ± 0.5 >50 0.4 ± 0.02	>50 >50 0.7 ± 0.05	21.7 ± 2.4 7.2 ± 3.3 3.1 ± 0.3	36.9 ± 0.2 41.8 ± 3.6 0.5 ± 0.03	>50 >50 11.1 ± 0.3
**3m** **5m** **6m**		3.5 ± 0.4 0.2 ± 2×10^−3^ 0.01 ± 1×10^−4^	18.9 ± 0.8 0.2 ± 4×10^−5^ 0.01 ± 6×10^−5^	41.8 ± 2.3 0.2 ± 0.3 0.07 ± 3×10^−3^	0.6 ± 0.1 0.3 ± 0.01 0.03 ± 2×10^−4^	>50 28.4 ± 0.4 0.6 ± 0.1
**3n** **5n** **6n**		1.0 ± 0.1 0.3 ± 8×10^−3^ 0.2 ± 7×10^−3^	46.2 ± 1.4 0.6 ± 0.2 1.0 ± 2	22.1 ± 1.0 1.1 ± 0.3 1.0 ± 0.2	7.6 ± 0.3 39.5 ± 0.5 1.3 ± 0.2	>50 32.8 ± 0.8 46.5 ± 0.7
**3o** **5o** **6o**		16.6 ± 1.0 2.8 ± 0.2 0.8 ± 0.06	>50 7.0 ± 0.9 4.7 ± 0.2	>50 15.4 ± 1.0 1.6 ± 0.5	>50 >50 1.5 ± 0.2	>50 >50 45.3 ± 0.6
**3p** **5p** **6p**		32.1 ± 2.3 1.3 ± 0.2 0.06 ± 2×10^−3^	>50 1.3 ± 0.2 0.2 ± 9×10^−3^	>50 3.0 ± 0.1 0.1 ± 2×10^−3^	6.8 ± 0.2 41.7 ± 1.9 0.2 ± 0.07	>50 >50 21.5 ± 0.8
**3q** **5q** **6q**		>50 0.2 ± 7×10^−3^ 0.02 ± 2×10^−4^	>50 2.7 ± 0.2 0.02 ± 2×10^−4^	>50 27.0 ± 0.6 0.2 ± 2×10^−3^	18.7 ± 1.0 29.4 ± 1.0 0.07 ± 2×10^−3^	>50 >50 4.9 ± 0.3
**ML302A** **ML302** **ML302F**		4.8 ± 0.3 0.1 ± 9×10^−3^ 0.02 ± 2×10^−4^	9.6 ± 0.4 0.09 ± 2×10^−3^ 0.02 ± 3×10^−4^	21.3 ± 0.5 0.3 ± 0.01 0.08 ± 2×10^−3^	3.6 ± 0.7 0.06 ± 5×10^−3^ 0.04 ± 2×10^−3^	>50 15.6 ± 0.7 2.4 ± 0.1
**10**		0.5 ± 0.04	3.2 ± 0.2	0.6 ± 0.05	0.2 ± 0.02	>50
**6r**		3.2 ± 0.2	1.8 ± 3×10^−3^	>50	0.05 ± 1×10^−3^	1.7 ± 0.6
**6s**		0.06 ± 3×10^−3^	0.08 ± 2×10^−3^	0.2 ± 0.03	0.1 ± 3×10^−3^	1.1 ± 1.7

**Fig. 2:**
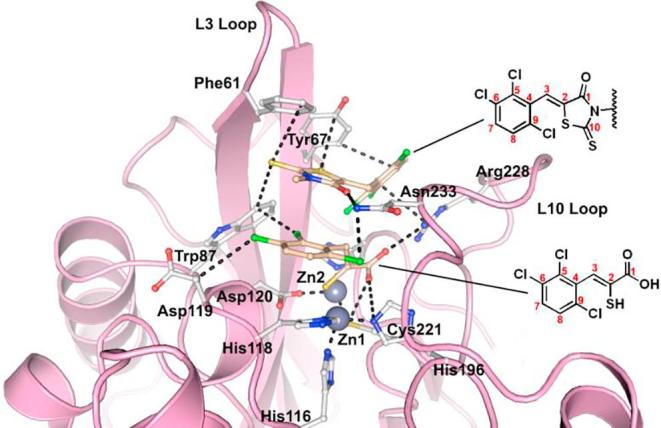
Prior crystallographic analysis revealed that **ML302** undergoes fragmentation to form the enethiol inhibitor **ML302F** (PDB ID: 4PVO),^24^ which coordinates to the di-Zn(II) containing active site. All figures are labelled using the BBL numbering scheme.

Aside from the previously reported formation of ternary complexes^24^ (Fig. 2), some of the results do, however, suggest the intact rhodanines may have inhibitory activity as precedented by work from Spicer et al.^53^ Interestingly, although the 2,4-dioxo-1,3-thiazolidin analogue (**10**) of **ML302** is less active than **ML302** against all tested MBLs, it did manifest activity against SPM-1, BcII, and VIM-2, being much less active against IMP-1 and particularly, NDM-1. We did not observe hydrolysis of **10** by NMR on the timescale of the inhibition studies, ^1^H NMR (700 MHz) in the presence or absence of NDM-1 MBL (Fig. S1). Although we cannot rule out the hydrolysis of **10** to form **ML302F** at low levels, these results suggest that further SAR studies on the intact rhodanine scaffold, or preferably more stable analogues of it, are of interest. One possibility is that the intact rhodanines can bind to the active site in a manner not involving metal chelation, as recently reported for another series of MBL inhibitors.^54^

The results (Table 1) reveal some SAR trends – some of the di-/tri-substituted amides and enethiols were clearly more active than the mono-substituted compounds. However, because the amides may be under-going hydrolysis/inhibiting *via* more than one mode of action, care must be taken in comparing the SAR for the two series. Thus, in most cases, the amides **5a-5e**, showed almost no inhibition against all the subclass B1 MBLs (IC_50_ values ∼50 or >50 µM), when the *para*-position of the phenyl ring was mono-functionalised with halogen or alkyl groups. By contrast, e.g., the mono-*ortho*-substituted **5f-5i** (IC_50_ values 2.0–52.1 µM), and di-*ortho*-substituted **5j** (IC_50_ values < 2.0 µM), and 2,3,6-trifluoro **5k** (IC_50_ values < 3.5 µM) analogues did manifest significant inhibition for all the tested B1 subclass MBLs, with the exception of NDM-1.

Similar trends were observed for the enethiols **6a-6L**, although IC_50_ values are mostly in the nanomolar range across all the MBLs tested. **6j** (IC_50_ values for SPM-1, IMP-1, BcII, VIM-2 and NDM-1 of 4.3 nM, 30 nM, 60 nM, 50 nM and 4.4 µM, respectively), inhibited with a similar potency compared to **ML302F** across the panel (IC_50_ values for SPM-1, IMP-1, BcII, VIM-2 and NDM-1 of 20 nM, 20 nM, 80 nM, 40 nM and 2.4 µM, respectively). The results with enethiols **6j** and **6L** led to further investigations on aromatic substitutions. The di-*ortho*-substituted **6m** (IC_50_ values from 10 nM to 2.1 µM), **6p** (IC_50_ values from 60 nM to 41.5 µM) and **6q** (IC_50_ values from 20 nM to 4.9 µM) showed much better inhibition compared to the 2,3-substituted (**6n**) (IC_50_ values from 200 nM to > 50 µM) or 2,5-substituted (**6o**) (IC_50_ values from 800 nM to 46.9 µM) analogues. Compounds with increased steric bulk, e.g. thianaphthene **6L** or **6r**, still manifested potent or moderately potent inhibition against most of the MBLs, with one clear exception in each case (NDM-1 with **6L** and BcII with **6r**).

We then synthesised and tested a set of analogues to investigate the importance of the different functional groups in the enethiols (**6a-q**, **ML302F**). The hydroxyl analogue of **6c**, i.e. **17** (**Table S1**), was near inactive (at 100 µM), as was the 2-methyloxazol-5(4H)-one (**16**), precursor of **17**, supporting the importance of the sulphur atom for binding and inhibition. The phosphoric acids **22** and **26** were also inactive, implying the importance of the carboxylate in inhibition (**Table S1**). The saturated analogues of **6a**, i.e. **13b** (IC_50_ values for SPM-1, IMP-1, BcII, VIM-2 and NDM-1; 70 nM, 50 nM, 1.4 µM, 70 nM and 38.7 µM, respectively) and its saturated truncated form, **13a** (IC_50_ values for SPM-1, IMP-1, BcII, VIM-2 and NDM-1 of 50 nM, 70 nM, 100 nM, 40 nM and 12.9 µM, respectively) manifested comparable potency against subclass B1 MBLs compared to **6a** (IC_50_ values for SPM-1, IMP-1, BcII, VIM-2 and NDM-1; 3 nM, 30 nM, 700 nM, 400 nM and 7.9 µM, respectively). However, **13a**/**13b** were much less active against CphA (IC_50_ values for **6a**, **13a** and **13b**; 2.1 µM, 71.0 µM and 130.0 µM, respectively). **13a** was more active than **13b** against BcII and NDM-1. The hydroxyl analogues, i.e. mandelic acid and 3-phenylactic acid, were inactive (at 100 µM), supporting the importance of the thiol for potent MBL inhibition in this series (Table 2). The observations support previous findings for the use of the α-mercaptocarboxylic acid motif for MBL binding/inhibition.^55^ Although relative acidity of the functional groups may be a factor, the increased inhibition observed for the (racemic) saturated α-mercaptocarboxylic acids (**13a**, **13b**) compared to the analogous enethiol (**6a**) could be a result of the different spatial relationship of the thiol and the carboxylate, enabling the saturated α-mercaptocarboxylic acids to bind better.

**Table 2:** IC_50_ values for the inhibition of MBLs by α-mercaptocarboxylic acids, and α-hydroxy-carboxylic acids and enethiols.

**Figure:**
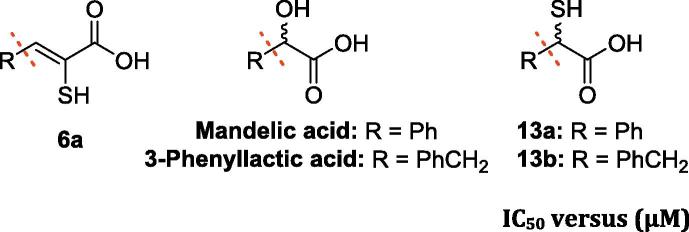

				IC_50_ versus (µM)			
**R**	**Compound**	**SPM-1**	**IMP-1**	**BcII**	**VIM-2**	**NDM-1**	**CphA**
	**6a**	0.3 ± 7×10^−3^	0.3 ± 0.03	0.7 ± 0.2	0.4 ± 0.02	7.9 ± 0.1	2.1 ± 0.02
	**13a**	0.05 ± 1×10^−3^	0.07 ± 3×10^−3^	0.1 ± 4×10^−3^	0.04 ± 1×10^−3^	12.9 ± 1.0	71.0 ± 1.0
	**13b**	0.07 ± 1×10^−3^	0.05 ± 2×10^−3^	1.4 ± 0.3	0.07 ± 2×10^−3^	38.7 ± 4.7	130.0 ± 10

	**Mandelic acid**	*NI*	*NI*	*NI*	*NI*	*NI*	*NI*
	**3-Phenyllactic acid**	*NI*	*NI*	*NI*	*NI*	*NI*	*NI*

*NI*: No observed inhibition at 100 µM.

All of the enethiols, except **6r** (as well as **5d, 5r,** which are intact rhodanines), were near inactive (at 200 µM) against a panel of SBLs (**Table S2**). The observed weak SBL inhibition by **5r** and **6r** (residual activities at 200 µM for TEM-1, CTX-M−15, AmpC and OXA-10; ∼6%, ∼40%, ∼18% and ∼47% and ∼1%, ∼65%, ∼41% and ∼ 47%, respectively) may be due to active site hydrophobic interactions involving the tri-aromatic ring system.

We then screened selected enethiols (**6a**, **6b**, **6g**, **6j**, **6k**, **6L**, **6m** and **ML302F**) in the presence of their amide analogues (**5a**, **5b**, **5g**, **5j**, **5k**, **5L**, **5m** and **ML302**) for potential inhibition *via* the formation of a ternary complex (Table 3). The use of a 1:1 mixture of **ML302** and **ML302F** (**ML302M)** manifested a > 20-fold increase in potency (IC_50_ = 1.8 nM) compared to the use of either **ML302** (IC_50_ = 60 nM) or **ML302F** (IC_50_ = 40 nM) individually against VIM-2, as observed previously.^12^ The mixture **ML302M** was also moderately more active against IMP-1 (IC_50_ = 3 nM), and more potent against CphA (IC_50_ = 20 nM) than **ML302** (IC_50_ = 90 nM, >50 µM, respectively) or **ML302F** alone (IC_50_ = 20 nM and 200 nM, respectively). However, the increased activity with the mixture was not observed in the inhibition of the other subclass B1 MBLs (BcII, SPM-1, NDM-1) tested, arguing against the formation of a ternary complex in these cases (Table 3). This observation is consistent with the results of our previous study,^24^ i.e. that a ternary complex can be accommodated by VIM-2, but not BcII, suggesting different binding modes for these two enzymes.^24^ Further, no evidence for enhanced inhibition relative to the enethiols alone was observed for any of the other tested mixtures, (**5a**, **5b**, **5g**, **5j**, **5k**, **5L**, **5m** and **6a**, **6b**, **6g**, **6j**, **6k**, **6L**, **6m**). Thus, the available evidence is that formation of ternary complex is limited to specific enethiol inhibitor-MBL combinations.

**Table 3:** Observed inhibition of MBLs by a 1:1 mixture of rhodanine amides (**5**) and enethiols (**6**) compared to their inhibition by the separate molecules.

**Compound/mixture**	**Enzyme**	**IC_50_ when R = (µM)**							
		**a**	**b**	**g**	**J**	**k**	**l**	**m**	**ML302/F**
Amide, **5**	**SPM-1**	>50	>50	9.8 ± 0.4	0.1 ± 4×10^−3^	1.5 ± 0.2	>50	0.2 ± 2×10^−3^	0.1 ± 9×10^−3^
Enethiol**, 6**		0.3 ± 7×10^−3^	0.3 ± 7×10^−3^	0.1 ± 3×10^−3^	4.3 × 10^−3^ ±2.9 × 10^−5^	0.1 ± 2×10^−3^	0.4 ± 0.02	0.01 ± 1×10^−4^	0.02 ± 2×10^−4^
**5 + 6** (1:1)		2.3 ± 0.09	42.4 ± 0.7	1.3 ± 0.2	0.1 ± 0.02	0. 4 ± 0.2	0.7 ± 0.3	0.02 ± 2×10^−4^	0.02 ± 2×10^−3^

Amide, **5**	**IMP-1**	22.7 ± 0.3	>50	7.6 ± 0.1	0.3 ± 0.01	1.6 ± 0.4	>50	0.2 ± 4×10^−3^	0.09 ± 2×10^−3^
Enethiol**, 6**		0.3 ± 0.03	0.2 ± 5×10^−3^	0.4 ± 0.01	0.03 ± 3×10^−4^	0.4 ± 0.02	0.7 ± 0.05	0.01 ± 6×10^−5^	0.02 ± 3×10^−4^
**5 + 6** (1:1)		0.2 ± 0.01	0.1 ± 3×10^−3^	0.4 ± 0.01	0.02 ± 3×10^−4^	0.2 ± 5×10^−3^	0.5 ± 0.03	7.0 × 10^−3^ ± 8×10^−5^	3.0 × 10^−3^ ± 4×10^−5^

Amide, **5**	**BcII**	>50	>50	>50	2.0 ± 0.2	3.5 ± 0.2	7.2 ± 3.3	1.5 ± 0.2	0.3 ± 0.01
Enethiol**, 6**		0.7 ± 0.2	0.7 ± 0.2	0.9 ± 0.1	0.06 ± 2×10^−3^	0.3 ± 0.01	3.1 ± 0.3	0.07 ± 3×10^−3^	0.08 ± 2×10^−3^
**5 + 6** (1:1)		1.8 ± 0.3	1 ± 0.1	3.2 ± 0.06	0.1 ± 0.01	0.6 ± 0.4	6.3 ± 0.4	0.03 ± 1×10^−3^	0.03 ± 1×10^−3^

Amide, **5**	**VIM-2**	16.4 ± 0.1	>50	2.0 ± 0.2	0.8 ± 0.1	2.6 ± 0.1	41.8 ± 3.6	0.3 ± 0.01	0.06 ± 5×10^−3^
Enethiol**, 6**		0.4 ± 0.02	0.4 ± 0.01	0.4 ± 0.02	0.05 ± 1×10^−3^	0.3 ± 0.01	0.5 ± 0.03	0.03 ± 2×10^−4^	0.04 ± 2×10^−3^
**5 + 6** (1:1)		0.2 ± 0.01	0.3 ± 0.01	0.4 ± 0.02	0.05 ± 1×10^−3^	0.2 ± 0.01	2.3 ± 0.2	0.02 ± 1×10^−4^	1.8 × 10^−3^ ± 3×10^−4^

Amide, **5**	**NDM-1**	26.7 ± 1.0	28.0 ± 1.0	>50	>50	40.1 ± 2.1	>50	28.4 ± 0.4	15.6 ± 0.7
Enethiol**, 6**		7.9 ± 0.1	3.7 ± 0.1	5.1 ± 0.5	4.4 ± 0.2	3.3 ± 0.1	11.1 ± 0.3	0.6 ± 0.1	2.4 ± 0.1
**5 + 6** (1:1)		18.5 ± 0.1	16.2 ± 0.5	33.8 ± 1.0	2.8 ± 0.4	28.6 ± 0.7	>50	1.7 ± 0.3	1.3 ± 0.4

Amide, **5**	**CphA**	>50	–	–	–	–	–	>50	>50
Enethiol**, 6**		2.1 ± 0.02	–	–	–	–	–	0.8 ± 0.02	0.2 ± 4×10^−3^
**5 + 6** (1:1)		2.7 ± 0.04	–	–	–	–	–	0.4 ± 0.01	0.02 ± 1×10^−3^

*Note*: that in most cases the mixture is of similar potency to the enethiol alone, but that in a few cases (notably ML302/ML302F) the mixture is more potent.

### 3.3 Structural studies

**Fig. 3:**
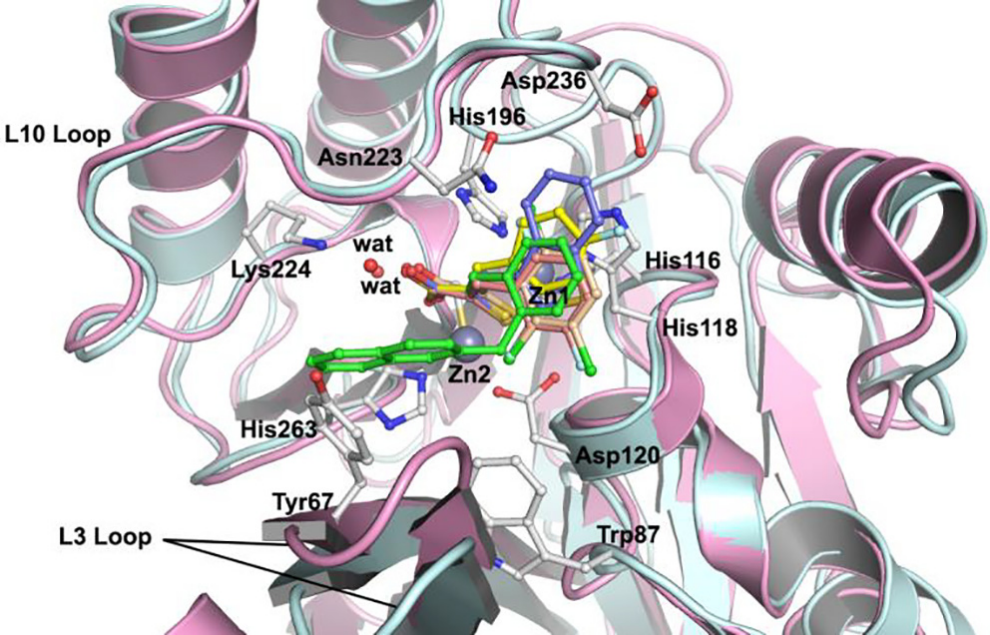
Superimposition of structures of BcII (turquoise) (PDB ID: 5JMX, 6EUM, 6EWE, 6F2N) with VIM-2 (pink) (PDB ID: 6EW3) showing the similarity in binding modes for **6c** (yellow), **6k** (salmon), **6L** (purple), **6s** (green), and **ML302F** (wheat). In each MBL the thiolate interacts with both Zn(II) ions and the inhibitor carboxylate interacts with Zn2 and (Lys-224 of BcII/Arg233 of VIM-2).

Previously, we have reported crystallographic studies of VIM-2 and the BcII MBLs in complex with **ML302F** and in the case of VIM-2, **ML302**.^24^ In the case of BcII, a single **ML302F** molecule was apparent at the active site.^24^ Unexpectedly, when **ML302** was co-crystallised with VIM-2, each of the two molecules in the asymmetric unit had **ML302F** chelating Zn(II) at the active site. However, an additional molecule of **ML302** that was apparent only near the active site of chain A (and not chain B), was positioned to interact with **ML302F**, *via* staggered π-stacking between the rhodanine ring of **ML302** and the 2,3,6-trichlorophenyl ring of **ML302F** (Fig. 2).^24^ In order to further investigate the mode of enethiol binding to MBLs in relation to our SAR results, we obtained four additional high resolution crystal structures of BcII co-crystallised with enethiols **6c** (Fig. S9, PDB ID: 5JMX), **6k** (Fig. S10, PDB ID: 6EUM), **6L** (Fig. S11, PDB ID: 6EWE), and **6s** (Fig. 3, PDB ID: 6F2N). We also obtained a new structure of VIM-2 in complex with **ML302F** (**Fig. S12**, PDB ID: 6EW3) using a low volume soaking method.^56^ (Note: The geometric restraints generated by GRADE for the C=C double bond length of the enethiols reported here are 1.4 Å, whereas that in our previously reported VIM-2:ML302F structure (PDB: 4PVO^24^) was slightly longer (1.5 Å) due to the geometric restraints output by ELBOW.^24^)

**Fig. 4:**
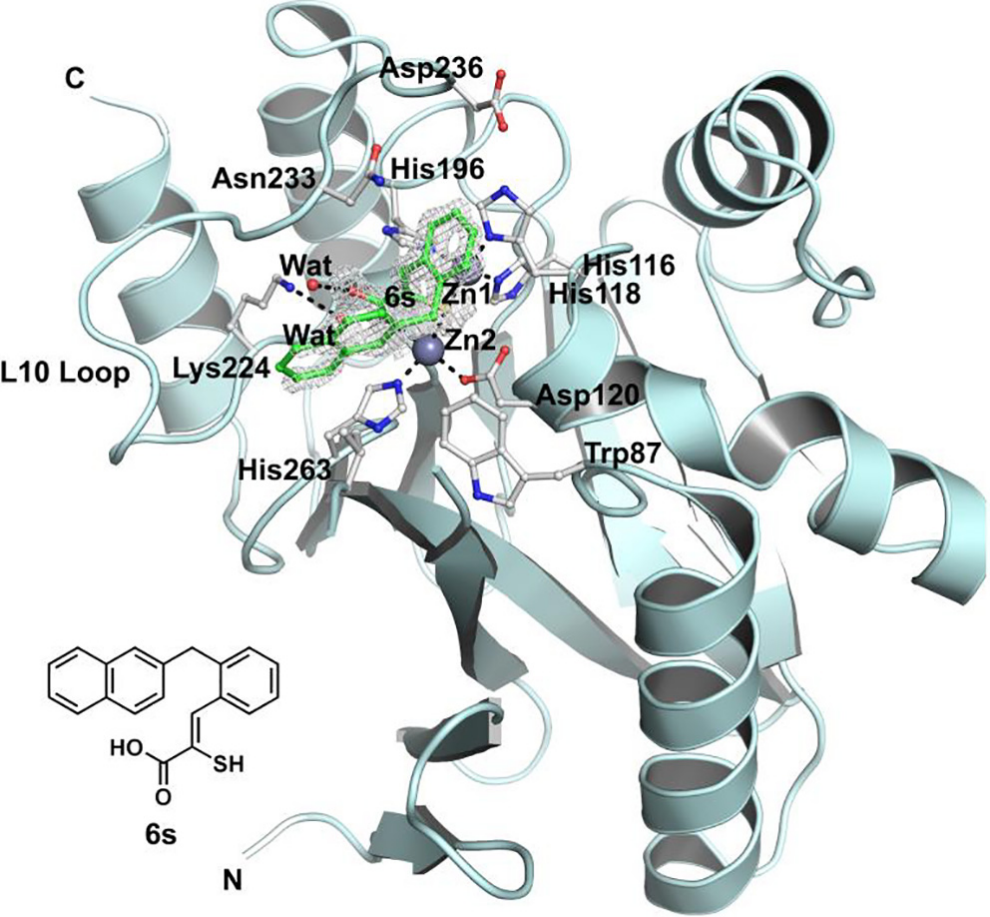
View from a crystal structure of BcII (turquoise) in complex with **6s** (green). Active site residues shown as ball-and-stick with atoms coloured C (white), O (red), N (blue), Zn (grey spheres), water (red spheres). Ligand interactions are indicated with black dashed lines. Ligand mFo-DFc OMIT maps contoured to 3.0 σ are shown as light grey mesh.

Comparison of the BcII structures in complex with the different enethiols reveal that the core enethiols have a remarkably similar binding mode to **ML302F**, with the thiol(ate) displacing the bridging water molecule normally located between the two Zn(II) ions (Zn1 and Zn2) and the inhibitor carboxylate ligating to Zn2. The enethiol linked phenyl rings of the inhibitors all occupy the same region of the active site (Fig. 3). As observed for binding of the products of MBL-catalysed β-lactam hydrolysis, in the structure of NDM-1 (PDB ID: 4EYF)^56^ (Fig. S13), one of the enethiol carboxylate oxygens is positioned to interact with Zn2. The other oxygen is positioned to interact with the N^ε^ amino group of Lys-224 (Fig. 4). As observed in the VIM-2:**ML302F** complex, the plane of the phenyl side chain on all of the enethiol inhibitors is rotated about the C3–C4 bond such that it is not co-planar with the plane of the enethiol alkene, likely hindering conjugation. For **ML302F** the skewed arrangement was thought to be, at least partially, caused by steric hindrance due to the *ortho* di-chloro substituents on the phenyl ring as proposed previously,^24^ Because enethiols without *ortho*-substituents are also observed to retain similar conformations (as evidenced by a crystal structure of BcII in complex with **6c**, Fig. S9), the *ortho*-substitution may not be an essential factor in obtaining potent inhibition by the enethiols.

Superimposing the structure of BcII in complex with **6s** and VIM-2 in complex with **ML302F**, implies that there may be a steric clash between the naphthalene side chain of **6s** and Tyr67 on the L3 loop of VIM-2, suggesting unfavourable binding. However, the BcII and VIM-2 IC_50_ values for **6s** are comparable (IC_50_ =  0.2 µM and 0.1 µM, respectively), indicating **6s** might adopt a different conformation when binding to VIM-2 and/or that it induces a conformational change of the VIM-2 L3 loop.

## 4 Conclusions

The overall results reveal that rhodanine derived species have potential as broad spectrum MBL inhibitors, which might be in part due to the proposal that enethiol carboxylate binding mimics that of β-lactam hydrolysis product (Fig. S13). Their capacity to inhibit SBLs and penicillin binding proteins appears more limited, at least among those compounds tested in this study.^24^, ^26^ Although the enethiols (**6a-6q**), which are derived by rhodanine hydrolysis, are the most potent of the series identified, the SAR on compounds with intact rhodanine ring structures suggests that rhodanine related heterocycles that do not chelate *via* a thiol/sulphur may also have potential as MBL inhibitors. Recent work on another series suggests that such compounds have potential to inhibit without active site metal chelation.^57^ The proposal of different binding modes for the enethiols (**6a-61**) and rhodanine amides (**5a-51**) is supported by the observation of only partially overlapping SAR trends for the two series.

We have previously reported structural evidence that **ML302**/**ML302F** can form a ternary complex with VIM-2.^24^ The SAR results presented here suggest that formation of such a ternary complex is not a general feature of rhodanine derived MBL inhibition and hence, although interesting, is unlikely to be a productive path for the development of broad spectrum clinically useful MBL inhibitors.

Rhodanines are often characterised as ‘difficult to progress’ and ‘promiscuous’ compounds.^57^, ^58^, ^59^ Our work reveals further complexities involved in interpreting assay results involving rhodanines. Despite their complex nature, one rhodanine is clinically approved for use in nerve damage due to diabetes mellitus (Epalrestat®, an aldose reductase inhibitor)^60^ and other rhodanine-related heterocycles are in development.^61^ The results presented here support the proposal that rhodanines (at least) have potential as promiscuous enzyme inhibitors/protein binders, in part owing to their tendency to undergo hydrolysis to products, including enethiols, which have potential to inhibit the multiple metallo-enzymes present in cells, including related MBL fold enzymes, which have important biological roles beyond antibiotic resistance including in nucleic acid repair and metabolism.^62^ Our results also imply that any medicinal chemistry studies employing rhodanine inhibitors should as a matter of course include testing of their hydrolysis products.
