# Supplementary material for: Structure activity relationship studies on rhodanines and derived enethiol inhibitors of metallo-β-lactamases
Source: Bioorg Med Chem. 2018 Jul 15;26(11):2928–36. doi: 10.1016/j.bmc.2018.02.043 (PMC6008492; doi:10.1016/j.bmc.2018.02.043)
Supplement: Supplementary data 1 [file mmc1.pdf]

Supplementary Information for:

Structure activity relationship studies on rhodanines and derived enethiol inhibitors of metallo-β-lactamases

Dong Zhang[a], Marios S. Markoulides[a], Dmitrijs Stepanovs[a,1], Anna M. Rydzik[a] , Ahmed El-Hussein[a,2] Corentin Bon[a,3], Jos J. A. G. Kamps[a], Klaus-Daniel Umland[a,4], Patrick M. Collins[b], Samuel T. Cahill[a], David Y. Wang[a], Frank von Delft[b,d,e], Timothy D. W. Claridge[a], Jürgen Brem[a], Michael A. McDonough[a], and Christopher J. Schofield[a,*]

[a] *Department of Chemistry, University of Oxford, Chemistry Research Laboratory, 12 Mansfield Road, Oxford,OX1 3TA, United Kingdom*
[b] *Diamond Light Source, Harwell Science and Innovation Campus, Didcot, OX11 0DE, United Kingdom*
[c] *The National Institute of Laser Enhanced Science, Cairo University, Egypt*
[d] *Structural Genomics Consortium (SGC), University of Oxford, Oxford, OX3 7DQ, UK3*
[e] *Department of Biochemistry, University of Johannesburg, Aukland Park, 2006, South Africa*

**To whom correspondence should be addressed. Email: christopher.schofield@chem.ox.ac.uk*
Telephone: +44 (0)1865 275 625. Fax: +44 (0)1865 285 002. Address: Chemistry Research Laboratory, 12 Mansfield Road, Oxford, OX1 3TA, United Kingdom

* Corresponding author. Tel.: +44 (0)1865 285 000; fax: +44 (0)1865 285 002; e-mail: christopher.schofield@chem.ox.ac.uk
[1] Present address: Latvian Institute of Organic Synthesis, Aizkraukles Str. 21 Riga, LV-1006, Latvia.
[2] Present address: Boehringer Ingelheim Pharma GmbH & Co KG Birkendorfer Strasse 65, 88397 Biberach an der Riss, Germany.
[3] Present address (on leave from): The National Institute of Laser Enhanced Science, Cairo University, Egypt.
[4] Present address: École polytechnique université, Paris saclay Route de Saclay, 91128 Palaiseau.
[5] Present address: Vifor Pharma, Flughofstrasse 61, 8152 Glattbrugg, Switzerland .

Table of Contents

1. Extended materials and methods S3

2. Compound characterisation S4

3. NMR ^{1}H and ^{13}C spectra S22

4. Extended biochemical results and tables S84

Table S1 Residual activities 16, 17, 22, 26 against MBLs S84
Figure S1 SBL inhibition S85
Figure S2 BCII IC_{50} traces S87
Figure S3 IMP-1 IC_{50} traces S89
Figure S4 NDM-1 IC_{50} traces S91
Figure S5 SPM-1 IC_{50} traces S93
Figure S6 VIM-2 IC_{50} traces S95
Figure S7 NMR reaction time course spectra S97
Figure S8 NMR reaction time course plot S98

5. Crystallography S99

Table S2 Crystallographic data processing and refinement statistics S99
Figure S9 Structure of BcII:**6c** S100
Figure S10 Structure of BcII:**6k** S101
Figures S11 Structure of BcII:**6l** S102
Figure S12 Structure of VIM-2:**ML302F** S103
Figures S13 MBL product binding S104

6. Author contributions S105

7. References S106

1. Extended Materials and Methods:

Reagents were obtained from commercial sources and either used as supplied or purified using appropriate standard procedures.

Solvents (including dry solvents) for chemical transformations, work-up and chromatography were from Sigma-Aldrich (Dorset, UK) at HPLC grade, and used without further purification.

Column chromatography was performed using prepacked SNAP columns on a Biotage SP1 Purification system (Uppsala, Sweden).

Thin-layer chromatography (TLC) was carried out using Merck aluminium plates coated with $HF_{254/366}$ silica gel. Visualization was performed with a 254 nm ultraviolet (UV) light source and/or by immersion in potassium permanganate or phosphomolybdic acid (PMA) solutions.

Microwave assisted reactions were performed using a Biotage Initiator™ microwave synthesizer in sealed vials.

Deuterated solvents were from Cambridge Isotopes, Apollo Scientific Ltd, and/or Sigma-Aldrich.

^{1}H and **^{13}C NMR** spectra were recorded either using a Bruker Avance III HD NanoBay 400 spectrometer, Bruker Avance III HD NanoBay 600 spectrometer, or using a Bruker Avance II 500 with 13C cryoprobe spectrometers. Residual non-deuterated solvent was used as the internal standard for ^{1}H NMR spectra, and a carbon signal of the solvent was used as the internal standard for ^{13}C NMR spectra. Chemical shifts (δ) are given in parts per million (ppm) downfield from tetramethylsilane (TMS). The resonance multiplicity patterns are described as singlet (s), broad singlet (br s), doublet (d), triplet (t), quartet (q), quintet (quin), multiplet (m), or a combination of these. Coupling constants (J) are quoted in hertz (Hz) to the nearest 0.5 Hz. Peak assignments were aided by COSY, HMQC, and/or HMBC whenever necessary.

High-resolution mass spectra were recorded either using a Bruker MicroTOF instrument using an ESI source and Time of Flight (TOF) analyser.

IR spectra were recorded on a Bruker Tensor 27 Attenuated Total Reflection (ATR) FT-IR spectrometer, and absorbance bands are quoted in cm^{-1}.

2. Compound Characterisation:

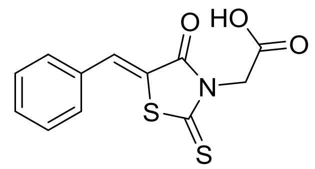

(5-Benzylidene-4-oxo-2-thioxothiazolidin-3-yl)acetic acid 3a: isolated as a yellow solid (85%). **mp** 217-236 °C. ***R$_f$*** = 0.60 (CH_2Cl_2 : MeOH, 9 : 1). **IR** ν_{max} (ATR)/cm^{-1}: 3160 (COO-H), 1731 (HOC=O), 1683 (NC=O), 1588 (SC=C). **HRMS** (ESI−): calculated for $C_{12}H_8NO_3S_2$ [M − H]$^-$ requires 277.9951; found 277.9955. **^{1}H NMR** (400 MHz, DMSO-d$_6$): δ_H 7.89 (s, 1H), 7.68 (d, *J* = 7.0 Hz, 2H), 7.47-7.63 (m, 3H), 4.68 (s, 2H). **^{13}C NMR** (100 MHz, DMSO-d$_6$) δ_C 193.77, 167.81, 166.95, 134.11, 133.35, 131.63, 131.22, 130.03, 122.55, 46.14.

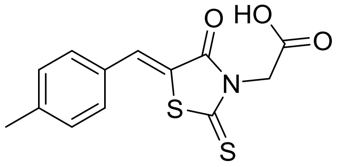

(5-(4-Methylbenzylidene)-4-oxo-2-thioxothiazolidin-3-yl)acetic acid 3b: isolated as a yellow solid (79%). **mp** 247-359 °C. ***R$_f$*** = 0.55 (CH_2Cl_2 : MeOH, 9 : 1). **IR** ν_{max} (ATR)/cm^{-1}: 3095 (COO-H), 1699 (HOC=O), 1656 (NC=O), 1594 (SC=C). **HRMS** (ESI−): calculated for $C_{13}H_{10}NO_3S_2$ [M − H]$^-$ requires 292.0108; found 292.0114. **^{1}H NMR** (400 MHz, DMSO-d$_6$) δ_H 7.70 (s, 1H), 7.53 (d, *J* = 9.0 Hz, 2H), 7.23 (d, *J* = 9.0 Hz, 2H), 4.71 (s, 2H), 2.55 (s, 3H). **^{13}C NMR** (100 MHz, DMSO-d$_6$) δ_C 193.41, 166.42, 165.76, 163.00, 134.14, 132.93, 132.13, 125.89, 123.23, 55.81, 45.88.

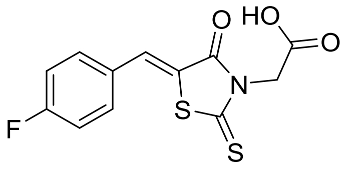

(5-(4-Fluorobenzylidene)-4-oxo-2-thioxothiazolidin-3-yl)acetic acid 3c: isolated as a yellow solid (82%). **mp** 225-235 °C. ***R$_f$*** = 0.55 (CH_2Cl_2 : MeOH, 9 : 1). **IR** ν_{max} (ATR)/cm^{-1}: 3172 (COO-H), 1732 (HOC=O), 1676 (NC=O), 1581 (SC=C). **HRMS** (ESI−): calculated for $C_{12}H_8FNO_3S_2$ [M]$^-$ requires 295.9857; found 295.9861. **^{1}H NMR** (400 MHz, DMSO-d$_6$) δ_H 7.91 (s, 1 H), 7.77 (dd, *J* = 8.7, 5.0 Hz, 2H), 7.43 (t, *J* = 8.5 Hz, 2H), 4.68 (s, 2H). **^{13}C NMR** (100 MHz, DMSO-d$_6$) δ_C 193.59, 167.70, 166.90, 163.75 (d, *J* = 251.5 Hz), 133.79 (d, *J* = 9.0 Hz), 133.00, 130.07 (d, *J* = 3.0 Hz), 122.23, 117.24 (d, *J* = 22.0 Hz), 46.14.

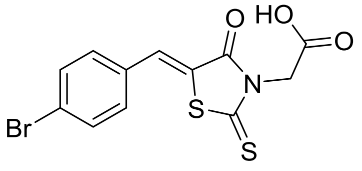

(5-(4-Bromobenzylidene)-4-oxo-2-thioxothiazolidin-3-yl)acetic acid 3d: isolated as a yellow solid (84%). **mp** 239-253 °C. ***R$_f$*** = 0.50 (CH_2Cl_2 : MeOH, 9 : 1). **IR** ν_{max} (ATR)/cm^{-1}: 3090 (COO-H), 1709 (HOC=O), 1589 (NC=O), 1587 (SC=C). **HRMS** (ESI−): calculated for $C_{12}H_7BrNO_3S_2$ [M − H]$^-$ requires 355.9056; found 355.9062. **^{1}H NMR** (400 MHz, DMSO-d$_6$) δ_H 7.85 (s, 1H), 7.76 (d, *J* = 8.5 Hz, 2H), 7.60 (d, *J* = 8.5 Hz, 2H), 4.72 (s, 2H); **^{13}C NMR** (100 MHz, DMSO-d$_6$) δ_C 193.38, 167.73, 166.81, 133.00, 132.94, 132.48, 125.37, 123.17, 45.75, 44.14.

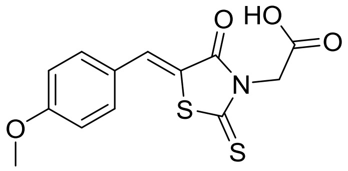

(5-(4-Methoxybenzylidene)-4-oxo-2-thioxothiazolidin-3-yl)acetic acid 3e: isolated as a yellow solid (80%). **mp** 187-243 °C. ***R**$_f$* = 0.50 (CH_2Cl_2 : MeOH, 9 : 1). **IR** ν_{max} (ATR)/cm^{-1}: 2963 (COO-H), 1679 (HOC=O), 1586 (NC=O), 1564 (SC=C). **HRMS** (ESI−): calculated for $C_{13}H_{10}NO_4S_2$ [M − H]$^-$ requires 308.0057; found 308.0062. **^{1}H NMR** (400 MHz, DMSO-d$_6$) δ_H 7.82 (s, 1H), 7.63 (d, *J* = 9.0 Hz, 2H), 7.12 (d, *J* = 9.0 Hz, 2H), 4.68 (s, 2H), 3.85 (s, 3H). **^{13}C NMR** (100 MHz, DMSO-d$_6$) δ_C 193.54 167.86, 166.99, 162.15, 134.33, 133.53, 125.89, 119.10, 115.66, 56.08, 45.96.

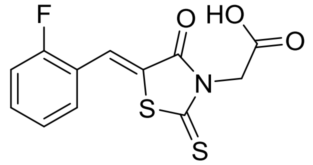

(5-(2-Fluorobenzylidene)-4-oxo-2-thioxothiazolidin-3-yl)acetic acid 3f: isolated as an orange solid (76%). **mp** 230-249 °C. ***R**$_f$* = 0.50 (CH_2Cl_2 : MeOH, 9 : 1). **IR** ν_{max} (ATR)/cm^{-1}: 2993 (COO-H), 1713 (HOC=O), 1604 (NC=O), 1397 (SC=C). **HRMS** (ESI−): calculated for $C_{12}H_7FNO_3S_2$ [M − H]$^-$ requires 295.9857; found 295.9860.

^{1}H NMR (400 MHz, DMSO-d$_6$) δ_H 7.82 (s, 1 H), 7.60 (t, *J* = 7.0 Hz, 2H), 7.37-7.47 (m, 2H), 4.69 (s, 2H). **^{13}C NMR** (100 MHz, DMSO-d$_6$) δ_C 193.46, 167.70, 166.71, 161.12 (d, *J* = 253.0 Hz), 134.02 (d, *J* = 9.0 Hz), 130.19, 126.15 (d, *J* = 3.5 Hz), 125.72-124.28 (m), 121.19 (d, *J* = 12.0 Hz), 116.84 (d, *J* = 21.5 Hz), 46.09.

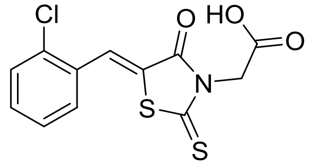

(5-(2-Chlorobenzylidene)-4-oxo-2-thioxothiazolidin-3-yl)acetic acid 3g: isolated as a yellow solid (77%). **mp** 234-237 °C. ***R**$_f$* = 0.55 (CH_2Cl_2 : MeOH, 9 : 1). **IR** ν_{max} (ATR)/cm^{-1}: 2926 (COO-H), 1708 (HOC=O), 1594 (NC=O), 1433 (SC=C). **HRMS** (ESI−): calcualted for $C_{12}H_7ClNO_3S_2$ [M − H]$^-$ requires 311.9561; found 311.9567. **^{1}H NMR** (400 MHz, DMSO-d$_6$) δ_H 7.95 (s, 1 H), 7.66 (dd, *J* = 6.0, 3.5 Hz, 1H), 7.58-7.62 (m, 1H), 7.52-7.57 (m, 1H), 4.72 (s, 2H). **^{13}C NMR** (100 MHz, DMSO-d$_6$) δ_C 193.54, 167.70, 166.58, 135.38, 132.99, 131.16, 130.99, 130.02, 128.83, 125.99, 45.86.

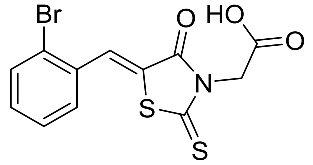

(5-(2-Bromobenzylidene)-4-oxo-2-thioxothiazolidin-3-yl)acetic acid 3h: isolated as a yellow solid (83%). **mp** 235-249 °C. ***R**$_f$* = 0.55 (CH_2Cl_2 : MeOH, 9 : 1). **IR** ν_{max} (ATR)/cm^{-1}: 3028 (COO-H), 1706 (HOC=O), 1594 (NC=O), 1429 (SC=C). **HRMS** (ESI−) calculated for $C_{12}H_7BrNO_3S_2$ [M − H]$^-$ requires 355.9056; found 355.9060.

^{1}H NMR (400 MHz, DMSO-*d*$_6$) δ_H 8.00 (s, 1H), 7.57 (d, *J* = 8.0 Hz, 1H), 7.33 (m, 3H), 4.27 (s, 2H). **^{13}C NMR** (100 MHz, DMSO-d$_6$) δ_C 193.20, 167.94, 165.43, 141.02, 137.48, 136.99, 130.14, 130.55, 129.98, 126.23, 105.01, 45.73.

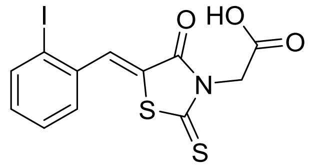

(5-(2-Iodobenzylidene)-4-oxo-2-thioxothiazolidin-3-yl)acetic acid 3i: isolated as a yellow solid (75%). **mp** 229-240 °C. ***R****f* = 0.55 (CH_2Cl_2 : MeOH, 9 : 1). **IR** ν_{max} (ATR)/cm^{-1}: 2938 (COO-H), 1703 (HOC=O), 1588 (NC=O), 1393 (SC=C). **HRMS** (ESI−): calculated for $C_{12}H_7NO_3S_2$ $[M - H]^-$ requires 403.8918; found 403.8925. **^{1}H NMR** (400 MHz, DMSO-d$_6$) δ_H 8.06 (d, *J* = 8.0 Hz, 1H), 7.82 (s, 1H), 7.51-7.62 (m, 2H), 7.20-7.28 (m, 1H), 4.71 (s, 2H). **^{13}C NMR** (100 MHz, DMSO-d$_6$) δ_C 193.69, 167.74, 166.43, 140.73, 136.71, 136.16, 132.87, 129.83, 129.49, 125.73, 104.00, 45.90.

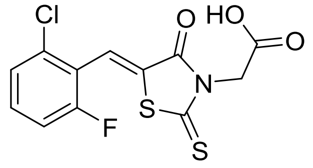

(5-(2-Chloro-6-fluorobenzylidene)-4-oxo-2-thioxothiazolidin-3-yl)acetic acid 3j: isolated as a yellow solid (84%). **mp** 237-250 °C. ***R****f* = 0.50 (CH_2Cl_2 : MeOH, 9 : 1). **IR** ν_{max} (ATR)/cm^{-1}: 3026 (COO-H), 1717 (HOC=O), 1598 (NC=O), 1393 (SC=C). **HRMS** (ESI−): calculated for $C_{12}H_6ClFNO_3S_2$ $[M - H]^-$ requires 329.9467; found 329.9473. **^{1}H NMR** (400 MHz, DMSO-d$_6$) δ_H 13.54 (br s, 1H), 7.79 (s, 1H), 7.56-7.66 (m, 1H), 7.48-7.55 (m, 1H), 7.41 (t, *J* = 9.5 Hz, 1H), 4.74 (s, 2H). **^{13}C NMR** (100 MHz, DMSO-d$_6$) δ_C 193.38 (d, *J* = 6.5 Hz), 167.70, 165.99, 159.44 (d, *J* = 253.0 Hz), 134.86 (d, *J* = 5.0 Hz), 133.84 (d, *J* = 10.0 Hz), 129.74 (d, *J* = 1.5 Hz), 126.83 (d, *J* = 3.0 Hz), 125.18, 120.22 (d, *J* = 18.0 Hz), 115.94 (d, *J* = 22.0 Hz), 45.63.

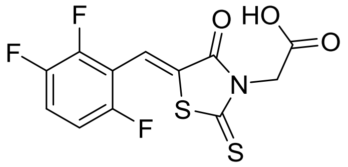

(4-Oxo-2-thioxo-5-(2,3,6-trifluorobenzylidene)thiazolidin-3-yl)acetic acid 3k: isolated as an orange solid (78%). **mp** 195-228 °C. ***R****f* = 0.50 (CH_2Cl_2 : MeOH, 9 : 1). **IR** ν_{max} (ATR)/cm^{-1}: 3212 (COO-H), 1709 (HOC=O), 1681 (NC=O), 1602 (SC=C). **HRMS** (ESI−): calculated for $C_{12}H_5F_3NO_3S_2$ $[M - H]^-$ requires 331.9668; found 331.9669. **^{1}H NMR** (400 MHz, DMSO-d$_6$) δ_H 7.72 (s, 1H), 7.76 (m, 1H), 7.38 (m, 1H), 4.75 (s, 2H). **^{13}C NMR** (100 MHz, DMSO-d$_6$) δ_C 193.31, 167.71, 166.22, 155.25 (d, *J* = 249.5 Hz), 148.96-146.01 (m), 148.48-145.33 (m), 130.05, 120.83 (dd, *J* = 19.5, 10.5 Hz), 119.90, 112.91 (dd, *J* = 24.5, 4.0 Hz), 112.75-112.28 (m), 46.23.

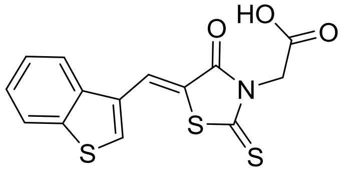

(5-(Benzo[b]thiophen-3-ylmethylene)-4-oxo-2-thioxothiazolidin-3-yl)acetic acid 3l: isolated as a yellow solid (76%). **mp** 242-266 °C. ***R****f* = 0.60 (CH_2Cl_2 : MeOH, 9 : 1). **IR** ν_{max} (ATR)/cm^{-1}: 3132 (COO-H), 1703 (HOC=O), 1702 (NC=O), 1587 (SC=C). **HRMS** (ESI−): calculated for $C_{14}H_8NO_4S_2$ $[M - H]^-$ requires 333.9672; found 333.9678. **^{1}H NMR** (400 MHz, DMSO-d$_6$) δ_H 7.95 (s, 1H), 7.49-7.71 (m, 4H), 7.55 (s, 1H), 4.72 (s, 2H). **^{13}C NMR** (100 MHz, DMSO-d$_6$) δ_C 193.54, 167.70, 166.58, 135.38, 132.99, 131.16, 130.99, 130.02, 128.83, 125.99, 45.86.

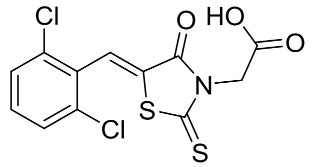

(5-(2,6-Dichlorobenzylidene)-4-oxo-2-thioxothiazolidin-3-yl)acetic acid 3m: isolated as a yellow solid (83%). **mp** 235-237 °C. $\boldsymbol{R_f}$ = 0.50 (CH_2Cl_2 : MeOH, 9 : 1). **IR** ν_{max} (ATR)/cm^{-1}: 2931 (COO-H), 1710 (HOC=O), 1600 (NC=O), 1442 (SC=C).

HRMS (ESI+): calculated for $C_{12}H_7Cl_2NO_3S_2Na$ [M + Na]$^+$ requires 369.9137; found 369.9137. ^{1}**H NMR** (400 MHz, DMSO-d$_6$) δ_H 7.86 (s, 1H), 7.71-7.60 (m, 2H), 7.54 (ddd, J = 9.0, 7.3, 0.7 Hz, 1H), 4.68 (s, 3H). ^{13}**C NMR** (100 MHz, DMSO-d$_6$) δ_C 193.23, 167.66, 165.45, 133.43, 132.74, 131.37, 131.20, 129.41, 129.32, 46.27.

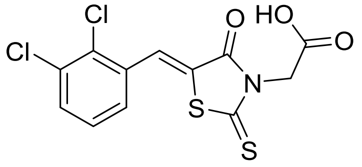

(5-(2,3-Dichlorobenzylidene)-4-oxo-2-thioxothiazolidin-3-yl)acetic acid 3n: isolated as a yellow solid (81%). **mp** 236-237 °C. $\boldsymbol{R_f}$ = 0.50 (CH_2Cl_2 : MeOH, 9 : 1). **IR** ν_{max} (ATR)/cm^{-1}: 2930 (COO-H), 1708 (HOC=O), 1598 (NC=O), 1440 (SC=C). **HRMS** (ESI−): calculated for $C_{12}H_6Cl_2NO_3S_2$ [M − H]$^-$ requires 345.9172; found 345.9167. ^{1}**H NMR** (400 MHz, DMSO-d$_6$) δ_H 7.83 (s, 1H), 7.70 (d, J = 8.5 Hz, 1H), 7.61 (dd, J = 8.5, 2.5 Hz, 1H), 7.55 (d, J = 2.5 Hz, 1H), 4.69 (s, 2H). ^{13}**C NMR** (100 MHz, DMSO-d$_6$) δ_C 193.01, 167.63, 166.34, 133.67, 133.16, 132.52, 132.38, 129.02, 127.81, 127.47, 46.10.

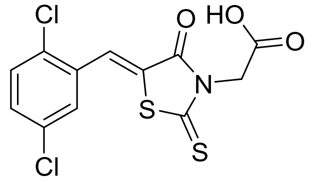

(5-(2,5-Dichlorobenzylidene)-4-oxo-2-thioxothiazolidin-3-yl)acetic acid 3o: isolated as a yellow solid (80%). **mp** 237-238 °C. $\boldsymbol{R_f}$ = 0.50 (CH_2Cl_2 : MeOH, 9 : 1). **IR** ν_{max} (ATR)/cm^{-1}: 2933 (COO-H), 1705 (HOC=O), 1595 (NC=O), 1447 (SC=C). **HRMS** (ESI−): calculated for $C_{12}H_6Cl_2NO_3S_2$ [M − H]$^-$ requires 345.9172; found 345.9167. ^{1}**H NMR** (400 MHz, DMSO-d$_6$) δ_H 13.59 (br s, 1H), 7.92 (s, 1H), 7.78 (dd, J = 7.5, 2.0 Hz, 1H), 7.59-7.50 (m, 2H), 4.75 (s, 2H). ^{13}**C NMR** (100 MHz, DMSO-d$_6$) δ_C 193.35, 167.65, 166.35, 133.68, 133.59, 132.97, 132.90, 129.57, 128.84, 128.51, 127.27, 45.56.

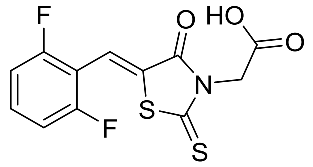

(5-(2,6-Difluorobenzylidene)-4-oxo-2-thioxothiazolidin-3-yl)acetic acid 3p: isolated as an orange solid (79%). **mp** 238-240 °C. $\boldsymbol{R_f}$ = 0.55 (CH_2Cl_2 : MeOH, 9 : 1). **IR** ν_{max} (ATR)/cm^{-1}: 2924 (COO-H), 1700 (HOC=O), 1575 (NC=O), 1438 (SC=C). **HRMS** (ESI−): calculated for $C_{12}H_6F_2NO_3S_2$ [M − H]$^-$ requires 313.9763; found 313.9760. ^{1}**H NMR** (400 MHz, DMSO-d$_6$) δ_H 7.71 (s, 1H), 7.66 (ddd, J = 8.5, 6.5, 2.0 Hz, 1H), 7.30 (t, J = 9.0 Hz, 2H). ^{13}**C NMR** (100 MHz, DMSO-d$_6$) δ_C 193.58, 167.74, 166.32, 159.98 (d, J = 253.0 Hz), 158.60 (d, J = 252.5 Hz), 134.55 (t, J = 11.0 Hz), 128.64, 121.09, 113.37-112.76 (m), 110.69 (t, J = 17.5 Hz), 45.92.

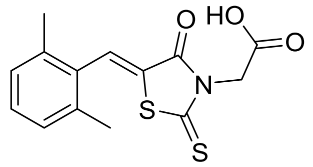

(5-(2,6-Dimethylbenzylidene)-4-oxo-2-thioxothiazolidin-3-yl)acetic acid 3q: isolated as a pale yellow solid (82%). **mp** 240-242 °C. $\boldsymbol{R_f}$ = 0.55 (CH_2Cl_2 : MeOH, 9 : 1). **IR** ν_{max} (ATR)/cm^{-1}: 2932 (COO-H), 1710 (HOC=O), 1588 (NC=O), 1449 (SC=C). **HRMS** (ESI−): calculated for $C_{14}H_{12}NO_3S_2$ [M − H]$^-$ requires 306.0264; found 306.0261. **^{1}H NMR** (400 MHz, DMSO-d$_6$) δ_H 13.53 (br s, 1H), 8.03 (s, 1H), 7.25 (m, 1H), 7.14 (d, J = 7.5 Hz, 2H), 4.73 (s, 2H), 2.22 (s, 6H). **^{13}C NMR** (100 MHz, DMSO-d$_6$) δ_C 194.17, 167.79, 165.13, 135.69, 135.63, 133.39, 129.64, 129.03, 128.33, 45.54, 20.17.

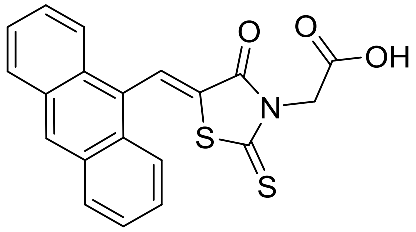

2-(5-(anthracen-9-ylmethylene)-4-oxo-2-thioxothiazolidin-3-yl)acetic acid 3r: isolated as an orange solid (90%). **mp** 240-242 °C. $\boldsymbol{R_f}$ = 0.55 (CH_2Cl_2 : MeOH, 9 : 1). **IR** ν_{max} (ATR)/cm^{-1}: 2967 (COO-H), 1707 (HOC=O), 1611(NC=O), 1401 (SC=C). **^{1}H NMR** (400 MHz, DMSO-d_6) δ_H 8.81 (s, 2 H), 8.22 (d, J=7.65 Hz, 2 H), 8.05 (d, J=8.55 Hz, 2 H), 7.59 - 7.71 (m, 5 H), 4.74 (s, 2 H) ppm. **^{13}C NMR** (101 MHz, DMSO-d_6) δ_C 194.0, 167.8, 132.3, 131.5, 131.2, 129.7, 128.5, 127.9, 127.4, 126.4, 125.4.

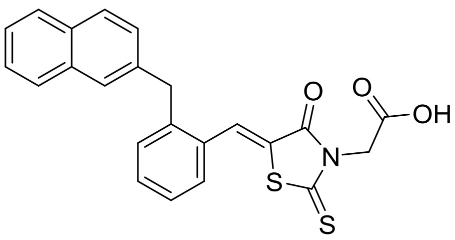

2-(5-(2-(naphthalen-2-ylmethyl)benzylidene)-4-oxo-2-thioxothiazolidin-3-yl)acetic acid 3s: isolated as an off white solid (88%). **mp** 251-259 °C. $\boldsymbol{R_f}$ = 0.55 (CH_2Cl_2 : MeOH, 9 : 1). **IR** ν_{max} (ATR)/cm^{-1}: 2899 (COO-H), 1721 (HOC=O), 1600 (NC=O), 1476 (SC=C). **HRMS** (ESI−): calculated for $C_{23}H_{16}NO_3S_2$ [M − H]$^-$ requires 418.0577; found 418.0570. **^{1}H NMR** (400 MHz, DMSO-d_6) δ_H 13.46 (s, 1H), 8.09 (s, 1H), 7.88 – 7.74 (m, 3H), 7.64 – 7.26 (m, 8H), 4.69 (s, 2H), 4.36 (s, 2H). **^{13}C NMR** (101 MHz, DMSO-d_6) δ_C 193.55, 167.21, 165.85, 141.94, 137.69, 133.06, 131.96, 131.79, 131.62, 131.43, 131.28, 128.28, 128.12, 127.66, 127.48, 127.39, 127.15, 126.59, 126.22, 125.59, 123.80, 44.95.

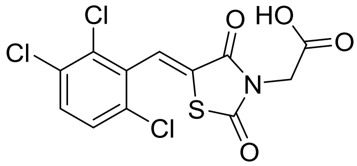

2-(2,4-Dioxo-5-(2,3,6-trichlorobenzylidene)thiazolidin-3-yl)acetic acid 9: isolated as a pale yellow solid (82%). **mp** 150-153 °C. $\boldsymbol{R_f}$ = 0.50 (CH_2Cl_2 : MeOH, 9 : 1). **IR** ν_{max} (ATR)/cm^{-1}: 2947 (COO-H), 1711 (HOC=O), 1600 (NC=O), 1458 (SC=C). **HRMS** (ESI+): calculated for $C_{12}H_5Cl_3NO_4S$ [M − H]$^-$ requires 363.9010; found 363.9008. **^{1}H NMR** (400 MHz, CD_3OD) δ_H 7.85 (s, 1H), 7.64 (dd, J = 9.0, 0.7 Hz, 1H), 7.52 (d, J = 9.0 Hz, 1H), 4.33 (s, 2H). **^{13}C NMR** (100 MHz, CD_3OD) δ_C 169.69, 167.53, 165.25, 134.62, 133.46, 133.30, 133.20, 132.90, 132.24, 130.50, 130.02, 43.21.

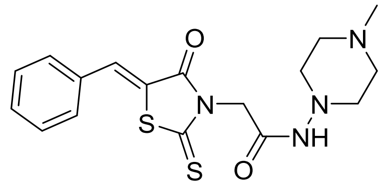

(5-Benzylidene-4-oxo-2-thioxothiazolidin-3-yl)-N-(4-methylpiperazin-1-yl)acetamide 5a: isolated as a yellow solid (35%). **mp** >255 °C (dec.). ***R**$_f$* = 0.40 (CH_2Cl_2 : MeOH, 9 : 1). **IR** ν_{max} (ATR)/cm^{-1}: 3215 (NH), 1713 (HNC=O), 1671 (NC=O), 1597 (SC=C). **HRMS** (ESI+): calculated for $C_{17}H_{21}N_4O_2S_2$ [M + H]$^+$ requires 377.1100; found 377.1100. **^{1}H NMR** (400 MHz, $CDCl_3$) δ_H 7.76 (s, 1H), 7.53 – 7.41 (m, 5H), 4.94 (s, 2H), 3.61 – 3.39 (m, 5H), 1.69 (5H). **^{13}C NMR** (100 MHz, $CDCl_3$) δ_C 193.80, 167.58, 162.23, 133.71, 133.43, 130.85, 130.71, 129.42, 123.12, 46.18, 45.07, 43.63, 26.42, 25.48, 24.49.

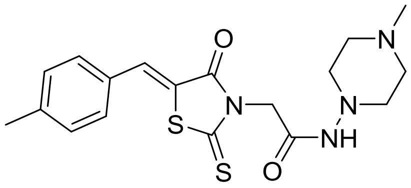

(5-(4-Methylbenzylidene)-4-oxo-2-thioxothiazolidin-3-yl)-N-(4-methylpiperazin-1-yl)acetamide 5b: isolated as a yellow solid (37%). **mp** >248 °C (dec.). ***R**$_f$* = 0.40 (CH_2Cl_2 : MeOH, 9 : 1). **IR** ν_{max} (ATR)/cm^{-1}: 3209 (NH), 1712 (HNC=O), 1670 (NC=O), 1595 (SC=C). **HRMS** (ESI+): calculated for $C_{18}H_{23}N_4O_2S_2$ [M + H]$^+$ requires 391.1257; found 391.1256. **^{1}H NMR** (600 MHz, DMSO-d$_6$) δ_H 9.07 (s, 1H), 7.84 (s, 1H), 7.58 (d, *J* = 8.0 Hz, 2H), 7.40 (d, *J* = 8.0 Hz, 2H), 4.93 (s, 2H), 3.20-2.57 (m, 8H), 2.39 (s, 3H), 2.22 (s, 3H). **^{13}C NMR** (150 MHz, DMSO-d$_6$) δ_C 193.21, 167.03, 166.66, 141.70, 133.77, 133.59, 130.83, 130.78, 130.19, 129.29, 129.09, 54.97, 54.18, 45.18, 44.91, 21.16.

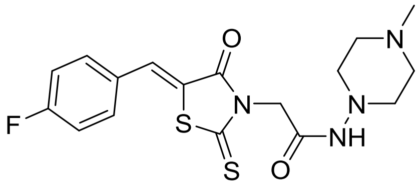

(4-Fluorobenzylidene)-4-oxo-2-thioxothiazolidin-3-yl)-N-(4-methylpiperazin-1-yl)acetamide 5c: isolated as an orange solid (35%). **mp** >266 °C (dec.). ***R**$_f$* = 0.35 (CH_2Cl_2 : MeOH, 9 : 1). **IR** ν_{max} (ATR)/cm^{-1}: 3213 (NH), 1712 (HNC=O), 1669 (NC=O), 1585 (SC=C). **HRMS** (ESI+): calculated for $C_{17}H_{20}FN_4O_2S_2$ [M + H]$^+$ requires 395.1006; found 395.1004. **^{1}H NMR** (600 MHz, DMSO-d$_6$) δ_H 9.06 (s, 1H), 7.89 (s, 1H), 7.79-7.75 (m, 2H), 7.46-7.40 (m, 2H), 4.93 (s, 2H), 3.12-2.30 (m, 8H), 2.18 (s, 3H). **^{13}C NMR** (150 MHz, DMSO-d$_6$) δ_C 193.83, 167.08, 166.56, 162.56 (d, *J* = 105.0 Hz), 133.73 (d, *J* = 9.0 Hz), 132.84, 130.11, 122.42 (d, *J* = 2.5 Hz), 117.25 (d, *J* = 22.0 Hz), 55.64, 54.80, 45.87, 45.45.

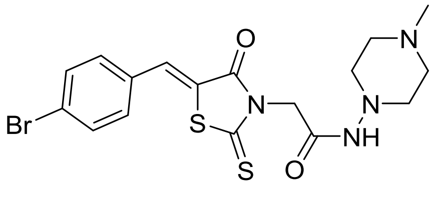

(5-(4-Bromobenzylidene)-4-oxo-2-thioxothiazolidin-3-yl)-N-(4-methylpiperazin-1-yl)acetamide 5d: isolated as a pale yellow solid (36%). **mp** >267 °C (dec.). ***R**$_f$* = 0.35 (CH_2Cl_2 : MeOH, 9 : 1). **IR** ν_{max} (ATR)/cm^{-1}: 3212 (NH), 1712 (HNC=O), 1670 (NC=O), 1598 (SC=C). **HRMS** (ESI+): calculated for $C_{17}H_{20}BrN_4O_2S_2$ [M + H]$^+$ requires 455.0206; found 455.0202. **^{1}H NMR** (600 MHz, $CDCl_3$) δ_H 7.69 (s, 1H), 7.62 (d, *J* = 8.5 Hz, 2H), 7.37 (d, *J* = 8.5 Hz, 2H), 6.36 (s, 1H), 5.08 (s, 2H), 3.27 – 2.55 (m, 8H), 2.32 (s, 3H). **^{13}C NMR** (150 MHz, $CDCl_3$) δ_C 193.10, 167.54, 166.99, 132.82, 132.36, 132.25, 131.94, 125.61, 123.99, 56.56, 54.62, 45.77, 45.05.

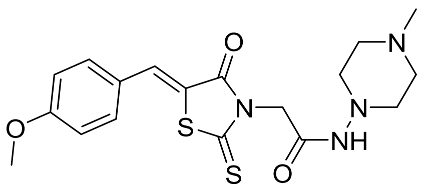

(5-(4-Methoxybenzylidene)-4-oxo-2-thioxothiazolidin-3-yl)-N-(4-methylpiperazin-1-yl)acetamide 5e: isolated as a yellow solid (31%). **mp** >253 °C (dec.). $\boldsymbol{R_f}$ = 0.35 (CH_2Cl_2 : MeOH, 9 : 1). **IR** ν_{max} (ATR)/cm^{-1}: 3215 (NH), 1708 (HNC=O), 1667 (NC=O), 1583 (SC=C). **HRMS** (ESI+): calculated for $C_{18}H_{23}N_4O_3S_2$ [M + H]$^+$ requires 407.1206; found 407.1201. ^{1}**H NMR** (600 MHz, CD_2Cl_2) δ_H 7.71 (s, 1H), 7.49 (d, J = 9.0 Hz, 2H), 7.01 (d, J = 9.0 Hz, 2H), 5.03 (s, 2H), 3.86 (s, 3H), 3.11-2.58 (m, 8H), 2.24 (s, 3H). ^{13}**C NMR** (150 MHz, CD_2Cl_2) δ_C 194.34, 168.03, 167.38, 162.43, 134.06, 133.29, 126.51, 120.37, 115.44, 56.65, 56.10, 55.09, 45.96, 45.53.

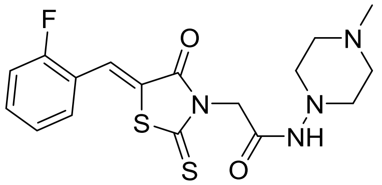

(5-(2-Fluorobenzylidene)-4-oxo-2-thioxothiazolidin-3-yl)-N-(4-methylpiperazin-1-yl)acetamide 5f: isolated as an orange solid (31%). **mp** >269 °C (dec.). $\boldsymbol{R_f}$ = 0.35 (CH_2Cl_2 : MeOH, 9 : 1). **IR** ν_{max} (ATR)/cm^{-1}: 3211 (NH), 1717 (HNC=O), 1670 (NC=O), 1605 (SC=C). **HRMS** (ESI+): calculated for $C_{17}H_{20}FN_4O_2S_2$ [M + H]$^+$ requires 395.1006; found 395.1003. ^{1}**H NMR** (600 MHz, DMSO-d$_6$) δ_H 9.13 (s, 1H), 7.81 (s, 1H), 7.61 (ddt, J = 10.5, 6.0, 1.5 Hz, 2H), 7.42 (td, J = 8.5, 8.0, 3.0 Hz, 2H), 4.95 (s, 2H), 3.06-2.57 (m, 8H), 2.27 (s, 3H). ^{13}**C NMR** (150 MHz, DMSO-d$_6$) δ_C 193.22, 167.30, 166.08, 160.63 (d, J = 253.0 Hz), 133.52 (d, J = 9.0 Hz), 129.69, 125.68, 124.84 (d, J = 10.5 Hz), 124.42 (d, J = 6.0 Hz), 120.75 (d, J = 12.0 Hz), 116.38 (d, J = 21.5 Hz), 54.58, 53.84, 46.28, 45.01.

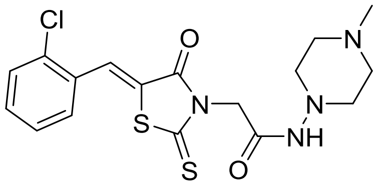

(5-(2-Chlorobenzylidene)-4-oxo-2-thioxothiazolidin-3-yl)-N-(4-methylpiperazin-1-yl)acetamide 5g: isolated as a yellow solid (36%). **mp** >267 °C (dec.). $\boldsymbol{R_f}$ = 0.35 (CH_2Cl_2 : MeOH, 9 : 1). **IR** ν_{max} (ATR)/cm^{-1}: 3219 (NH), 1721 (HNC=O), 1672 (NC=O), 1600 (SC=C). **HRMS** (ESI+): calculated for $C_{17}H_{20}ClN_4O_2S_2$ [M + H]$^+$ requires 411.0711; found 411.0707. ^{1}**H NMR** (600 MHz, DMSO-d$_6$) δ_H 9.10 (s, 1H), 7.96 (s, 1H), 7.71-7.66 (m, 1H), 7.65-7.60 (m, 1H), 7.59-7.53 (m, 2H), 4.94 (s, 2H), 3.01-2.63 (m, 8H), 2.21 (s, 3H). ^{13}**C NMR** (150 MHz, DMSO-d$_6$) δ_C 193.78, 166.79, 166.51, 135.27, 132.95, 131.24, 130.99, 129.99, 128.87, 128.58, 126.27, 55.49, 54.68, 45.70, 45.49.

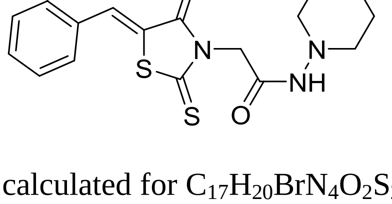

(5-(2-Bromobenzylidene)-4-oxo-2-thioxothiazolidin-3-yl)-N-(4-methylpiperazin-1-yl)acetamide 5h: isolated as a pale yellow solid (32%). **mp** >259 °C (dec.). $\boldsymbol{R_f}$ = 0.40 (CH_2Cl_2 : MeOH, 9 : 1). **IR** ν_{max} (ATR)/cm^{-1}: 3210 (NH), 1710 (HNC=O), 1659 (NC=O), 1591 (SC=C). **HRMS** (ESI+): calculated for $C_{17}H_{20}BrN_4O_2S_2$ [M + H]$^+$ requires 455.0206; found 455.0204. ^{1}**H NMR** (600 MHz, DMSO-d$_6$) δ_H 9.64 (s, 0H), 7.66 (d, J = 5.0 Hz, 1H), 7.60 (d, J = 7.4 Hz, 1H), 7.40 – 7.31 (m, 2H), 7.22 (d, J = 6.6

Hz, 1H), 4.77 (s, 1H), 3.46 – 2.62 (m, 11H), 2.26 (s, 2H). **^{13}C NMR** (150 MHz, DMSO-d_6) δ_C 193.32, 166.18, 162.07, 133.74, 132.37, 130.85, 129.55, 128.85, 125.84, 125.67, 125.62, 51.92, 50.66, 45.30, 44.83.

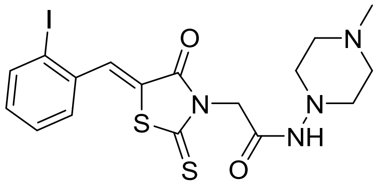

(5-(2-Iodobenzylidene)-4-oxo-2-thioxothiazolidin-3-yl)-N-(4-methylpiperazin-1-yl)acetamide 5i: isolated as a yellow solid (39%). **mp** >250 °C (dec.). ***R$_f$*** = 0.40 (CH_2Cl_2 : MeOH, 9 : 1). **IR** ν_{max} (ATR)/cm^{-1}: 3215 (NH), 1711 (HNC=O), 1672 (NC=O), 1592 (SC=C). **HRMS** (ESI+): calculated for $C_{17}H_{20}IN_4O_2S_2$ [M + H]$^+$ requires 503.0067; found 503.0062. **^{1}H NMR** (600 MHz, DMSO-d_6) δ_H 9.08 (s, 1H), 8.08 (t, *J* = 7.5 Hz, 1H), 7.76 (s, 1H), 7.64-7.51 (m, 1H), 7.26 (m, 2H), 4.94 (s, 2H), 3.02-2.58 (m, 8H), 2.18 (s, 3H). **^{13}C NMR** (150 MHz, DMSO-d_6) δ_C 193.70, 166.83, 166.53, 140.68, 136.43, 135.35, 132.60, 129.81, 129.39, 126.64, 103.69, 55.63, 54.79, 45.86, 45.49.

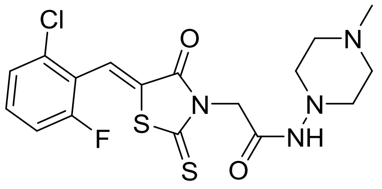

(5-(2-Chloro-6-fluorobenzylidene)-4-oxo-2-thioxothiazolidin-3-yl)-N-(4-methylpiperazin-1-yl)acetamide 5j: isolated as a yellow solid (35%). **mp** >261 °C (dec.). ***R$_f$*** = 0.35 (CH_2Cl_2 : MeOH, 9 : 1). **IR** ν_{max} (ATR)/cm^{-1}: 3211 (NH), 1723 (HNC=O), 1675 (NC=O), 1599 (SC=C). **HRMS** (ESI+): calculated for $C_{17}H_{19}ClFN_4O_2S_2$ [M + H]$^+$ requires 429.0616; found 429.0610. **^{1}H NMR** (600 MHz, DMSO-d_6) δ_H 9.09 (s, 1H), 7.78 (s, 1H), 7.59-7.65 (m, 1H), 7.54 (d, *J* = 8.0 Hz, 1H), 7.45 (t, *J* = 9.0 Hz, 1H), 4.92 (s, 2H), 2.91 (br s, 4H), 2.71 (br s, 4H), 2.18 (s, 3H). **^{13}C NMR** (150 MHz, DMSO-d_6) δ_C 193.16 (d, *J* = 6.2 Hz), 166.03, 165.74, 160.75 (d, *J* = 287.4 Hz), 134.27 (d, *J* = 5.0 Hz), 133.32 (d, *J* = 10.1 Hz), 129.67, 126.37 (d, *J* = 3.1 Hz), 124.26, 119.88 (d, *J* = 18.0 Hz), 115.50 (d, *J* = 22.1 Hz), 55.13, 54.28, 45.35, 45.13.

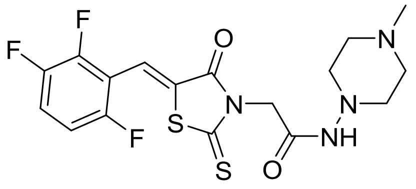

(4-Methylpiperazin-1-yl)-2-(4-oxo-2-thioxo-5-(2,3,6-trifluorobenzylidene)thiazolidin-3-yl)acetamide 5k: isolated as an orange solid (35%). **mp** >259 °C (dec.). ***R$_f$*** = 0.35 (CH_2Cl_2 : MeOH, 9 : 1). **IR** ν_{max} (ATR)/cm^{-1}: 3209 (NH), 1717 (HNC=O), 1668 (NC=O), 1596 (SC=C). **HRMS** (ESI+): calculated for $C_{17}H_{18}F_3N_4O_2S_2$ [M + H]$^+$ requires 431.0818; found 431.0815. **^{1}H NMR** (600 MHz, DMSO-d_6) δ_H 9.10 (s, 1H), 7.75 (qd, *J* = 9.5, 5.0 Hz, 1H), 7.68 (s, 1H), 7.37 (tdd, *J* = 9.5, 4.0, 2.0 Hz, 1H), 4.93 (s, 2H), 3.00-2.33 (m, 8H), 2.19 (s, 3H). **^{13}C NMR** (150 MHz, DMSO-d_6) δ_C 193.56, 166.46, 162.15, 157.08-154.13 (m), 147.49 (ddd, *J* = 254.5, 15.5, 7.5 Hz), 147.12 (ddd, *J* = 243.5, 12.5, 3.5 Hz), 130.31, 120.80 (dd, *J* = 19.5, 10.5 Hz), 119.85, 112.91 (ddd, *J* = 24.0, 7.0, 4.0 Hz), 112.63 (dd, *J* = 20.0, 14.0 Hz), 55.56, 54.74, 45.78, 45.65.

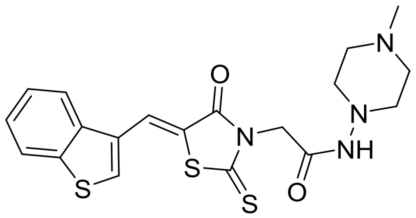

5-(Benzo[b]thiophen-3-ylmethylene)-4-oxo-2-thioxothiazolidin-3-yl)-N-(4-methylpiperazin-1-yl)acetamide 5l: isolated as a yellow solid (31%). **mp** >271 °C (dec.). $\boldsymbol{R_f}$ = 0.40 (CH_2Cl_2 : MeOH, 9 : 1). **IR** ν_{max} (ATR)/cm^{-1}: 3216 (NH), 1708 (HNC=O), 1674 (NC=O), 1598 (SC=C). **HRMS** (ESI+): calculated for $C_{19}H_{21}N_4O_2S_3$ $[M + H]^+$ requires 433.0821; found 433.0815. ^{1}**H NMR** (600 MHz, DMSO-d_6) δ_H 9.08 (s, 1H), 8.28 (s, 1H), 8.24-8.20 (m, 1H), 8.16-8.12 (m, 1H), 8.11 (s, 1H), 7.58-7.50 (m, 2H), 4.96 (s, 2H), 3.04-2.59 (m, 8H), 2.20 (s, 3H). ^{13}**C NMR** (150 MHz, DMSO-d_6) δ_C 193.66, 166.81, 166.63, 139.61, 138.09, 133.19, 129.68, 126.23, 125.93, 123.82, 123.74, 123.69, 122.30, 55.57, 54.75, 45.81, 45.45.

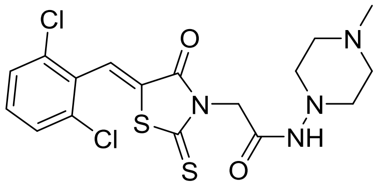

(5-(2,6-Dichlorobenzylidene)-4-oxo-2-thioxothiazolidin-3-yl)-N-(4-methylpiperazin-1-yl)acetamide 5m: isolated as a yellow solid (33%). **mp** >267 °C (dec.). $\boldsymbol{R_f}$ = 0.35 (CH_2Cl_2 : MeOH, 9 : 1). **IR** ν_{max} (ATR)/cm^{-1}: 3220 (NH), 1723 (HNC=O), 1675 (NC=O), 1603 (SC=C). **HRMS** (ESI+): calculated for $C_{17}H_{19}Cl_2N_4O_2S_2$ $[M + H]^+$ requires 445.0321; found 445.0315. ^{1}**H NMR** (400 MHz, DMSO-d_6) δ_H 9.38 (s, 1H), 7.83 (s, 1H), 7.65-7.60 (d, *J* = 7.5 Hz, 2H), 7.52 (t, *J* = 9.0 Hz, 1H), 4.98 (s, 2H), 3.41-2.86 (m, 8H), 2.68 (s, 3H). ^{13}**C NMR** (100 MHz, DMSO-d_6) δ_C 193.47, 166.79, 165.68, 133.45, 132.72, 131.22, 129.41, 128.87, 120.67, 52.90, 51.53, 46.02, 45.49.

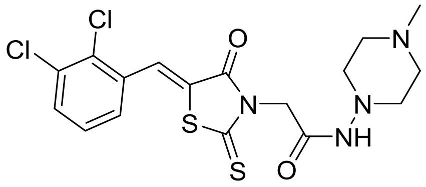

(5-(2,3-Dichlorobenzylidene)-4-oxo-2-thioxothiazolidin-3-yl)-N-(4-methylpiperazin-1-yl)acetamide 5n: isolated as a yellow solid (37%). **mp** >260 °C (dec.). $\boldsymbol{R_f}$ = 0.35 (CH_2Cl_2 : MeOH, 9 : 1). **IR** ν_{max} (ATR)/cm^{-1}: 3225 (NH), 1720 (HNC=O), 1677 (NC=O), 1599 (SC=C). **HRMS** (ESI+): calculated for $C_{17}H_{19}Cl_2N_4O_2S_2$ $[M + H]^+$ requires 445.0321; found 445.0315. ^{1}**H NMR** (400 MHz, DMSO-d_6) δ_H 9.41 (s, 1H), 7.83 (s, 1H), 7.70 (d, *J* = 8.5 Hz, 1H), 7.62 (dd, *J* = 8.5, 2.5 Hz, 1H), 7.56 (d, *J* = 2.5 Hz, 1H), 5.02 (s, 2H), 3.27-2.95 (m, 8H), 2.77 (s, 3H). ^{13}**C NMR** (100 MHz, DMSO-d_6) δ_C 192.83, 166.11, 166.06, 162.20, 133.18, 132.72, 132.09, 131.95, 128.58, 127.62, 127.03, 120.72, 52.12, 50.77, 45.41, 44.95.

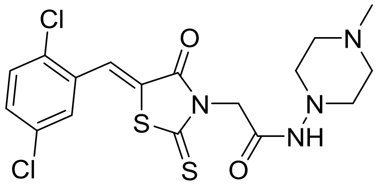

(5-(2,5-Dichlorobenzylidene)-4-oxo-2-thioxothiazolidin-3-yl)-N-(4-methylpiperazin-1-yl)acetamide 5o: isolated as a yellow solid (34%). **mp** >260 °C (dec.). $\boldsymbol{R_f}$ = 0.35 (CH_2Cl_2 : MeOH, 9 : 1). **IR** ν_{max} (ATR)/cm^{-1}: 3220 (NH), 1723 (HNC=O), 1681 (NC=O), 1597 (SC=C).

HRMS (ESI+): calculated for $C_{17}H_{19}Cl_2N_4O_2S_2$ $[M + H]^+$ requires 445.0321; found 445.0315. ^{1}**H NMR** (400 MHz, DMSO-d_6) δ_H 9.41 (s, 1H), 7.91 (s, 1H), 7.79 (d, *J* = 7.0 Hz, 1H), 7.61-7.50 (m, 2H), 5.02 (s,

2H), 3.19-2.91 (m, 8H), 2.78 (s, 3H). **^{13}C NMR** (100 MHz, DMSO-d$_6$) δ_C 193.62, 166.75, 166.64, 162.73, 133.67, 132.90, 129.62, 128.56, 128.50, 127.74, 127.59, 53.10, 52.65, 45.85, 45.36.

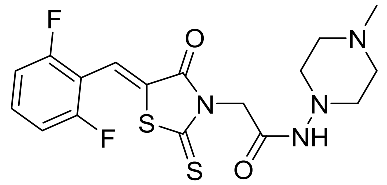

(5-(2,6-Difluorobenzylidene)-4-oxo-2-thioxothiazolidin-3-yl)-N-(4-methylpiperazin-1-yl)acetamide 5p: isolated as an orange solid (35%). **mp** >268 °C (dec.). ***R$_f$*** = 0.35 (CH_2Cl_2 : MeOH, 9 : 1). **IR** ν_{max} (ATR)/cm^{-1}: 3229 (NH), 1731 (HNC=O), 1696 (NC=O), 1602(SC=C). **HRMS** (ESI+): calculated for $C_{17}H_{19}F_2N_4O_2S_2$ [M + H]$^+$ requires 413.0912; found 413.0907. **^{1}H NMR** (400 MHz, DMSO-d$_6$) δ_H 9.87 (s, 1H), 7.72 (s, 1H), 7.71-7.62 (m, 1H), 7.32 (td, *J* = 9.0, 1.0 Hz, 2H), 4.99 (s, 2H), 3.49-2.89 (m, 8H), 2.69 (s, 3H). **^{13}C NMR** (100 MHz, DMSO-d$_6$) δ_C 193.79, 166.81, 166.54, 160.67 (d, *J* = 393.0 Hz), 134.40, 129.03 (d, *J* = 19.0 Hz), 120.88 (d, *J* = 14.8 Hz), 113.03 (d, *J* = 24.5 Hz), 110.75, 52.53, 51.36, 45.95, 45.51.

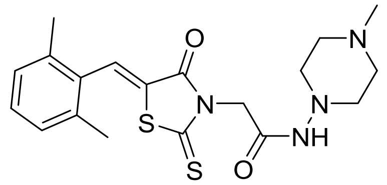

(5-(2,6-Dimethylbenzylidene)-4-oxo-2-thioxothiazolidin-3-yl)-N-(4-methylpiperazin-1-yl)acetamide 5q: isolated as a pale yellow solid (31%). **mp** >265 °C (dec.). ***R$_f$*** = 0.40 (CH_2Cl_2 : MeOH, 9 : 1). **IR** ν_{max} (ATR)/cm^{-1}: 3218 (NH), 1715 (HNC=O), 1677 (NC=O), 1586 (SC=C). **HRMS** (ESI+): calculated for $C_{19}H_{25}N_4O_2S_2$ [M + H]$^+$ requires 405.1413; found 405.1410. **^{1}H NMR** (400 MHz, DMSO-d$_6$) δ_H 9.30 (s, 1H), 8.00 (s, 1H), 7.25 (t, *J* = 7.5 Hz, 1H), 7.15 (d, *J* = 7.5 Hz, 2H), 4.98 (s, 2H), 3.27-2.73 (m, 8H), 2.55 (s, 3H). **^{13}C NMR** (100 MHz, DMSO-d$_6$) δ_C 194.43, 166.74, 165.40, 135.59, 135.08, 133.43, 129.53, 128.27, 120.66, 53.61, 52.93, 45.73, 45.32, 20.05.

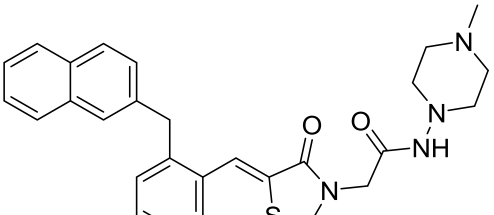

N-(4-methylpiperazin-1-yl)-2-(5-(2-(naphthalen-2-ylmethyl)benzylidene)-4-oxo-2-thioxothiazolidin-3-yl)acetamide 5s: isolated as a yellow solid (38%). **mp** >257 °C (dec.). ***R$_f$*** = 0.40 (CH_2Cl_2 : MeOH, 9 : 1). **IR** ν_{max} (ATR)/cm^{-1}: 3228 (NH), 1726 (HNC=O), 1680 (NC=O), 1591 (SC=C). **HRMS** (ESI+): calculated for $C_{28}H_{29}N_4O_2S_2$ [M + H]$^+$ requires 517.1732; found 517.1740. **^{1}H NMR** (400 MHz, DMSO-*d$_6$*) δ_H 9.37 (s, 1H), 8.08 – 8.01 (m, 1H), 7.89 – 7.74 (m, 3H), 7.59 (d, *J* = 1.5 Hz, 1H), 7.54 – 7.41 (m, 6H), 7.32 (dd, *J* = 8.5, 1.5 Hz, 1H), 4.94 (s, 1H), 4.56 (s, 1H), 4.35 (s, 2H), 3.33 – 2.90 (m, 8H), 2.70 (s, 3H). **^{13}C NMR** (100 MHz, DMSO-d$_6$) δ_C 194.19, 171.51, 162.68, 142.34, 138.16, 133.53, 132.45, 132.09, 131.94, 131.81, 131.67, 128.72, 128.60, 128.16, 127.97, 127.87, 127.61, 127.05, 126.71, 126.08, 124.64, 124.48, 52.03, 51.17, 50.04, 42.24.

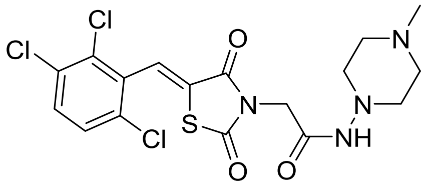

2-(2,4-Dioxo-5-(2,3,6-trichlorobenzylidene)thiazolidin-3-yl)-*N*-(4-methylpiperazin-1-yl)acetamide 10: isolated as a yellow solid (40%). **mp** >245 °C (dec.). ***R**$_f$* = 0.35 (CH_2Cl_2 : MeOH, 9 : 1). **IR** ν_{max} (ATR)/cm^{-1}: 3220 (NH), 1712 (HNC=O), 1668 (NC=O), 1591 (SC=C). **HRMS** (ESI+): calculated for $C_{19}H_{18}Cl_3N_4O_3S$ [M + H]$^+$ requires 463.0165; found 463.0173. **^{1}H NMR** (400 MHz, CD_3OD) δ_H 7.85 (s, 1H), 7.64 (d, *J* = 9.0 Hz, 1H), 7.52 (d, *J* = 9.0 Hz, 1H), 4.72 (s, 1H), 4.35 (s, 1H), 3.31 (s, 2H), 3.22 – 2.59 (m, 8H), 2.51 (s, 3H). **^{13}C NMR** (101 MHz, CD_3OD) δ_C 168.43, 166.49, 164.67, 133.38, 132.11, 131.96, 131.83, 131.58, 129.18, 128.64, 128.49, 54.00, 53.94, 53.52, 52.87, 43.42, 42.05.

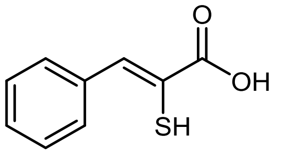

Mercapto-3-phenylacrylic acid 6a: isolated as off white crystals (79%). **mp** >300 ºC. **IR** ν_{max} (ATR)/cm^{-1}: 1574 (OC=O), 1387 (HSC=C). **HRMS** (ESI−): calculated for $C_9H_7O_2S$ [M – H]$^-$ requires 179.0172; found 179.0158. **^{1}H NMR** (400 MHz, CD_3OD) δ_H 7.81 (s, 1H), 7.72-7.58 (m, 2H), 7.49-7.41 (m, 2H), 7.39-7.31 (m, 1H). **^{13}C NMR** (100 MHz, CD_3OD) δ_C 166.77, 135.29, 134.12, 129.53, 128.46, 128.21, 123.67.

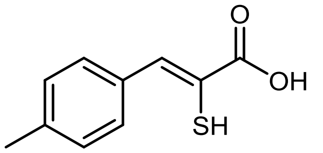

Mercapto-3-(p-tolyl)acrylic acid 6b: isolated as yellow crystals (77%). **mp** >300 ºC. **IR** ν_{max} (ATR)/cm^{-1}: 1615 (OC=O), 1378 (HSC=C). **HRMS** (ESI−): calculated for $C_{10}H_9O_2S$ [M − H]$^-$ requires 193.0329; found 193.0325. **^{1}H NMR** (400 MHz, CD_3OD) δ_H 7.66 (s, 1H), 7.45 (d, *J* = 8.0 Hz, 2H), 7.15 (d, *J* = 8.0 Hz, 2H), 2.26 (s, 3H). **^{13}C NMR** (100 MHz, CD_3OD) δ_C 166.89, 138.94, 134.20, 132.47, 129.64, 128.85, 122.35, 20.01.

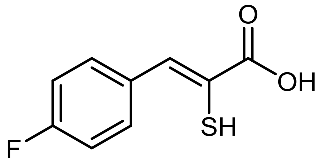

(4-Fluorophenyl)-2-mercaptoacrylic acid 6c: isolated as yellow crystals (82%). **mp** >300 ºC. **IR** ν_{max} (ATR)/cm^{-1}: 1601 (OC=O), 1364 (HSC=C). **HRMS** (ESI−): calculated for $C_9H_6FO_2S$ [M − H]$^-$ requires 197.0078; found 197.0075. **^{1}H NMR** (400 MHz, CD_3OD) δ_H 7.68 (s, 1H), 7.64-7.56 (m, 2H), 7.13-7.04 (m, 2H). **^{13}C NMR** (100 MHz, CD_3OD) δ_C 168.03, 163.92 (d, *J* = 249.0 Hz), 134.24, 133.08 (d, *J* = 8.5 Hz), 124.88, 116.51 (d, *J* = 22.0 Hz).

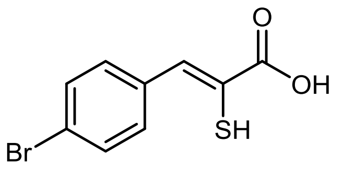

(4-Bromophenyl)-2-mercaptoacrylic acid 6d: isolated as yellow crystals (76%). **mp** >300 ºC. **IR** ν_{max} (ATR)/cm^{-1}: 1589 (OC=O), 1383 (HSC=C). **HRMS** (ESI−): calculated for $C_9H_6BrO_2S$ [M − H]$^-$ requires 256.9277; found 256.9281. **^{1}H NMR** (400 MHz, CD_3OD) δ_H 77.75 (s, 1H), 7.63-7.55 (m, 4H). **^{13}C NMR** (100 MHz, CD_3OD) δ_C 166.46, 134.38, 132.59, 131.40, 131.14, 124.82, 122.29.

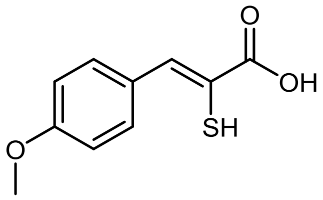

Mercapto-3-(4-methoxyphenyl)acrylic acid 6e: isolated as yellow crystals (81%). **mp** >300 ºC. **IR** ν_{max} (ATR)/cm^{-1}: 1589 (OC=O), 1389 (HSC=C). **HRMS** (ESI−): calculated for $C_9H_9O_3S$ [M − H]$^-$ requires 209.0278; found 209.0276. ^{1}**H NMR** (400 MHz, CD_3OD) δ_H 7.78 (s, 1H), 7.66 (d, J = 9.0 Hz, 2H), 7.01 (d, J = 9.0 Hz, 1H), 3.85 (s, 3H). ^{13}**C NMR** (100 MHz, CD_3OD) δ_C 167.07, 160.20, 134.14, 131.44, 127.78, 120.44, 113.63, 54.42.

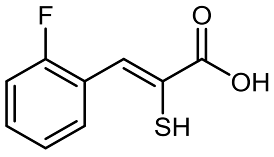

(2-Fluorophenyl)-2-mercaptoacrylic acid 6f: isolated as pale yellow crystals (76%). **mp** >300 ºC. **IR** ν_{max} (ATR)/cm^{-1}: 1619 (OC=O), 1330 (HSC=C). **HRMS** (ESI−): calculated for $C_9H_6FO_2S$ [M − H]$^-$ requires 197.0078; found 197.0081. ^{1}**H NMR** (400 MHz, CD_3OD) δ_H 7.91 (s, 1H), 7.89 (dd, J = 8.0, 1.5 Hz, 1H), 7.45-7.36 (m, 1H), 7.28 (td, J = 7.5, 1.0 Hz, 1H), 7.18 (ddd, J = 10.5, 8.0, 1.0 Hz, 1H). ^{13}**C NMR** (100 MHz, CD_3OD) δ_C 166.28, 160.40 (d, J = 250.5 Hz), 130.50 (d, J = 8.5 Hz), 129.18 (d, J = 2.5 Hz), 126.45, 125.37 (d, J = 6.5 Hz), 123.86 (d, J = 4.0 Hz), 123.17 (d, J = 12.5 Hz), 115.09 (d, J = 22.0 Hz).

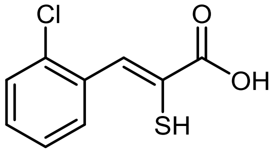

(2-Chlorophenyl)-2-mercaptoacrylic acid 6g: isolated as pale yellow crystals (81%). **mp** >300 ºC. **IR** ν_{max} (ATR)/cm^{-1}: 1588 (OC=O), 1360 (HSC=C). **HRMS** (ESI−): calculated for $C_9H_6ClO_2S$ [M − H]$^-$ requires 212.9783; found 212.9781. ^{1}**H NMR** (400 MHz, CD_3OD) δ_H 7.84 (s, 1H), 7.72-7.61 (m, 1H), 7.38 (dd, J = 8.0, 1.5 Hz, 1H), 7.32-7.19 (m, 2H). ^{13}**C NMR** (100 MHz, CD_3OD) δ_C 166.17, 133.80, 133.56, 130.42, 129.75, 129.47, 129.43, 127.04, 126.56.

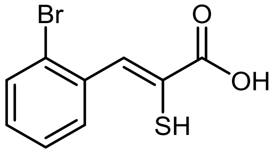

(2-Bromophenyl)-2-mercaptoacrylic acid 6h: isolated as pale yellow crystals (75%). **mp** >300 ºC. **IR** ν_{max} (ATR)/cm^{-1}: 1575 (OC=O), 1358 (HSC=C). **HRMS** (ESI−): calculated for $C_9H_6BrO_2S$ [M − H]$^-$ requires 256.9277; found 256.9280. ^{1}**H NMR** (400 MHz, CD_3OD) δ_H 7.77 (s, 1H), 7.65 (dd, J = 7.8, 1.5 Hz, 1H), 7.58 (dd, J = 8.0, 1.0 Hz, 1H), 7.33 (td, J = 7.5, 1.0 Hz, 1H), 7.15 (td, J = 7.5, 1.5 Hz, 1H). ^{13}**C NMR** (100 MHz, CD_3OD) δ_C 166.12, 135.44, 133.04, 132.73, 129.91, 129.59, 127.15, 126.97, 123.94.

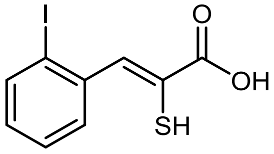

(2-Iodophenyl)-2-mercaptoacrylic acid 6i: isolated as light green crystals (78%). **mp** >300 ºC. **IR** ν_{max} (ATR)/cm^{-1}: 1577 (OC=O), 1385 (HSC=C). **HRMS** (ESI−): calculated for $C_9H_6IO_2S$ [M − H]$^-$ requires 304.9139; found 304.9143. ^{1}**H NMR** (400 MHz, CD_3OD) δ_H 9.60 7.85 (dd, J = 8.0, 1.0 Hz, 1H), 7.63 (d, J = 1.0 Hz, 1H), 7.56 (dd, J = 8.0, 1.5 Hz, 1H), 7.35 (td, J = 7.5, 1.0 Hz, 1H), 6.97 (td, J = 7.5, 1.5 Hz, 1H). ^{13}**C NMR** (100 MHz, CD_3OD) δ_C 166.06, 139.33, 139.08, 137.74, 129.82, 128.97, 127.90, 126.77, 99.35.

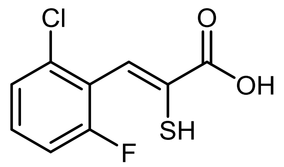

(2-Chloro-6-fluorophenyl)-2-mercaptoacrylic acid 6j: isolated as a white solid (81%). **mp** >300 ºC. **IR** ν_{max} (ATR)/cm^{-1}: 1578 (OC=O), 1370 (HSC=C). **HRMS** (ESI−): calculated for $C_9H_6FClO_2S$ [M − H]$^-$ requires 230.9688; found 230.9690. **^{1}H NMR** (400 MHz, CD_3OD) δ_H 7.53 (s, 1H), 7.40 (td, *J* = 8.0, 6.0 Hz, 1H), 7.33 (d, *J* = 8.0 Hz, 1H), 7.16 (t, *J* = 8.0 Hz, 1H). **^{13}C NMR** (100 MHz, CD_3OD) δ_C 165.26, 159.38 (d, *J* = 250.5 Hz), 133.86 (d, *J* = 5.0 Hz), 131.88, 130.52 (d, *J* = 9.5 Hz), 126.02, 125.11 (d, *J* = 3.5 Hz), 122.82 (d, *J* = 19.0 Hz), 114.21 (d, *J* = 22.5 Hz).

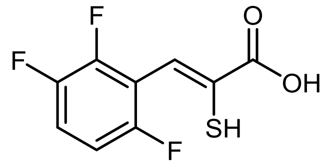

Mercapto-3-(2,3,6-trifluorophenyl)acrylic acid 6k: isolated as yellow crystals (79%). **mp** >300 ºC. **IR** ν_{max} (ATR)/cm^{-1}: 1622 (OC=O), 1371 (HSC=C). **HRMS** (ESI−): calculated for $C_9H_4F_3O_2S$ [M − H]$^-$ requires 232.9890; found 232.9892. **^{1}H NMR** (400 MHz, CD_3OD) δ_H 7.69 (s, 1H), 7.34 (ddt, *J* = 9.5, 5.0, 2.5 Hz, 1H), 7.09 (dddd, *J* = 10.5, 8.0, 6.5, 3.0 Hz, 1H). **^{13}C NMR** (100 MHz, CD_3OD) δ_C 166.91, 160.15 (dd, *J* = 11.1, 3.4 Hz), 148.08 – 147.28 (m), 145.95 – 144.50 (m), 131.33, 127.83 – 126.93 (m), 124.11, 111.66 (dd, *J* = 25.7, 3.6 Hz), 106.68 (dd, *J* = 28.4, 21.6 Hz).

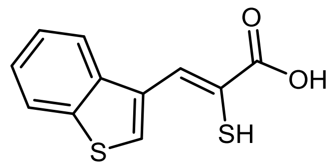

(Benzo[b]thiophen-3-yl)-2-mercaptoacrylic acid 6l: isolated as a yellow solid (75%). **mp** >300 ºC. **IR** ν_{max} (ATR)/cm^{-1}: 1672 (OC=O), 1381 (HSC=C). **HRMS** (ESI−): calculated for $C_{11}H_7O_2S$ [M − H]$^-$ requires 234.9893; found 234.9893. **^{1}H NMR** (400 MHz, CD_3OD) δ_H 9.60 (s, 1H), 7.95 (s, 1H), 7.92 (dd, J =7.5 Hz, 2H), 7.27-7.52 (m, 2H). **^{13}C NMR** (100 MHz, CD_3OD) δ_C 169.03, 14.38, 139.63, 139.02, 134.70, 124.73, 124.46, 123.46, 123.30, 121.23, 115.15.

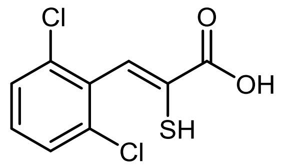

(2,6-Dichlorophenyl)-2-mercaptoacrylic acid 6m: isolated as yellow crystals (80%). **mp** >300 ºC. **IR** ν_{max} (ATR)/cm^{-1}: 1593 (OC=O), 1368 (HSC=C). **HRMS** (ESI−): calculated for $C_9H_6Cl_2O_2S$ [M − H]$^-$ requires 246.9393; found 246.9392. **^{1}H NMR** (400 MHz, CD_3OD) δ_H 7.44 (s, 1H), 7.39-7.32 (m, 2H), 7.28-7.20 (m, 1H). **^{13}C NMR** (100 MHz, CD_3OD) δ_C 165.23, 133.74, 133.36, 131.56, 130.08, 129.93, 128.00.

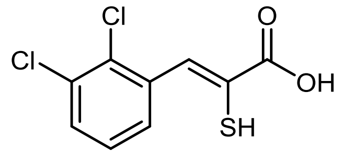

(2,3-Dichlorophenyl)-2-mercaptoacrylic acid 6n: isolated as yellow crystals (77%). **mp** >300 ºC. **IR** ν_{max} (ATR)/cm^{-1}: 1594 (OC=O), 1364 (HSC=C). **HRMS** (ESI−): calculated for $C_9H_5Cl_2O_2S$ [M − H]$^-$ requires 246.9393; found

246.9394. ^{1}**H NMR** (400 MHz, CD_3OD) δ_H 7.74 (s, 1H), 7.70 (d, J = 2.5 Hz, 1H), 7.39 (d, J = 8.5 Hz, 1H), 7.27 (dd, J = 8.5, 2.5 Hz, 1H). ^{13}**C NMR** (100 MHz, CD_3OD) δ_C 165.83, 135.22, 132.40, 132.18, 130.80, 129.45, 128.99, 128.88, 128.77.

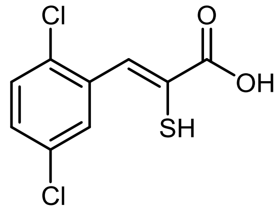

(2,5-Dichlorophenyl)-2-mercaptoacrylic acid 6o: isolated as yellow crystals (75%). **mp** >300 ºC. **IR** v_{max} (ATR)/cm^{-1}: 1582 (OC=O), 1367 (HSC=C). **HRMS** (ESI−): calculated for $C_9H_6Cl_2O_2S$ $[M-H]^-$ requires 246.9393; found 246.9393. ^{1}**H NMR** (400 MHz, CD_3OD) δ_H 7.79 (s, 1H), 7.62 (dd, J = 8.0, 1.5 Hz, 1H), 7.45 (dd, J = 8.0, 1.5 Hz, 1H), 7.29 (t, J = 8.0 Hz, 1H). ^{13}**C NMR** (100 MHz, CD_3OD) δ_C 165.96, 136.08, 133.12, 131.51, 128.66, 127.86, 127.33.

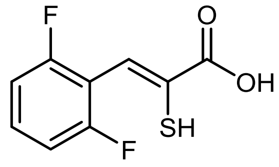

(2,6-Difluorophenyl)-2-mercaptoacrylic acid 6p: isolated as yellow crystals (82%). **mp** >300 ºC. **IR** v_{max} (ATR)/cm^{-1}: 1580 (OC=O), 1355 (HSC=C). **HRMS** (ESI−): calculated for $C_9H_6F_2O_2S$ $[M-H]^-$ requires 214.9984; found 214.9984. ^{1}**H NMR** (400 MHz, CD_3OD) δ_H 7.43 (s, 1H), 7.34 (tt, J = 8.5, 6.5 Hz, 1H), 6.94 (t, J = 8.0 Hz, 2H). ^{13}**C NMR** (100 MHz, CD_3OD) δ_C 165.35, 159.67 (d, J = 250.5 Hz), 132.04, 130.75 (t, J = 10.5 Hz), 122.21, 112.65 (m), 111.26 (m).

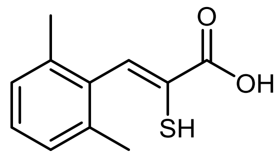

(2,6-Dimethylphenyl)-2-mercaptoacrylic acid 6q: isolated as pale yellow crystals (79%). **mp** >300 ºC. **IR** v_{max} (ATR)/cm^{-1}: 1599 (OC=O), 1370 (HSC=C). **HRMS** (ESI−): calculated for $C_{11}H_{11}O_2S$ $[M-H]^-$ requires 207.0485; found 207.0484. ^{1}**H NMR** (400 MHz, CD_3OD) δ_H 7.60 (s, 1H), 7.04 (dd, J = 8.5, 6.5 Hz, 1H), 6.97 (d, J = 7.0 Hz, 2H), 2.10 (s, 6H). ^{13}**C NMR** (100 MHz, CD_3OD) δ_C 165.84, 135.08, 134.95, 134.55, 128.49, 127.79, 127.29, 18.52.

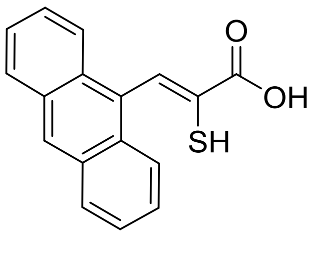

3-(anthracen-9-yl)-2-mercaptoacrylic acid 6r: isolated as yellow solid (33%). **mp** 252-254 ºC. **IR** v_{max} (ATR)/cm^{-1}: 1676 (OC=O), 1223 (HSC=C). **HRMS** (ESI−): calculated for $C_{17}H_{12}O_2NaS$ $[M+Na]^+$: 303.0450, found: 303.0452. ^{1}**H NMR** (400 MHz, CD_3OD) δ_H 8.63 (s, 1 H), 8.29 (s, 1 H), 8.12 - 8.18 (m, 2 H), 7.95 - 8.02 (m, 2 H), 7.52 - 7.59 (m, 4 H). ^{13}**C NMR** (100 MHz, CD_3OD) δ_C 180.7, 137.7, 132.9, 131.5, 129.2, 128.9, 128.3, 126.4, 126.0, 124.4.

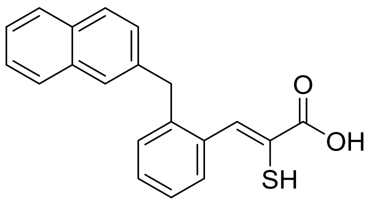

2-mercapto-3-(2-(naphthalen-2-ylmethyl)phenyl)acrylic acid 6s: isolated as white solid ().**mp** 250-256 ºC. **IR** v_{max} (ATR)/cm^{-1}: 1690 (OC=O), 1245 (HSC=C). **HRMS** (ESI−): calculated for $C_{20}H_{15}O_2S$ $[M-H]^-$: 320.0871, found: 320.0866. ^{1}**H NMR** (400 MHz, CD_3OD) δ_H 7.90 (s, 1H), 7.83 – 7.66

(m, 3H), 7.61 – 7.51 (m, 2H), 7.46 – 7.21 (m, 6H), 4.15 (s, 2H). **^{13}C NMR** (100 MHz, CD_3OD) δ_C 167.75, 140.98, 139.03, 136.34, 135.02, 134.91, 133.57, 131.66, 129.96, 129.64, 128.95, 128.54, 128.28, 128.07, 127.64, 127.53, 126.95, 126.36, 101.38, 40.63.

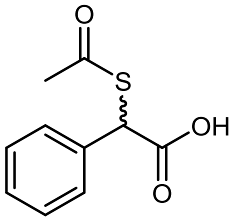

2-(Acetylthio)-2-phenylacetic acid 12a: isolated as an off white solid (80%). **mp** 136-139 °C. ***R$_f$*** = 0.45 (C_6H_{12}/EtOAc 6:4). **IR** ν_{max} (ATR)/cm^{-1}: 2946 (COO-H), 1711 (HOC=O), 1650 (SC=O). **HRMS** (ESI+): calculated for $C_{10}H_{10}O_3SNa$ $[M + Na]^+$ requires 233.0243; found 233.0244. **^{1}H NMR**(400 MHz, DMSO-d$_6$) δ_H 13.16 (br., s, 1H), 7.57-7.11 (m, 5H), 5.20 (s, 1H), 2.36 (s, 3H). **^{13}C NMR** (100 MHz, DMSO-d$_6$) δ_C 194.40, 170.98, 135.90, 129.29, 128.69, 51.41, 30.36.

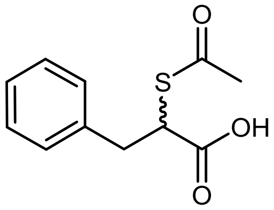

2-(Acetylthio)-3-phenylpropanoic acid 12b: isolated as pale yellow oil (85%). ***R$_f$*** = 0.50 (C_6H_{12}/EtOAc 6:4). **IR** ν_{max} (ATR)/cm^{-1}: 295 (COO-H), 1710 (HOC=O), 1648 (SC=O). **HRMS** (ESI+): calculated for $C_{11}H_{12}O_3SNa$ $[M + Na]^+$ requires 247.0399; found 247.0400. **^{1}H NMR**(400 MHz, DMSO-d$_6$) δ_H 12.96 (s, 1H), 7.33-7.27 (m, 2H), 7.23 (td, *J* = 6.0, 1.5 Hz, 3H), 4.25 (t, *J* = 8.0, 1H), 3.18 (dd, *J* = 14.0, 8.0 Hz, 1H), 2.94 (dd, *J* = 14.0, 7.5 Hz, 1H), 2.33 (s, 3H). **^{13}C NMR** (100 MHz, DMSO-d$_6$) δ_C 193.97, 172.07, 138.05, 129.45, 128.78, 127.23, 47.46, 37.68, 30.66.

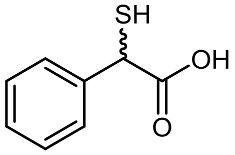

2-Mercapto-2-phenylacetic acid 13a: isolated as pale yellow oil (89%). **IR** ν_{max} (ATR)/cm^{-1}: 2946 (COO-H), 1711 (HOC=O). **HRMS** (ESI+): calculated for $C_8H_7O_2S$ $[M - H]^-$ requires 167.0172; found 167.0163. **^{1}H NMR**(400 MHz, DMSO-d$_6$) δ_H 13.00 (br., s, 1H), 7.47-7.25 (m, 5H), 4.80 (d, *J* = 7.5 Hz, 1H), 3.52 (d, *J* = 7.5 Hz, 1H). **^{13}C NMR** (100 MHz, DMSO-d$_6$) δ_C 172.92, 140.00, 128.89, 128.31, 128.06, 45.44.

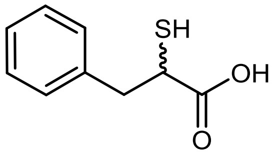

2-Mercapto-3-phenylpropanoic acid 13b: isolated as pale yellow oil (92%). **IR** ν_{max} (ATR)/cm^{-1}: 2952 (COO-H), 1719 (HOC=O). **HRMS** (ESI+): calculated for $C_9H_9O_2S$ $[M - Na]^-$ requires 181.0329; found 181.0325. **^{1}H NMR**(400 MHz, DMSO-d$_6$) δ_H 12.68 (s, 1H), 7.58-7.01 (m, 5H), 3.62 (td, *J* = 9.0, 6.5 Hz, 1H), 3.13 (dd, *J* = 14.0, 8.5 Hz, 1H), 3.12 (d, *J* = 9.0 Hz, 1H), 2.91 (dd, *J* = 14.0, 6.5 Hz, 1H). **^{13}C NMR** (100 MHz, DMSO-d$_6$) δ_C 174.16, 138.81, 129.49, 128.69, 127.00, 41.92, 41.45.

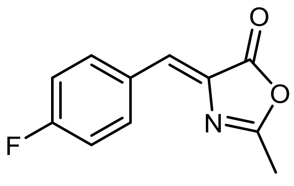

4-(4-Fluorobenzylidene)-2-methyloxazol-5(*4H*)-one 16: isolated as a yellow solid (65%). **mp** 147-150 °C (from *i*PrOH). ***R$_f$*** = 0.35 (7 : 3, hexane : EtOAc). **IR**, ν_{max} (ATR)/cm^{-1}: 2360 (s), 2341 (s), 1802 (s), 1773 (s), 1661 (s), 1630 (s), 1595

(s), 1586 (s), 1445 (s), 1427 (s), 1392 (s), 1232 (s), 1161 (s). **HRMS** (ESI+): calculated for $C_{11}H_9O_2NF$ $[M + H]^+$ requires 206.06118; found: 206.06137. **^{1}H NMR** (400 MHz, $CDCl_3$): δ_H 8.12-8.09 (2H, m, C*H* ar.), 7.15-7.11 (2H, m, C*H* ar.), 7.13 (1H, s, =C*H*), 2.41 (3H, s, C*H*$_3$). **^{13}C NMR** (100 MHz, $CDCl_3$): δ_C 167.85, 166.35, 165.62, 163.09, 134.52 (d, *J* = 9.0 Hz), 132.30, 130.17, 129.66 (d, *J* = 3.0 Hz), 116.30 (d, *J* = 22.0 Hz), 15.83. **^{19}F NMR** (376 MHz, $CDCl_3$): δ_F −106.87 (*F*ar.).

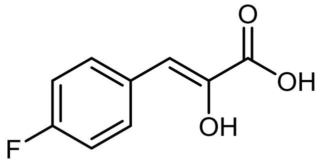

3-(4-Fluorophenyl)-2-hydroxyacrylic acid 17: isolated as a white solid (90%). **mp** 174-178 °C. **IR**, ν_{max} (ATR)/cm^{-1}: 2360 (s), 2341 (s), 1733 (w), 1717 (w), 1646 (w), 1603 (w), 1238 (w), 1144 (w). **HRMS** (ESI−): calculated for $C_9H_6O_3F$ $[M - H]^-$ requires 181.03065; found: 181.03047. **^{1}H NMR** (400 MHz, CD_3OD): δ_H 7.84-7.81 (2H, m, C*H* ar.), 7.09-7.05 (2H, m, C*H* ar.), 6.50 (1H, s, =C*H*). **^{13}C NMR** (100 MHz, CD_3OD): δ_C 168.20, 164.43, 161.99, 142.03 (d, *J* = 3.0 Hz), 132.84 (d, *J* = 3.0 Hz), 132.57 (d, *J* = 8.0 Hz), 115.97 (d, *J* = 21.0 Hz), 110.29. **^{19}F NMR** (376 MHz, CD_3OD): δ_F −116.13 (*F*ar.).

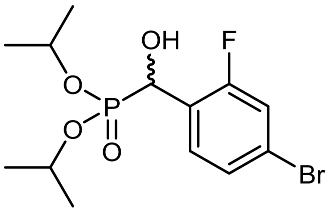

Diisopropyl ((4-bromo-2-fluorophenyl)(hydroxy)methyl)phosphonate 20: isolated as a white solid (90%). **mp** 67-70 °C. ***R$_f$*** = 0.25 (9.5 : 0.5, $CHCl_3$: MeOH). **IR**, ν_{max} (ATR)/cm^{-1}: 3226 (w), 2918 (s), 1468 (m), 1376 (w), 1191 (m, P=O), 1113 (m), 1041 (m, P-O-C), 844 and 802 (w, P-O-C), 722 (w, CH_2). **HRMS** (ESI+): calculated for $C_{13}H_{19}BrFO_4PNa$ $[M + Na]^+$ requires 391.00806; found: 391.00800. **^{1}H NMR** (400 MHz, $CDCl_3$): δ_H 7.57 (1H, tdd, *J* = 7.5, 2.5, 0.5 Hz, C*H* ar.), 7.35 (1H, ddt, *J* = 8.0, 2.0, 0.5 Hz, C*H* ar.), 7.25 (1H, ddd, *J* = 9.5, 2.0, 1.0 Hz, C*H* ar.), 5.26 (1H, dd, *J* = 11.5, 6.0 Hz, PC*H*), 4.72 (2H, dddt, *J* = 27.0, 12.0, 7.0, 6.0 Hz, C*H*), 4.03 (1H, dd, *J* = 8.0, 6.0 Hz, O*H*), 1.47-1.25 (12H, m, 4x C*H*$_3$). **^{13}C NMR** (100 MHz, $CDCl_3$): δ_C 160.49 (d, *J* = 7.0 Hz), 158.49 (d, *J* = 7.0 Hz), 130.23 (apparent t, *J* = 4.0 Hz), 127.49 (apparent t, *J* = 3.0 Hz), 124.14 (dd, *J* = 14.0, 2.0 Hz), 121.88 (dd, *J* = 9.5, 4.0 Hz), 118.57 (dd, *J* = 25.5, 2.5 Hz), 72.25 (dd, *J* = 41.0, 7.5 Hz), 63.92 (dd, *J* = 165.0, 2.5 Hz), 24.08 (dd, *J* = 19.0, 3.5 Hz), 23.71 (dd, *J* = 29.5, 5.5 Hz). **^{19}F NMR** (376 MHz, $CDCl_3$, ^{1}H-decoupled): δ_F −114.10 (d, *J* = 7.5 Hz). **^{31}P NMR** (162 MHz, $CDCl_3$, ^{1}H-decoupled): δ_P 18.31 (d, *J* = 7.0 Hz).

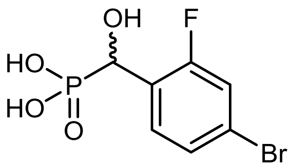

((4-Bromo-2-fluorophenyl)(hydroxy)methyl)phosphonic acid 22: isolated as a white solid (90%). **mp** >200 °C (dec.). **IR**, ν_{max} (ATR)/cm^{-1}: 3244 (br), 2980 (s), 2954 (s), 2291 (m), 1592 (s), 1483 (s), 1380 (m), 1190 (m); 1100 (m), 991 (m), 845 (w). **HRMS** (ESI+): calculated for $C_7H_6BrFO_4P$ $[M - H]^-$ requires 282.9171; found: 282.9173. **^{1}H NMR** (400 MHz, CD_3OD): δ_H 7.61 (1H, td, *J* = 8.0, 2.5 Hz, C*H* ar.), 7.45-7.09 (2H, m, C*H* ar.), 5.19 (1H, d, *J* = 14.0 Hz, C*H*). **^{13}C NMR** (125 MHz, CD_3OD): δ_C 161.06 (dd, *J* = 250.5, 6.0 Hz), 131.96, 128.41, 127.81 (d, *J* = 14.0 Hz), 122.21 (d, *J* = 9.0 Hz), 119.33 (d, *J* = 26.0 Hz), 64.88 (d, *J* =

163.5 Hz). **^{19}F NMR** (376 MHz, CD_3OD, ^{1}H-decoupled): δ_F −116.35 (*F*ar.). **^{31}P NMR** (162 MHz, $CDCl_3$, ^{1}H-decoupled): δ_P 18.62 (d, *J* = 6.0 Hz).

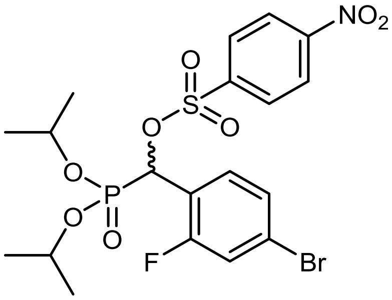

(4-Bromo-2-fluorophenyl)(diisopropoxyphosphoryl)methyl 4-nitrobenzene-sulfonate 23: isolated as a white solid (94%). **mp** 93-96 °C. ***R$_f$*** = 0.30 (99 : 1, $CHCl_3$: MeOH). **IR**, ν_{max} (ATR)/cm^{-1}: 2975 (s), 1515 (s), 1376 (s), 1210 (m, P=O), 1170 (m), 1011 (m), 870 and 855 (w), 810 (w), 787 (m), 745 (w). **HRMS** (ESI+): calculated for $C_{19}H_{22}O_8NBrFPSNa$ $[M + Na]^+$ requires 575.98634; found: 575.98578. **^{1}H NMR** (400 MHz, $CDCl_3$): δ_H 8.32-8.16 (2H, m, C*H* ar.), 8.06-7.83 (2H, m, C*H* ar.), 7.34 (1H, ddd, *J* = 9.0, 7.5, 2.0 Hz, C*H* ar.), 7.16 (2H, dt, *J* = 9.5, 1.5 Hz, C*H* ar.), 5.92 (1H, d, *J* = 16.0 Hz, PC*H*), 4.80 (1H, dp, *J* = 7.0, 6.0 Hz, CH_3C*H*), 4.57 (1H, dp, *J* = 7.0, 6.0 Hz, CH_3C*H*), 1.33 (6H, dd, *J* = 6.0, 5.0 Hz, 2x C*H*$_3$), 1.17 (6H, dd, *J* = 76.0, 6.0 Hz, 2x C*H*$_3$). **^{13}C NMR** (100 MHz, $CDCl_3$): δ_C 160.61, 158.12 (d, *J* = 7.0 Hz), 150.63, 141.99, 131.15 (apparent t, *J* = 3.0 Hz), 129.29, 127.93 (apparent t, *J* = 3.0 Hz), 124.33 (dd, *J* = 9.5, 3.0 Hz), 124.11, 118.89 (d, *J* = 25.0 Hz), 118.68, 73.43 (dd, *J* = 33.0, 7.0 Hz), 70.63 (dd, *J* = 178.0, 2.5 Hz), 24.10 (dd, *J* = 4.5, 4.0 Hz), 23.55 (dd, *J* = 35.0, 5.5 Hz). **^{19}F NMR** (376 MHz, $CDCl_3$, ^{1}H-decoupled): δ_F −113.59 (d, *J* = 5.5 Hz). **^{31}P NMR** (162 MHz, $CDCl_3$, ^{1}H-decoupled): δ_P 10.77 (d, *J* = 5.5 Hz).

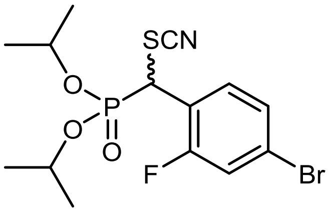

Diisopropyl ((4-bromo-2-fluorophenyl)(thiocyanato)methyl)phosphonate 24: isolated as a colourless oil (77%). **IR**, ν_{max} (ATR)/cm^{-1}: 2988 (s), 2170 (s), 1595 (s), 1460 (s), 1355 (m), 1259 (s), 1090 (m), 982 (w). **HRMS** (ESI+): calculated for $C_{14}H_{18}O_3NBrFPSNa$ $[M + Na]^+$ requires 431.98046; found: 431.98062. **^{1}H NMR** (400 MHz, $CDCl_3$): δ_H 7.63 (1H, td, *J* = 8.0, 2.0 Hz, C*H* ar.), 7.37 (1H, dd, *J* = 8.5, 2.0 Hz, C*H* ar.), 7.30 (1H, ddd, *J* = 9.0, 2.0, 1.0 Hz, C*H* ar.), 5.01-4.72 (2H, m), 4.59 (1H, dp, *J* = 7.5, 6.0 Hz, CH_3C*H*), 1.36 (6H, dd, *J* = 6.0, 1.5 Hz, 2x C*H*$_3$), 1.17 (6H, dd, *J* = 81.5, 6.0, 2x C*H*$_3$). **^{13}C NMR** (100 MHz, $CDCl_3$): δ_C 159.88 (dd, *J* = 254.0, 8.0 Hz,), 132.45, 128.38 (dd, *J* = 4.0, 2.1 Hz), 123.85 (dd, *J* = 9.5, 2.5 Hz), 119.70 (dd, *J* = 14.0, 3.0 Hz), 119.42 (d, *J* = 25.0 Hz), 110.02 (d, *J* = 10.0 Hz), 73.66 (dd, *J* = 24.0, 7.5 Hz), 39.29 (dd, *J* = 152.0, 2.5 Hz), 24.07 (apparent t, *J* = 3.5 Hz), 23.53 (dd, *J* = 58.5, 5.5 Hz). **^{19}F NMR** (376 MHz, $CDCl_3$, ^{1}H-decoupled): δ_F −113.95 (d, *J* = 4.0 Hz). **^{31}P NMR** (162 MHz, $CDCl_3$, ^{1}H-decoupled): δ_P 13.98 (d, *J* = 3.5 Hz).

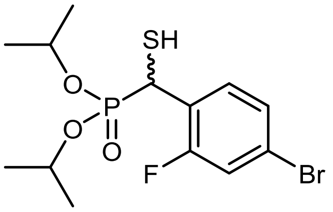

Diisopropyl ((4-bromo-2-fluorophenyl)(mercapto)methyl)phosphonate 25: isolated as a colourless oil (67%). ***R$_f$*** = 0.35 (9.5 : 0.5, $CHCl_3$: MeOH). **IR**, ν_{max} (ATR)/cm^{-1}: 2984 (m), 2940 (s), 2510 (m), 1585 (s), 1474 (s), 1380 (m), 1245 (m), 1109 (m), 980 (m), 780 (m). **HRMS** (ESI+): calculated for

$C_{13}H_{20}O_3BrFPS$ [M + H]$^+$ requires 385.00327; found: 385.00339. **^{1}H NMR** (400 MHz, $CDCl_3$): δ_H 7.56 (1H, td, *J* = 8.0, 2.0 Hz, C*H* ar.), 7.34-7.27 (1H, m, C*H* ar.), 7.21 (1H, ddd, *J* = 9.5, 2.0, 1.0 Hz, C*H* ar.), 4.81 (1H, dp, *J* = 7.5, 6.0 Hz, CH_3C*H*), 4.55 (1H, dp, *J* = 7.5, 6.0 Hz, CH_3C*H*), 4.34 (1 H, dd, *J* = 20.0, 9.0 Hz, SHC*H*), 2.64 (1H, dd, *J* = 11.0, 9.0 Hz, S*H*), 1.36 (3H, d, *J* = 6.0 Hz, CH_3), 1.29 (6H, dd, *J* = 34.0, 6.0 Hz, 2x CH_3), 1.04 (3H, d, *J* = 6.0 Hz, CH_3). **^{13}C NMR** (125 MHz, $CDCl_3$): δ_C 161.19 (dd, *J* = 36.0, 8.0 Hz), 159.55 (dd, *J* = 252.0, 8.0 Hz), 159.19 (dd, *J* = 36.0, 8.0 Hz), 132.27 (d, *J* = 50.0 Hz), 131.54 (t, *J* = 3.5 Hz), 128.00 (t, *J* = 3.0 Hz), 127.83 (d, *J* = 3.0 Hz), 124.44 (dd, *J* = 14.5, 3.0 Hz), 122.85-121.53 (m), 119.73-118.70 (m), 118.63, 72.79 (d, *J* = 7.5 Hz), 72.56 (d, *J* = 7.5 Hz), 45.47 (dd, *J* = 145.0, 119.0 Hz), 30.15 (dd, *J* = 152.5, 3.0 Hz), 24.55-24.10 (m), 23.67 (ddd, *J* = 54.0, 5.5, 3.0 Hz). **^{19}F NMR** (376 MHz, $CDCl_3$, ^{1}H-decoupled): δ_F −114.84 (d, *J* = 4.5 Hz). **^{31}P NMR** (162 MHz, $CDCl_3$, ^{1}H-decoupled): δ_P 19.72 (d, *J* = 4.5 Hz).

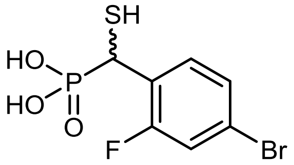

((4-Bromo-2-fluorophenyl)(mercapto)methyl)phosphonic acid 26: isolated as a white solid (92%). **mp** >190 °C (dec.). **IR**, ν_{max} (ATR)/cm^{-1}: 3234 (br), 2983 (s), 2941 (s), 2520 (m), 2292 (m), 1588 (s), 1473 (s), 1383 (m), 1192 (m); 1106 (m), 992 (m). **HRMS** (ESI+): calculated for $C_7H_8O_3BrFPS$ [M + H]$^+$ requires 300.90937; found: 300.90955. **^{1}H NMR** (400 MHz, CD_3OD): δ_H 7.47 (1H, qd, *J* = 8.0, 2.0 Hz, C*H* ar.), 7.32 (1H, d, *J* = 8.5 Hz, C*H* ar.), 7.18 (1H, dd, *J* = 9.5, 2.0 Hz, C*H* ar.), 4.42 (1H, dd, *J* = 22.5, 20.5 Hz, PC*H*). **^{13}C NMR** (125 MHz, CD_3OD): δ_C 163.63-161.91 (m), 160.59 (dd, *J* = 36.5, 7.5 Hz, 123.61-121.87 (m), 121.38 (dd, *J* = 14.0, 2.5 Hz), 120.56 (d, *J* = 25.5 Hz), 119.70 (dd, *J* = 26.0, 12.0 Hz), 111.11 (d, *J* = 9.0 Hz), 75.47 (dd, *J* = 13.5, 7.5 Hz). **^{19}F NMR** (376 MHz, CD_3OD, ^{1}H-decoupled): δ_F −115.64 (rotamer A), −115.87 (rotamer B). **^{31}P NMR** (162 MHz, CD_3OD, ^{1}H-decoupled): δ_P 15.38 (conformational isomer A), 15.25 (conformational isomer B).

3. ^{1}H and ^{13}C NMR spectra:

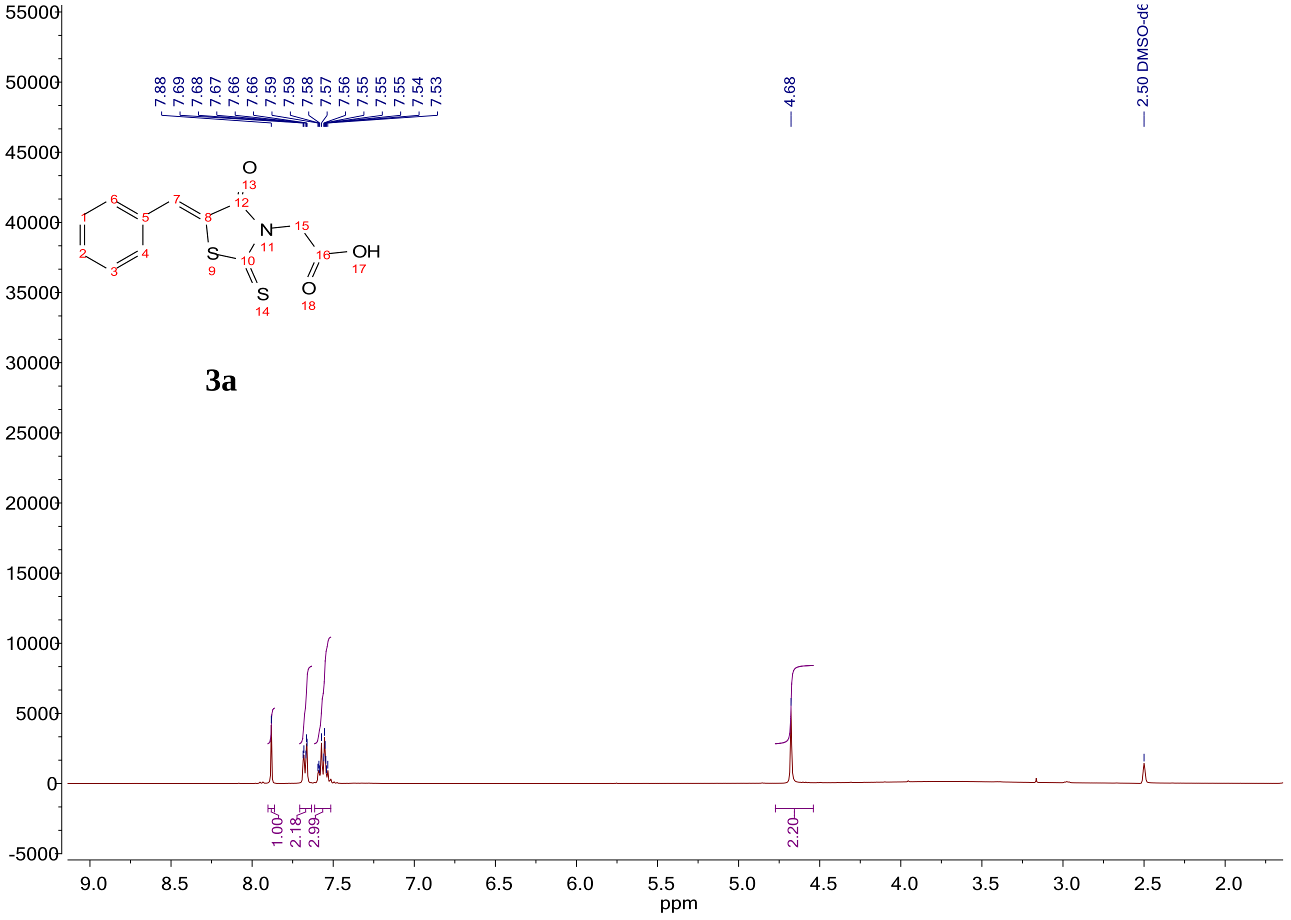

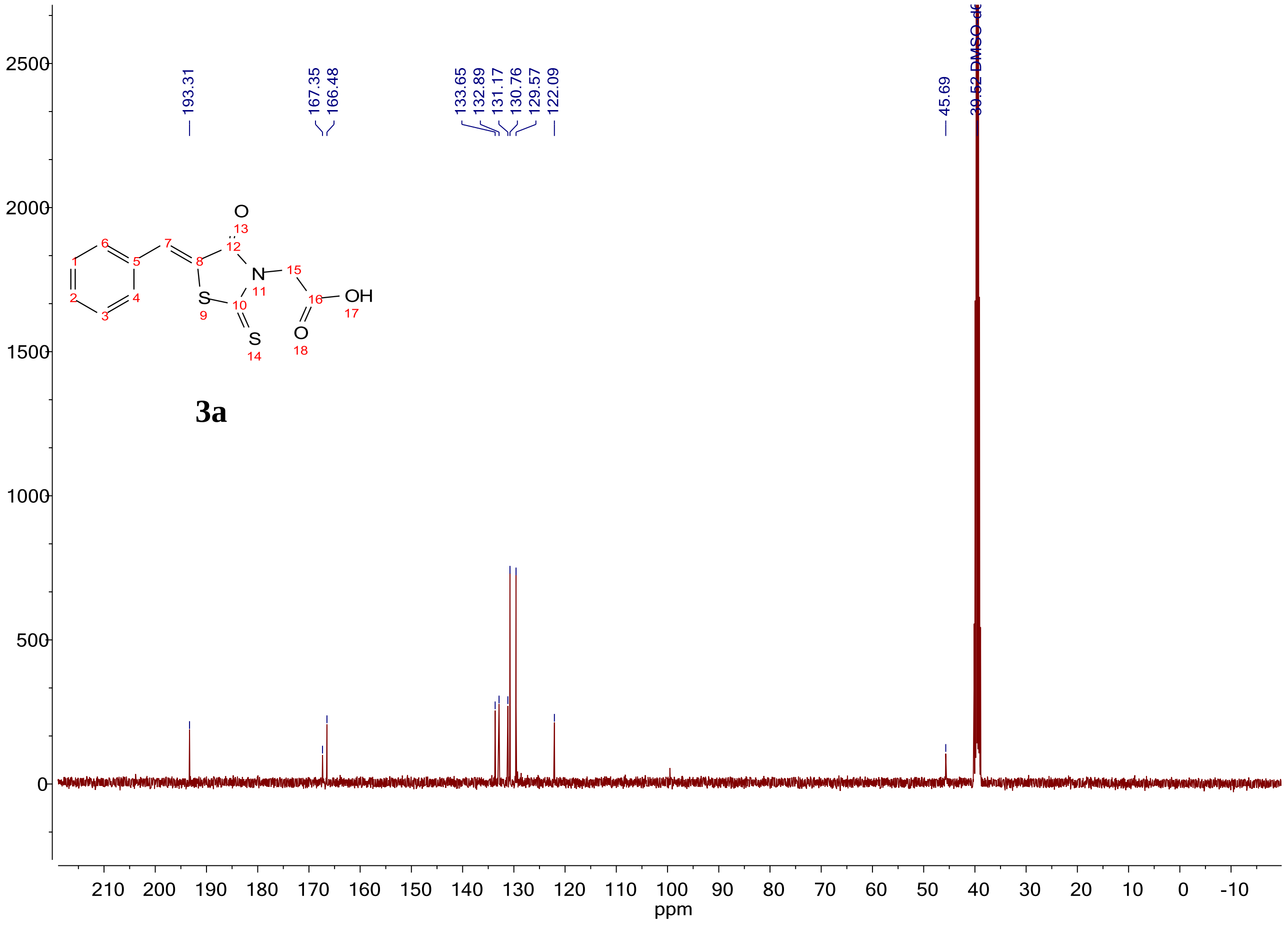

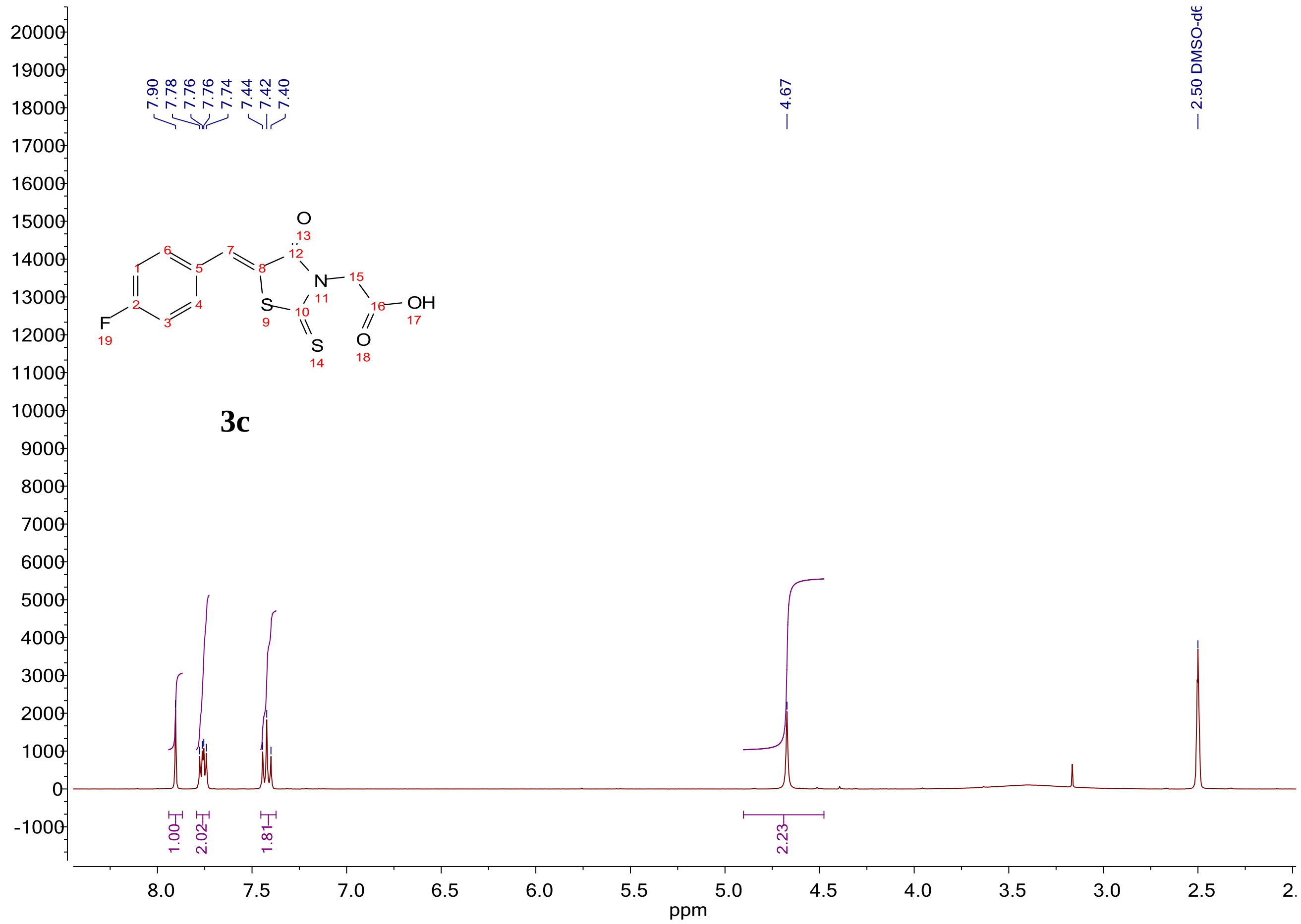
7.90
7.78
7.76
7.76
7.74
7.44
7.42
7.40
4.67
2.50 DMSO-d6
3c
1.00
2.02
1.81
2.23
ppm

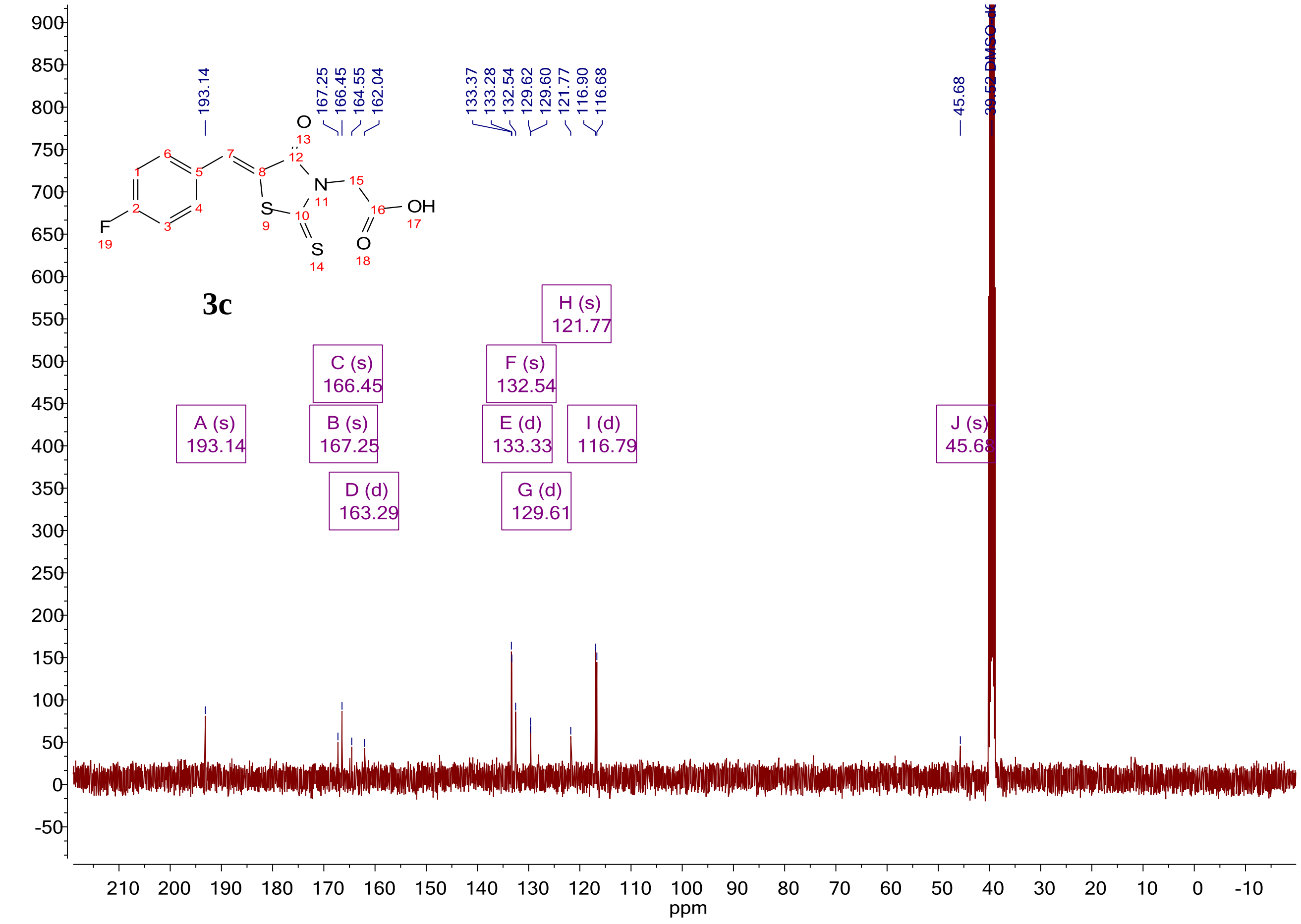
193.14
167.25
166.45
164.55
162.04
133.37
133.28
132.54
129.62
129.60
121.77
116.90
116.68
45.68
39.52 DMSO-d6
3c
H (s) 121.77
C (s) 166.45
F (s) 132.54
A (s) 193.14
B (s) 167.25
E (d) 133.33
I (d) 116.79
J (s) 45.68
D (d) 163.29
G (d) 129.61
ppm

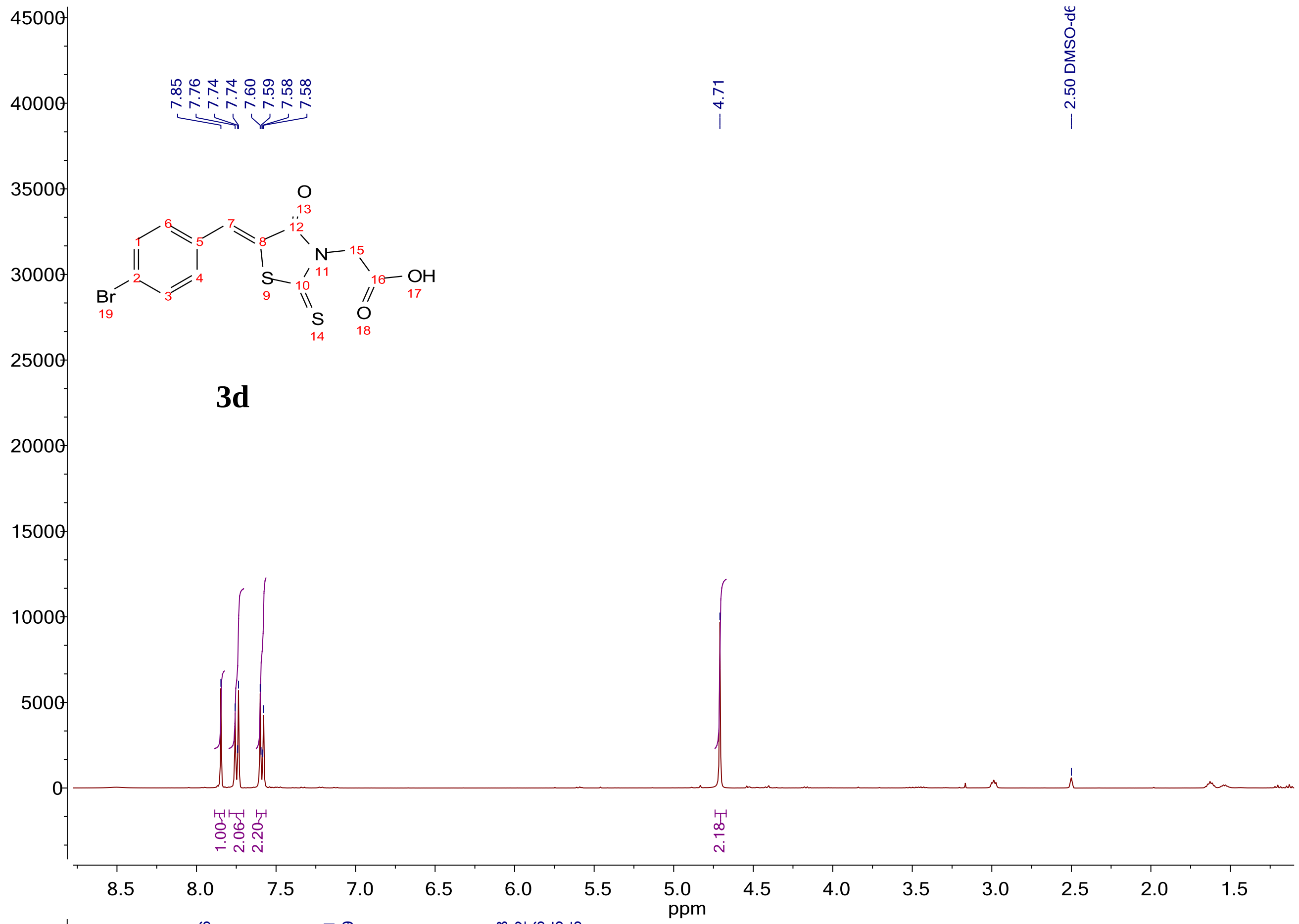

7.85
7.76
7.74
7.74
7.60
7.59
7.58
7.58
4.71
2.50 DMSO-d6
O
13
12
7
6
1
5
8
N
11
15
16
OH
17
S
9
10
2
4
3
Br
19
S
14
O
18
3d
1.00
2.06
2.20
2.18
ppm

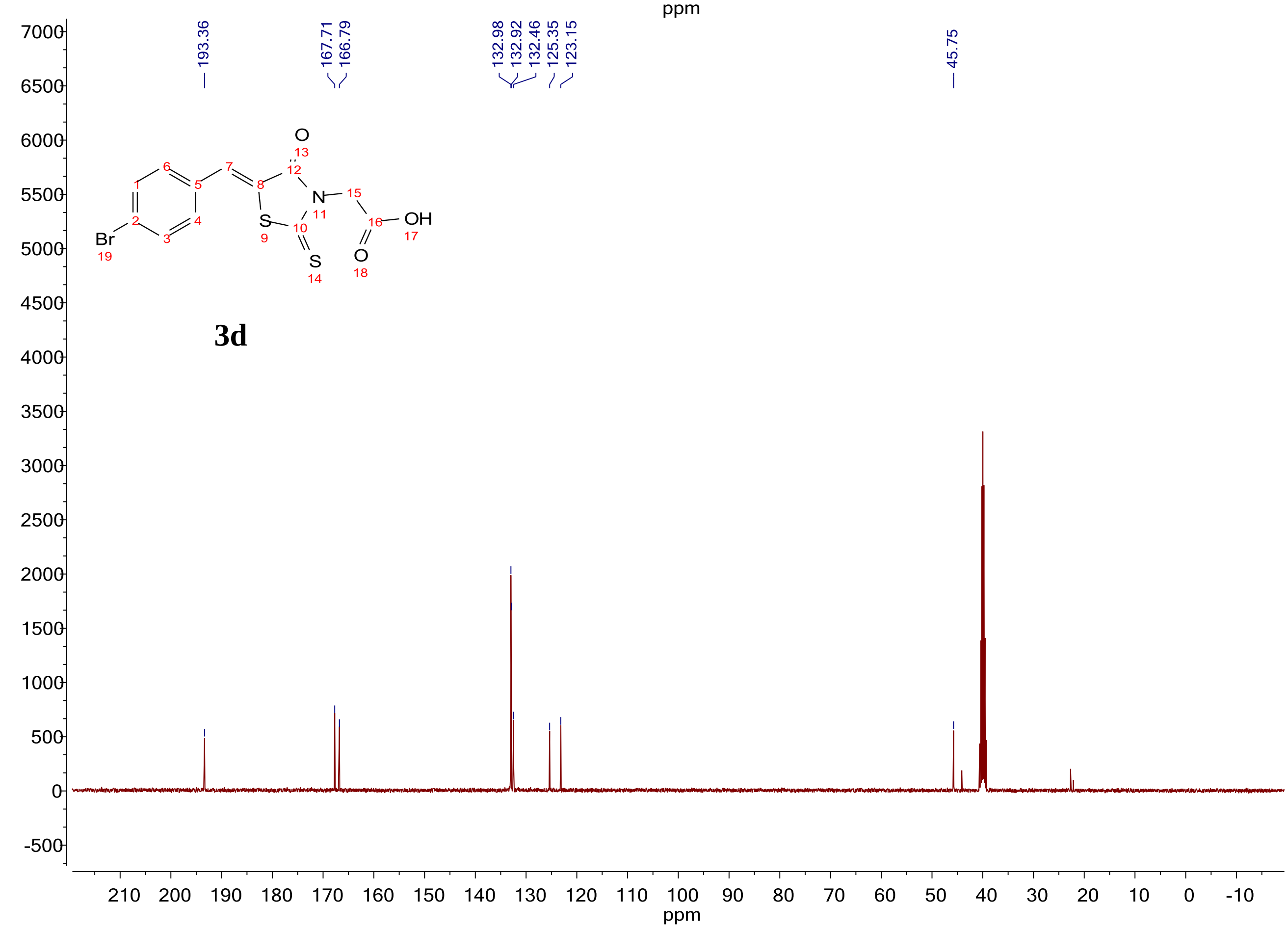

193.36
167.71
166.79
132.98
132.92
132.46
125.35
123.15
45.75
3d
ppm

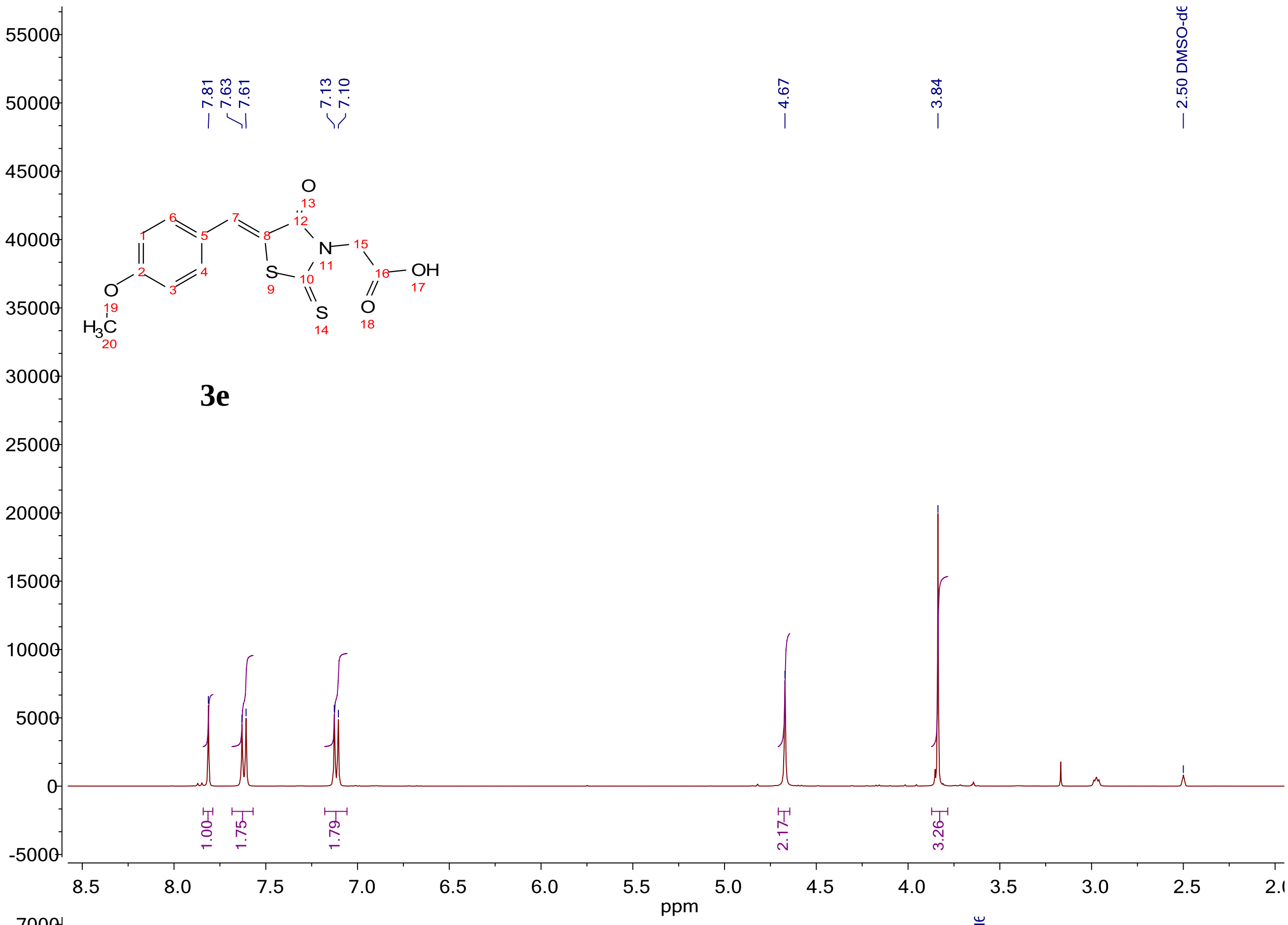
7.81
7.63
7.61
7.13
7.10
4.67
3.84
2.50 DMSO-d6
1.00
1.75
1.79
2.17
3.26
ppm
3e

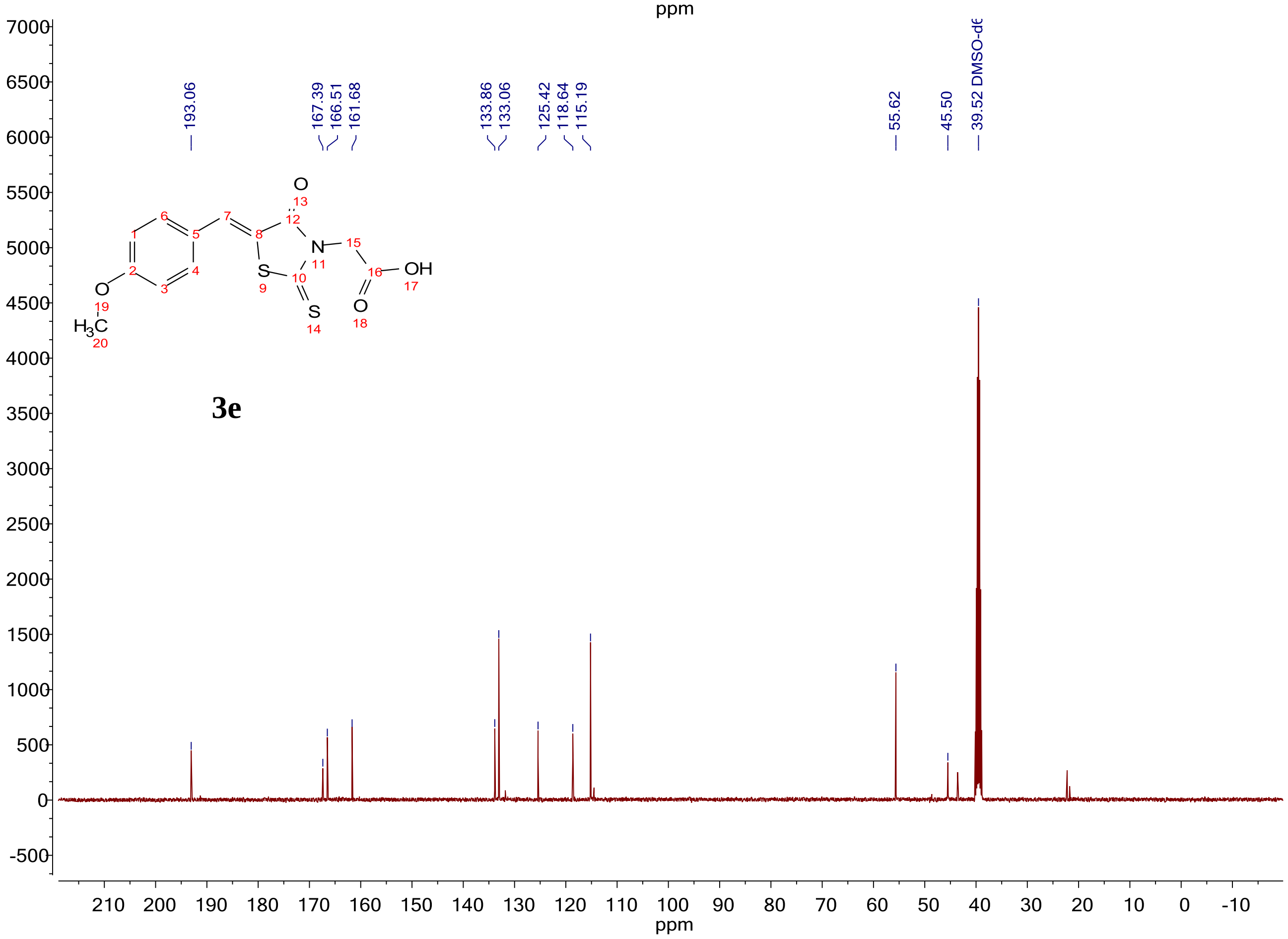
193.06
167.39
166.51
161.68
133.86
133.06
125.42
118.64
115.19
55.62
45.50
39.52 DMSO-d6
ppm
3e

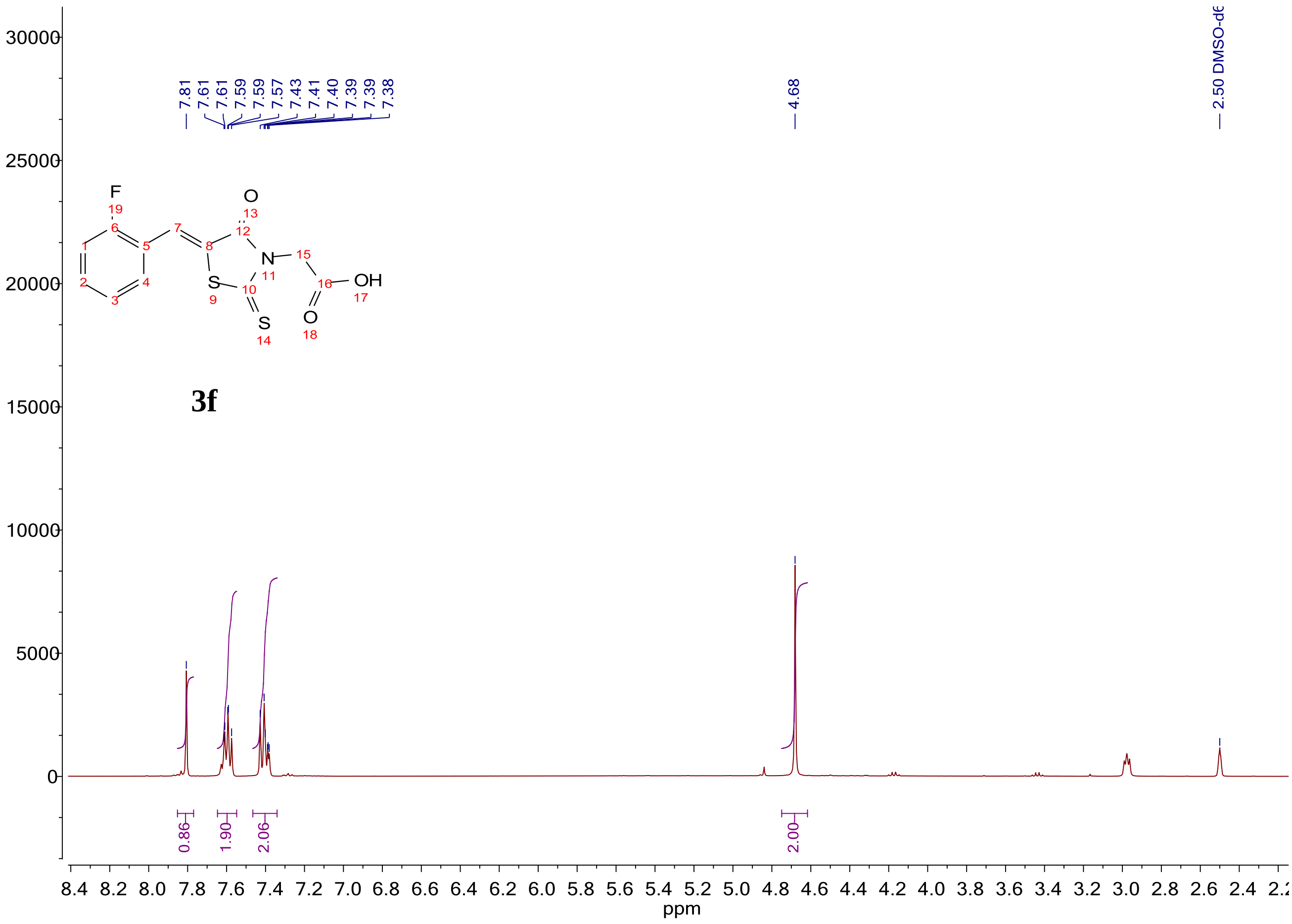

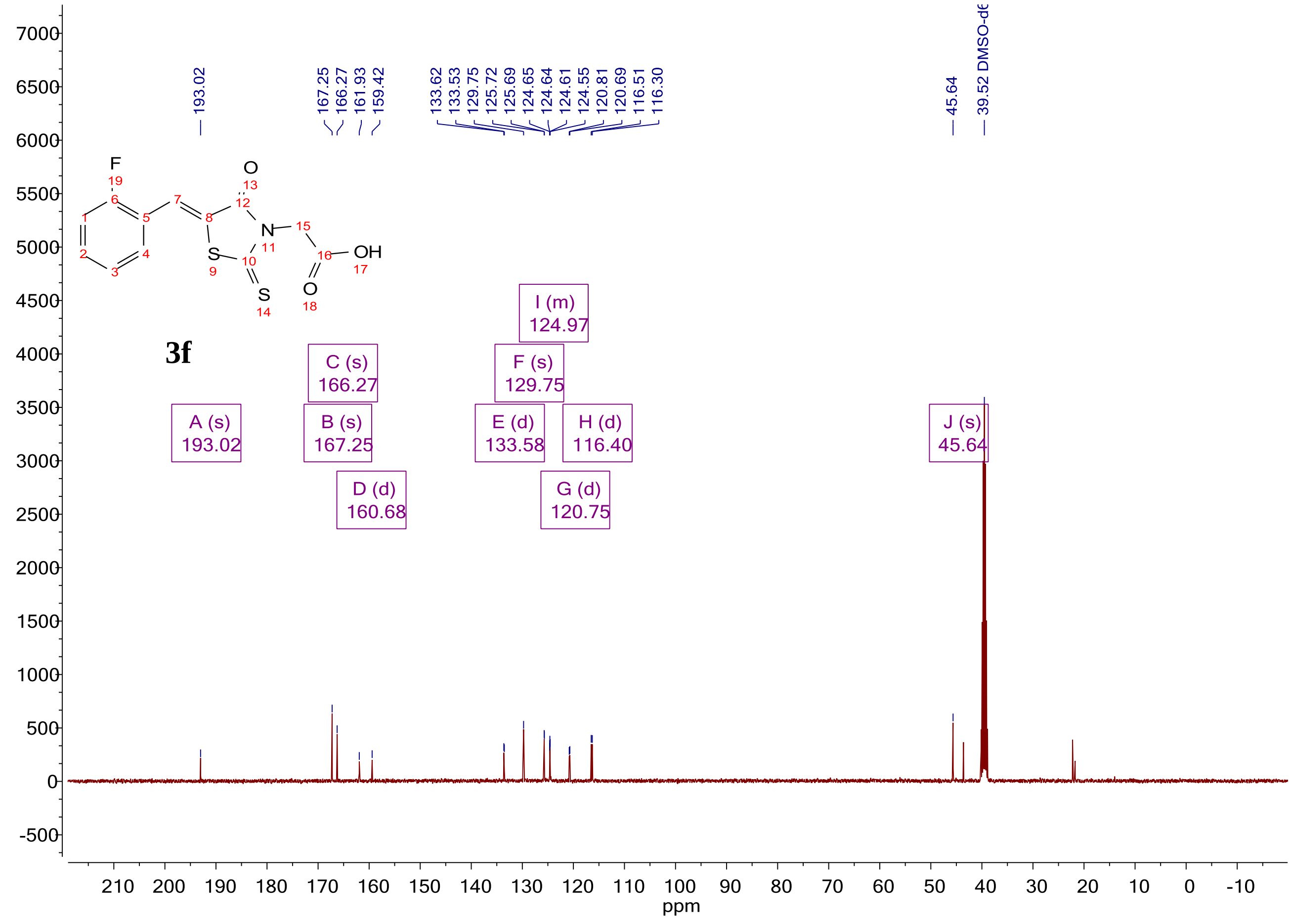

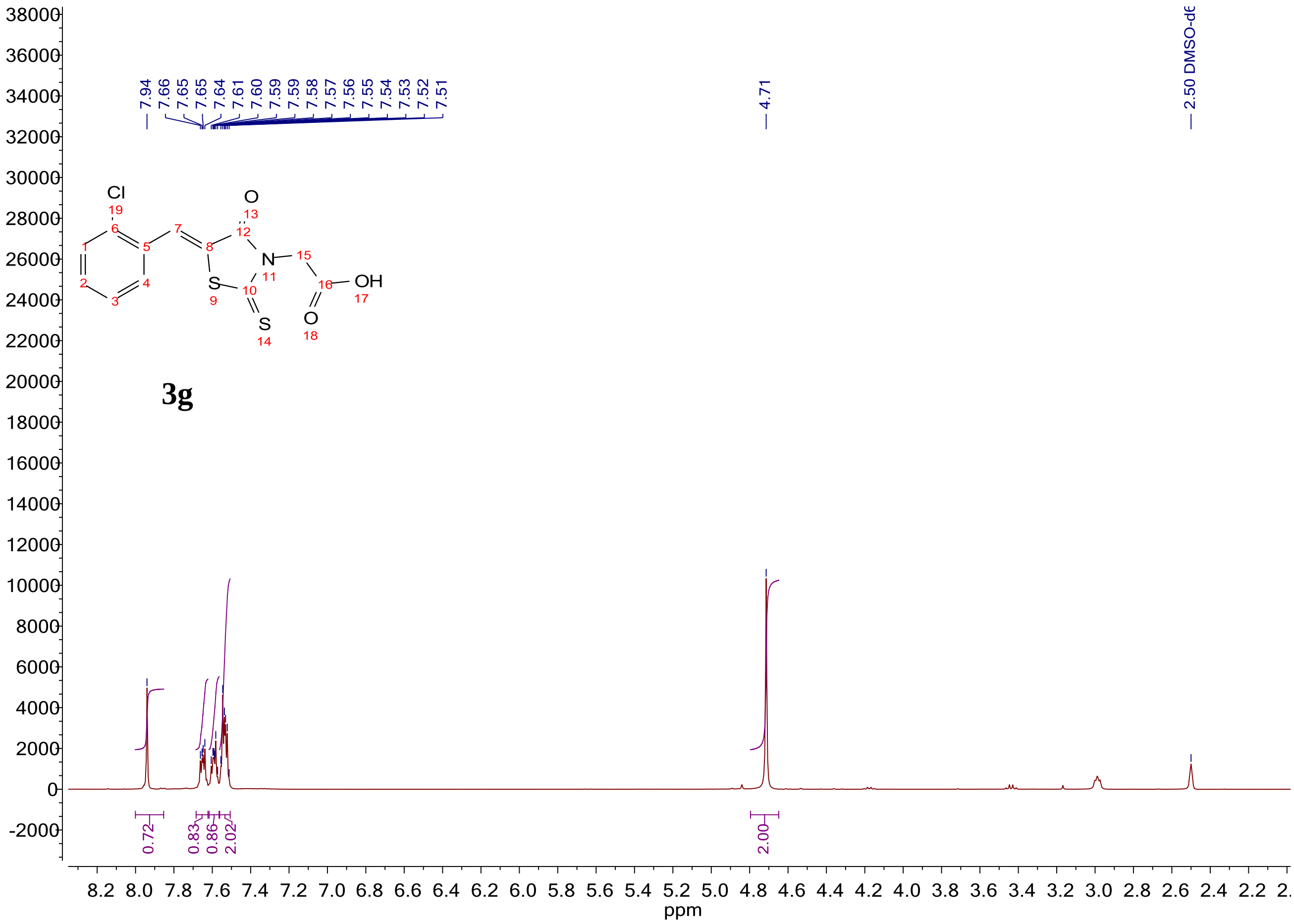
3g
7.94
7.66
7.65
7.65
7.64
7.61
7.60
7.59
7.59
7.58
7.57
7.56
7.55
7.54
7.53
7.52
7.51
4.71
2.50 DMSO-d6
0.72
0.83
0.86
2.02
2.00
ppm

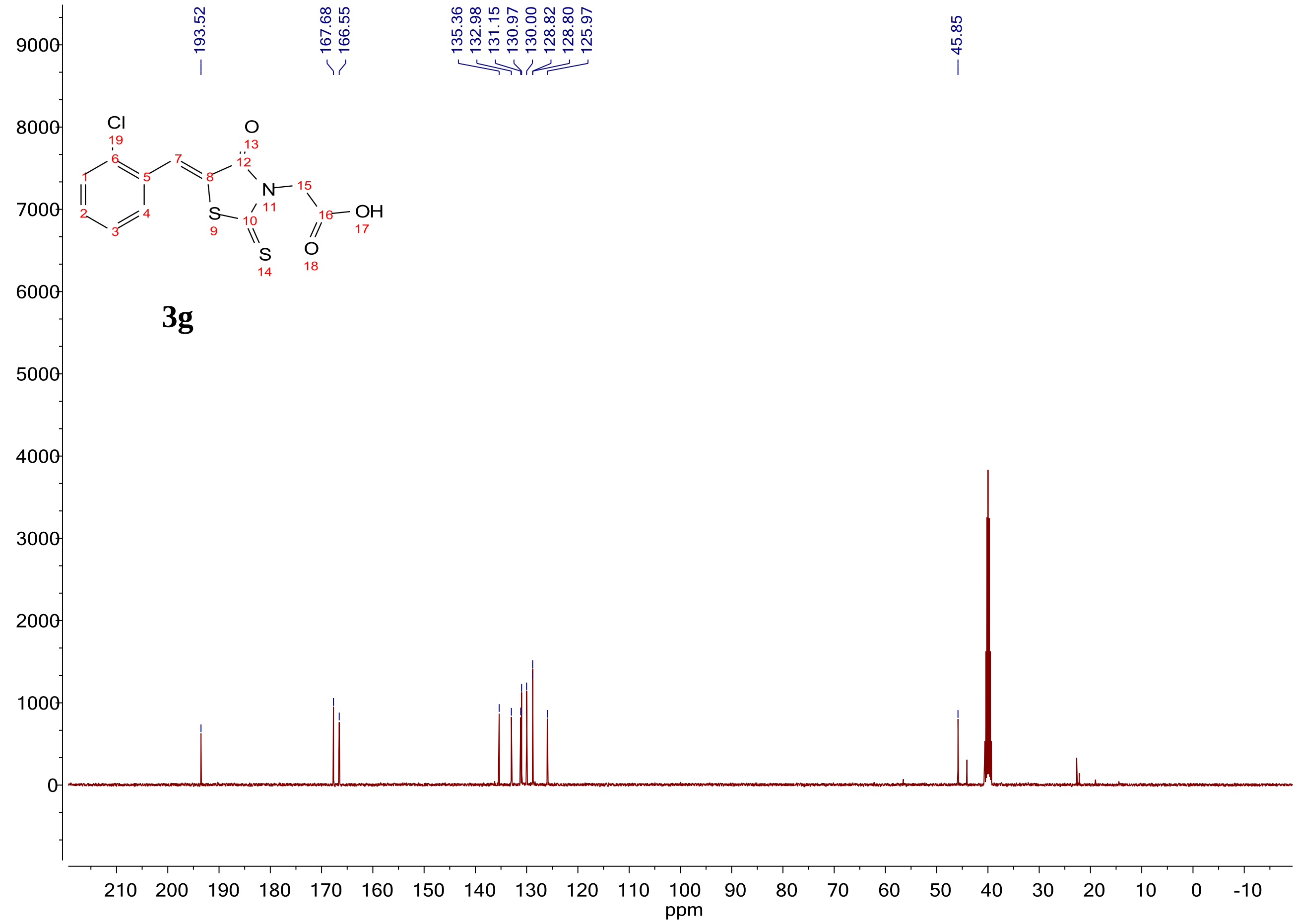
3g
193.52
167.68
166.55
135.36
132.98
131.15
130.97
130.00
128.82
128.80
125.97
45.85
ppm

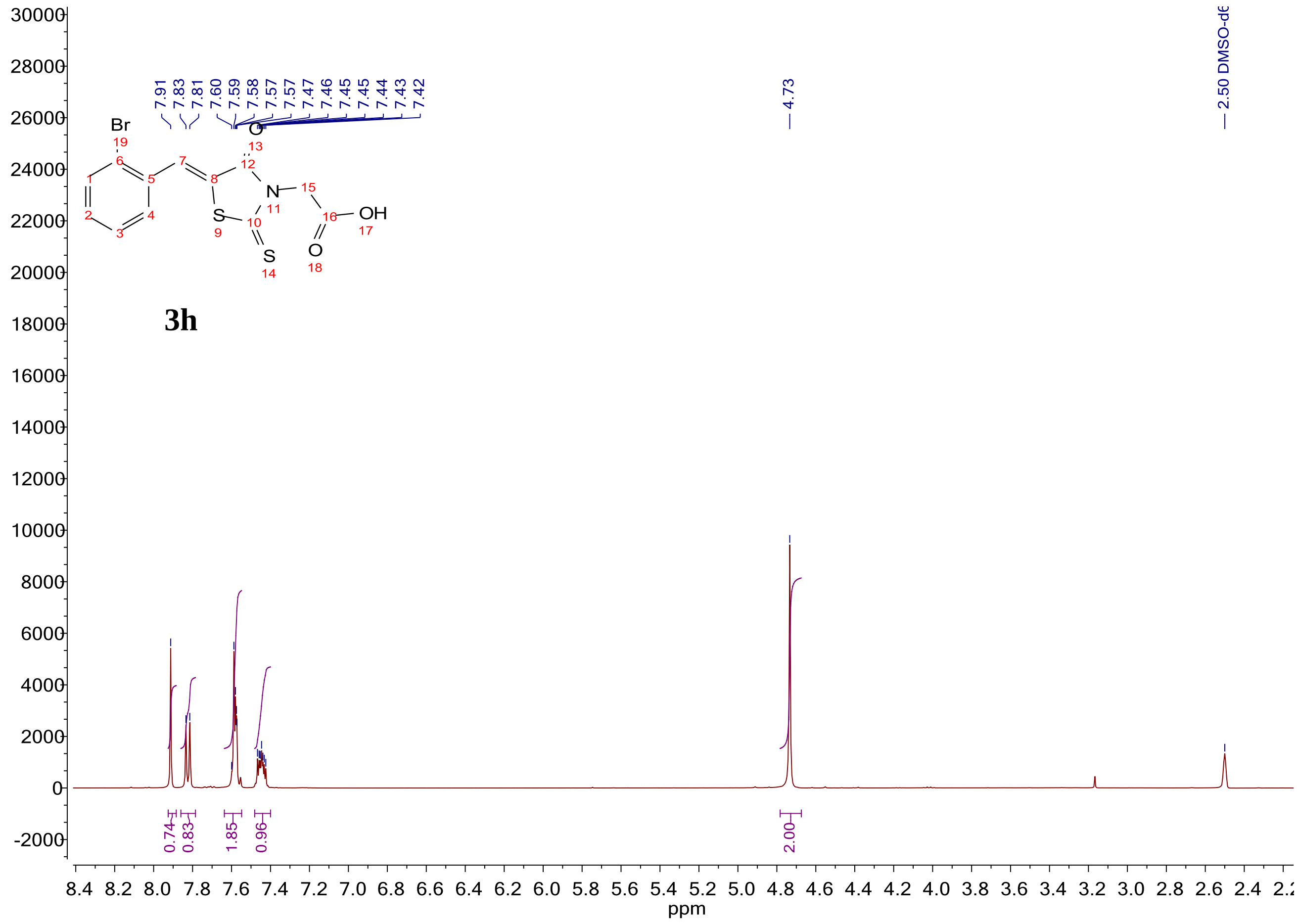
7.91
7.83
7.81
7.60
7.59
7.58
7.57
7.57
7.47
7.46
7.45
7.45
7.44
7.43
7.42
4.73
2.50 DMSO-d6
Br
O
N
OH
S
S
O
3h
0.74
0.83
1.85
0.96
2.00
ppm

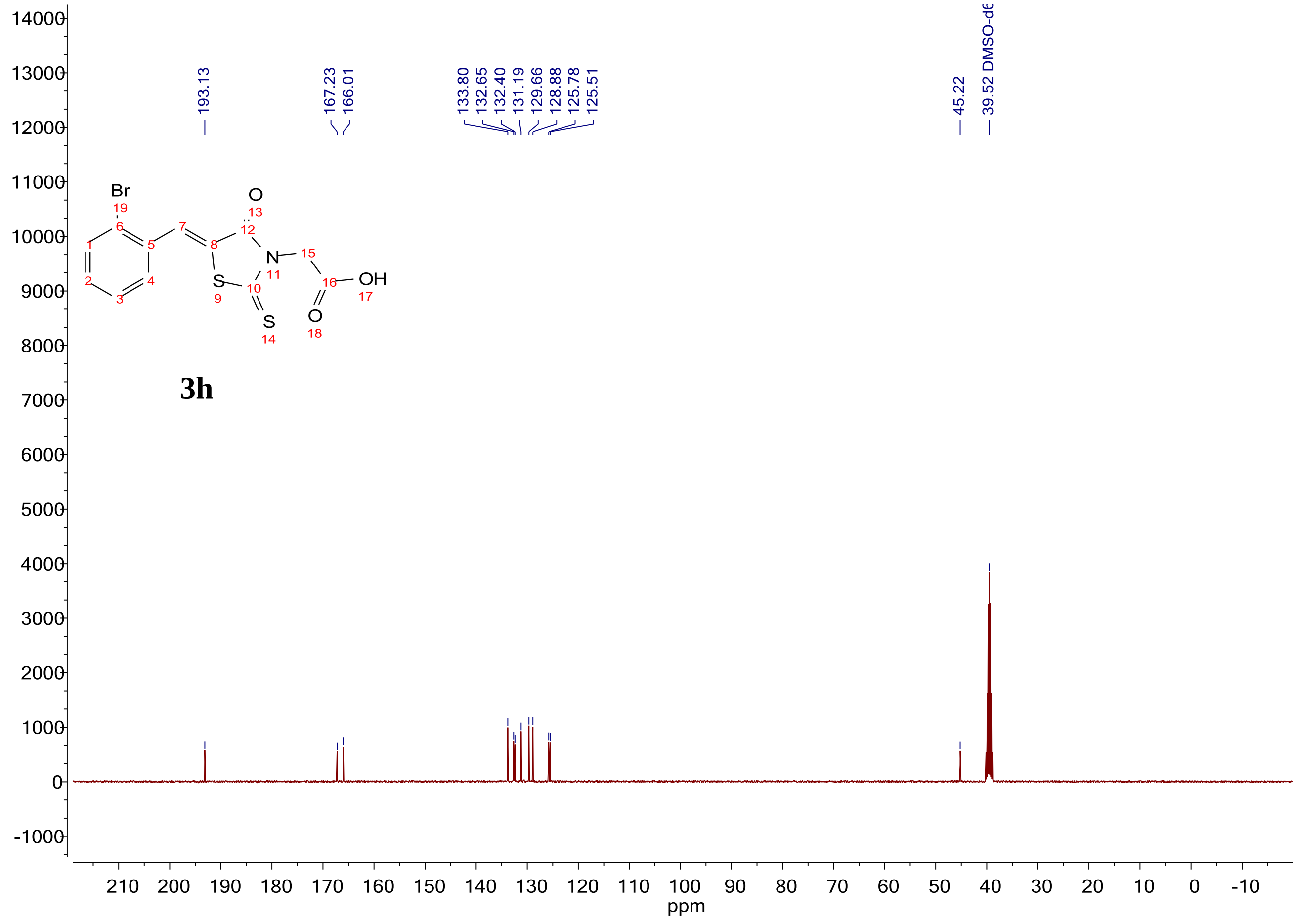
193.13
167.23
166.01
133.80
132.65
132.40
131.19
129.66
128.88
125.78
125.51
45.22
39.52 DMSO-d6
Br
O
N
OH
S
S
O
3h
ppm

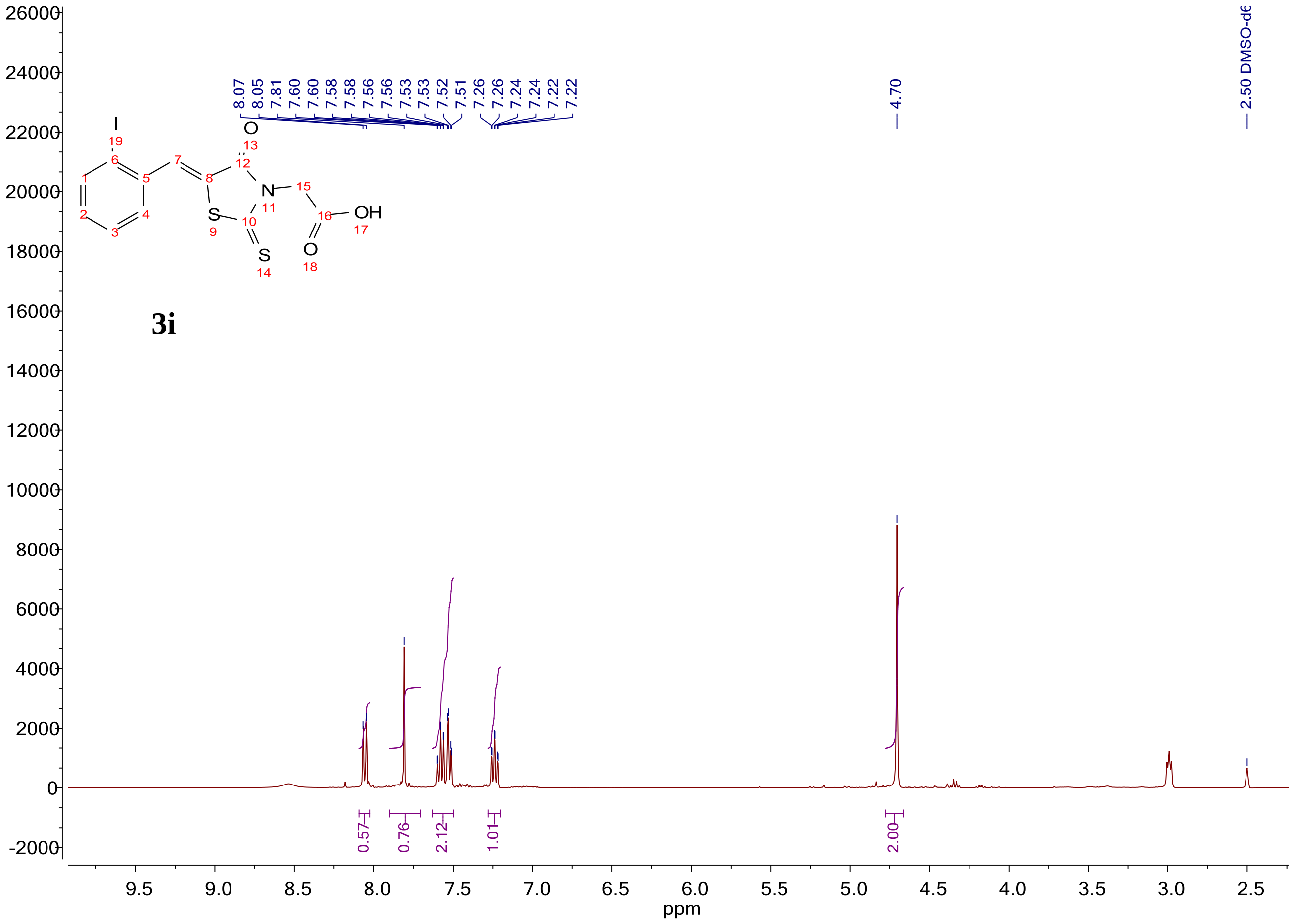
8.07 8.05 7.81 7.60 7.60 7.58 7.58 7.56 7.56 7.53 7.53 7.52 7.51 7.26 7.26 7.24 7.24 7.22 7.22
4.70
2.50 DMSO-d6
3i
0.57
0.76
2.12
1.01
2.00
ppm

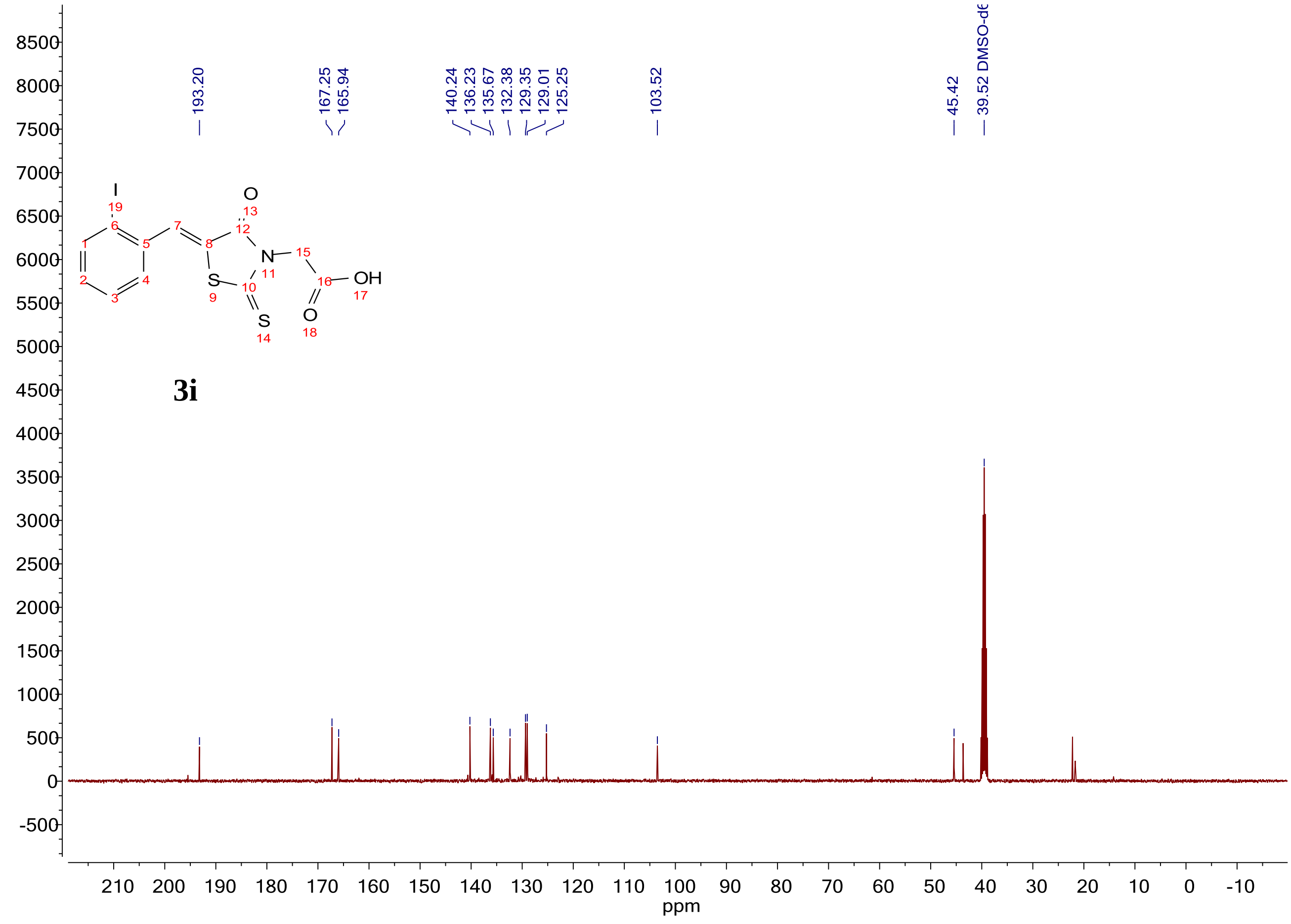
193.20
167.25 165.94
140.24 136.23 135.67 132.38 129.35 129.01 125.25
103.52
45.42
39.52 DMSO-d6
3i
ppm

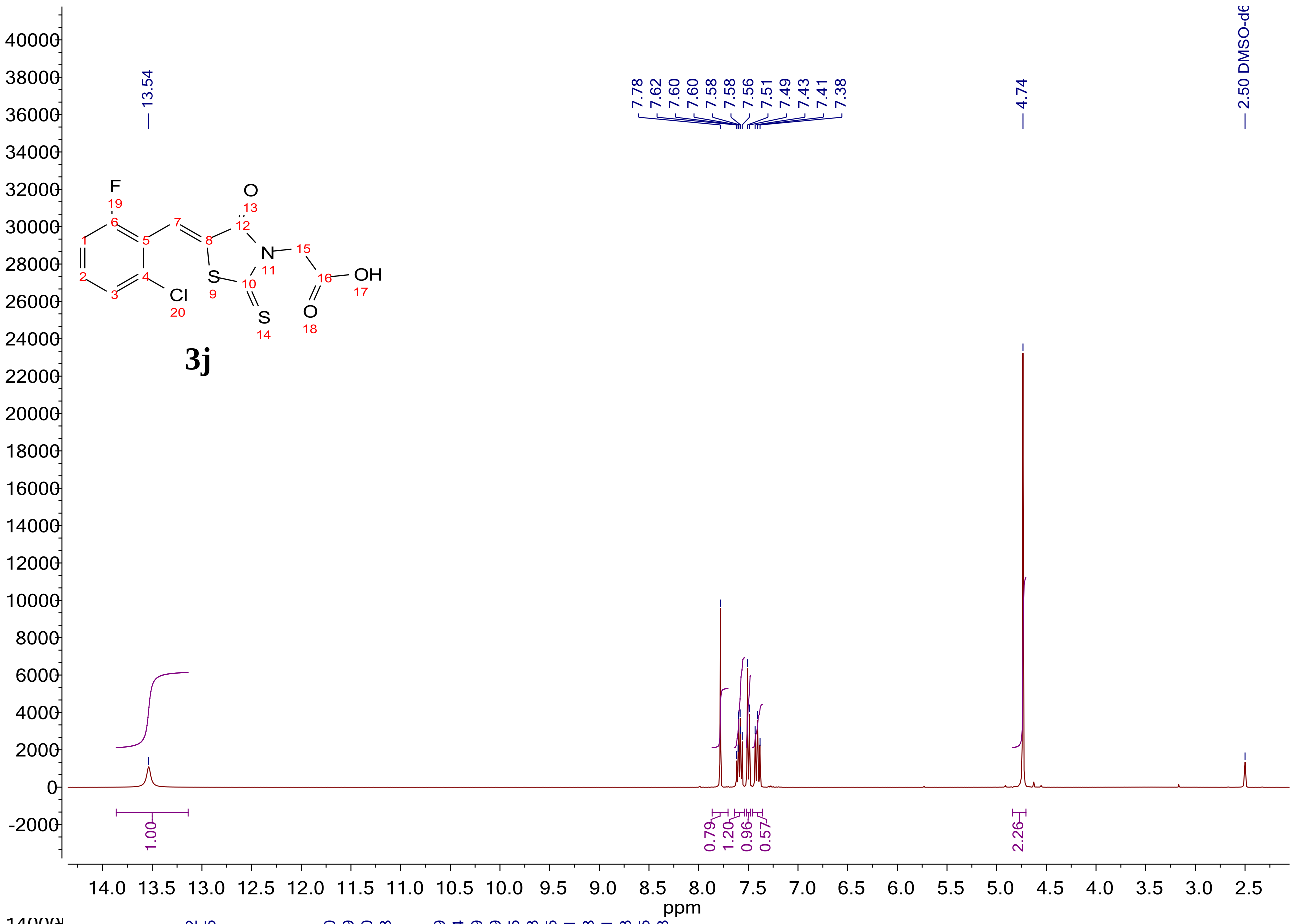
13.54
7.78
7.62
7.60
7.60
7.58
7.58
7.56
7.51
7.49
7.43
7.41
7.38
4.74
2.50 DMSO-d6
3j
1.00
0.79
1.20
0.96
0.57
2.26
ppm

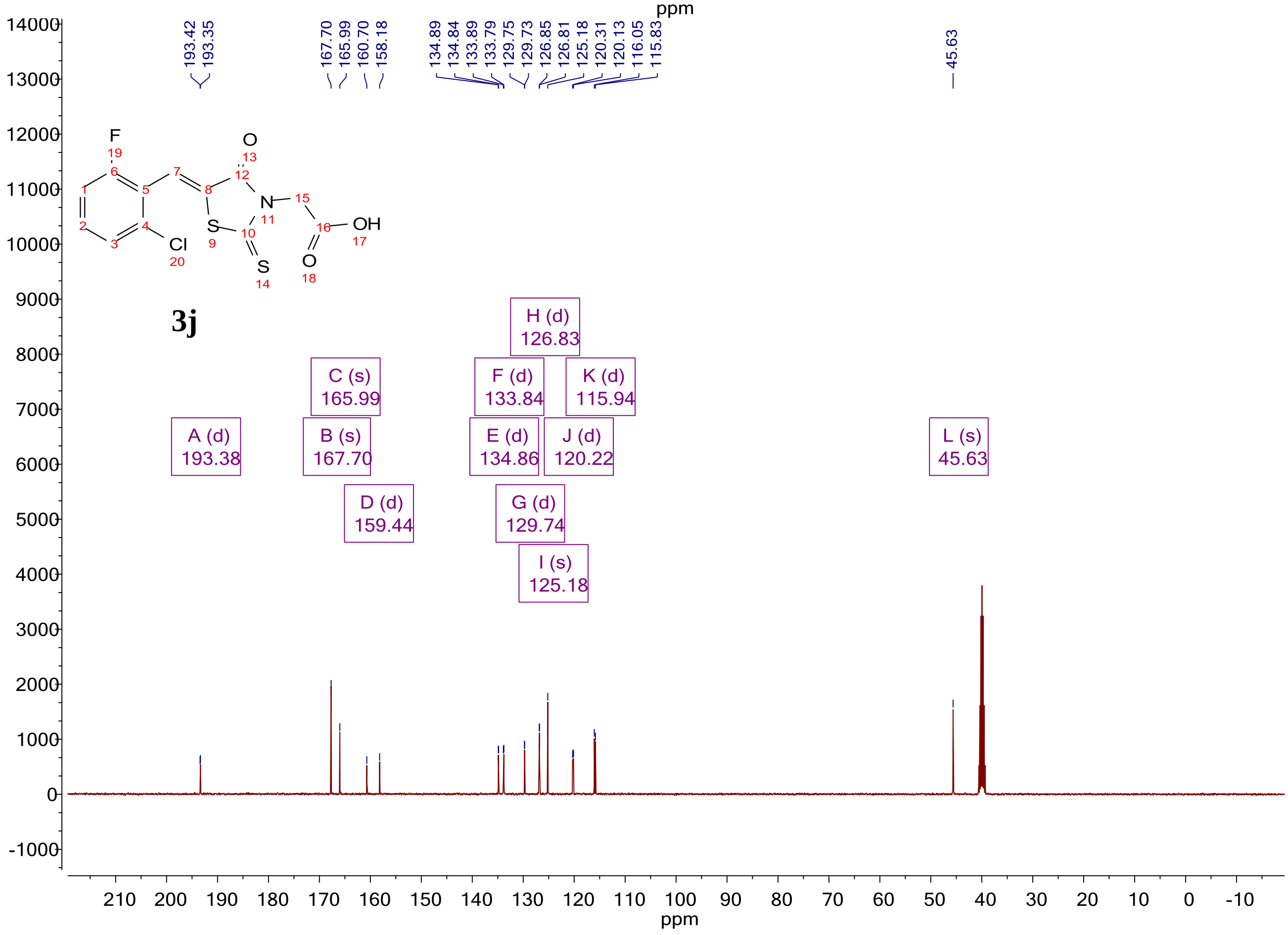
193.42
193.35
167.70
165.99
160.70
158.18
134.89
134.84
133.89
133.79
129.75
129.73
126.85
126.81
125.18
120.31
120.13
116.05
115.83
45.63
3j
H (d) 126.83
C (s) 165.99
F (d) 133.84
K (d) 115.94
A (d) 193.38
B (s) 167.70
E (d) 134.86
J (d) 120.22
L (s) 45.63
D (d) 159.44
G (d) 129.74
I (s) 125.18
ppm

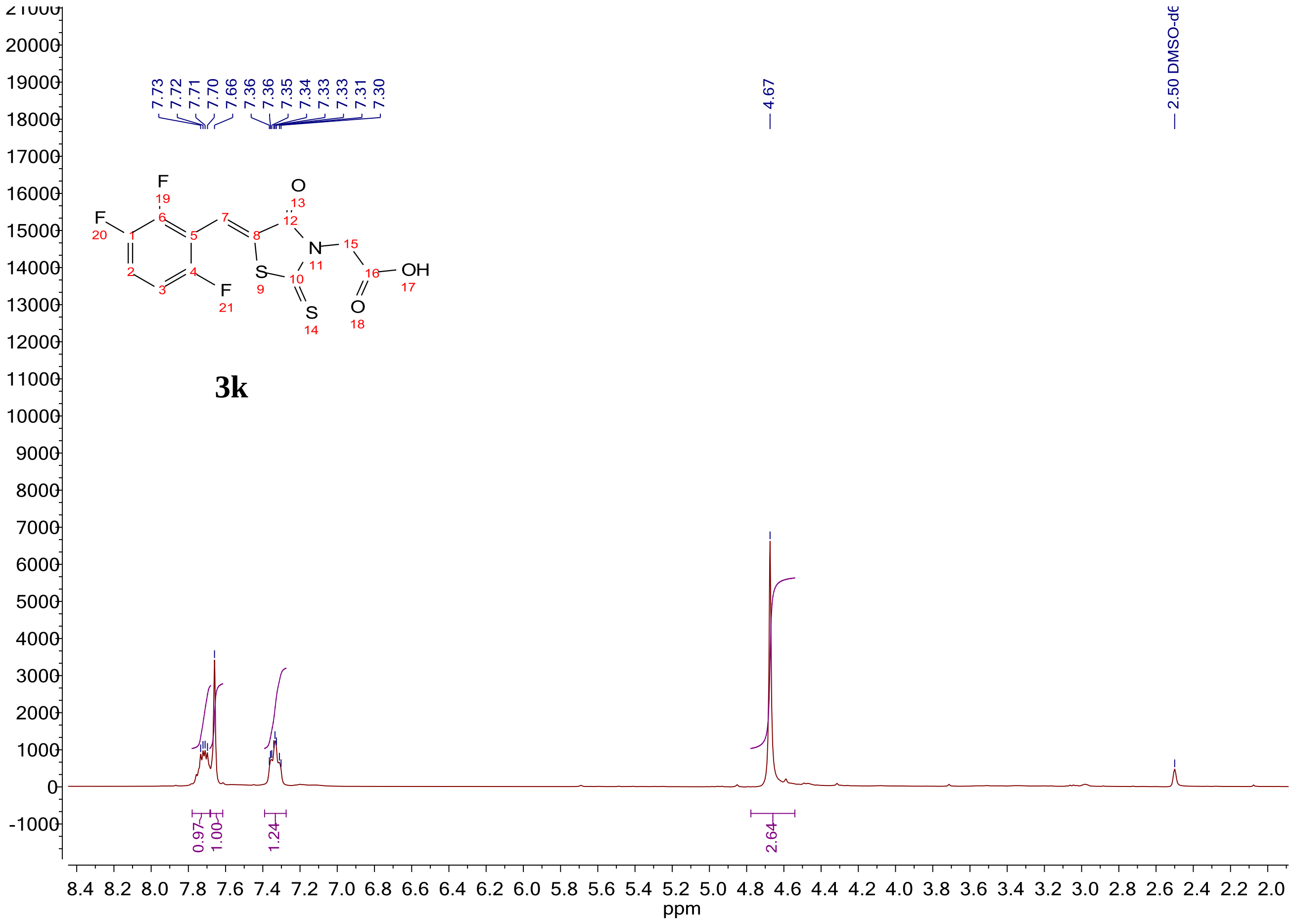

7.73
7.72
7.71
7.70
7.66
7.36
7.36
7.35
7.34
7.33
7.33
7.31
7.30
4.67
2.50 DMSO-d6
0.97
1.00
1.24
2.64
ppm

3k

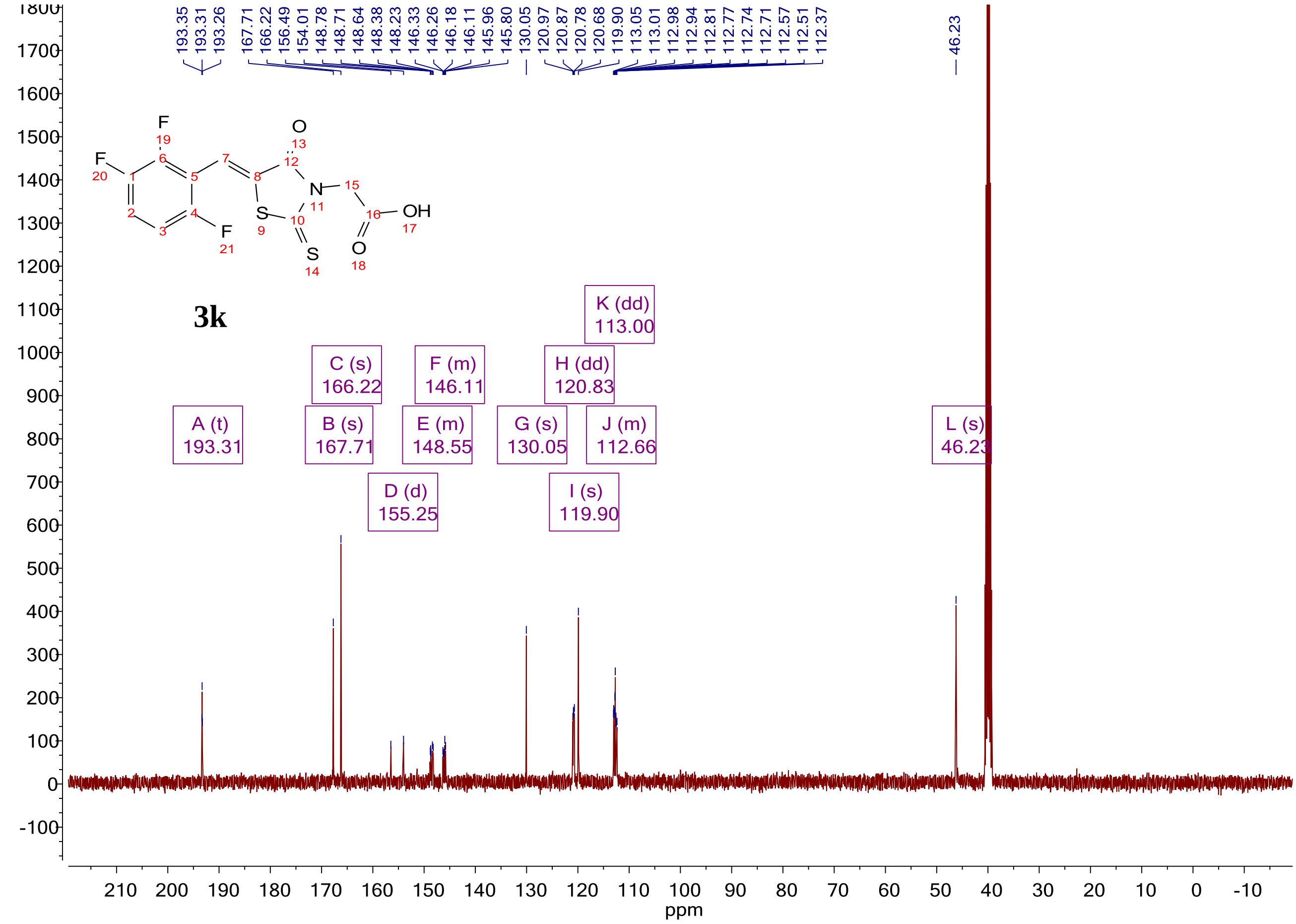

193.35
193.31
193.26
167.71
166.22
156.49
154.01
148.78
148.71
148.64
148.38
148.23
146.33
146.26
146.18
146.11
145.96
145.80
130.05
120.97
120.87
120.78
120.68
119.90
113.05
113.01
112.98
112.94
112.81
112.77
112.74
112.71
112.57
112.51
112.37
46.23
A (t) 193.31
B (s) 167.71
C (s) 166.22
D (d) 155.25
E (m) 148.55
F (m) 146.11
G (s) 130.05
H (dd) 120.83
I (s) 119.90
J (m) 112.66
K (dd) 113.00
L (s) 46.23
ppm

3k

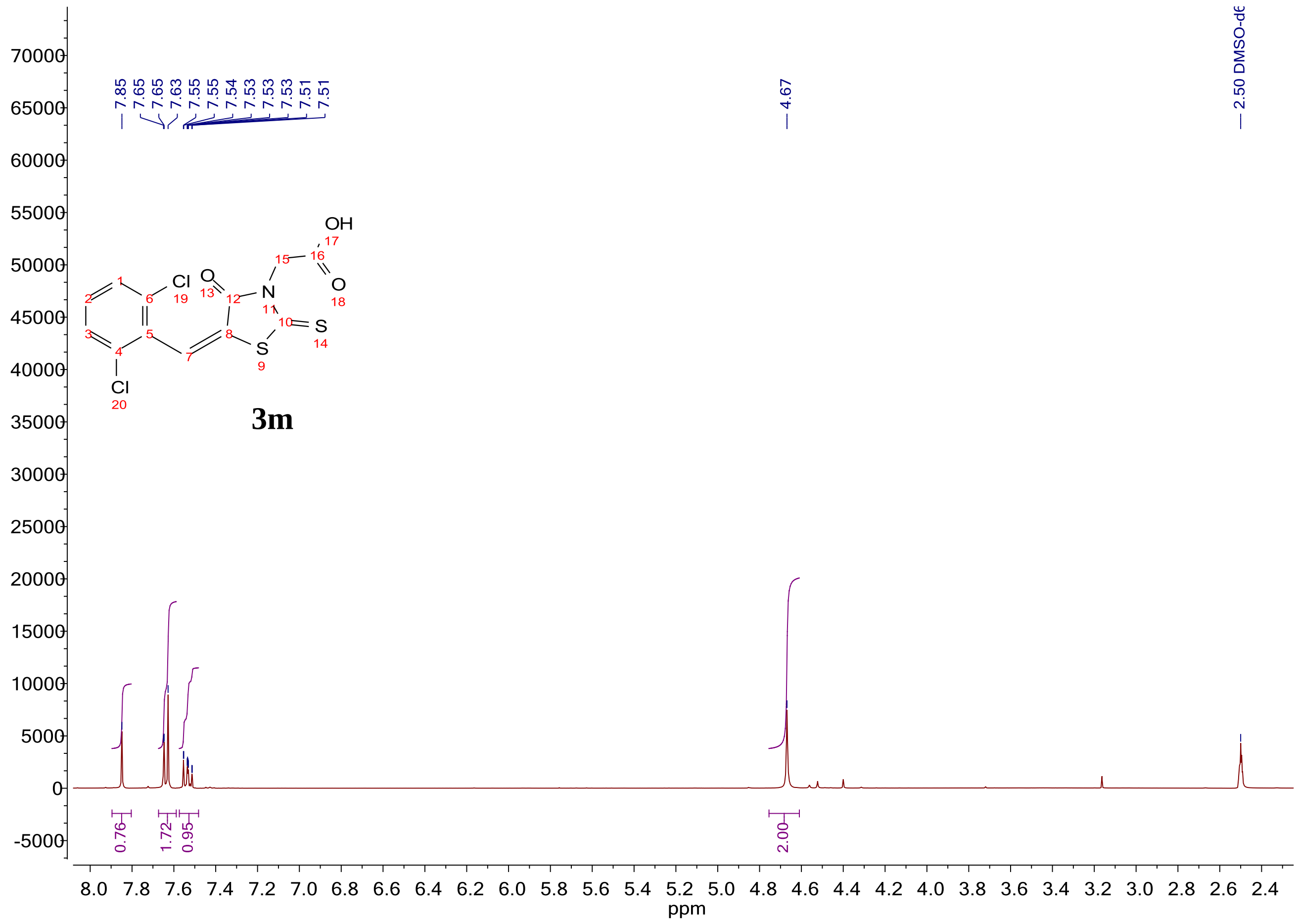
7.85
7.65
7.65
7.63
7.55
7.55
7.54
7.53
7.53
7.53
7.51
7.51
4.67
2.50 DMSO-d6
3m
0.76
1.72
0.95
2.00
ppm

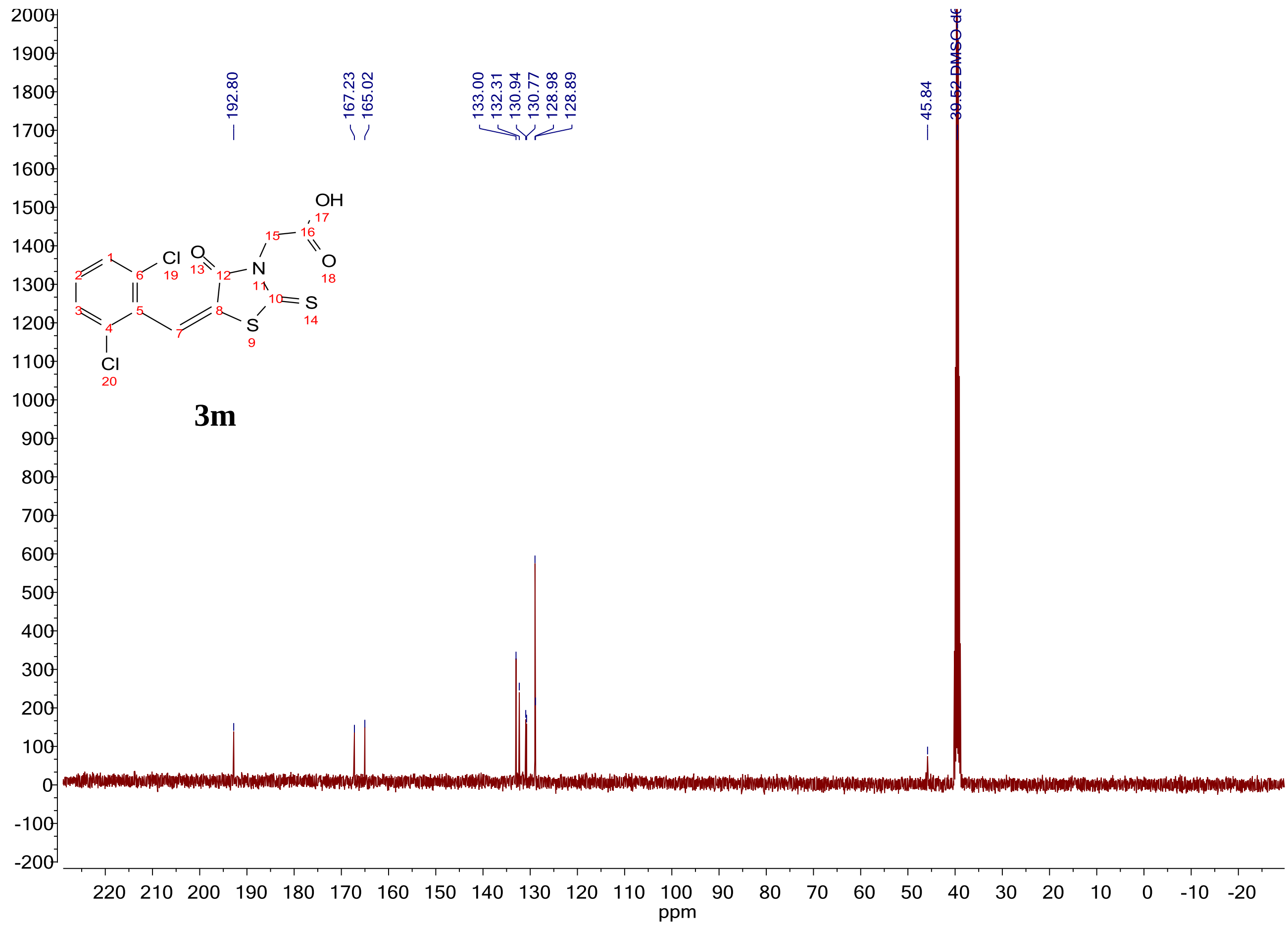
192.80
167.23
165.02
133.00
132.31
130.94
130.77
128.98
128.89
45.84
39.52 DMSO-d6
3m
ppm

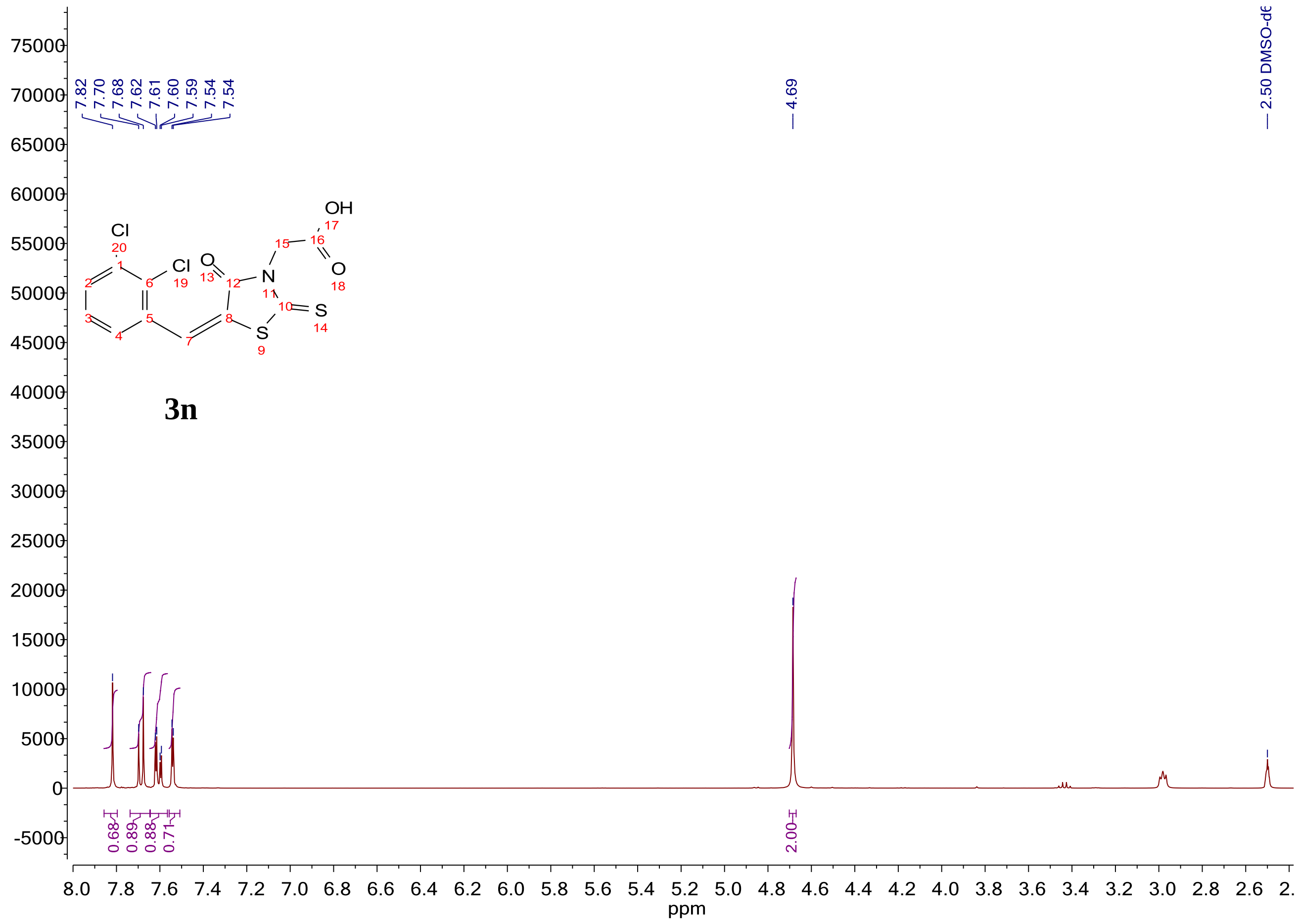
7.82
7.70
7.68
7.62
7.61
7.60
7.59
7.54
7.54
4.69
2.50 DMSO-d6
Cl
Cl
O
N
OH
O
S
S
3n
0.68
0.89
0.88
0.71
2.00
ppm

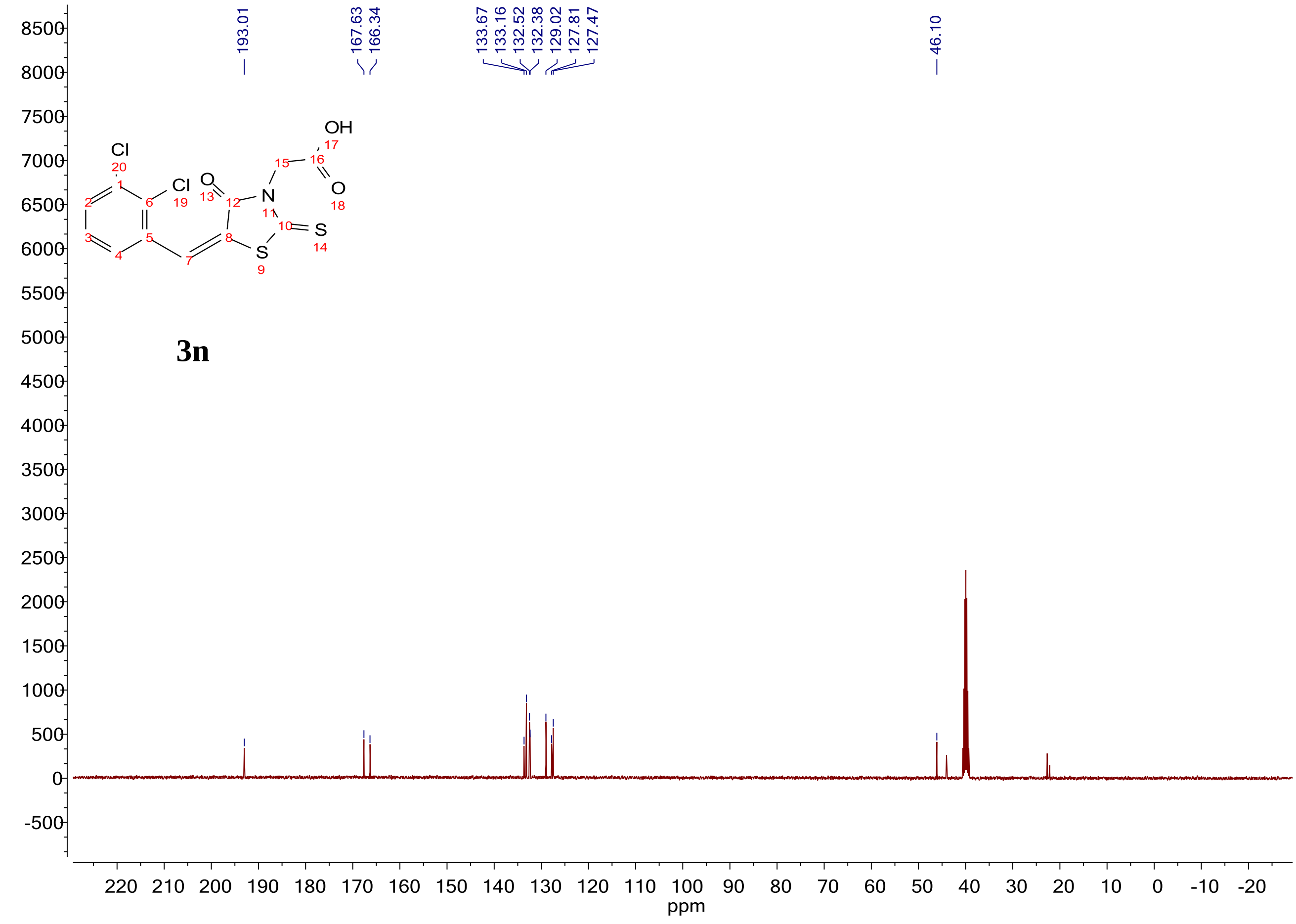
193.01
167.63
166.34
133.67
133.16
132.52
132.38
129.02
127.81
127.47
46.10
Cl
Cl
O
N
OH
O
S
S
3n
ppm

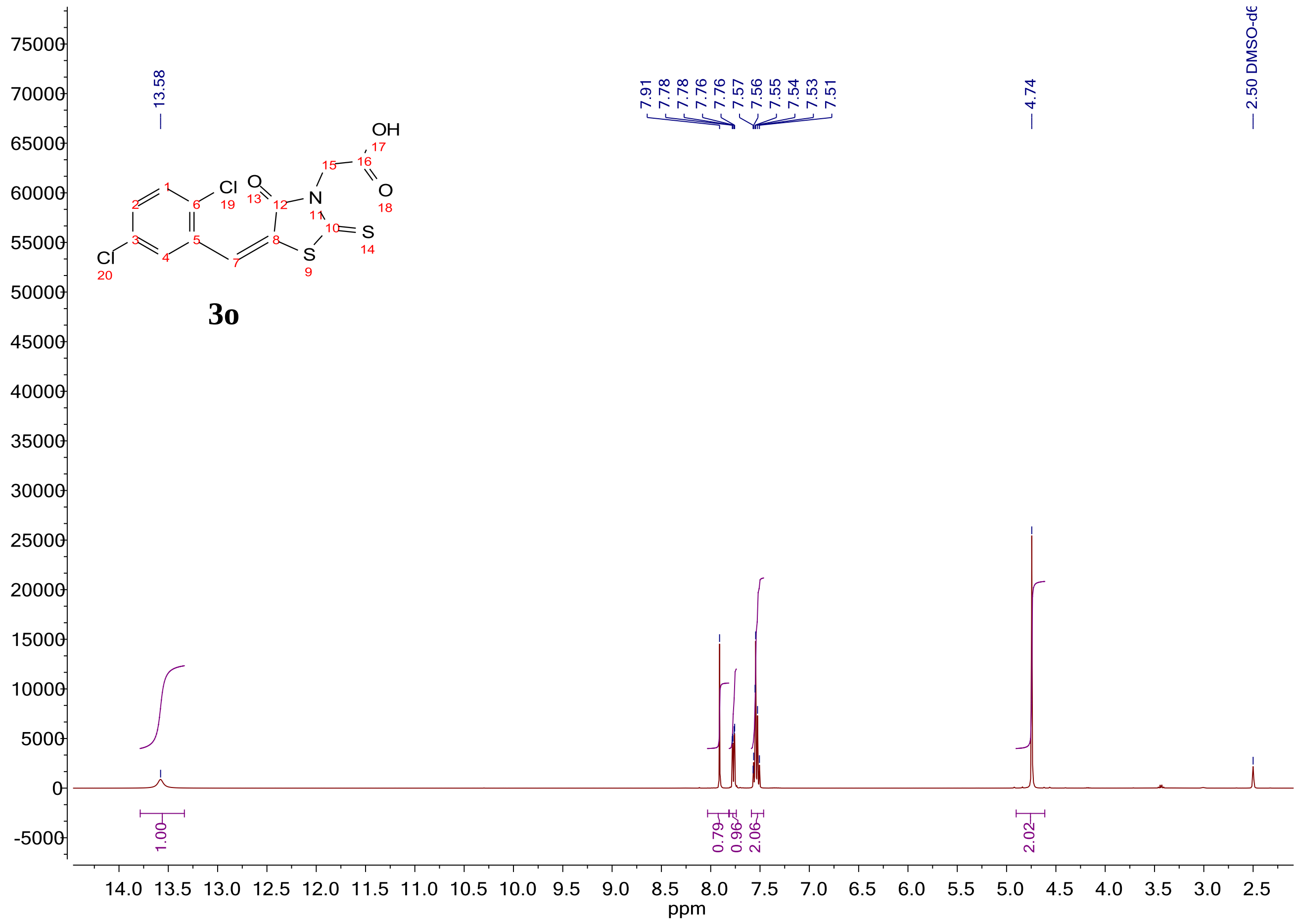
13.58
7.91
7.78
7.78
7.76
7.76
7.57
7.56
7.55
7.54
7.53
7.51
4.74
2.50 DMSO-d6
1.00
0.79
0.96
2.06
2.02
ppm
OH
O
N
S
Cl
Cl

3o

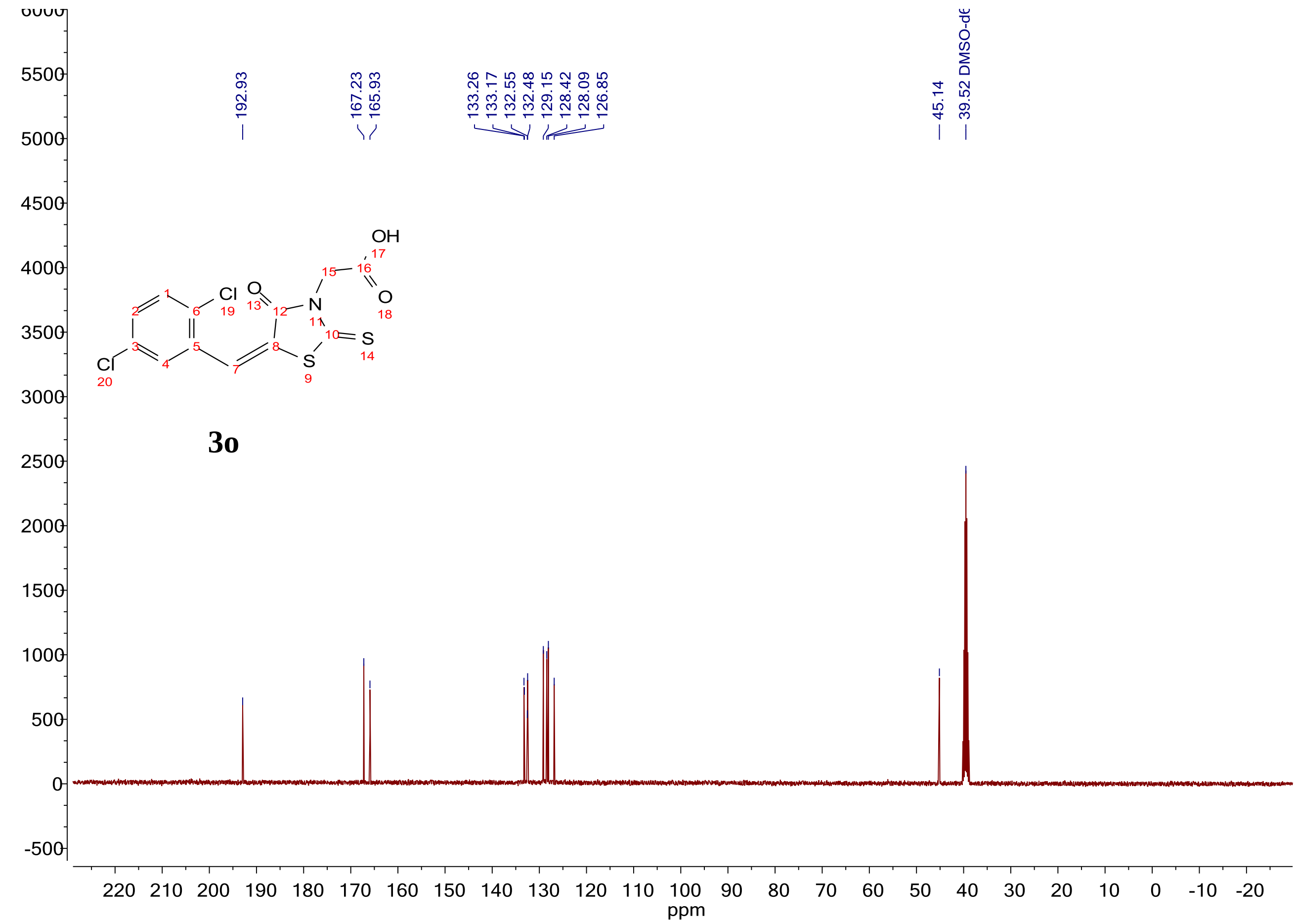
192.93
167.23
165.93
133.26
133.17
132.55
132.48
129.15
128.42
128.09
126.85
45.14
39.52 DMSO-d6
ppm

3o

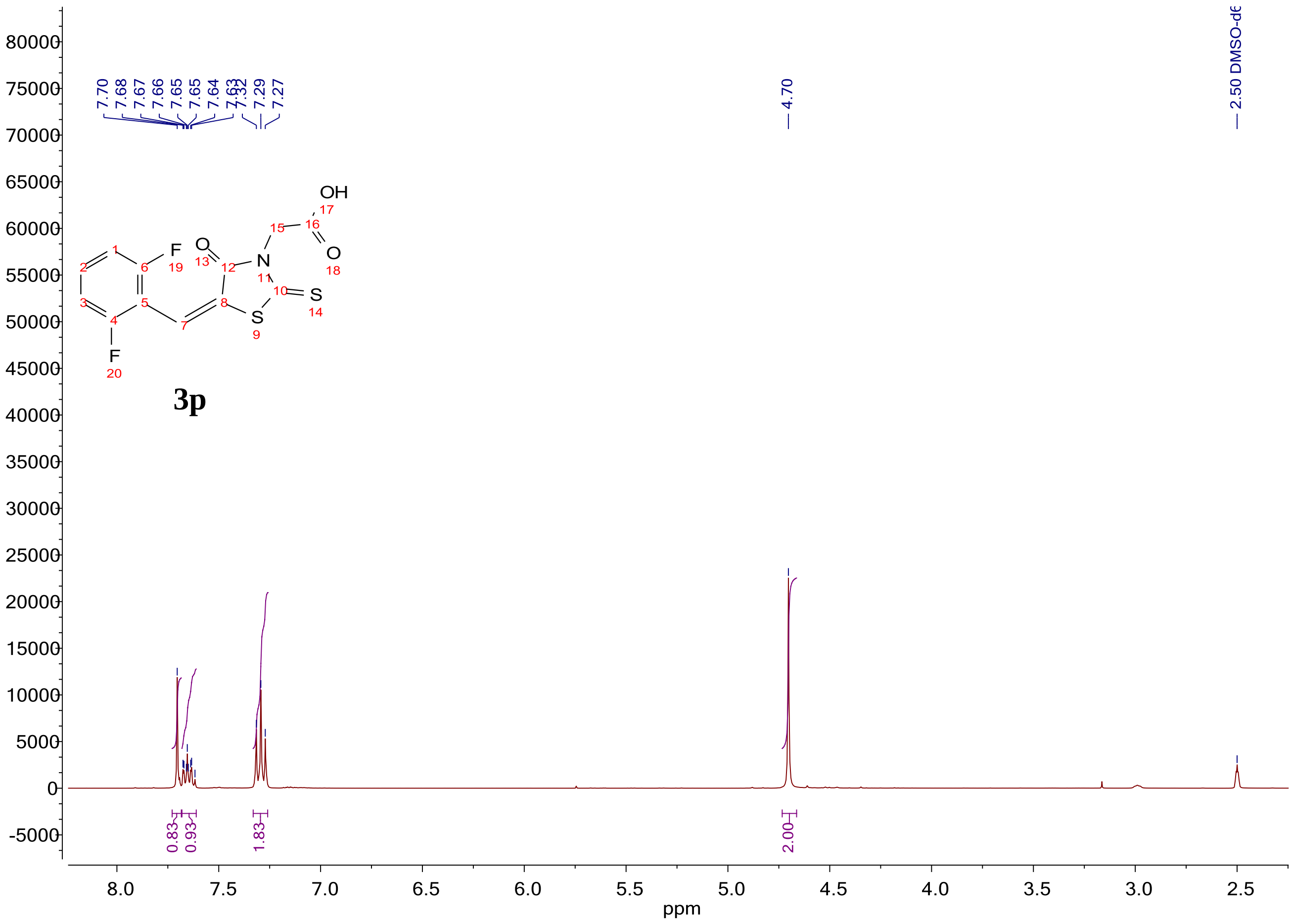

7.70
7.68
7.67
7.66
7.65
7.65
7.64
7.63
7.32
7.29
7.27
4.70
2.50 DMSO-d6
OH
O
F
O
N
S
S
F
0.83
0.93
1.83
2.00
ppm

3p

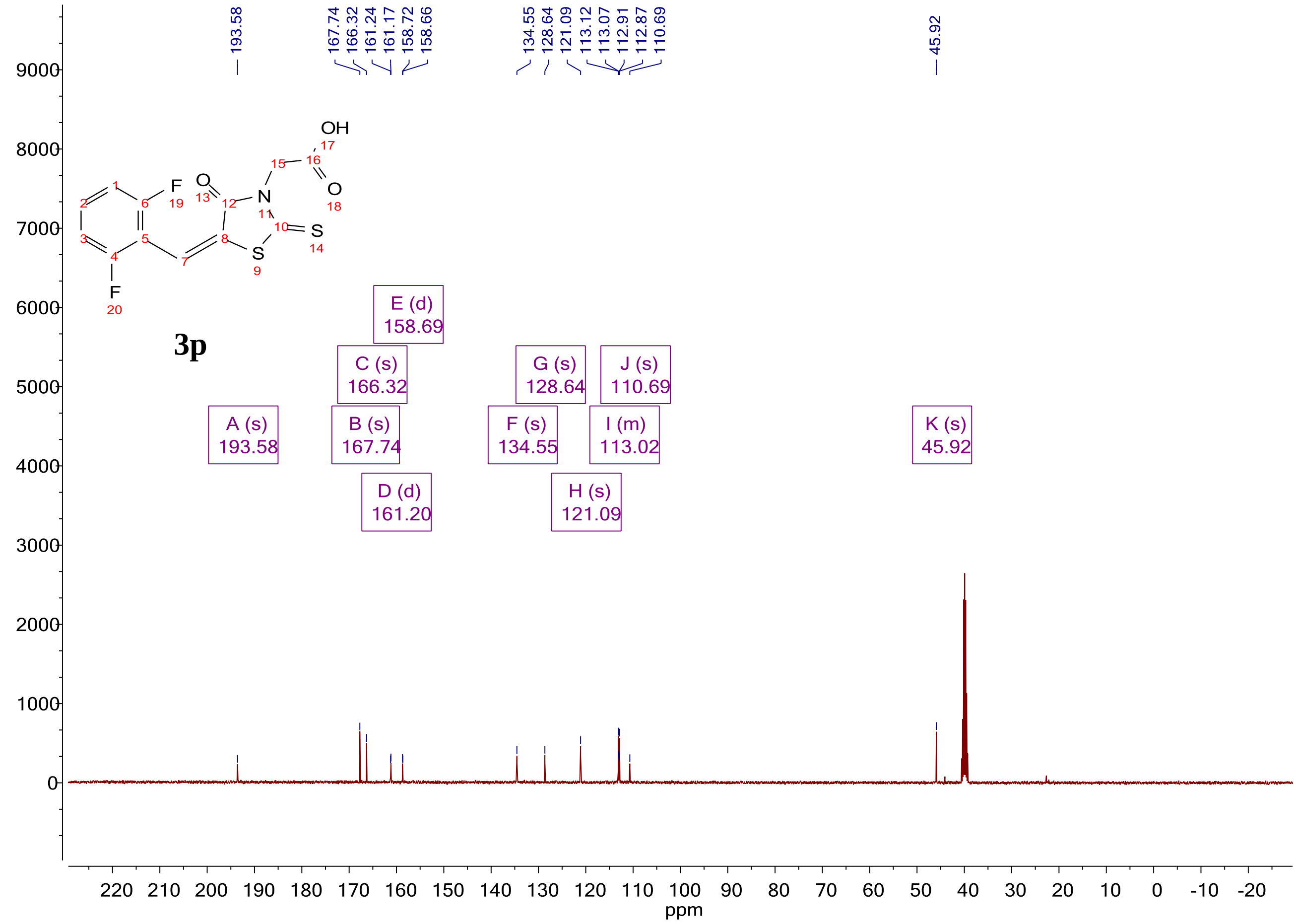

193.58
167.74
166.32
161.24
161.17
158.72
158.66
134.55
128.64
121.09
113.12
113.07
112.91
112.87
110.69
45.92
A (s) 193.58
B (s) 167.74
C (s) 166.32
D (d) 161.20
E (d) 158.69
F (s) 134.55
G (s) 128.64
H (s) 121.09
I (m) 113.02
J (s) 110.69
K (s) 45.92
ppm

3p

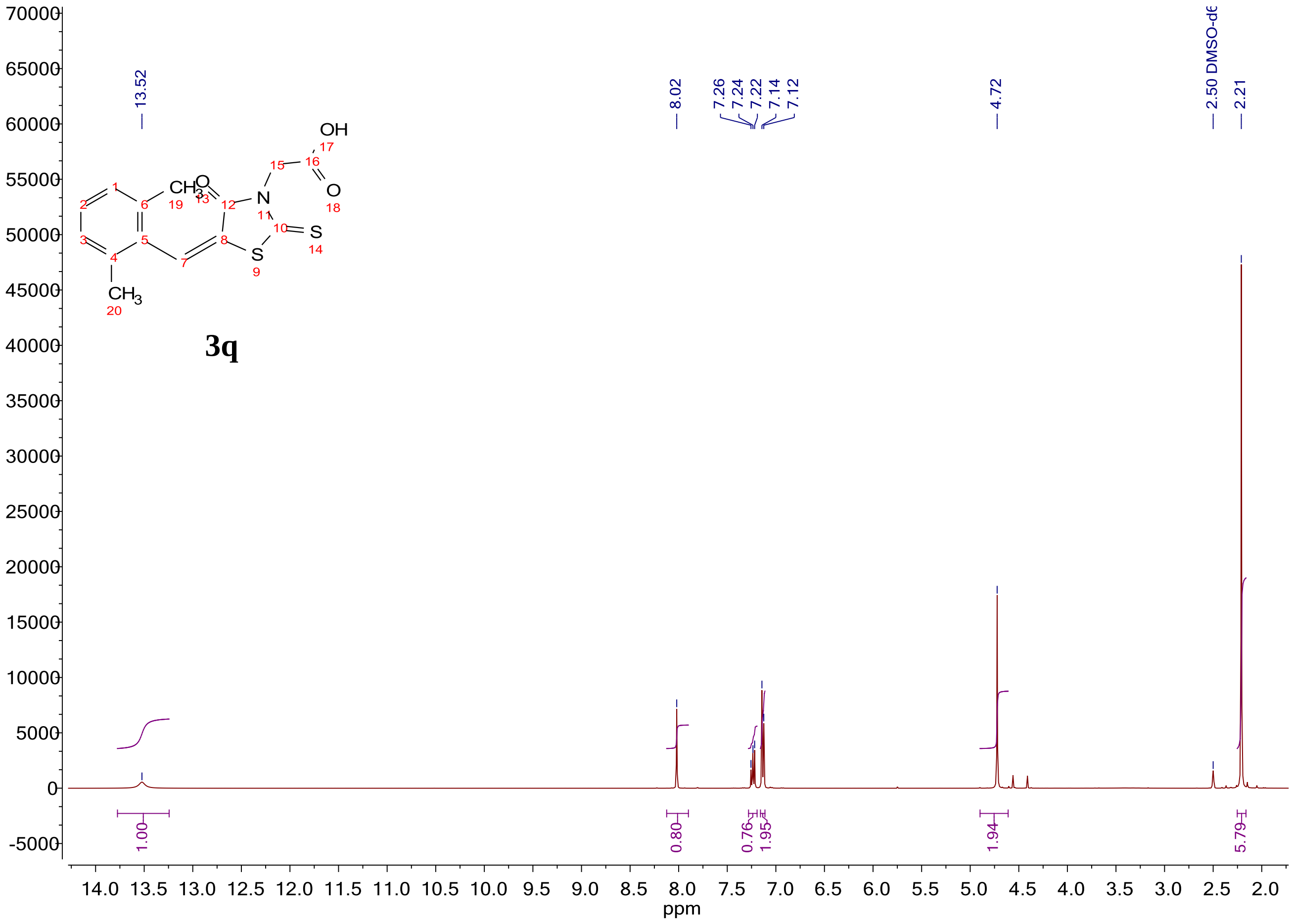
13.52
8.02
7.26
7.24
7.22
7.14
7.12
4.72
2.50 DMSO-d6
2.21
OH
N
S
CH3
3q
1.00
0.80
0.76
1.95
1.94
5.79
ppm

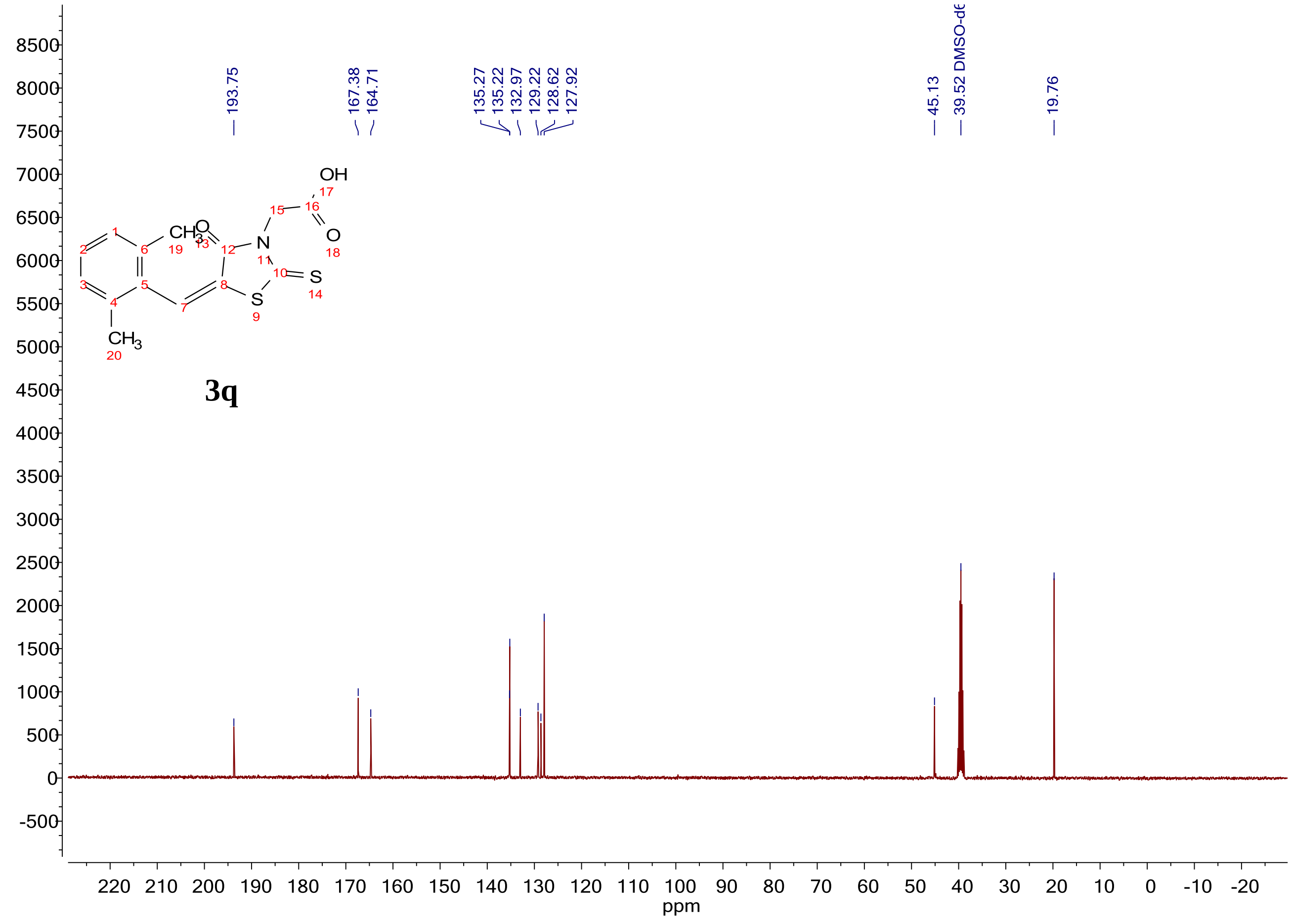
193.75
167.38
164.71
135.27
135.22
132.97
129.22
128.62
127.92
45.13
39.52 DMSO-d6
19.76
OH
N
S
CH3
3q
ppm

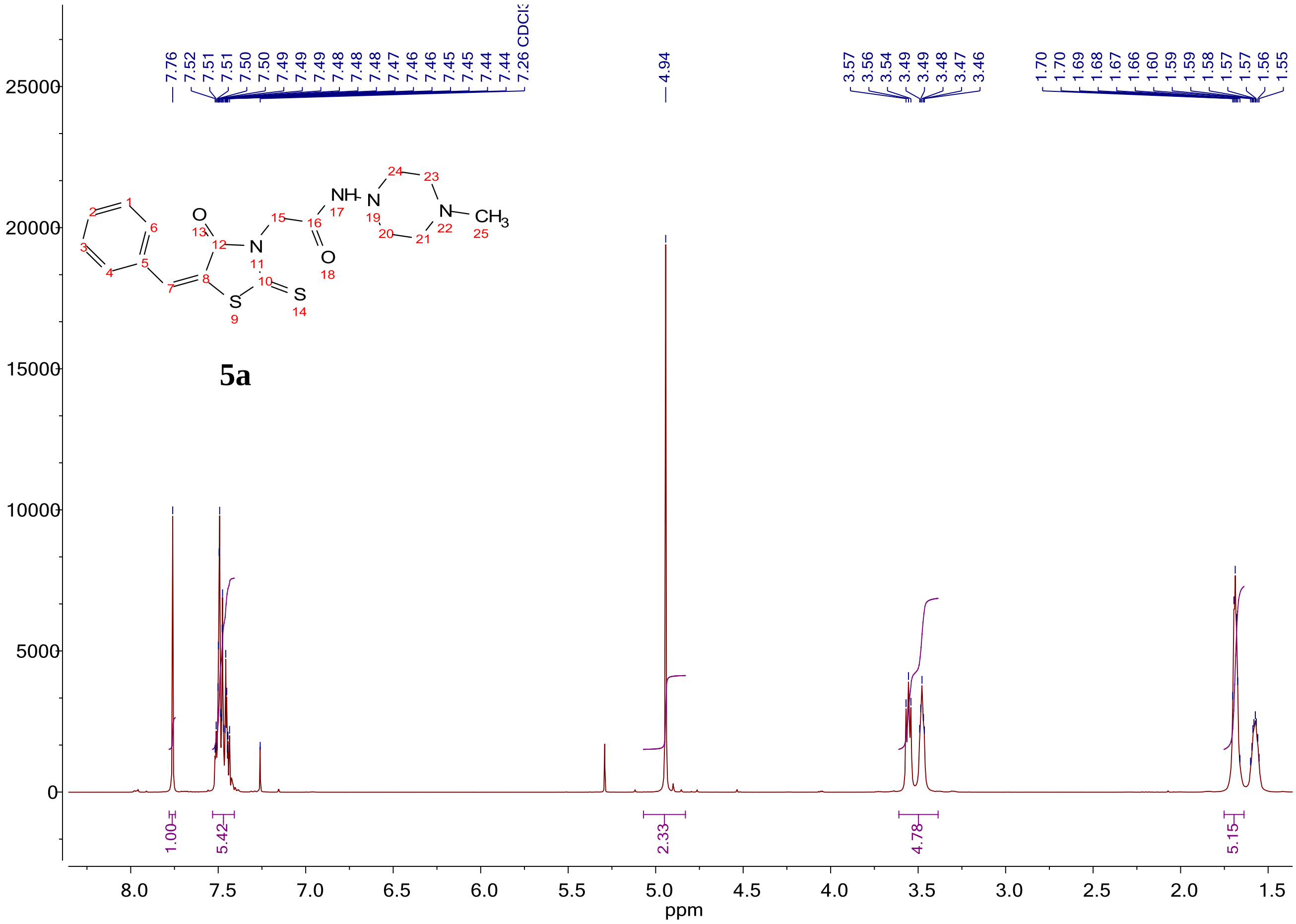
7.76
7.52
7.51
7.51
7.50
7.50
7.49
7.49
7.49
7.48
7.48
7.48
7.47
7.46
7.46
7.45
7.45
7.44
7.44
7.26 CDCl3
4.94
3.57
3.56
3.54
3.49
3.49
3.48
3.47
3.46
1.70
1.70
1.69
1.68
1.67
1.66
1.60
1.59
1.59
1.58
1.57
1.57
1.56
1.55
5a
1.00
5.42
2.33
4.78
5.15
ppm

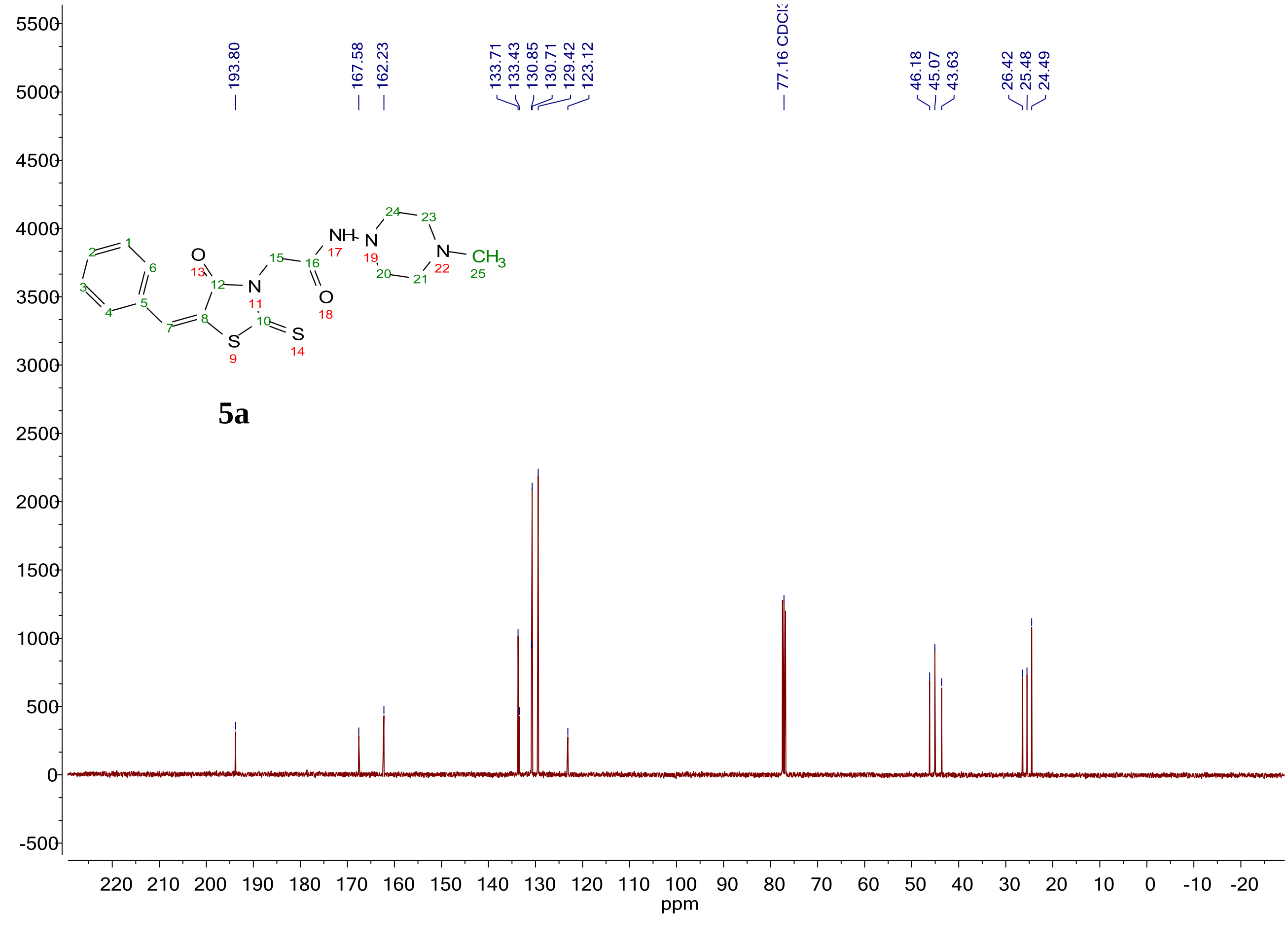
193.80
167.58
162.23
133.71
133.43
130.85
130.71
129.42
123.12
77.16 CDCl3
46.18
45.07
43.63
26.42
25.48
24.49
5a
ppm

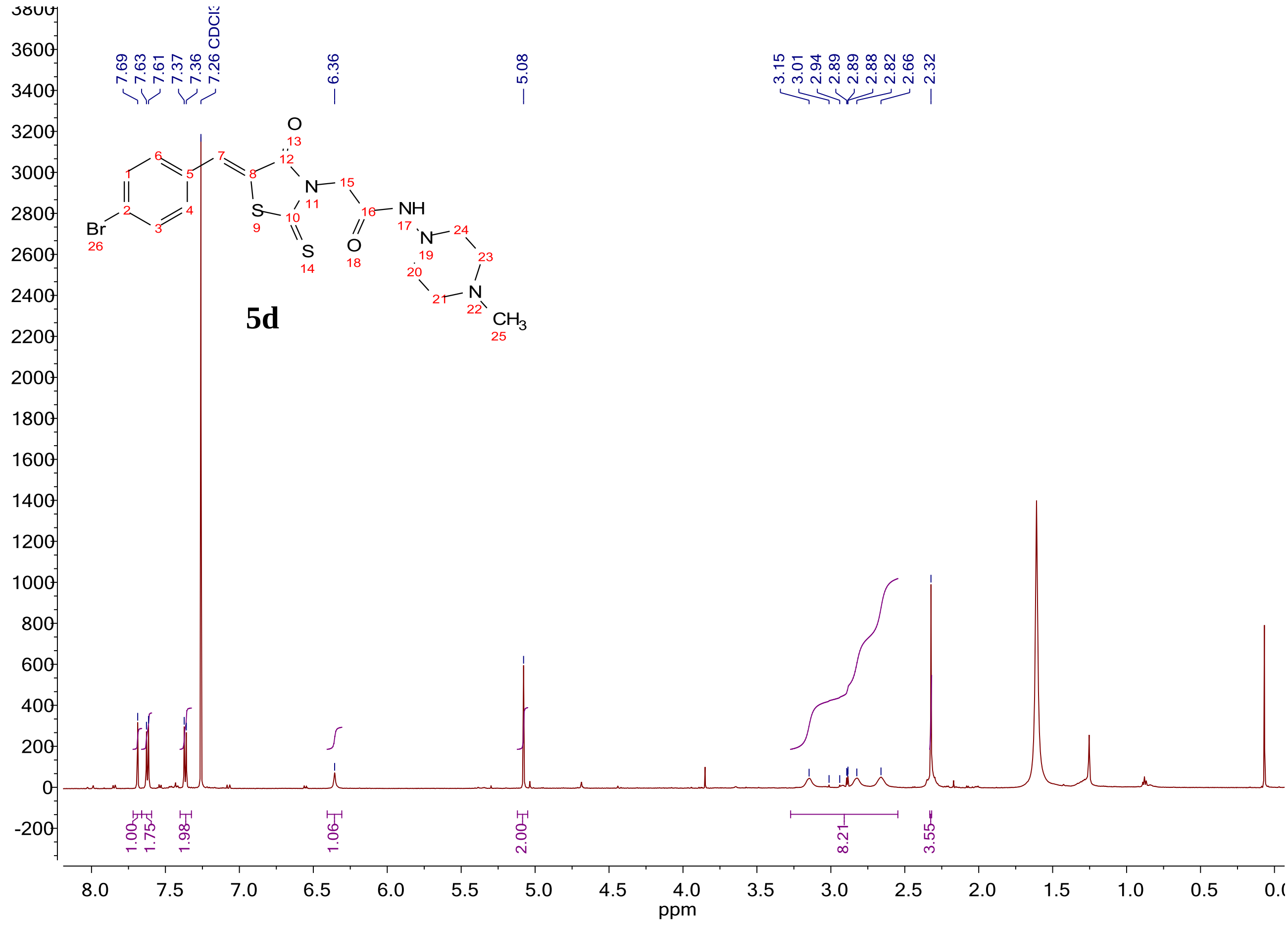

7.69
7.63
7.61
7.37
7.36
7.26 CDCl3
6.36
5.08
3.15
3.01
2.94
2.89
2.89
2.88
2.82
2.66
2.32
5d
1.00
1.75
1.98
1.06
2.00
8.21
3.55
ppm

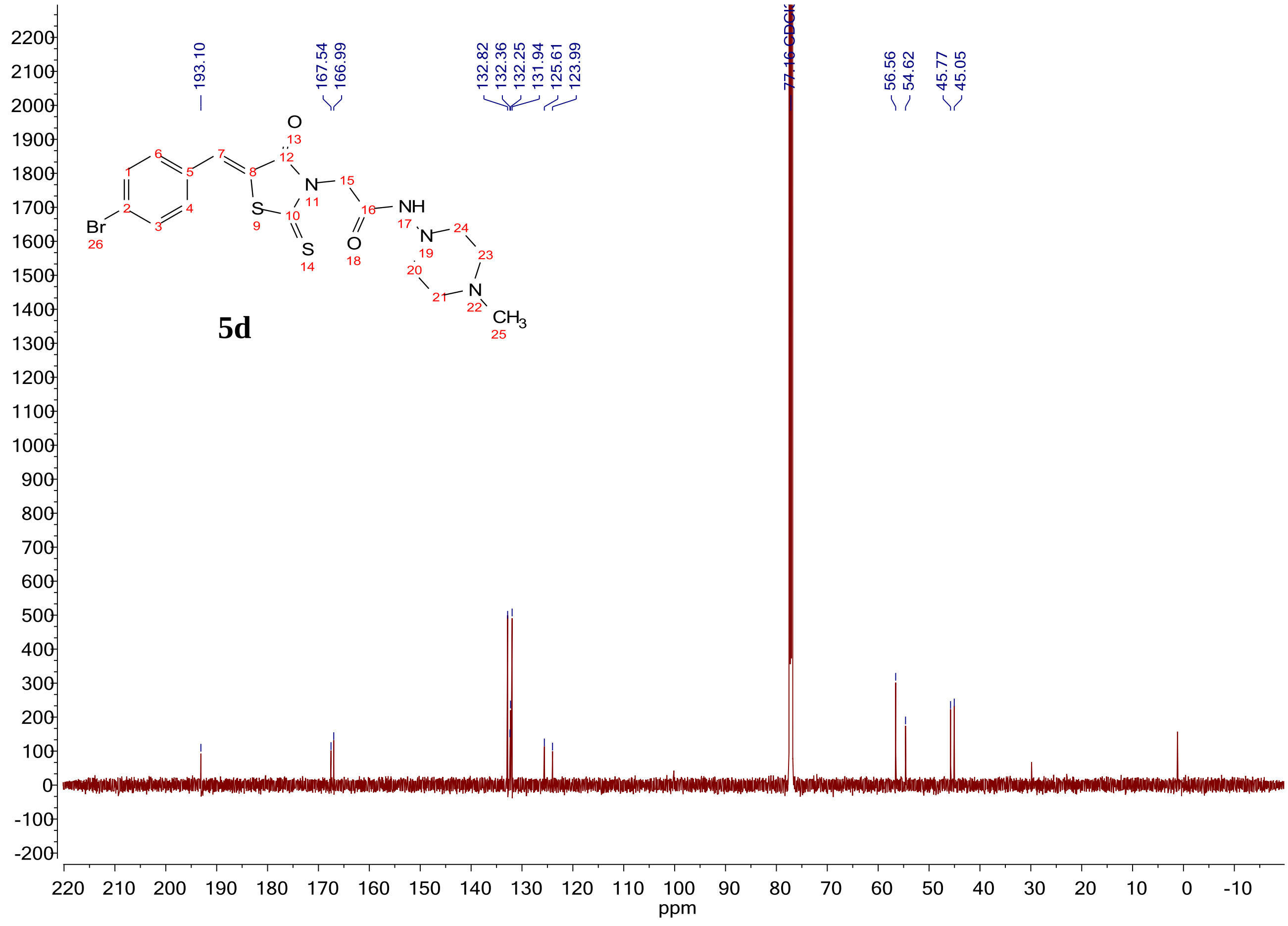

193.10
167.54
166.99
132.82
132.36
132.25
131.94
125.61
123.99
77.16 CDCl3
56.56
54.62
45.77
45.05
5d
ppm

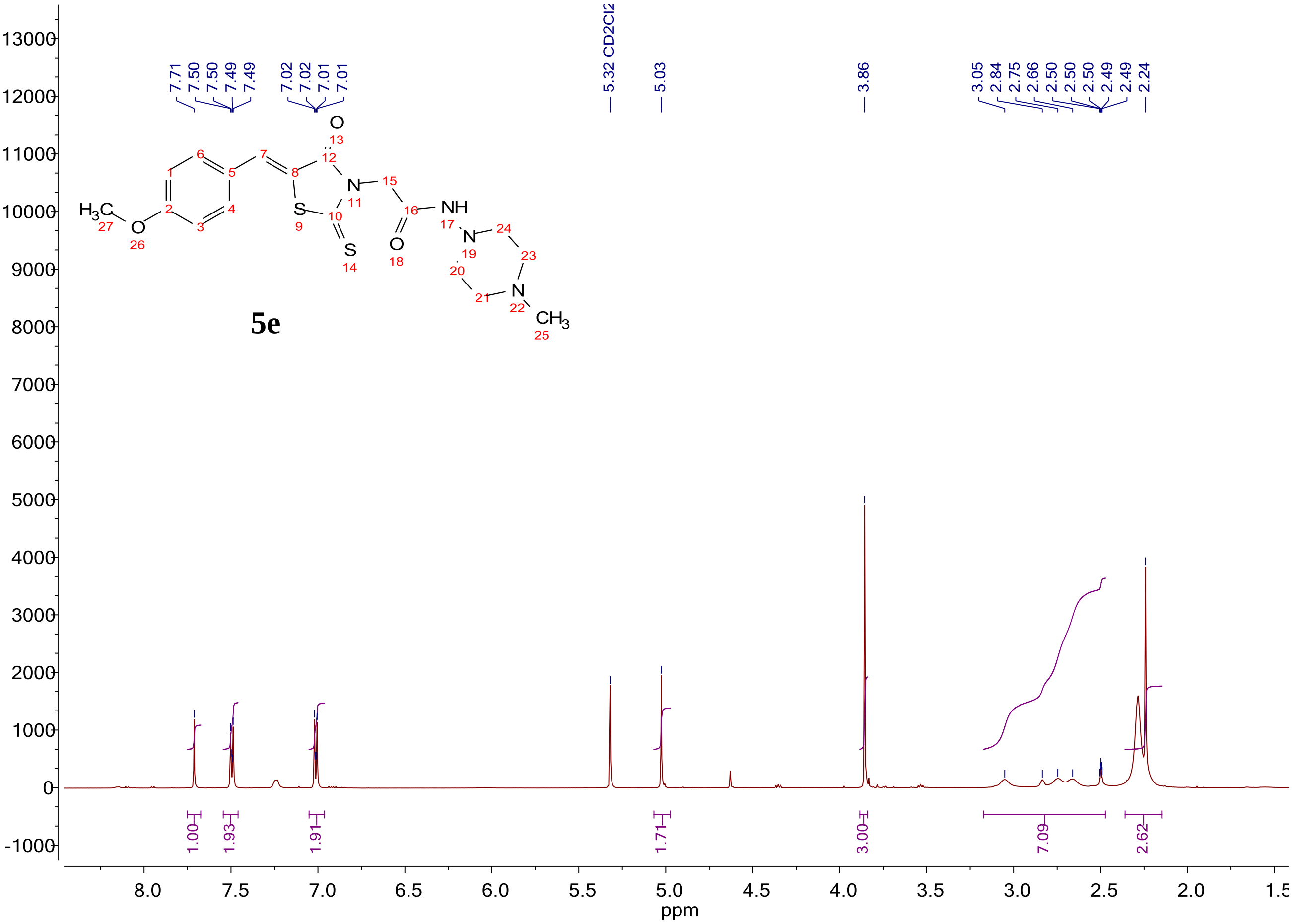
7.71
7.50
7.50
7.49
7.49
7.02
7.02
7.01
7.01
5.32 CD2Cl2
5.03
3.86
3.05
2.84
2.75
2.66
2.50
2.50
2.50
2.49
2.49
2.24
5e
1.00
1.93
1.91
1.71
3.00
7.09
2.62
ppm

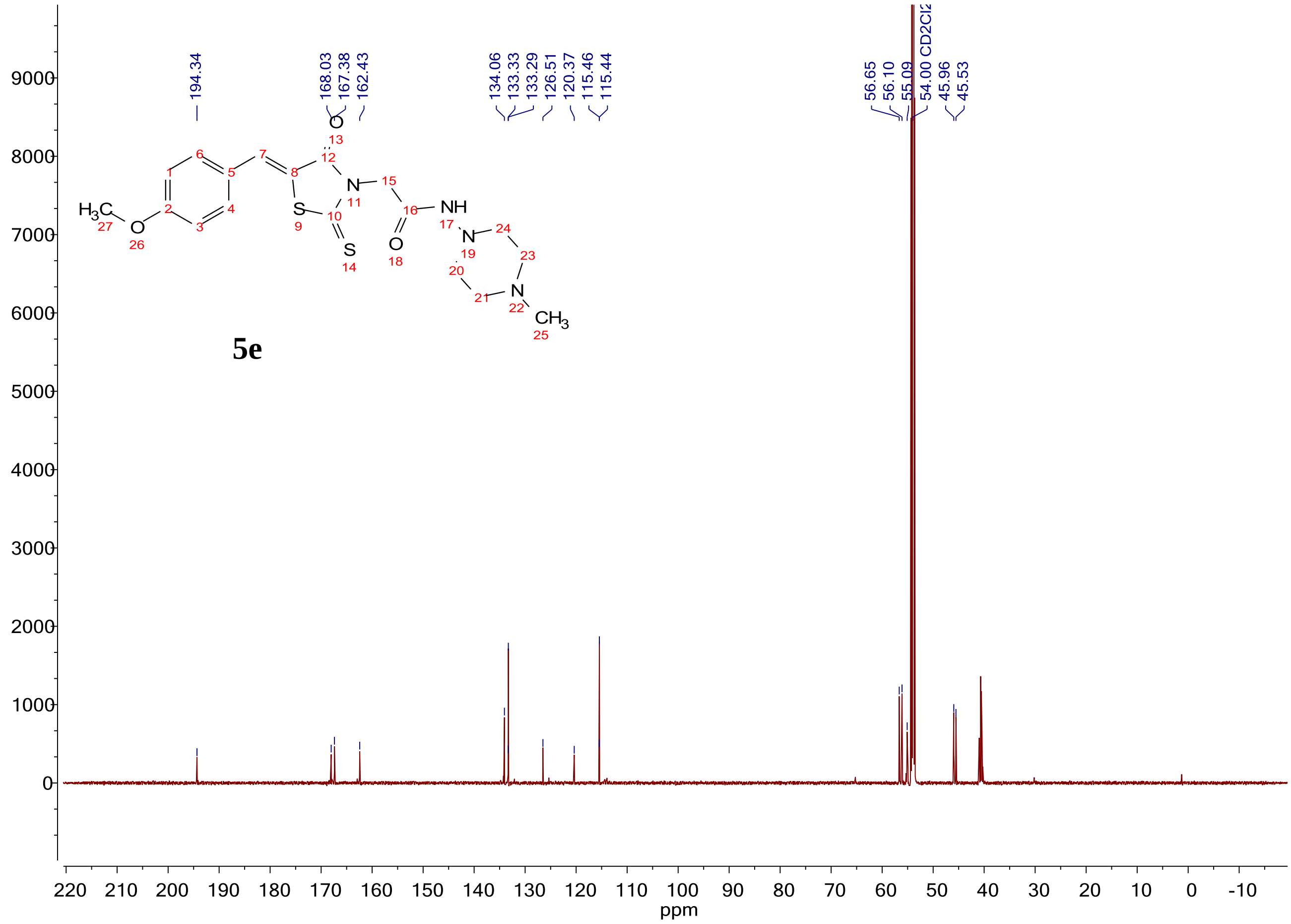
194.34
168.03
167.38
162.43
134.06
133.33
133.29
126.51
120.37
115.46
115.44
56.65
56.10
55.09
54.00 CD2Cl2
45.96
45.53
5e
ppm

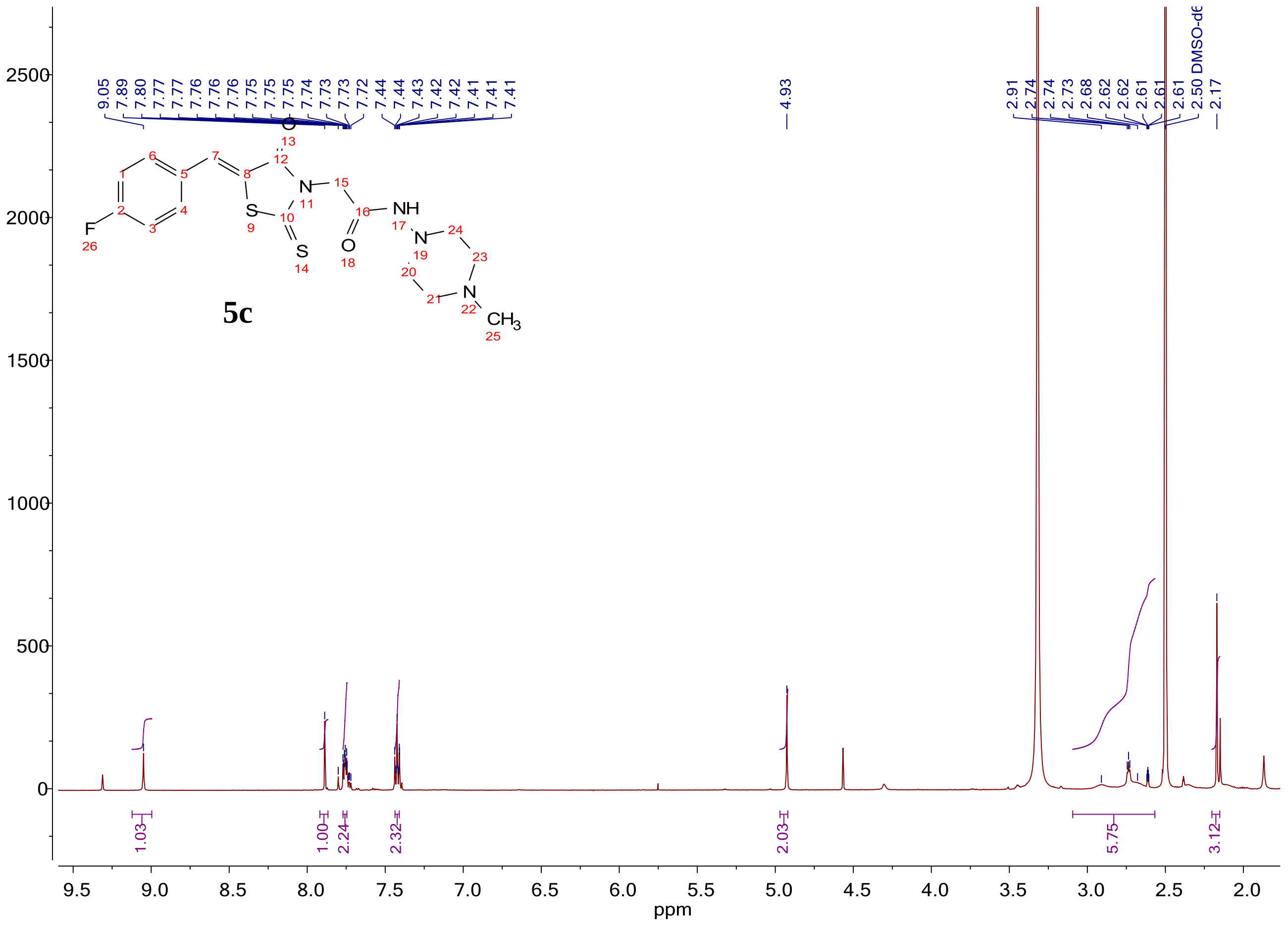
5c
ppm

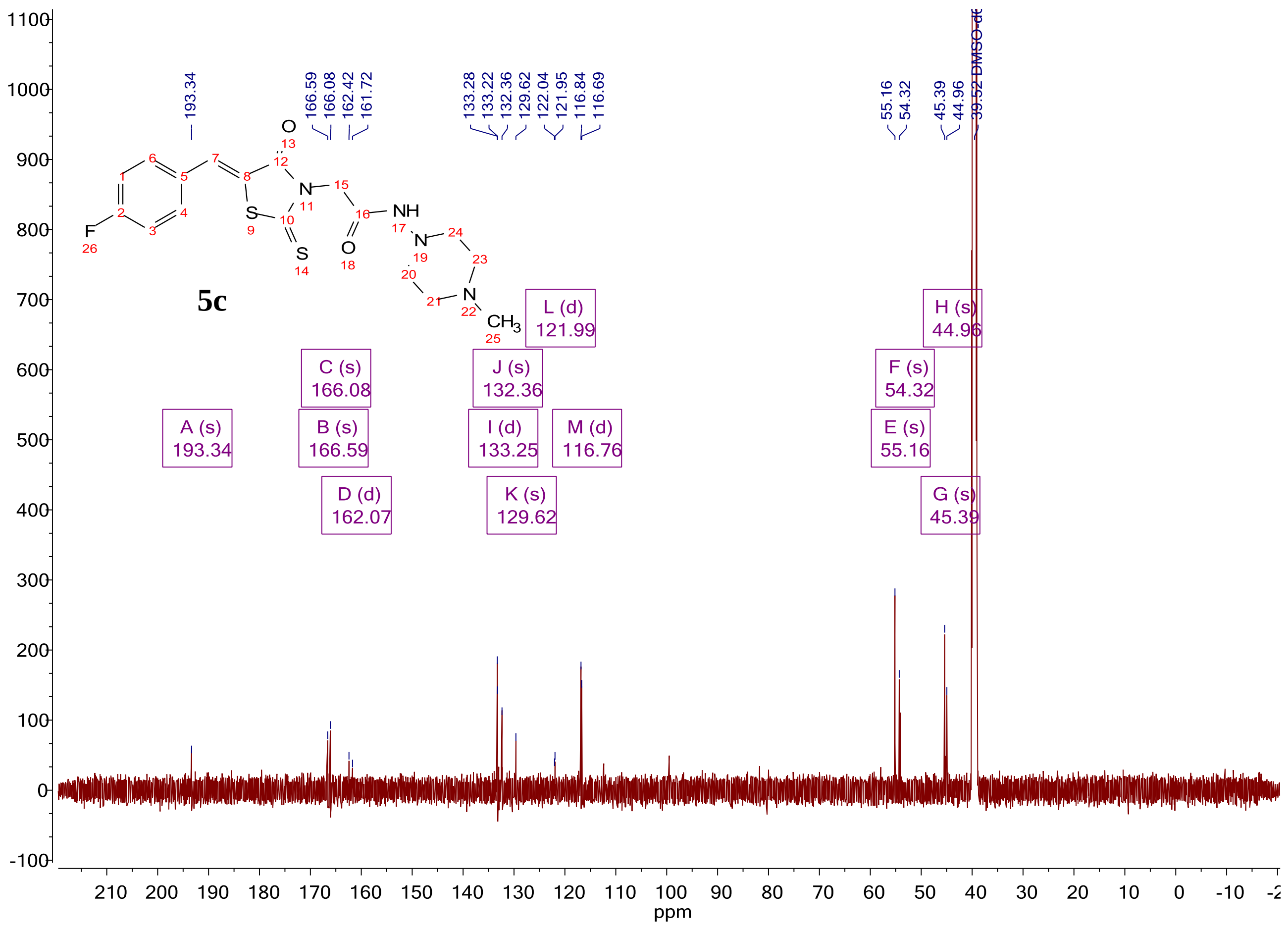
5c
A (s) 193.34
B (s) 166.59
C (s) 166.08
D (d) 162.07
E (s) 55.16
F (s) 54.32
G (s) 45.39
H (s) 44.96
I (d) 133.25
J (s) 132.36
K (s) 129.62
L (d) 121.99
M (d) 116.76
ppm

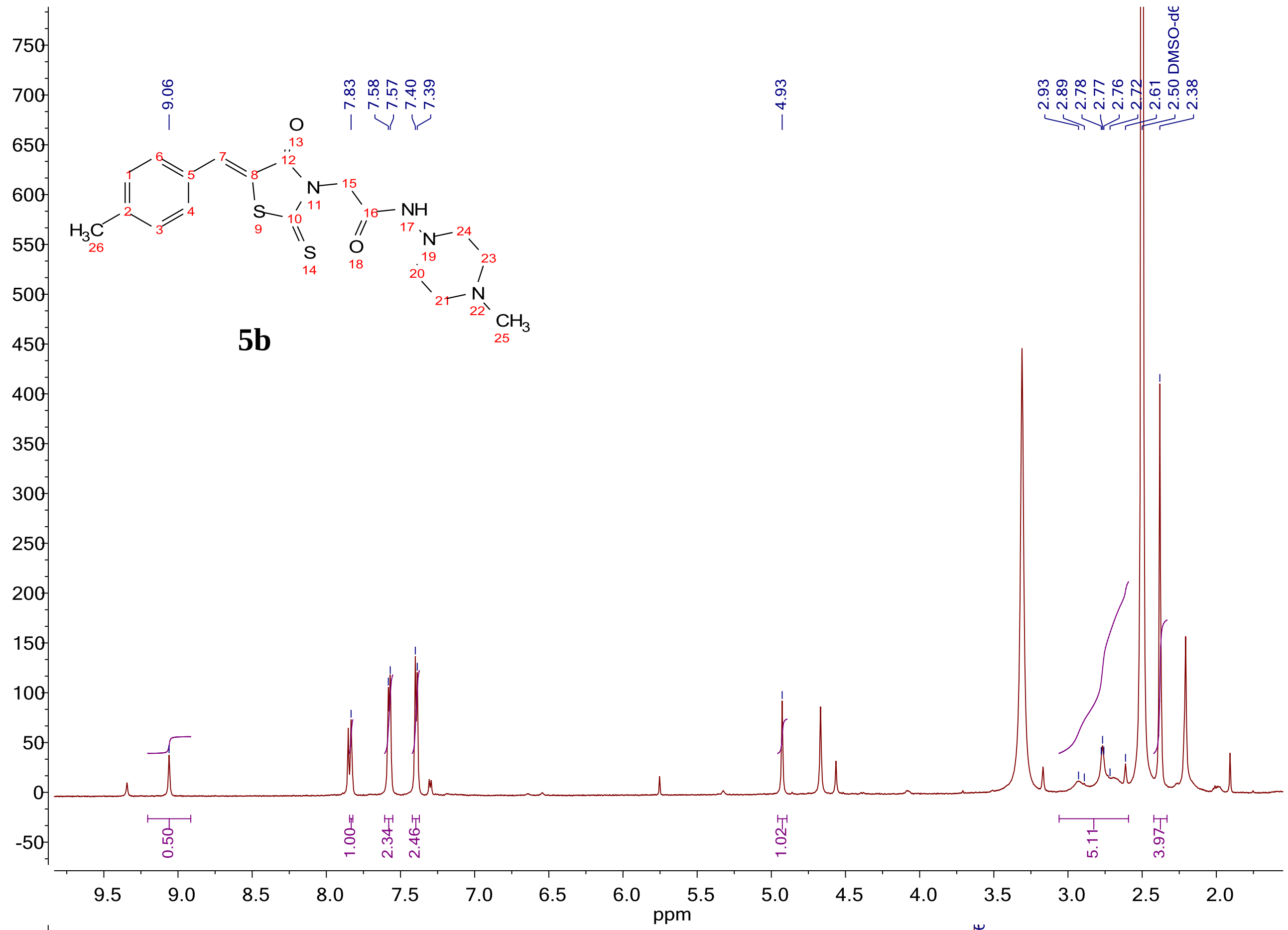
9.06
7.83
7.58
7.57
7.40
7.39
4.93
2.93
2.89
2.78
2.77
2.76
2.72
2.61
2.50 DMSO-d6
2.38
5b
0.50
1.00
2.34
2.46
1.02
5.11
3.97
ppm

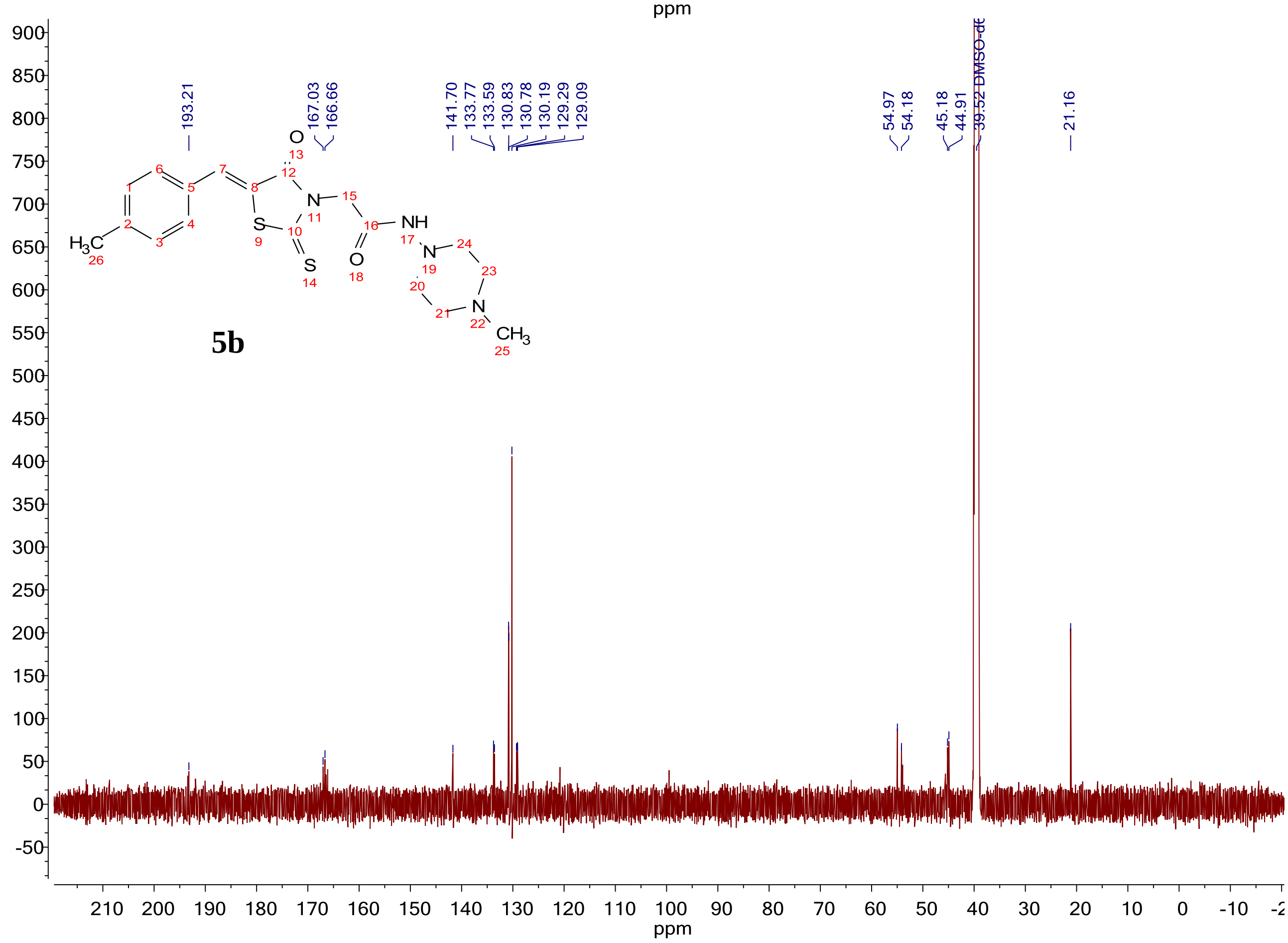
193.21
167.03
166.66
141.70
133.77
133.59
130.83
130.78
130.19
129.29
129.09
54.97
54.18
45.18
44.91
39.52 DMSO-d6
21.16
5b
ppm

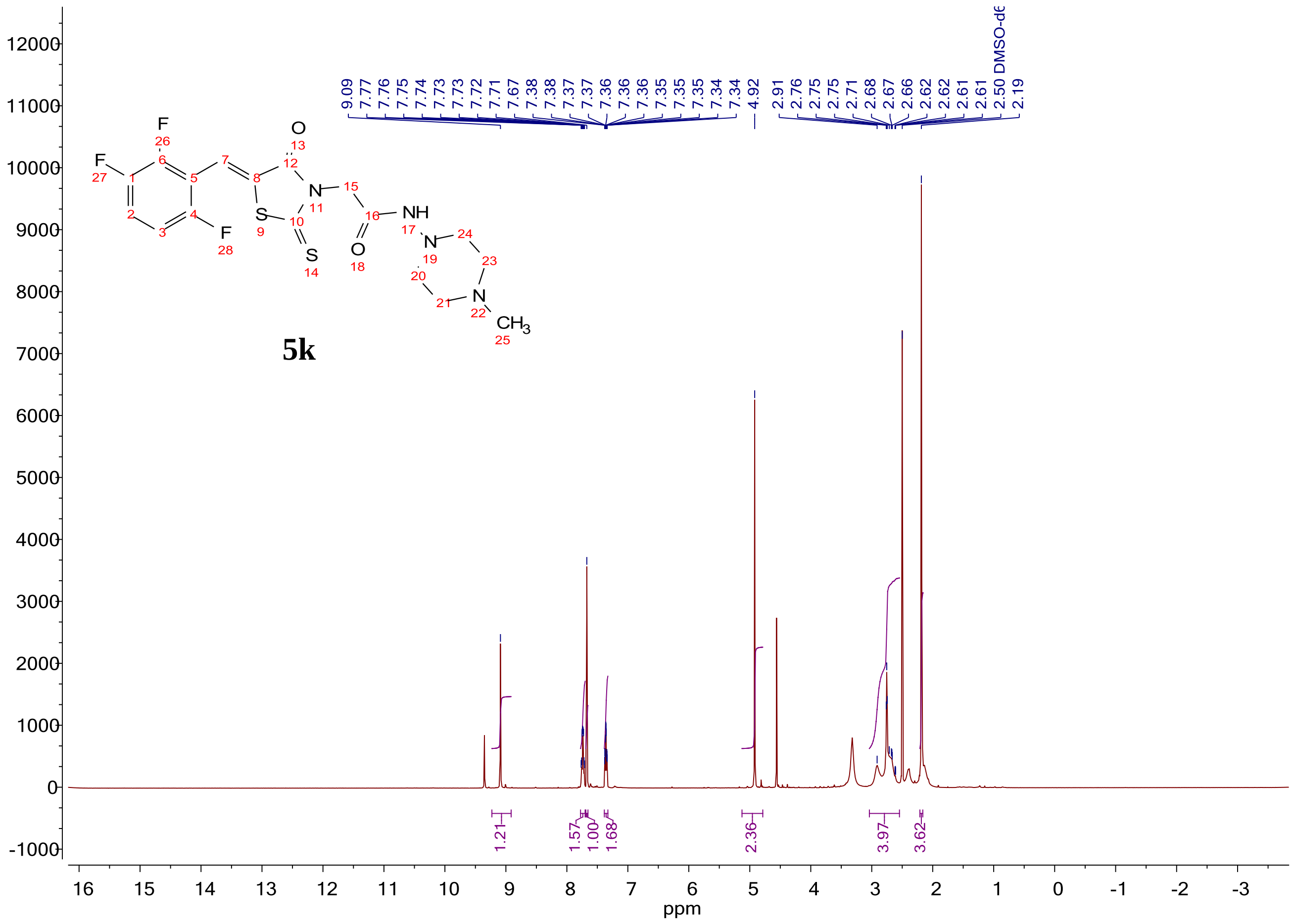

5k

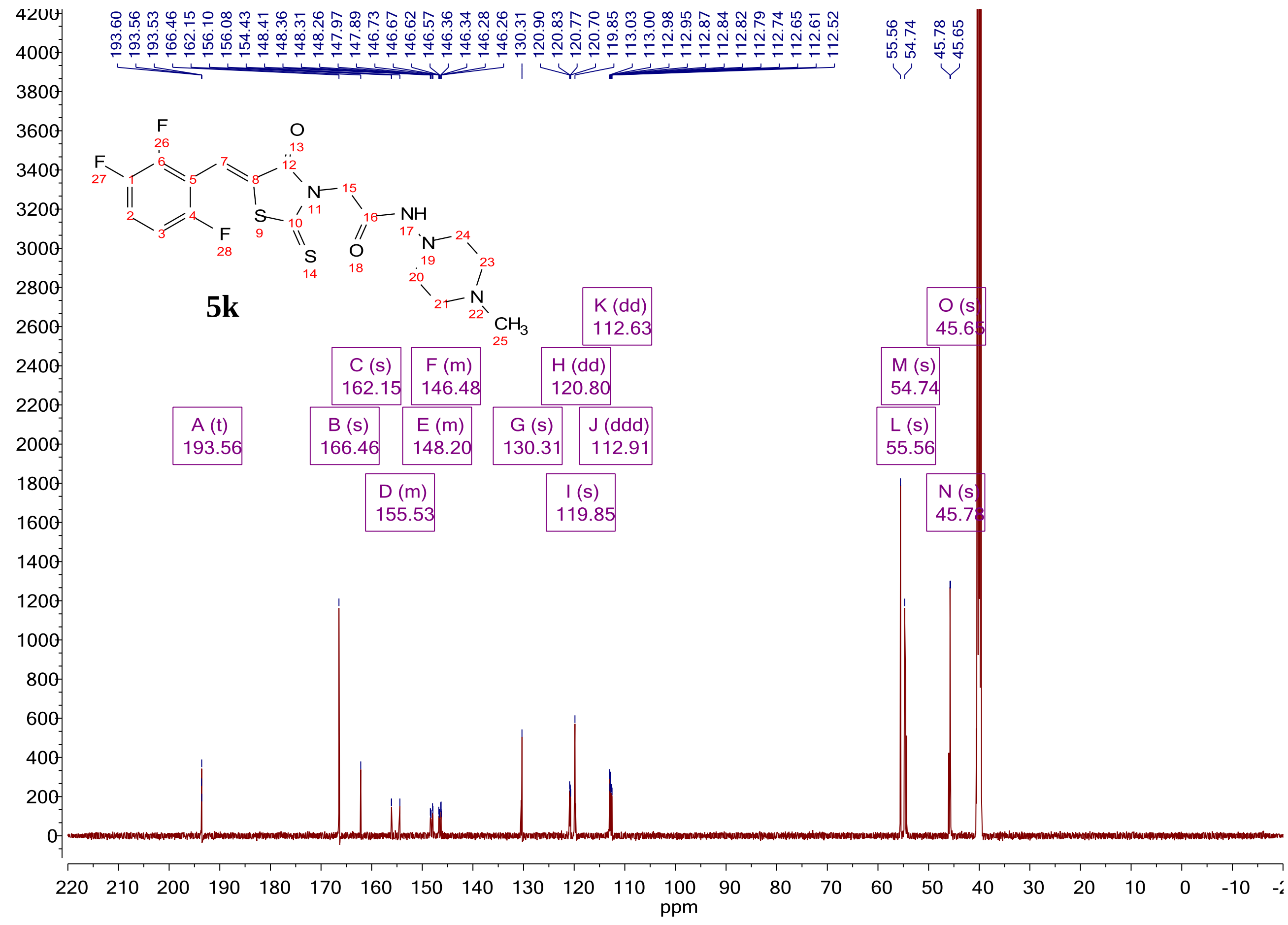

5k

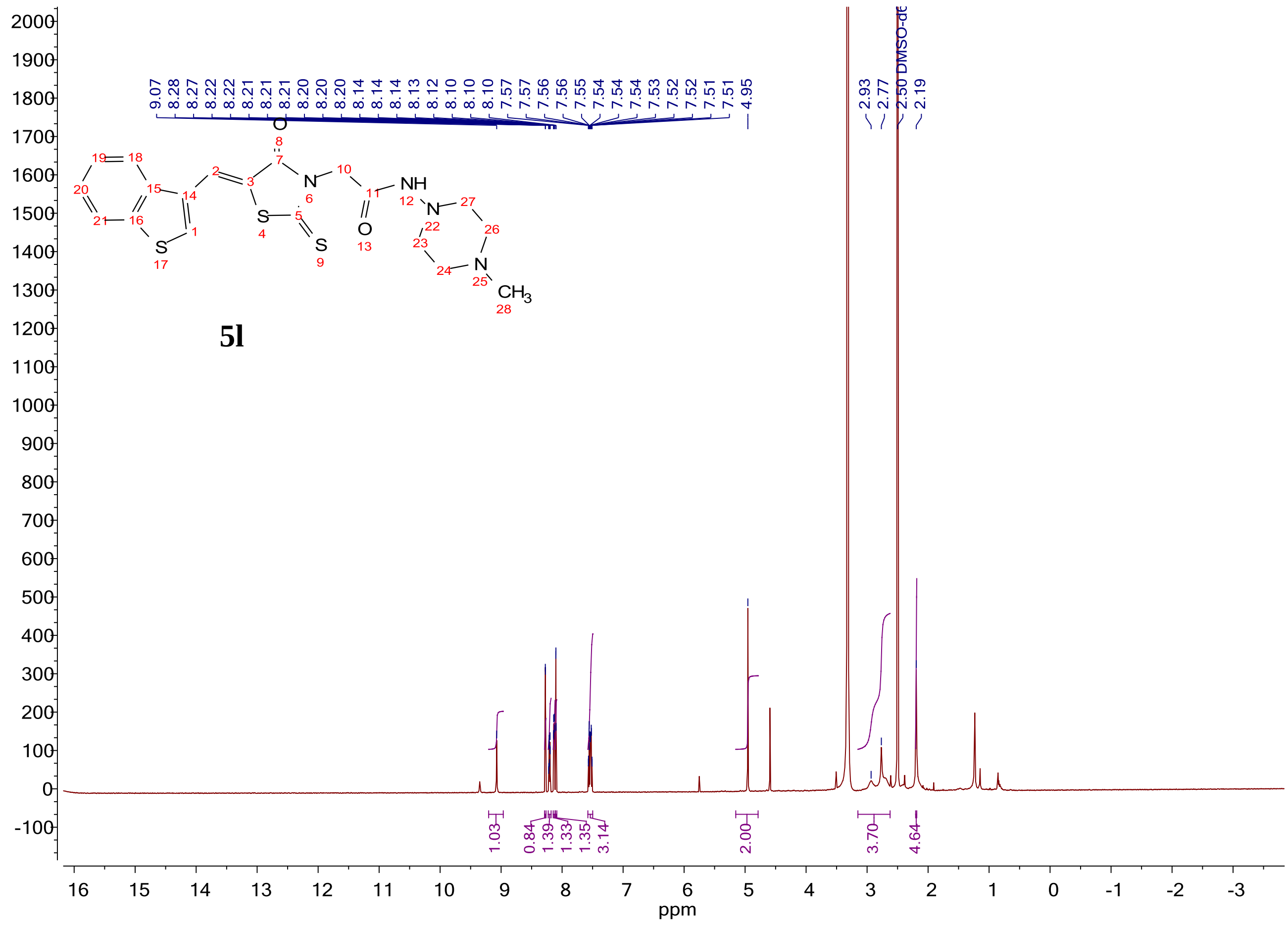

5l

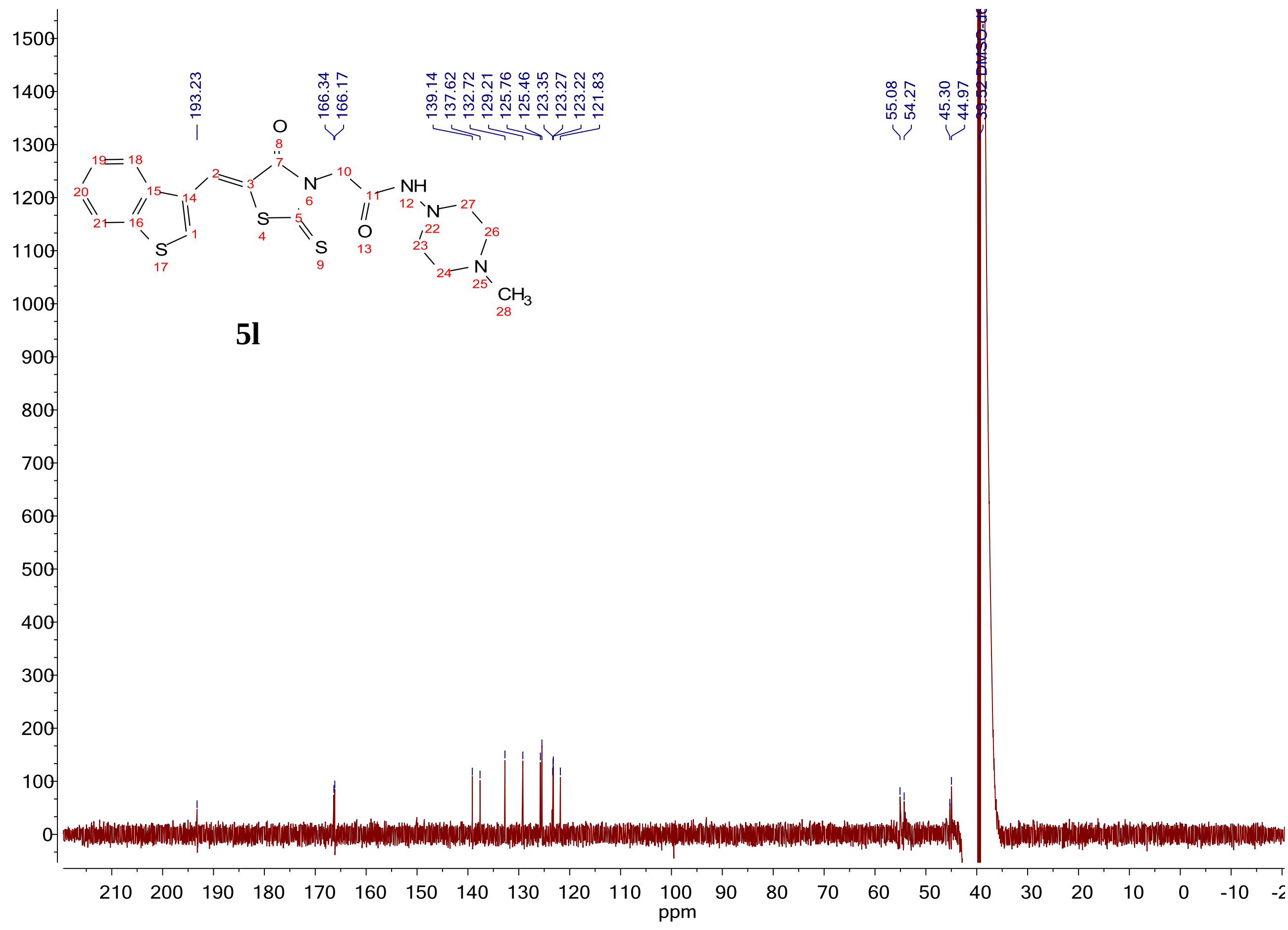

5l

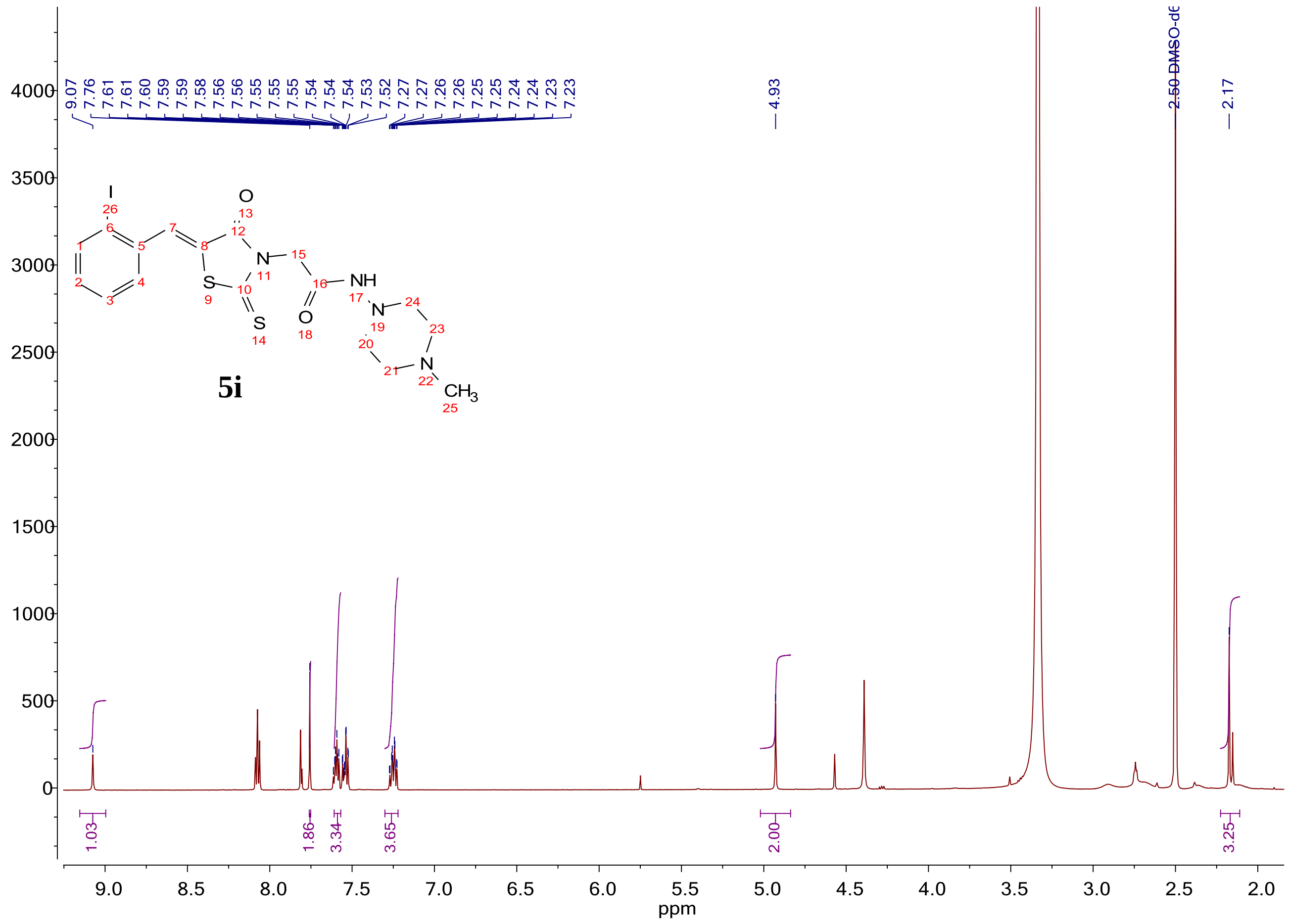
9.07
7.76
7.61
7.61
7.60
7.59
7.59
7.58
7.56
7.56
7.55
7.55
7.55
7.54
7.54
7.53
7.52
7.27
7.27
7.26
7.26
7.25
7.25
7.24
7.24
7.23
7.23
4.93
2.50 DMSO-d6
2.17
5i
1.03
1.86
3.34
3.65
2.00
3.25
ppm

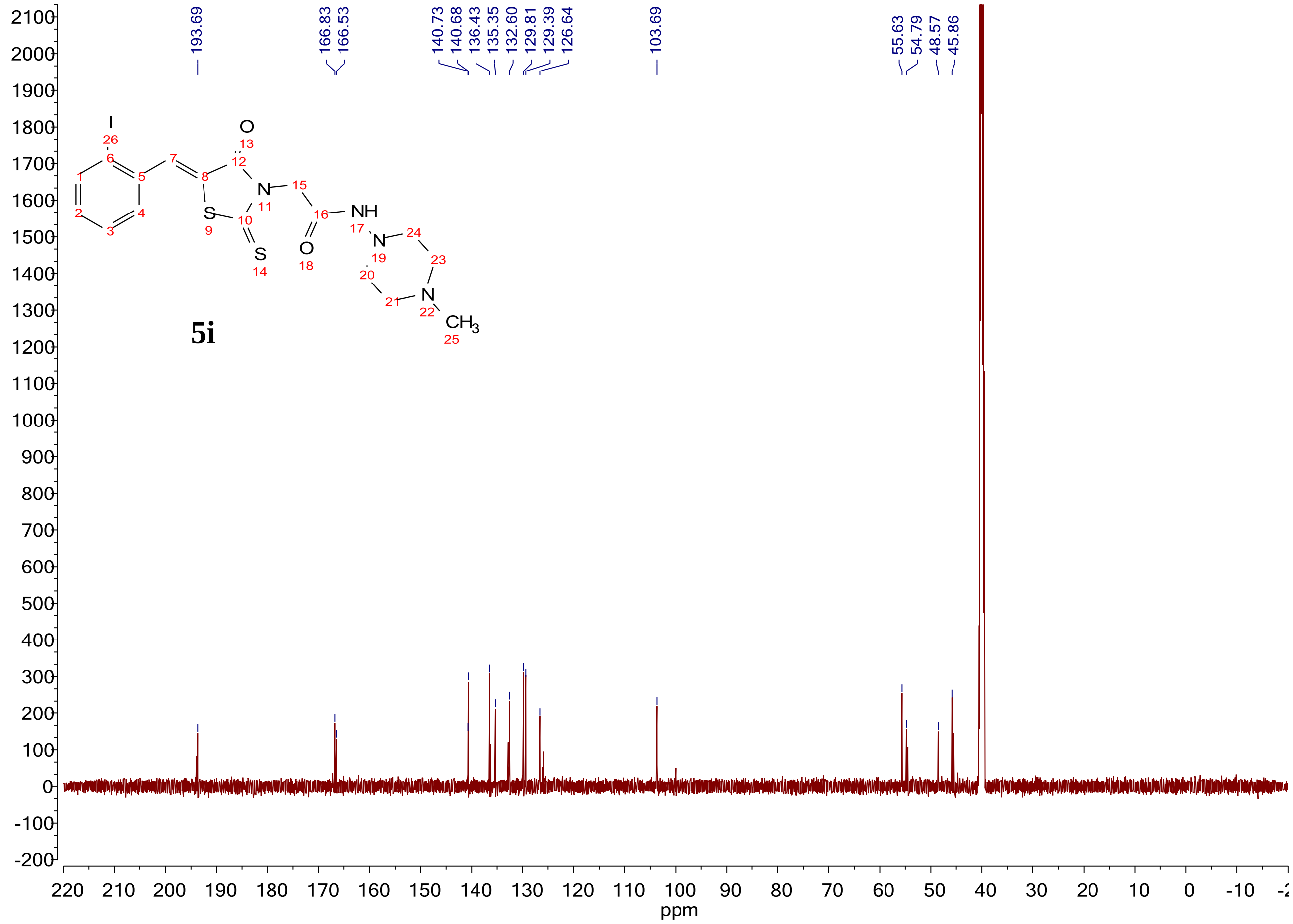
193.69
166.83
166.53
140.73
140.68
136.43
135.35
132.60
129.81
129.39
126.64
103.69
55.63
54.79
48.57
45.86
5i
ppm

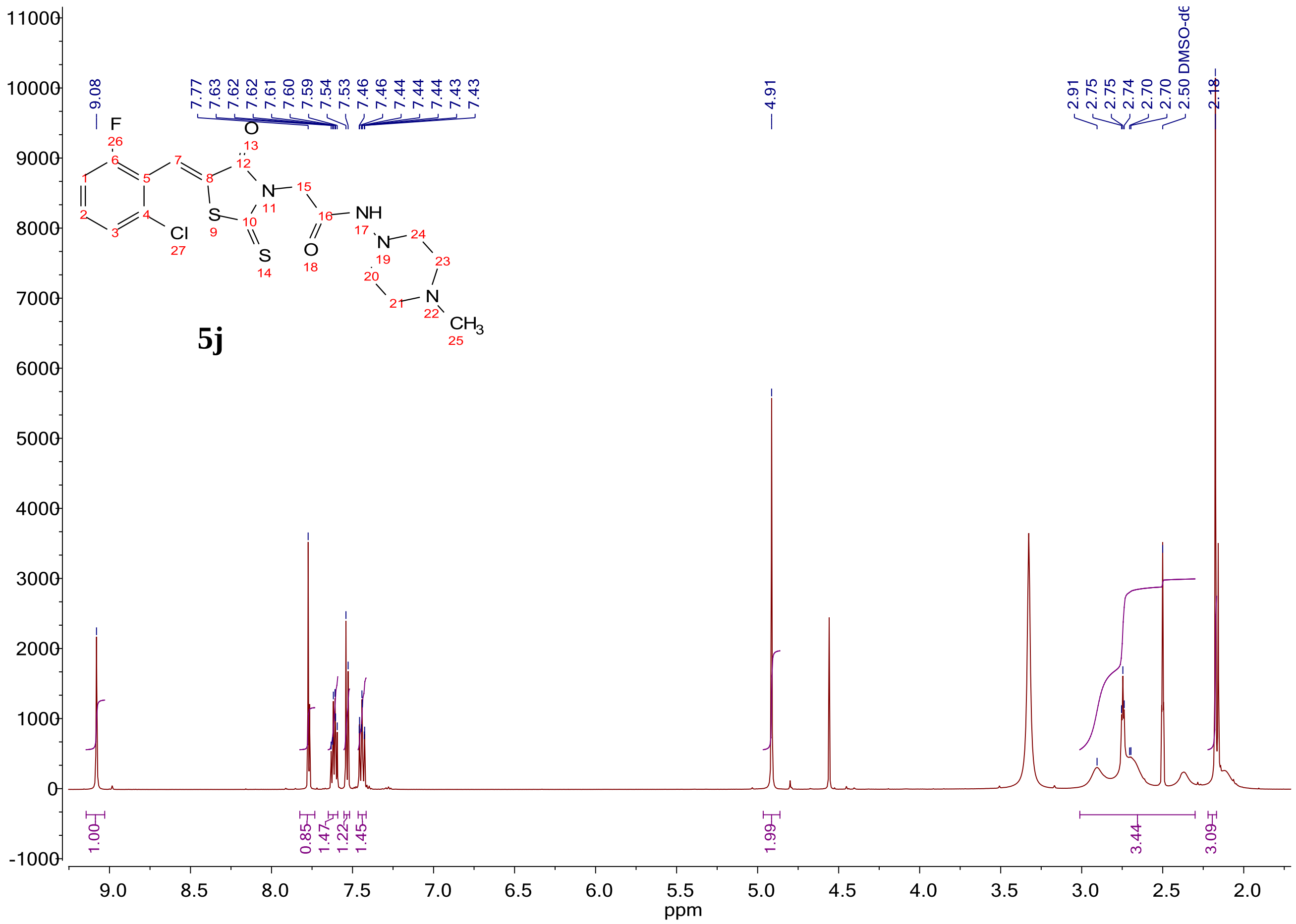
5j
9.08
7.77 7.63 7.62 7.62 7.61 7.60 7.59 7.54 7.53 7.46 7.46 7.44 7.44 7.44 7.43 7.43
4.91
2.91 2.75 2.75 2.74 2.70 2.70 2.50 DMSO-d6 2.18
1.00 0.85 1.47 1.22 1.45 1.99 3.44 3.09
ppm

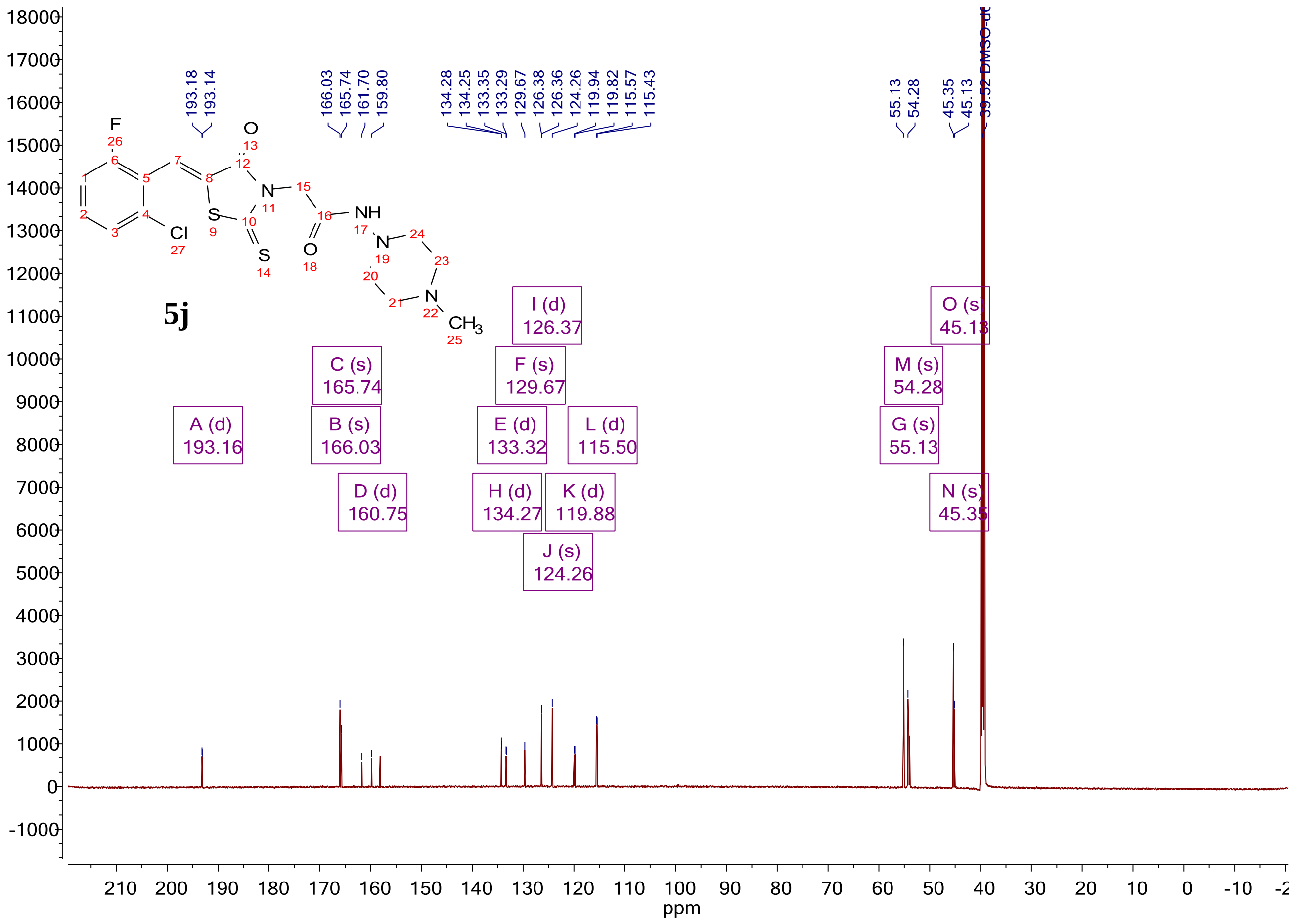
5j
193.18 193.14
166.03 165.74 161.70 159.80
134.28 134.25 133.35 133.29 129.67 126.38 126.36 124.26 119.94 119.82 115.57 115.43
55.13 54.28 45.35 45.13 39.52 DMSO-d6
A (d) 193.16
B (s) 166.03
C (s) 165.74
D (d) 160.75
E (d) 133.32
F (s) 129.67
G (s) 55.13
H (d) 134.27
I (d) 126.37
J (s) 124.26
K (d) 119.88
L (d) 115.50
M (s) 54.28
N (s) 45.35
O (s) 45.13
ppm

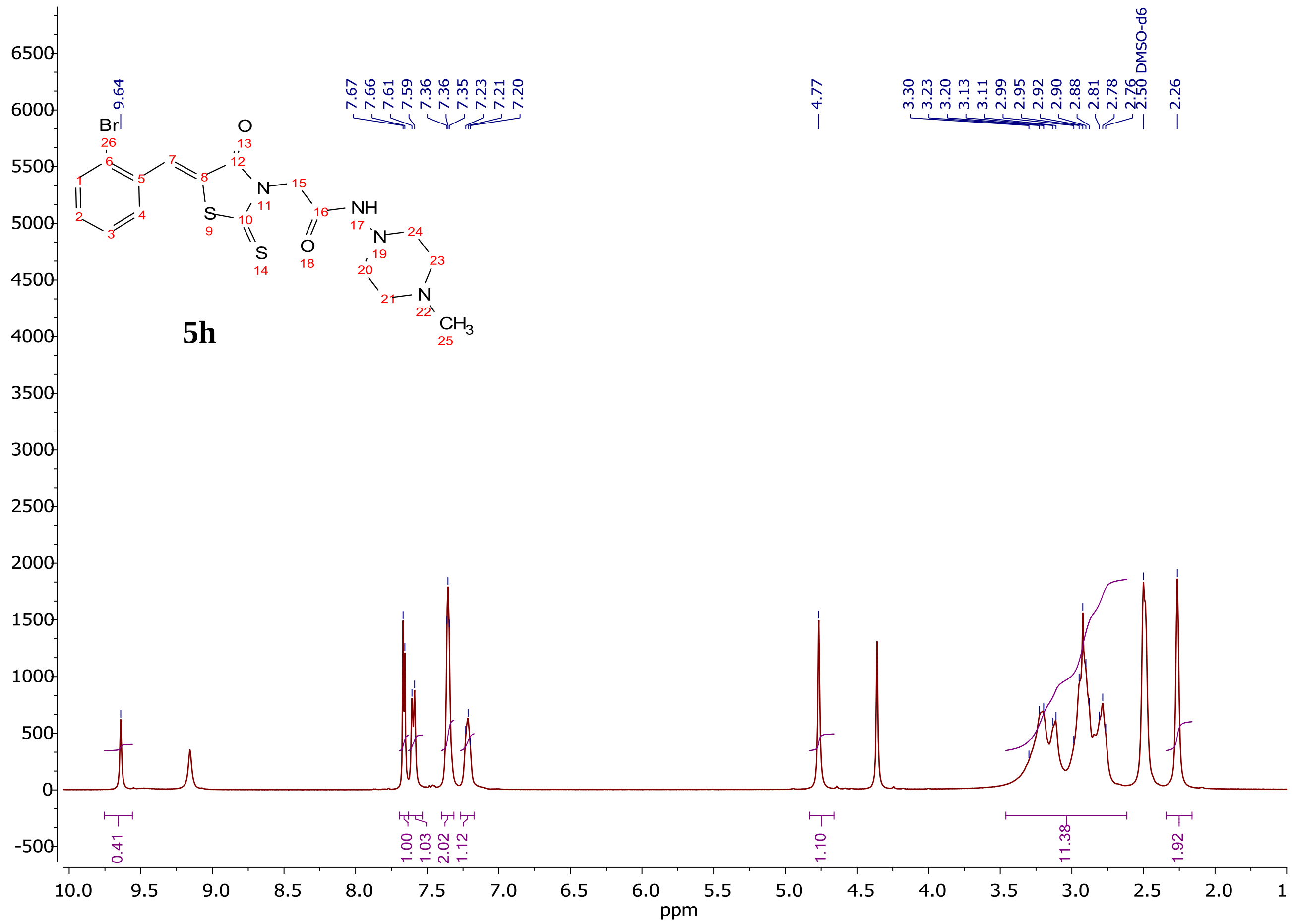
9.64
7.67 7.66 7.61 7.59 7.36 7.36 7.35 7.23 7.21 7.20
4.77
3.30 3.23 3.20 3.13 3.11 2.99 2.95 2.92 2.90 2.88 2.81 2.78 2.76 2.50 DMSO-d6
2.26
0.41 1.00 1.03 2.02 1.12 1.10 11.38 1.92
5h
ppm

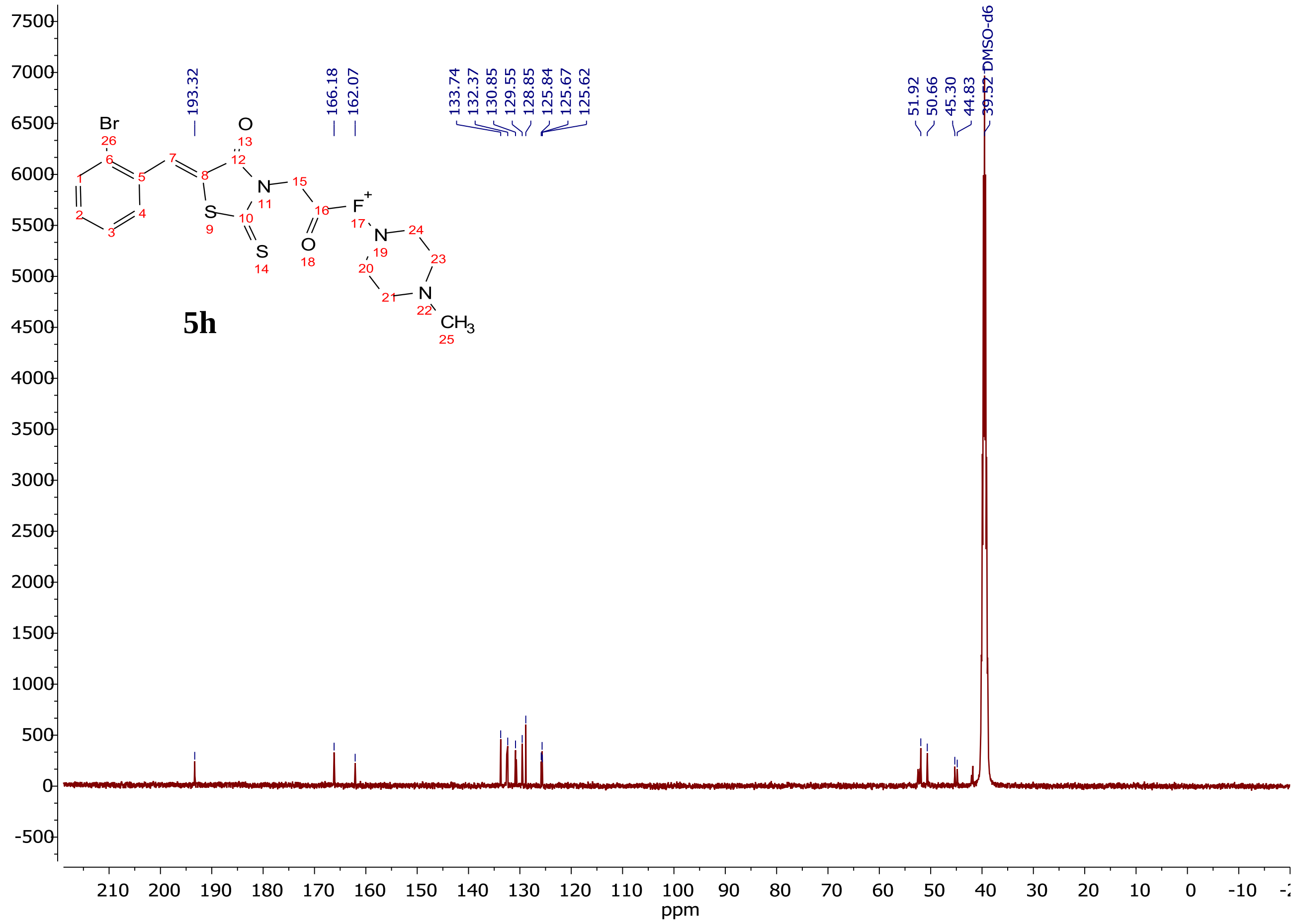
193.32
166.18 162.07
133.74 132.37 130.85 129.55 128.85 125.84 125.67 125.62
51.92 50.66 45.30 44.83 39.52 DMSO-d6
5h
ppm

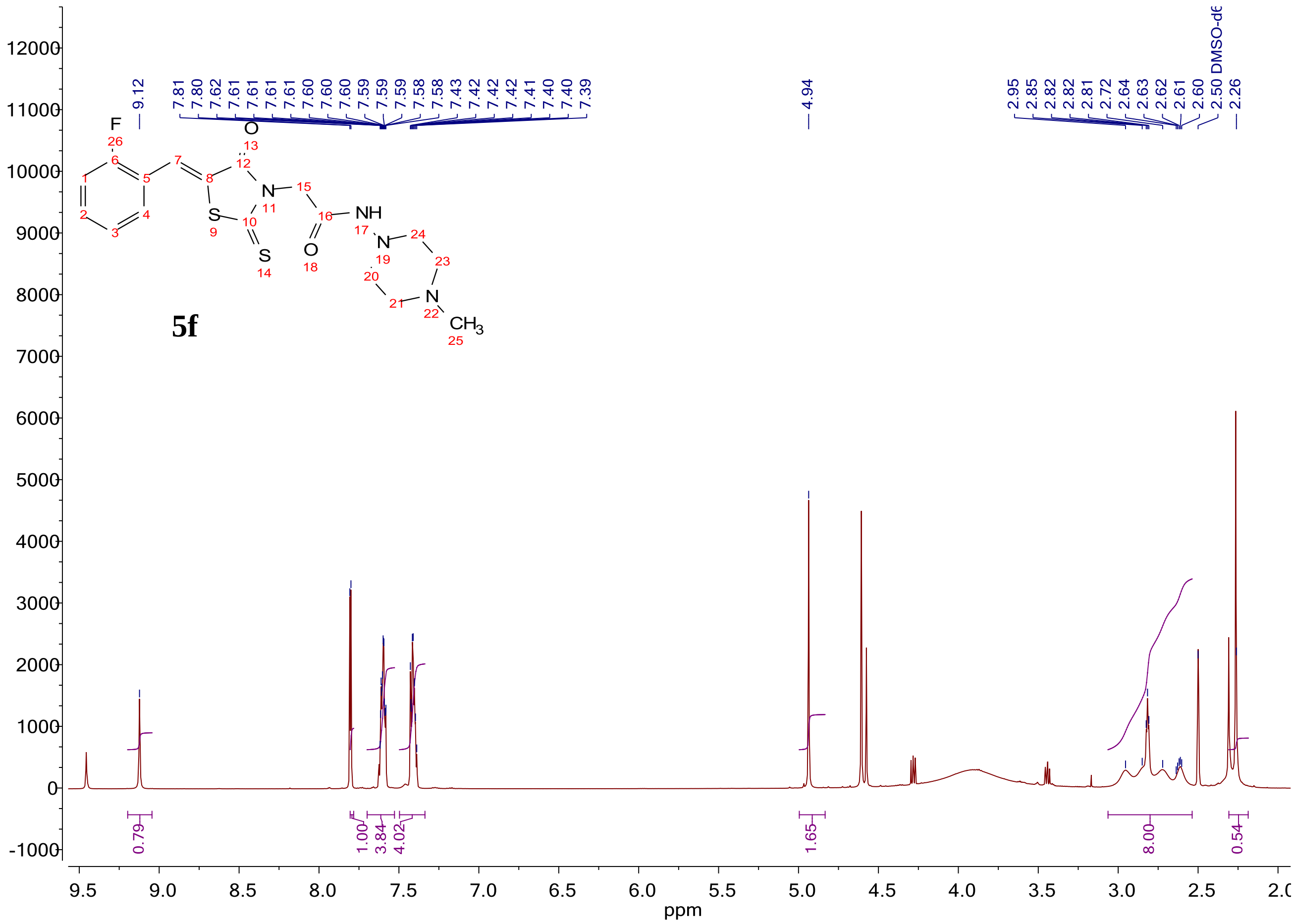
5f
9.12
7.81
7.80
7.62
7.61
7.61
7.61
7.61
7.60
7.60
7.59
7.59
7.59
7.58
7.43
7.42
7.42
7.41
7.40
7.40
7.39
4.94
2.95
2.85
2.82
2.82
2.81
2.72
2.64
2.63
2.62
2.61
2.60
2.50 DMSO-d6
2.26
0.79
1.00
3.84
4.02
1.65
8.00
0.54
ppm

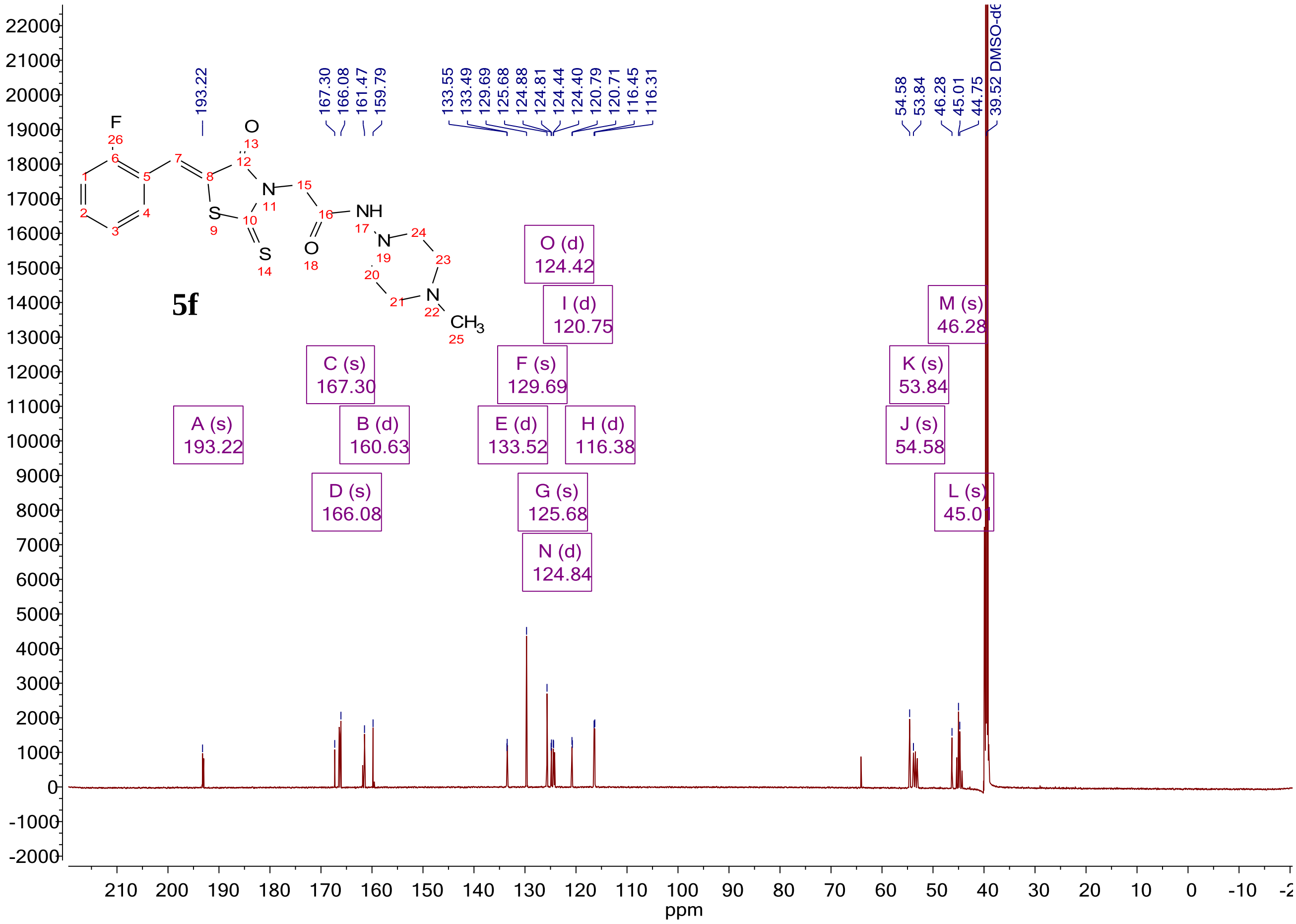
5f
193.22
167.30
166.08
161.47
159.79
133.55
133.49
129.69
125.68
124.88
124.81
124.44
124.40
120.79
120.71
116.45
116.31
54.58
53.84
46.28
45.01
44.75
39.52 DMSO-d6
A (s) 193.22
B (d) 160.63
C (s) 167.30
D (s) 166.08
E (d) 133.52
F (s) 129.69
G (s) 125.68
H (d) 116.38
I (d) 120.75
J (s) 54.58
K (s) 53.84
L (s) 45.01
M (s) 46.28
N (d) 124.84
O (d) 124.42
ppm

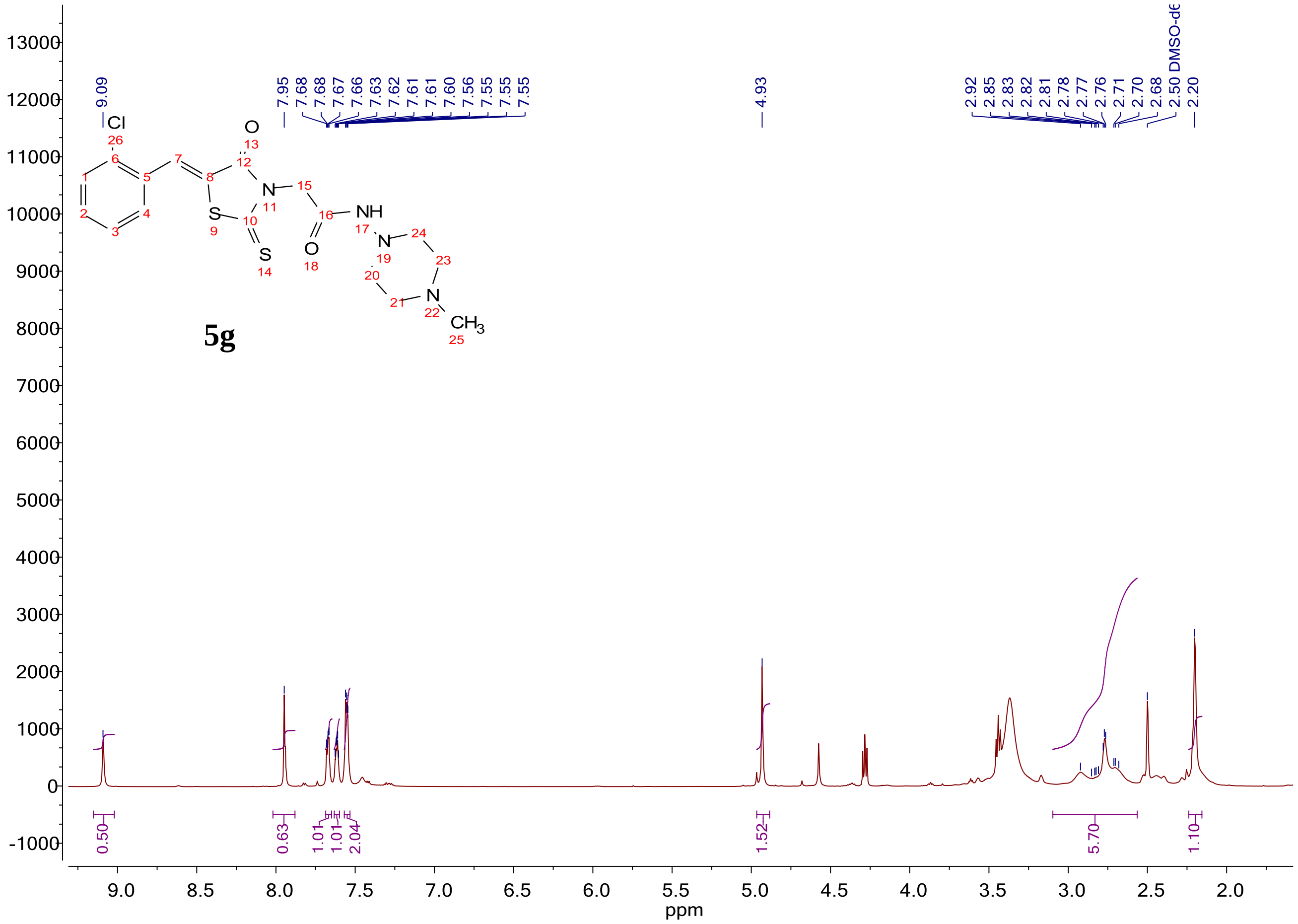
9.09
7.95
7.68
7.68
7.67
7.66
7.63
7.62
7.61
7.61
7.60
7.56
7.55
7.55
7.55
4.93
2.92
2.85
2.83
2.82
2.81
2.78
2.77
2.76
2.71
2.70
2.68
2.50 DMSO-d6
2.20
5g
0.50
0.63
1.01
1.01
2.04
1.52
5.70
1.10
ppm

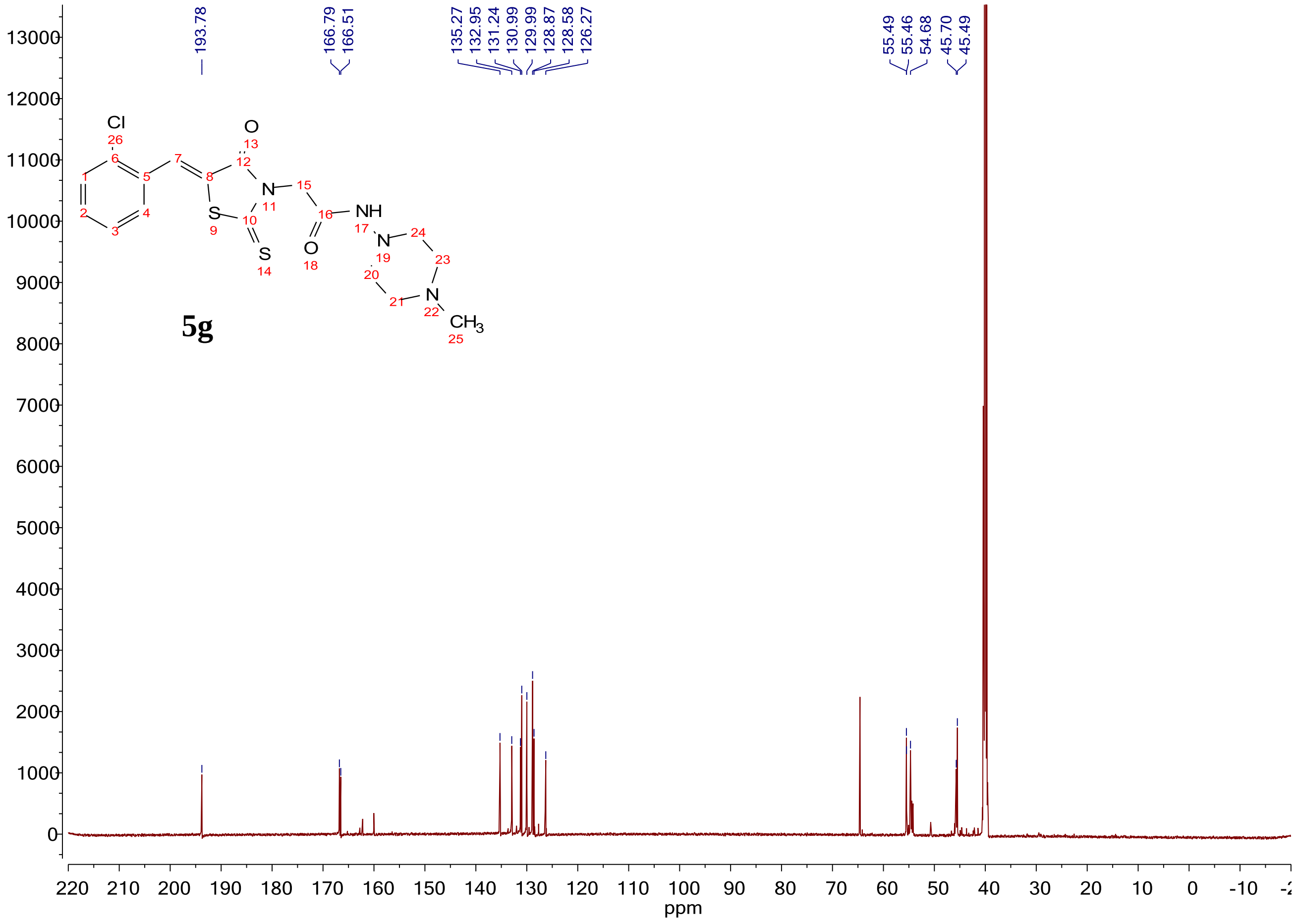
193.78
166.79
166.51
135.27
132.95
131.24
130.99
129.99
128.87
128.58
126.27
55.49
55.46
54.68
45.70
45.49
5g
ppm

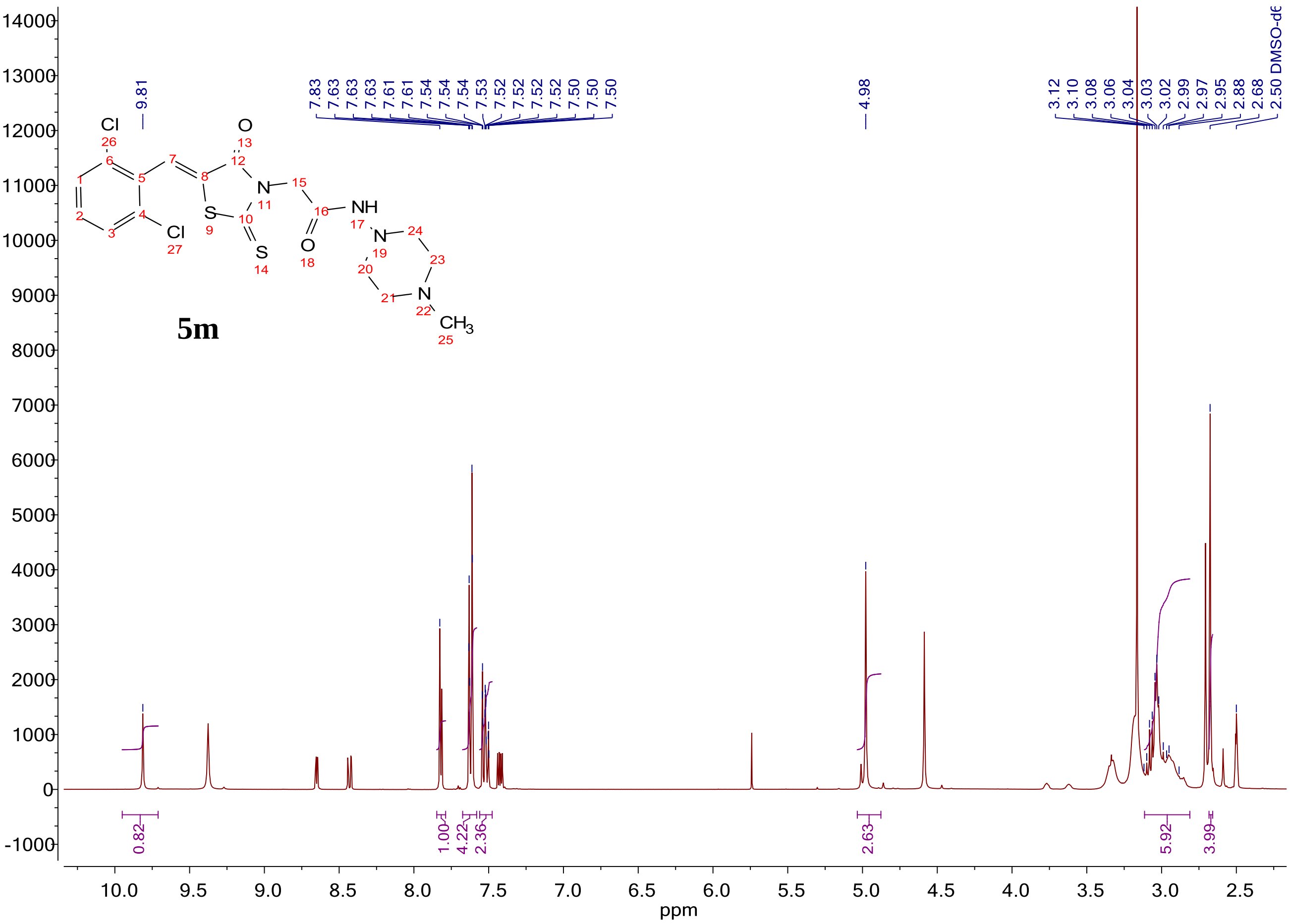
9.81
7.83
7.63
7.63
7.63
7.61
7.54
7.54
7.53
7.52
7.52
7.52
7.52
7.50
7.50
7.50
4.98
3.12
3.10
3.08
3.06
3.04
3.03
3.02
2.99
2.97
2.95
2.88
2.68
2.50 DMSO-d6
5m
0.82
1.00
4.22
2.36
2.63
5.92
3.99
ppm

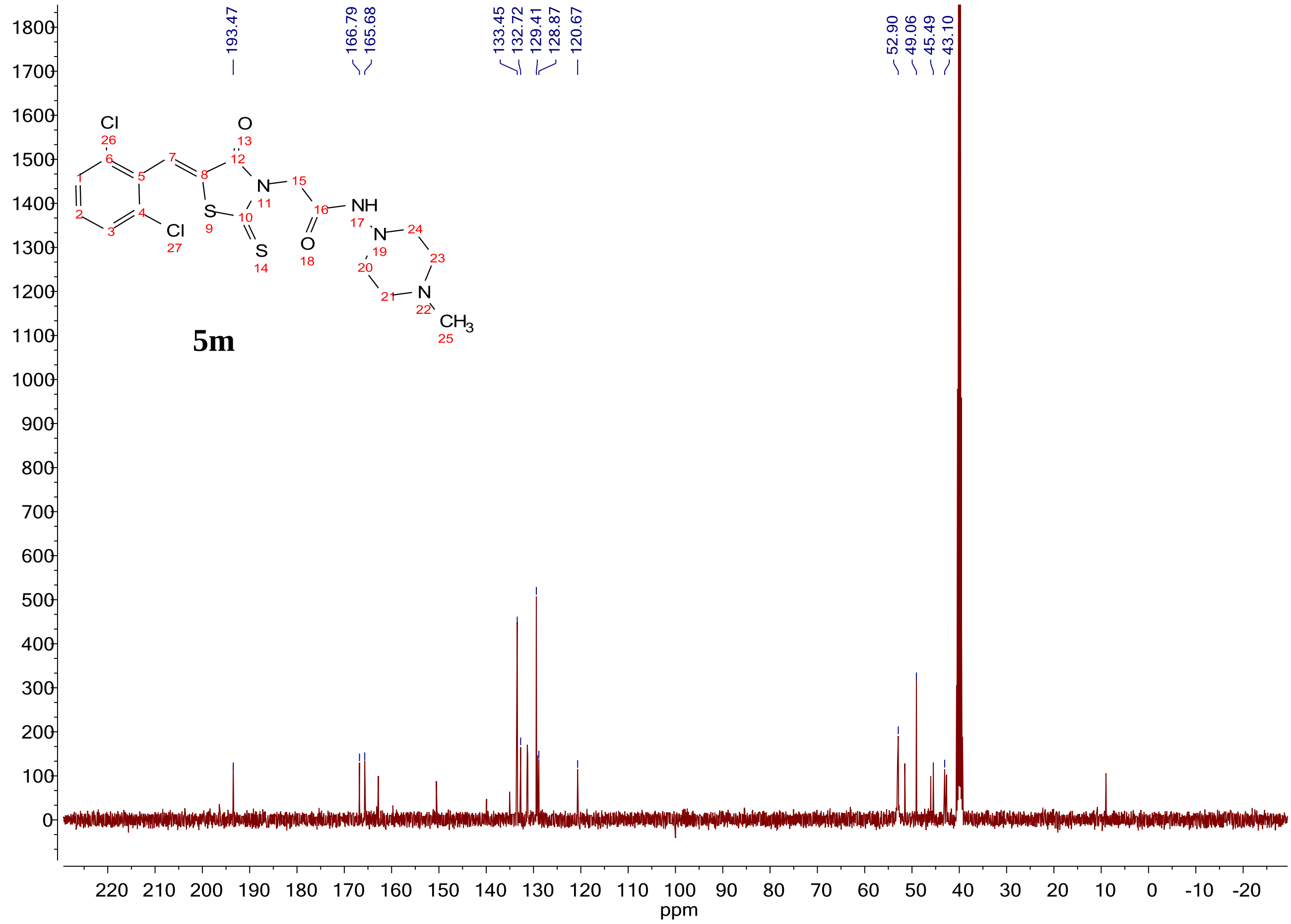
193.47
166.79
165.68
133.45
132.72
129.41
128.87
120.67
52.90
49.06
45.49
43.10
5m
ppm

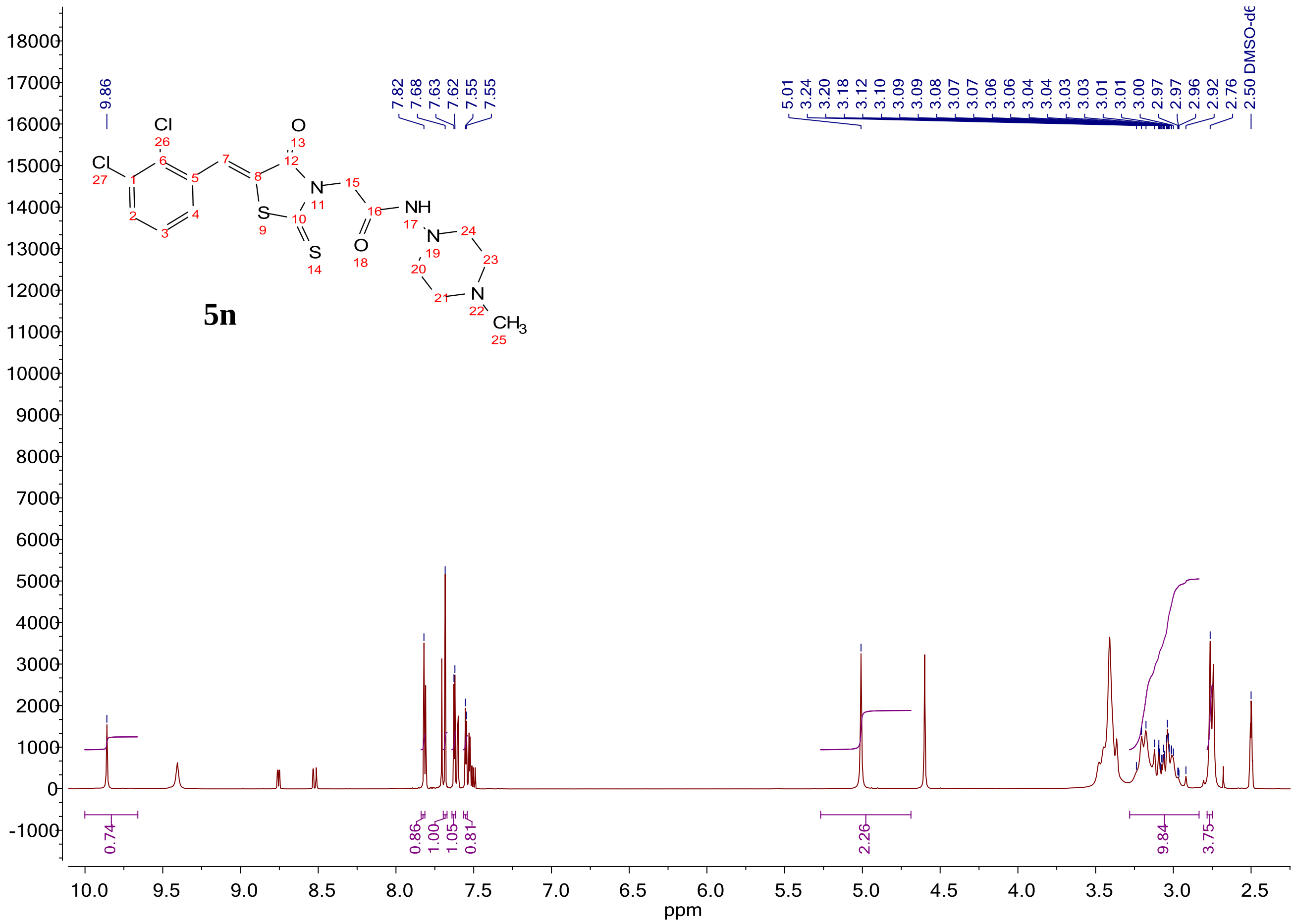
9.86
7.82
7.68
7.63
7.62
7.55
7.55
5.01
3.24
3.20
3.18
3.12
3.10
3.09
3.09
3.08
3.07
3.07
3.06
3.06
3.04
3.04
3.03
3.03
3.01
3.01
3.00
2.97
2.97
2.96
2.92
2.76
2.50 DMSO-d6
5n
0.74
0.86
1.00
1.05
0.81
2.26
9.84
3.75
ppm

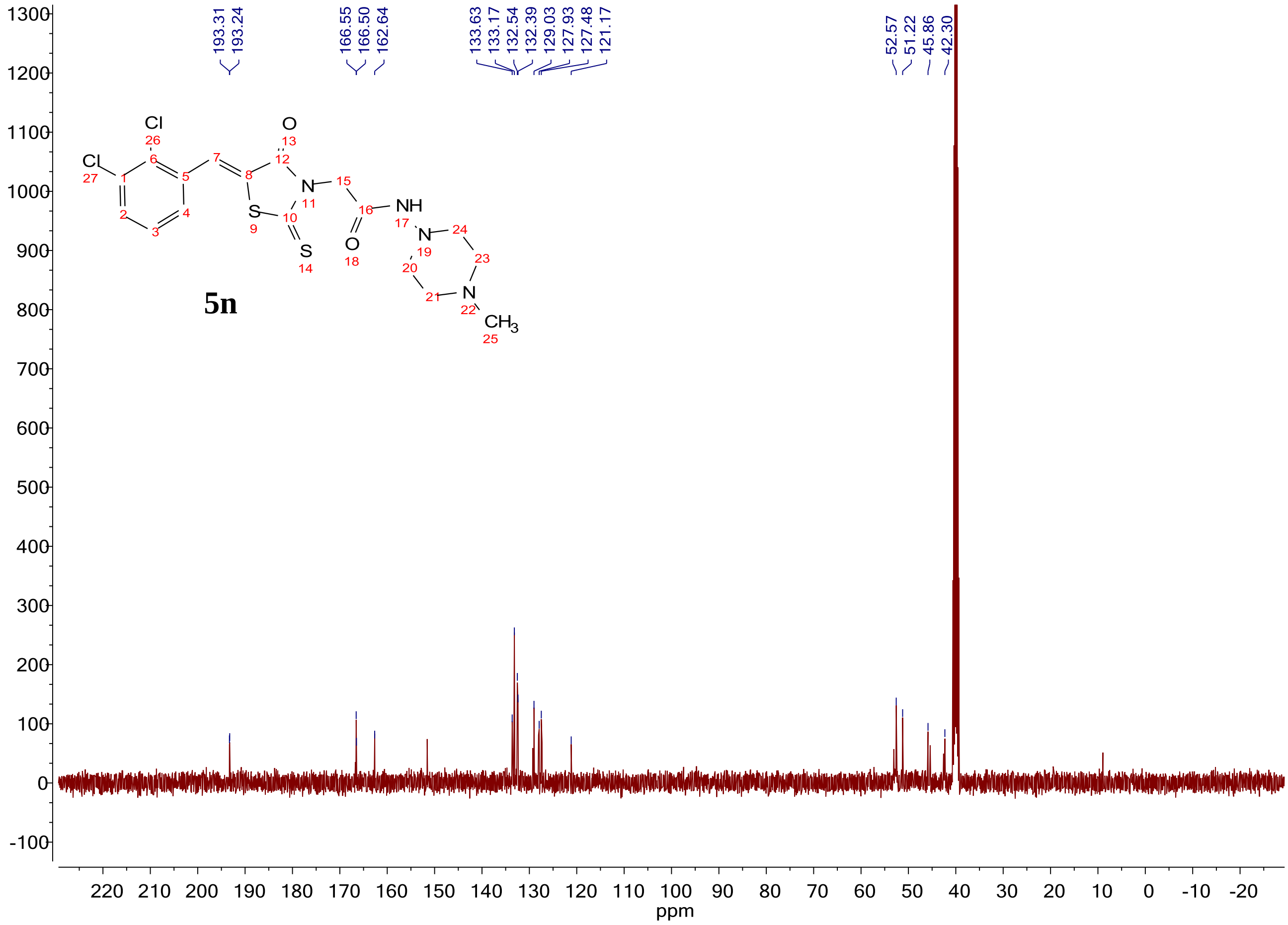
193.31
193.24
166.55
166.50
162.64
133.63
133.17
132.54
132.39
129.03
127.93
127.48
121.17
52.57
51.22
45.86
42.30
5n
ppm

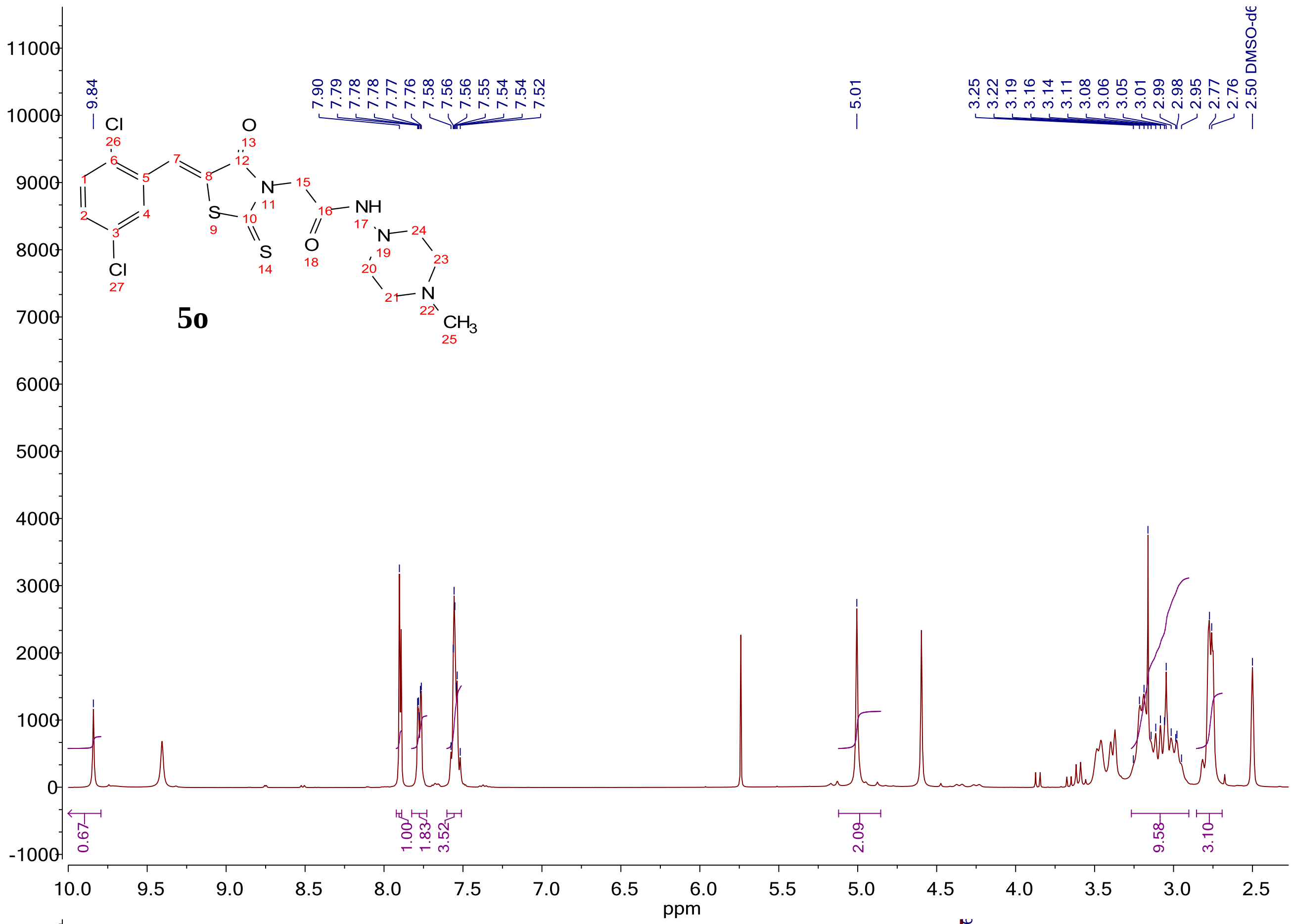
9.84
7.90 7.79 7.78 7.78 7.77 7.76 7.58 7.56 7.56 7.55 7.54 7.54 7.52
5.01
3.25 3.22 3.19 3.16 3.14 3.11 3.08 3.06 3.05 3.01 2.99 2.98 2.95 2.77 2.76
2.50 DMSO-d6
5o
0.67
1.00
1.83
3.52
2.09
9.58
3.10
ppm

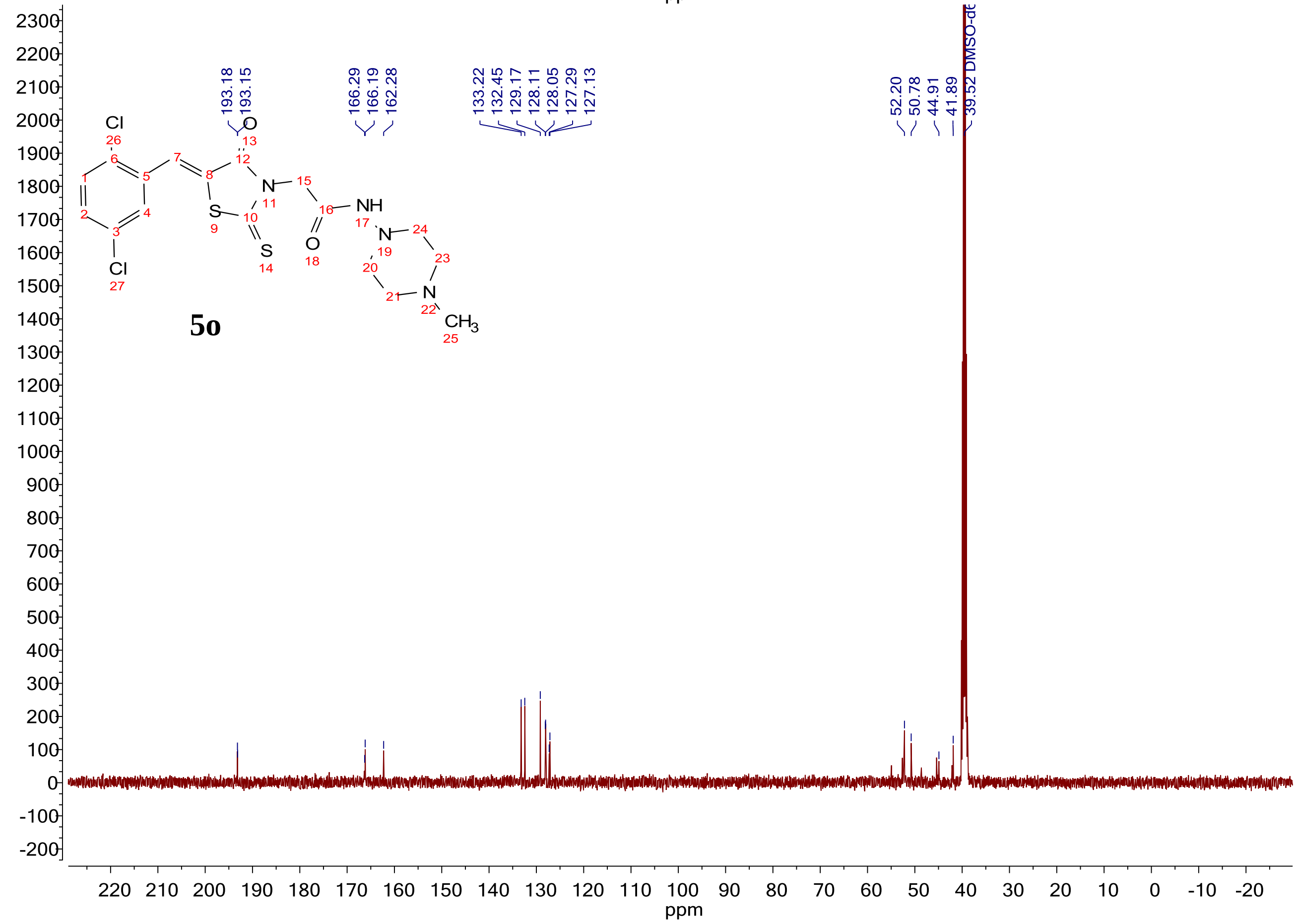
193.18 193.15
166.29 166.19 162.28
133.22 132.45 129.17 128.11 128.05 127.29 127.13
52.20 50.78 44.91 41.89
39.52 DMSO-d6
5o
ppm

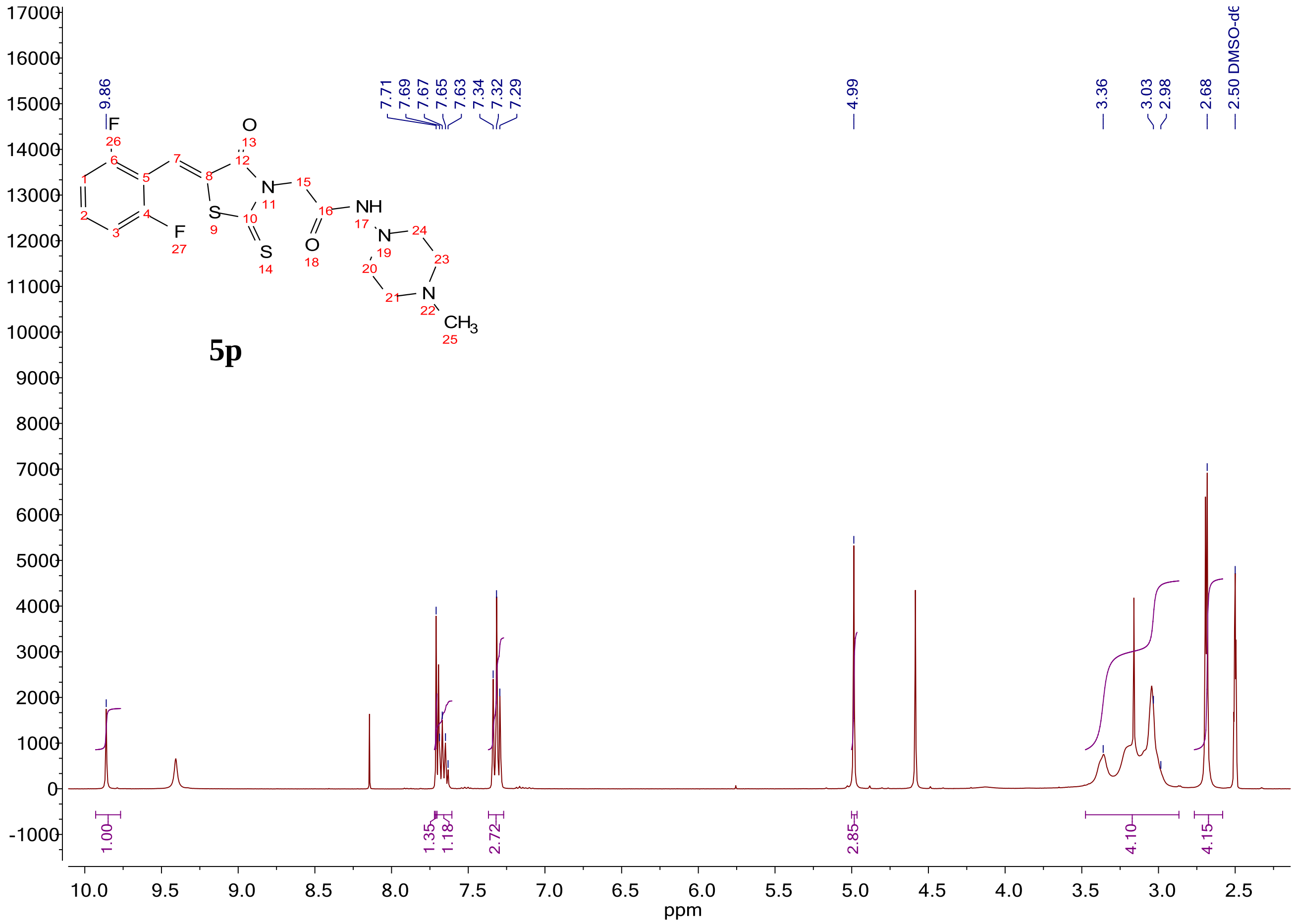

9.86
7.71
7.69
7.67
7.65
7.63
7.34
7.32
7.29
4.99
3.36
3.03
2.98
2.68
2.50 DMSO-d6
5p
1.00
1.35
1.18
2.72
2.85
4.10
4.15
ppm

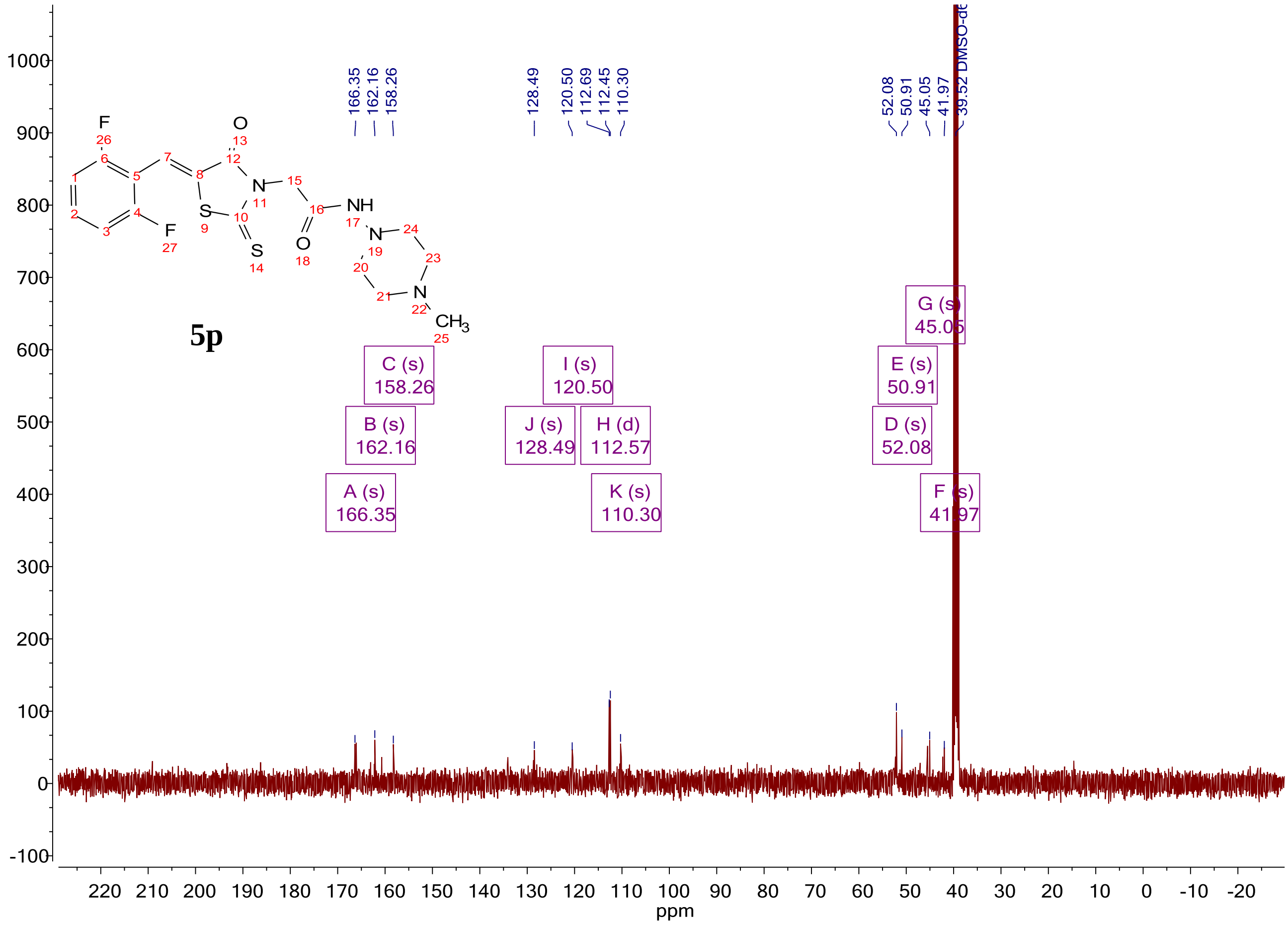

166.35
162.16
158.26
128.49
120.50
112.69
112.45
110.30
52.08
50.91
45.05
41.97
39.52 DMSO-d6
5p
A (s) 166.35
B (s) 162.16
C (s) 158.26
D (s) 52.08
E (s) 50.91
F (s) 41.97
G (s) 45.05
H (d) 112.57
I (s) 120.50
J (s) 128.49
K (s) 110.30
ppm

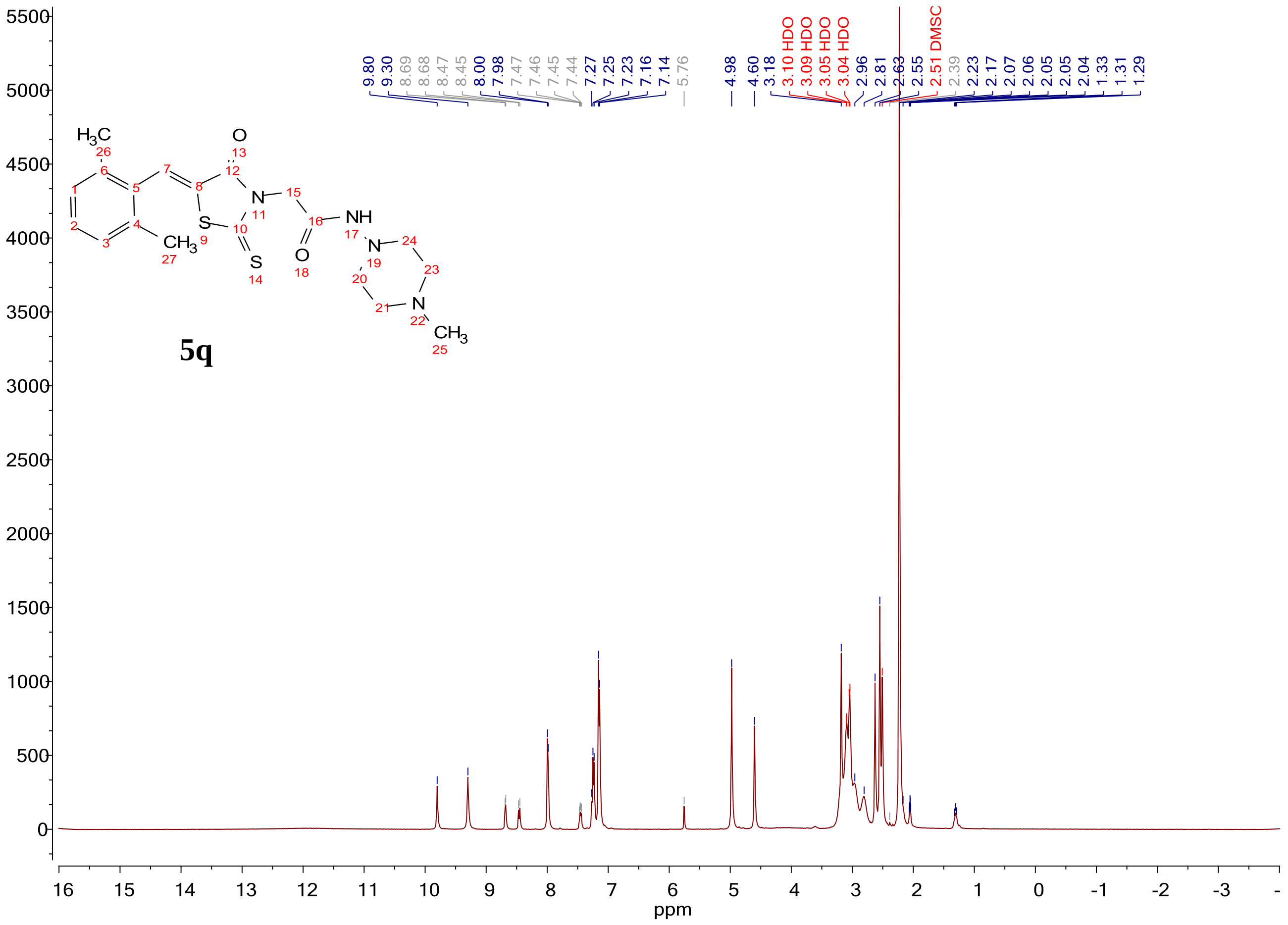
9.80
9.30
8.69
8.68
8.47
8.45
8.00
7.98
7.47
7.46
7.45
7.44
7.27
7.25
7.23
7.16
7.14
5.76
4.98
4.60
3.18
3.10 HDO
3.09 HDO
3.05 HDO
3.04 HDO
2.96
2.81
2.63
2.55
2.51 DMSO
2.39
2.23
2.17
2.07
2.06
2.05
2.05
2.04
1.33
1.31
1.29
5q
ppm

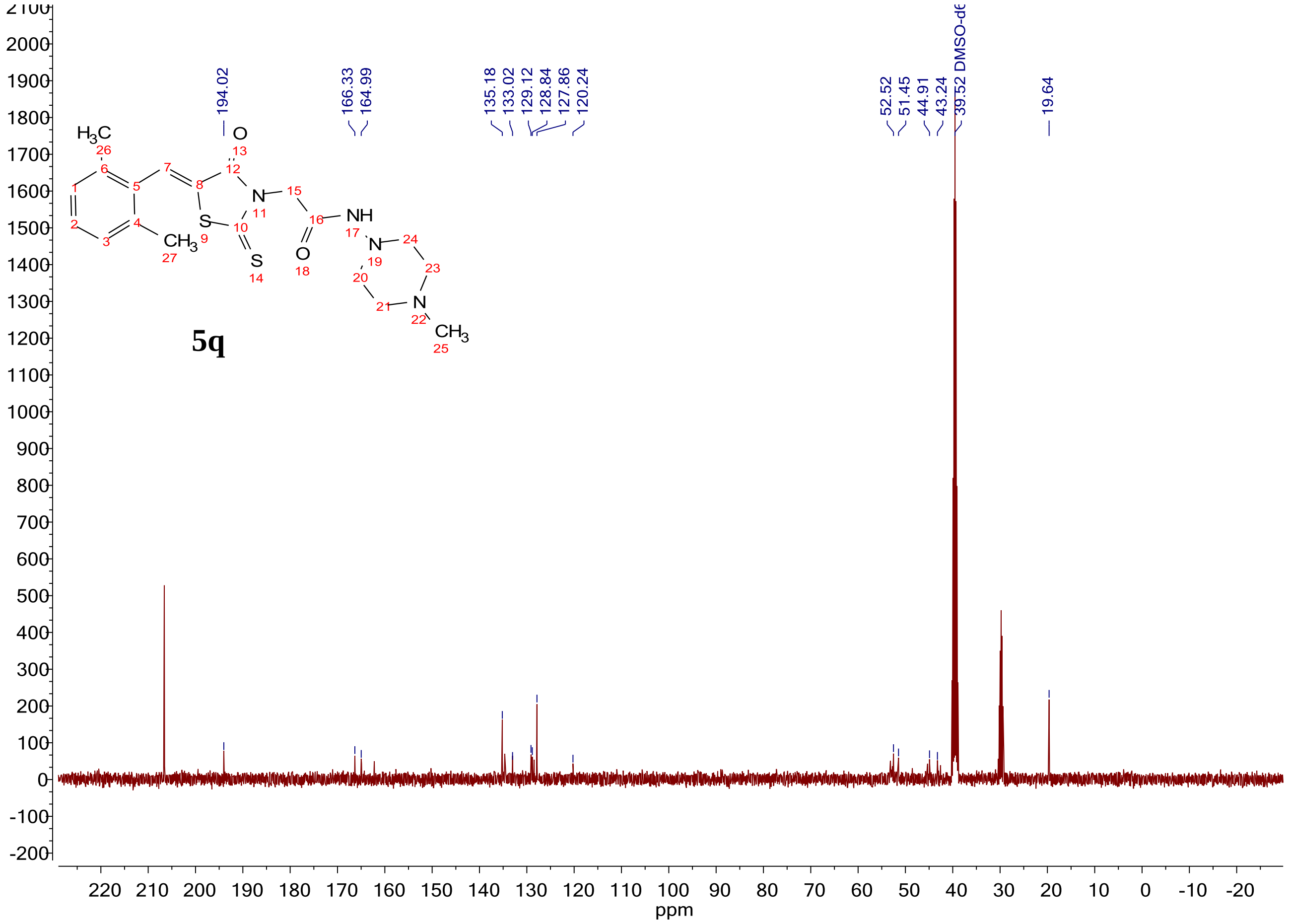
194.02
166.33
164.99
135.18
133.02
129.12
128.84
127.86
120.24
52.52
51.45
44.91
43.24
39.52 DMSO-d6
19.64
5q
ppm

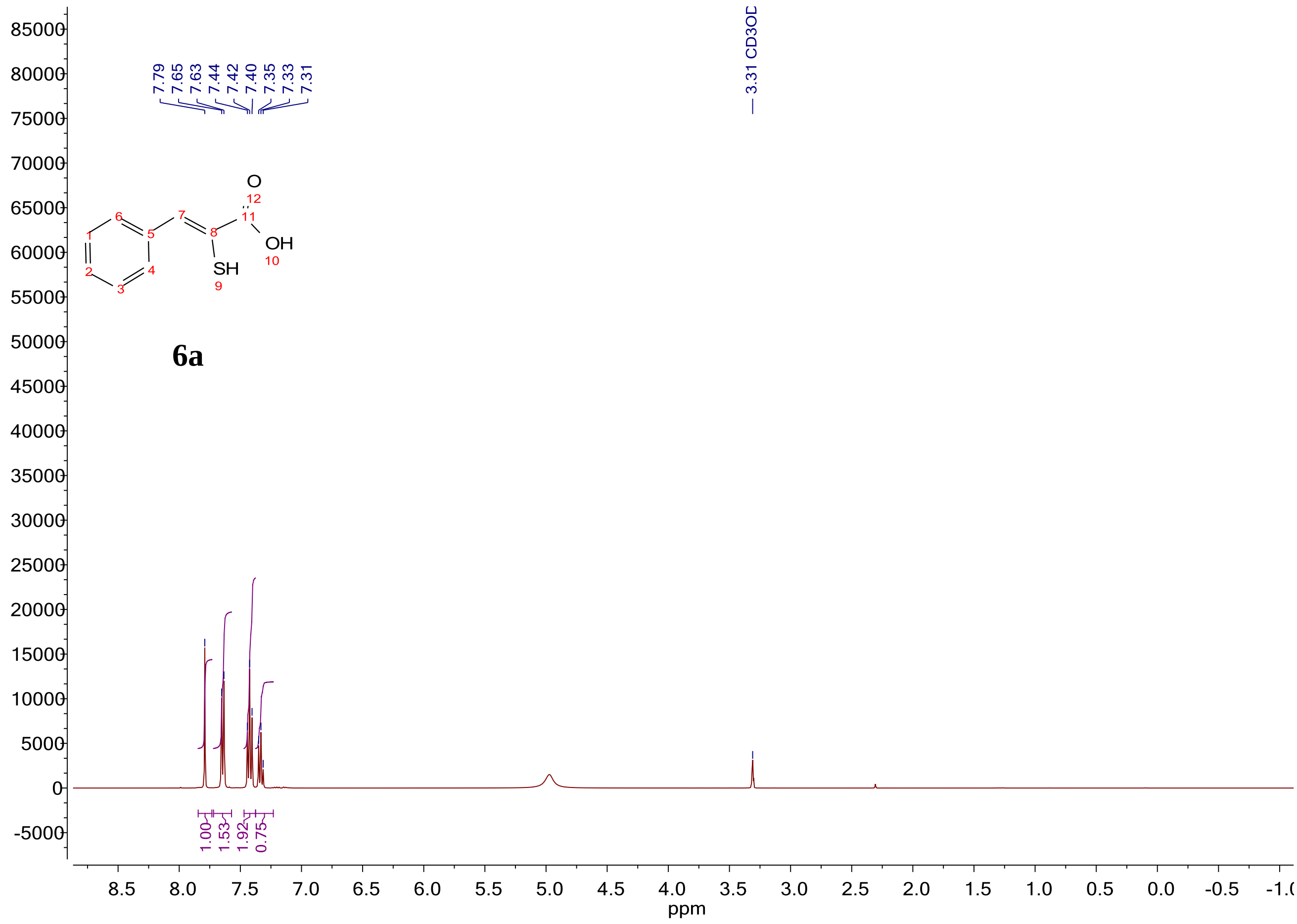
7.79
7.65
7.63
7.44
7.42
7.40
7.35
7.33
7.31
3.31 CD3OD
O
12
11
OH
10
SH
9
1
2
3
4
5
6
7
8
6a
1.00
1.53
1.92
0.75
ppm

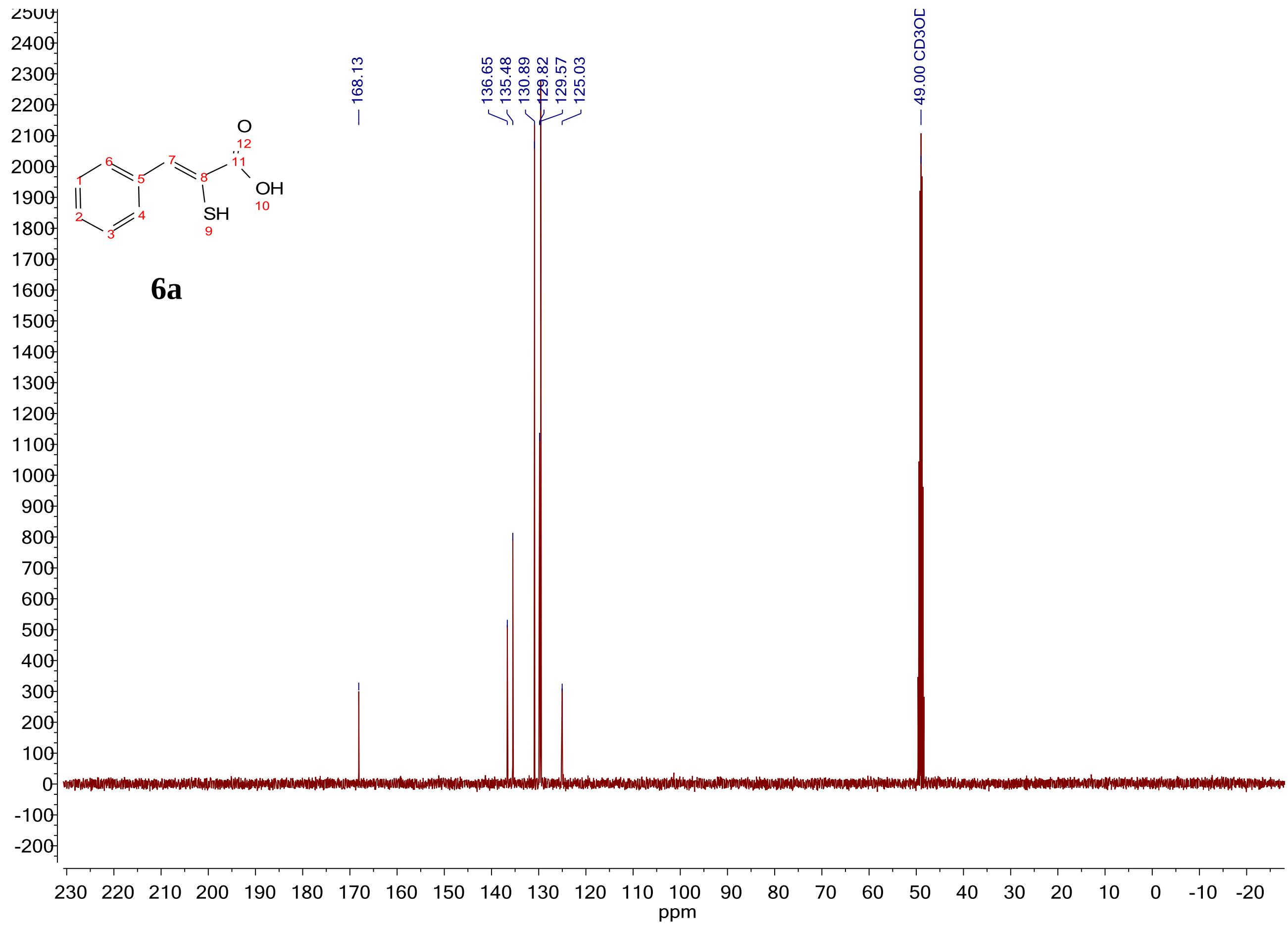
168.13
136.65
135.48
130.89
129.82
129.57
125.03
49.00 CD3OD
O
12
11
OH
10
SH
9
1
2
3
4
5
6
7
8
6a
ppm

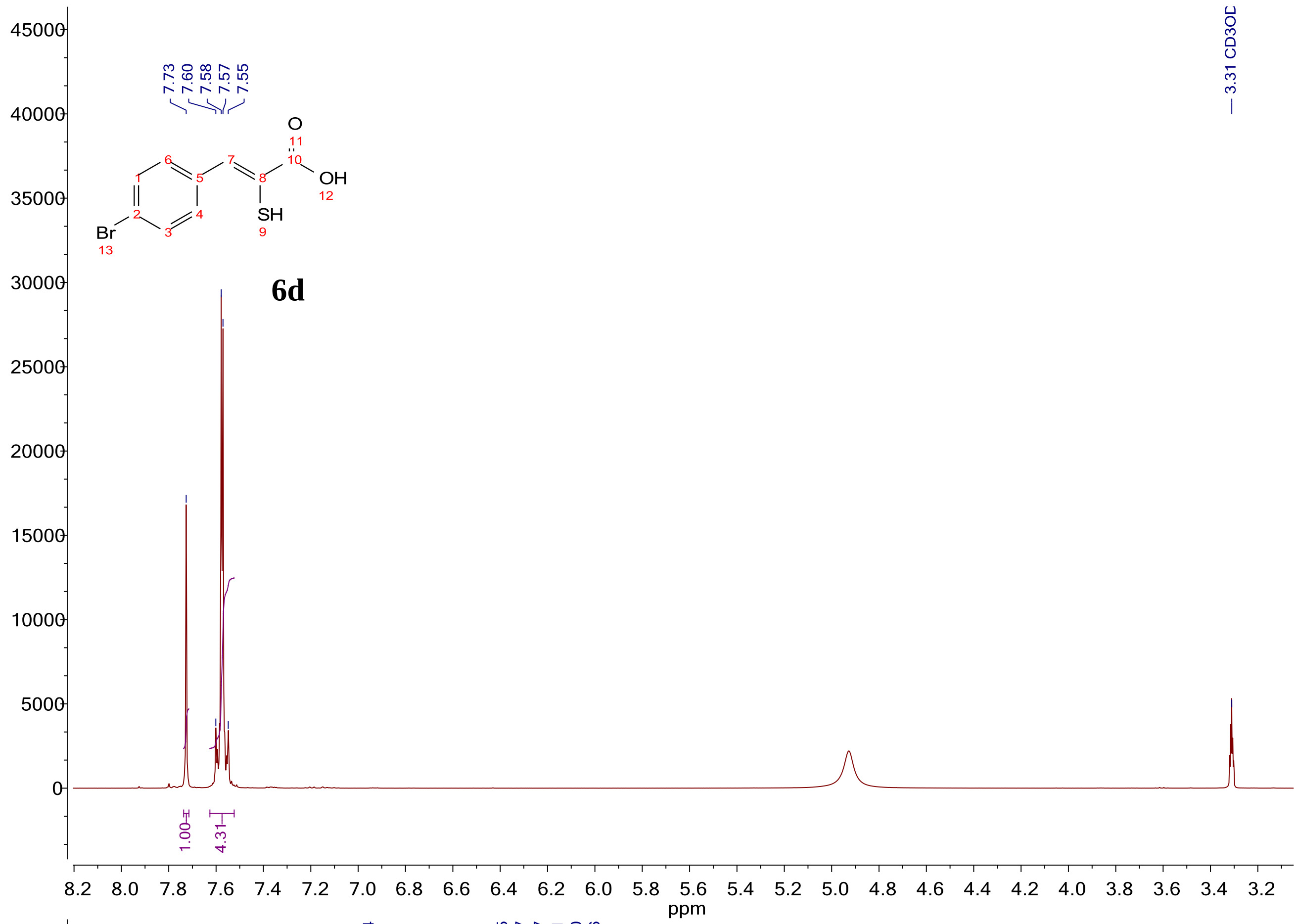
7.73
7.60
7.58
7.57
7.55
3.31 CD3OD
6d
1.00
4.31
ppm

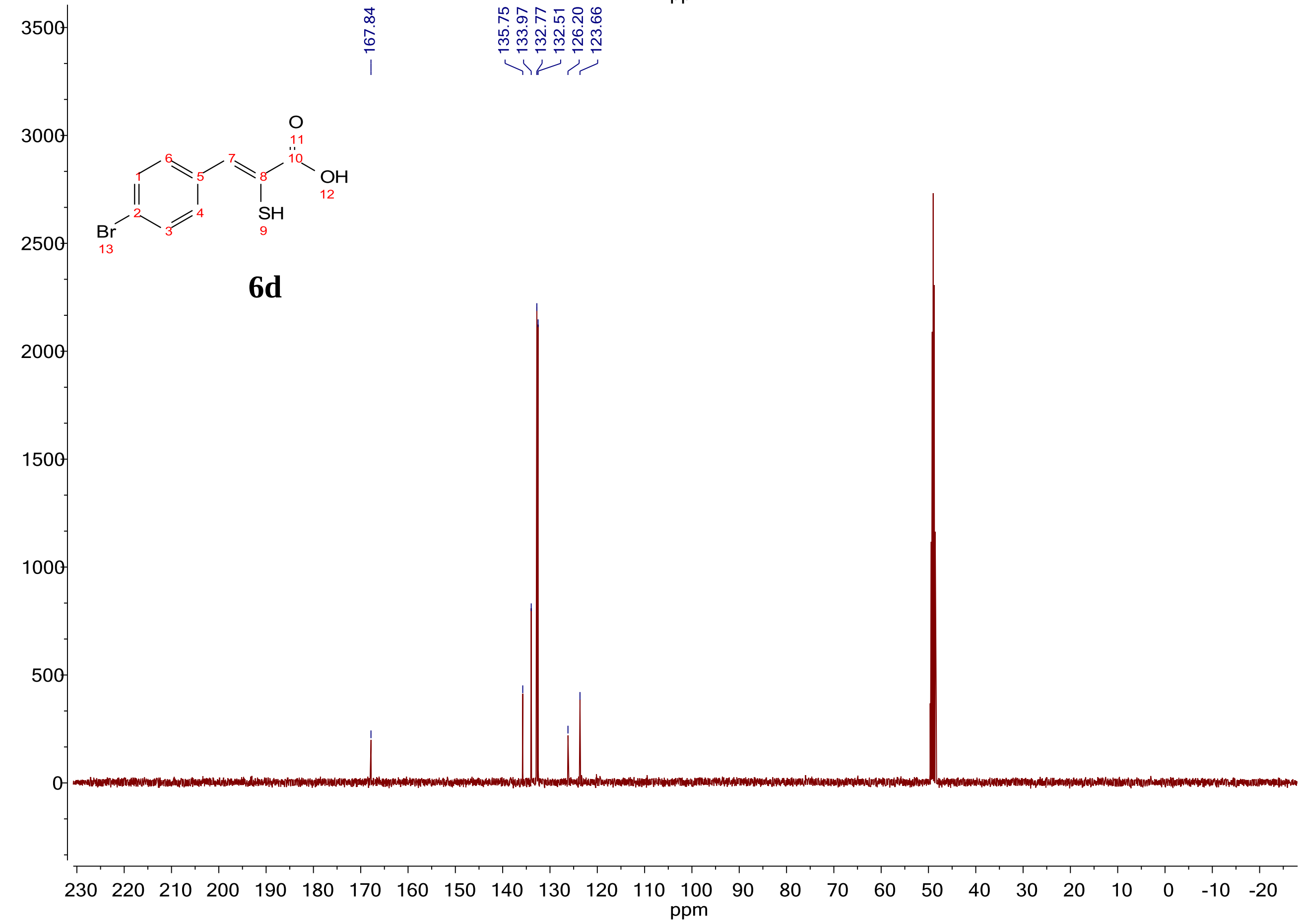
167.84
135.75
133.97
132.77
132.51
126.20
123.66
6d
ppm

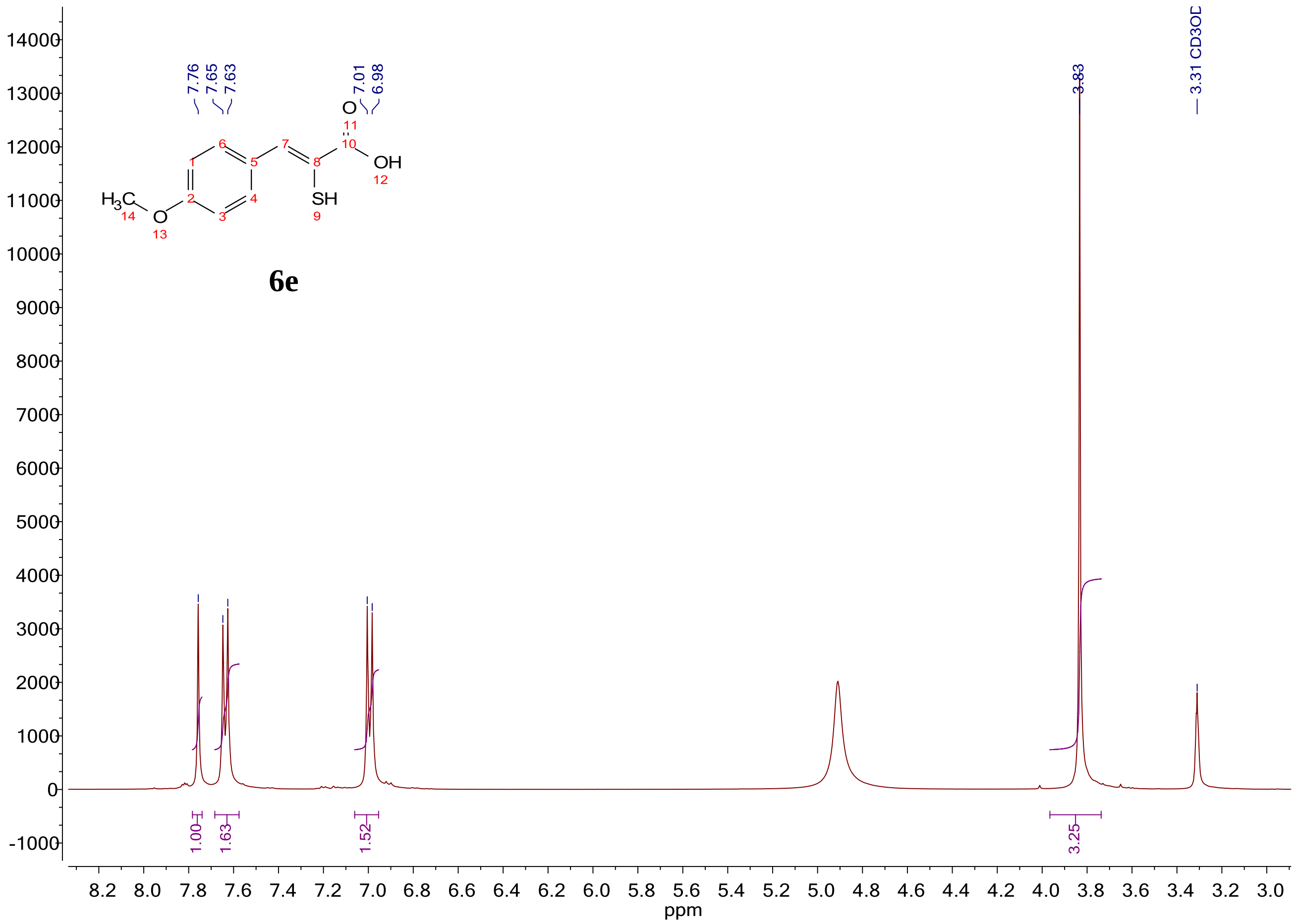
7.76
7.65
7.63
7.01
6.98
3.83
3.31 CD3OD
H3C
O
OH
SH
6e
1.00
1.63
1.52
3.25
ppm

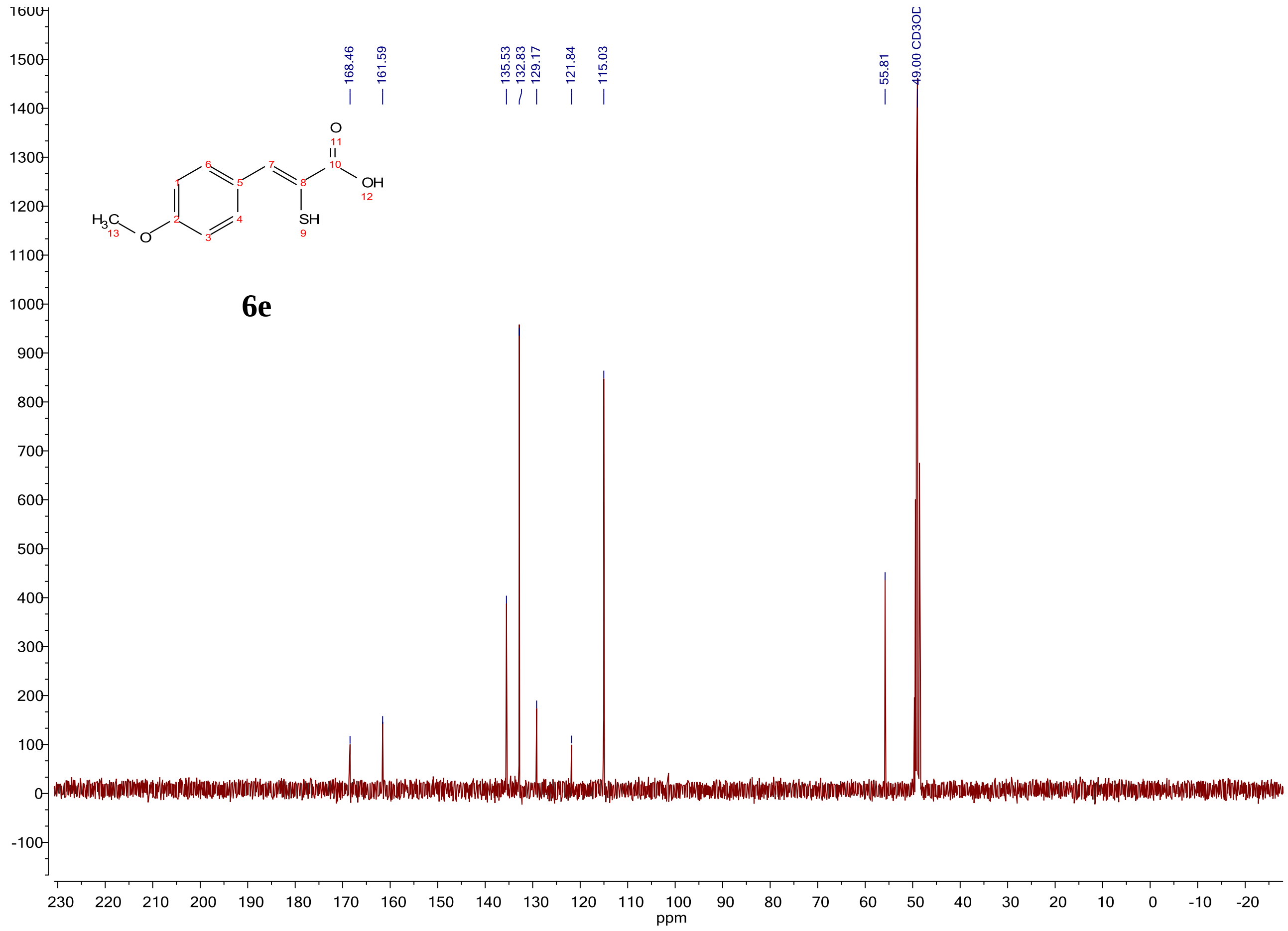
168.46
161.59
135.53
132.83
129.17
121.84
115.03
55.81
49.00 CD3OD
H3C
O
OH
SH
6e
ppm

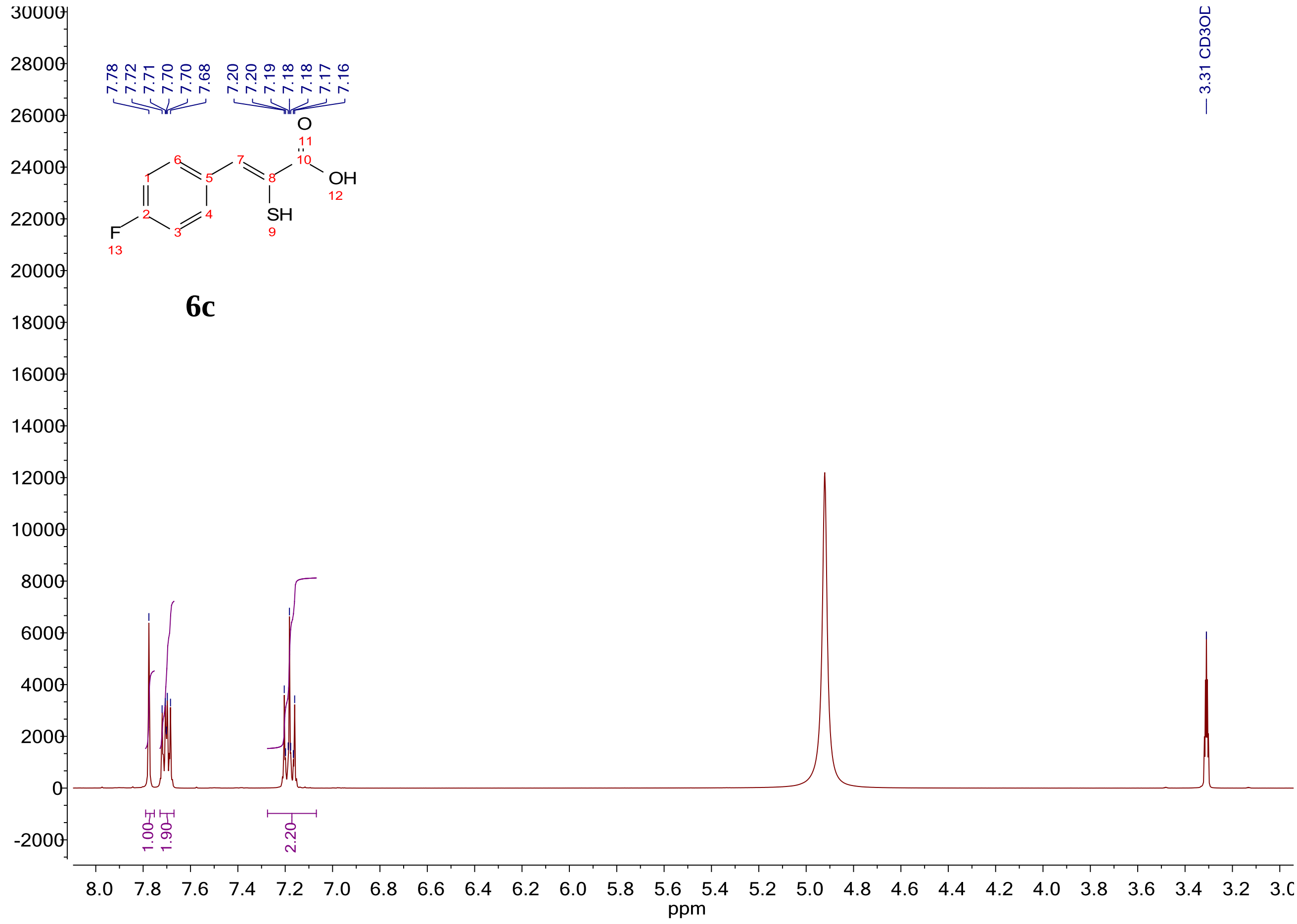

7.78
7.72
7.71
7.70
7.70
7.68
7.20
7.20
7.19
7.18
7.18
7.17
7.16
3.31 CD3OD
6c
1.00
1.90
2.20
ppm

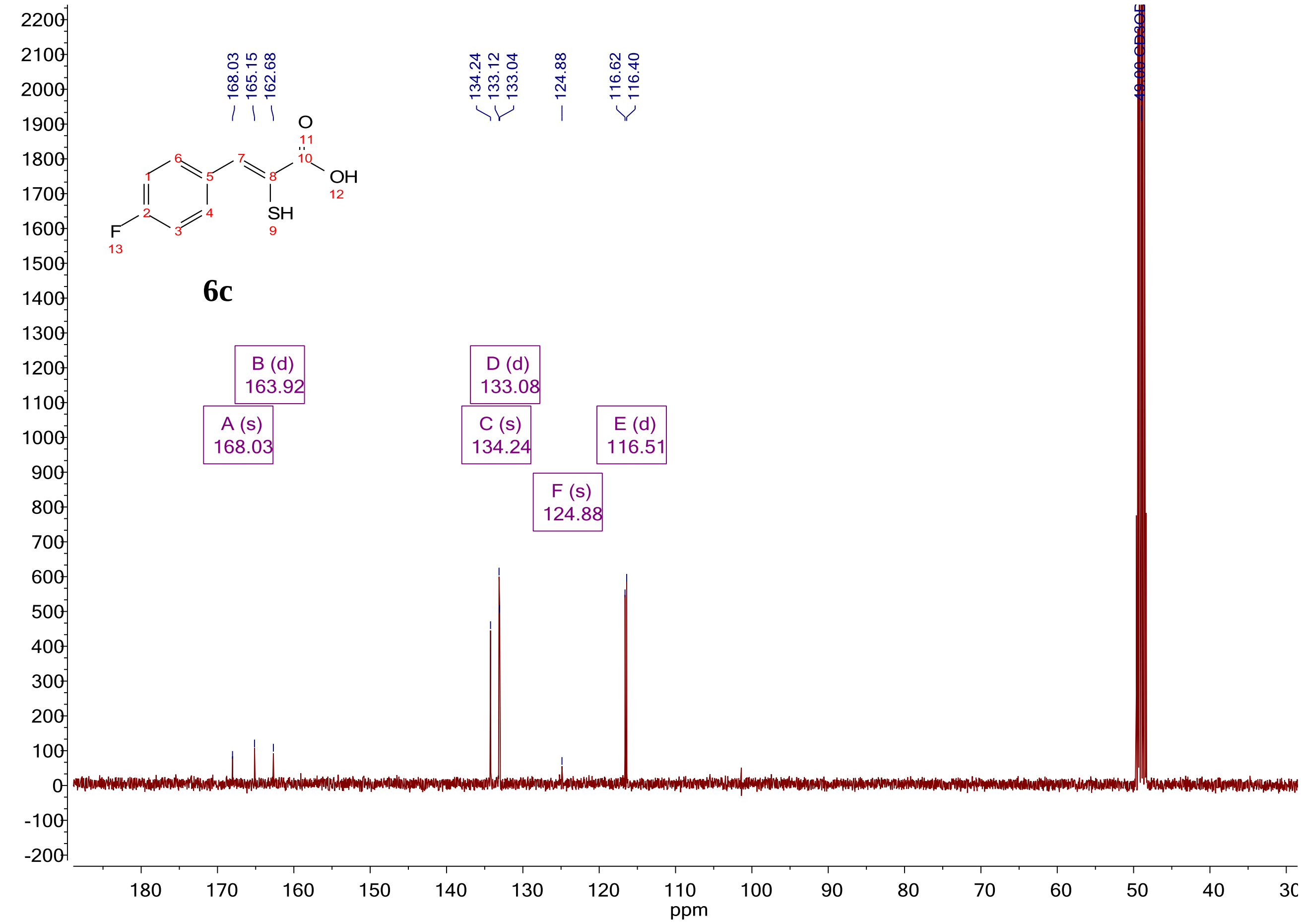

168.03
165.15
162.68
134.24
133.12
133.04
124.88
116.62
116.40
6c
B (d) 163.92
D (d) 133.08
A (s) 168.03
C (s) 134.24
E (d) 116.51
F (s) 124.88
ppm

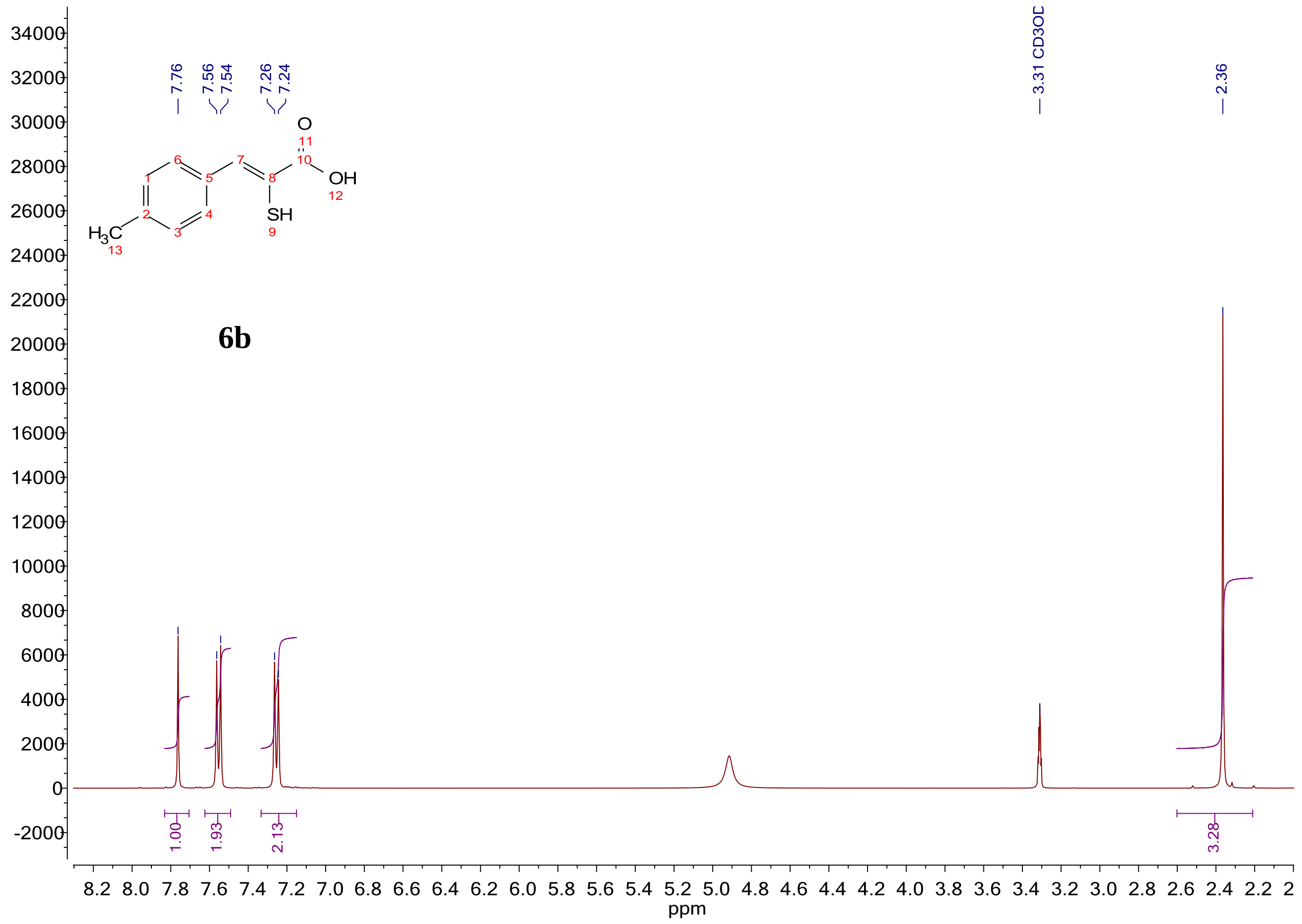
7.76
7.56
7.54
7.26
7.24
3.31 CD3OD
2.36
O
OH
SH
H3C
6b
1.00
1.93
2.13
3.28
ppm

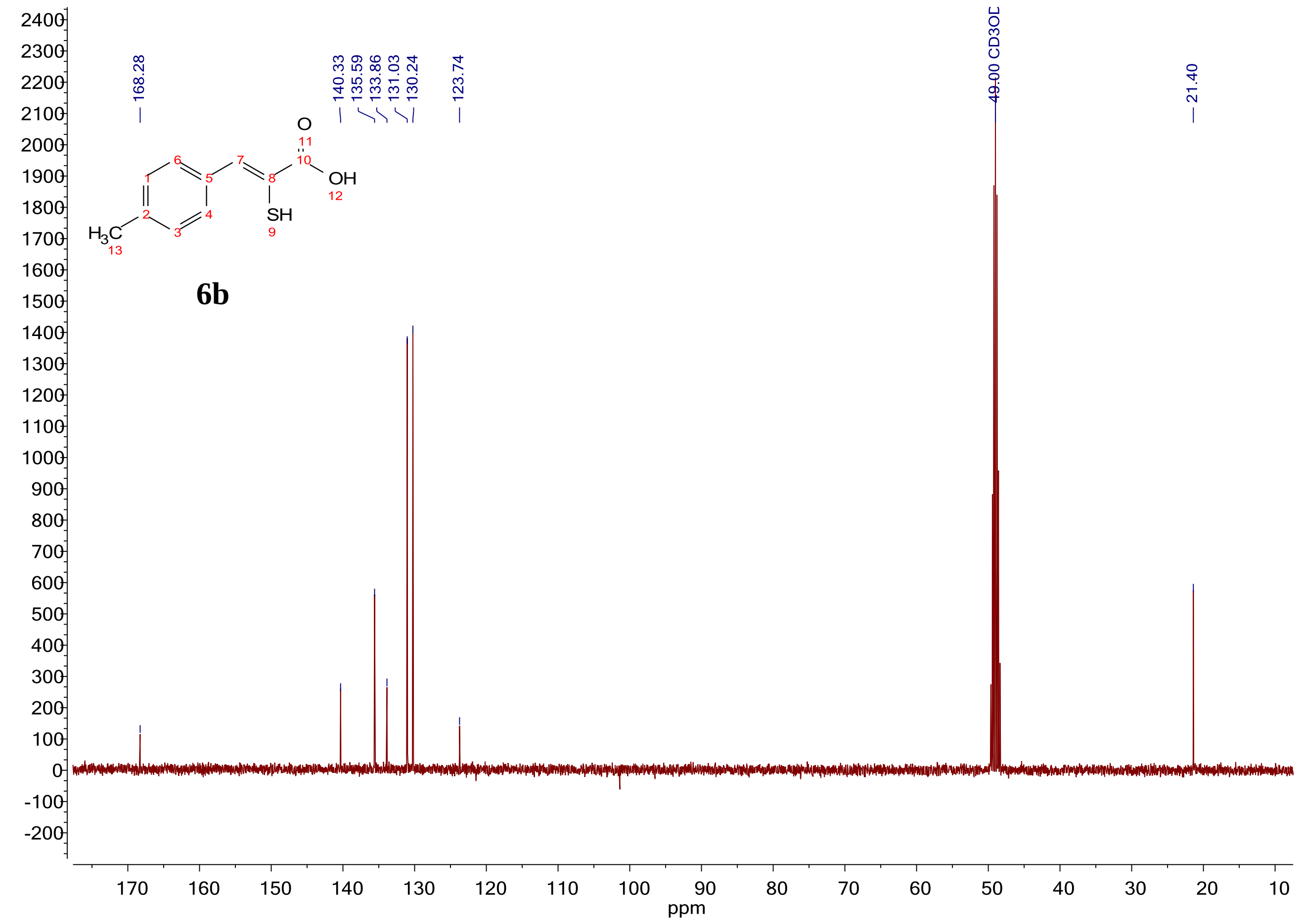
168.28
140.33
135.59
133.86
131.03
130.24
123.74
49.00 CD3OD
21.40
O
OH
SH
H3C
6b
ppm

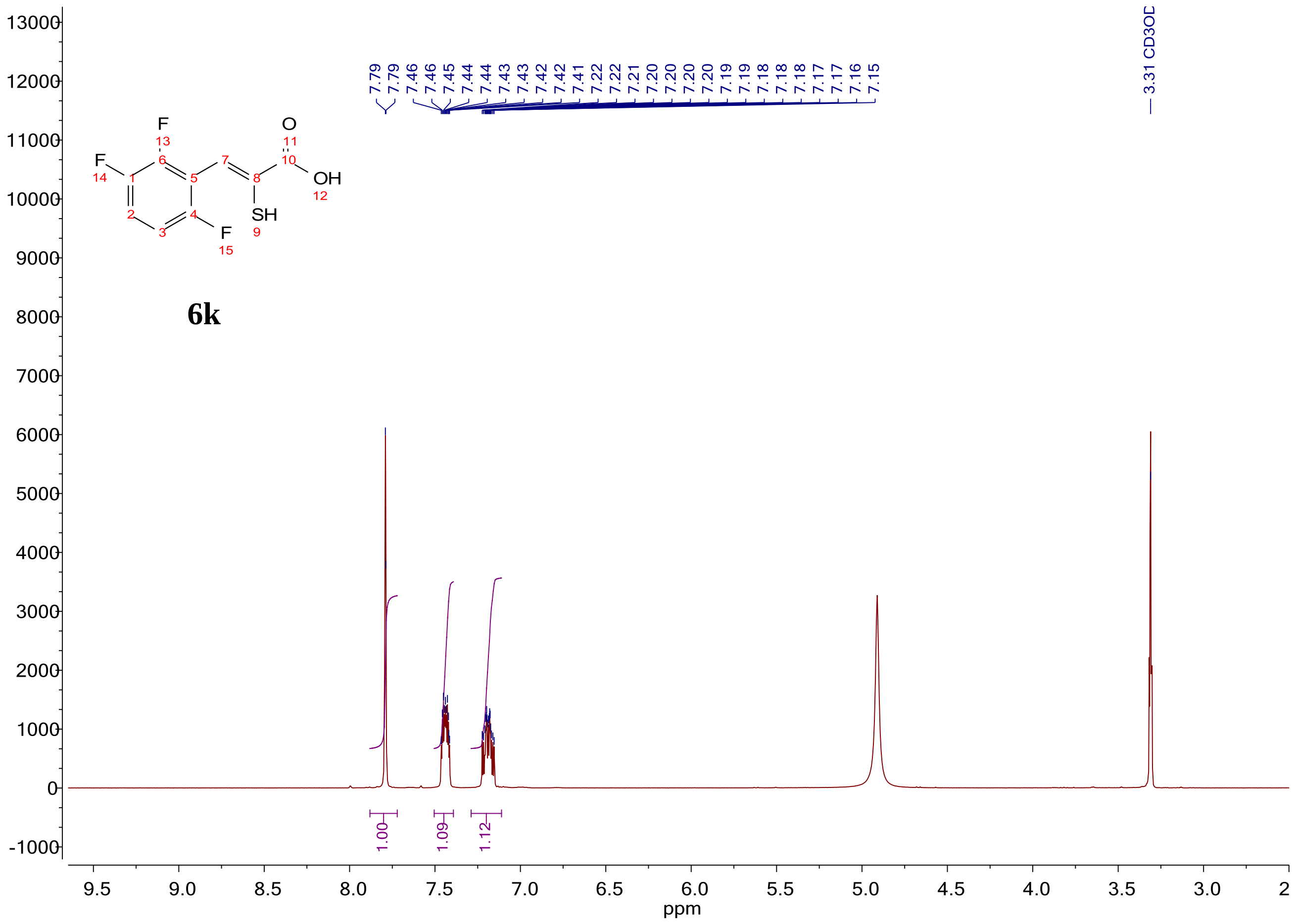

6k
7.79
7.79
7.46
7.45
7.44
7.44
7.43
7.43
7.42
7.42
7.41
7.22
7.22
7.21
7.20
7.20
7.20
7.20
7.19
7.19
7.18
7.18
7.17
7.17
7.16
7.15
3.31 CD3OD
1.00
1.09
1.12
ppm

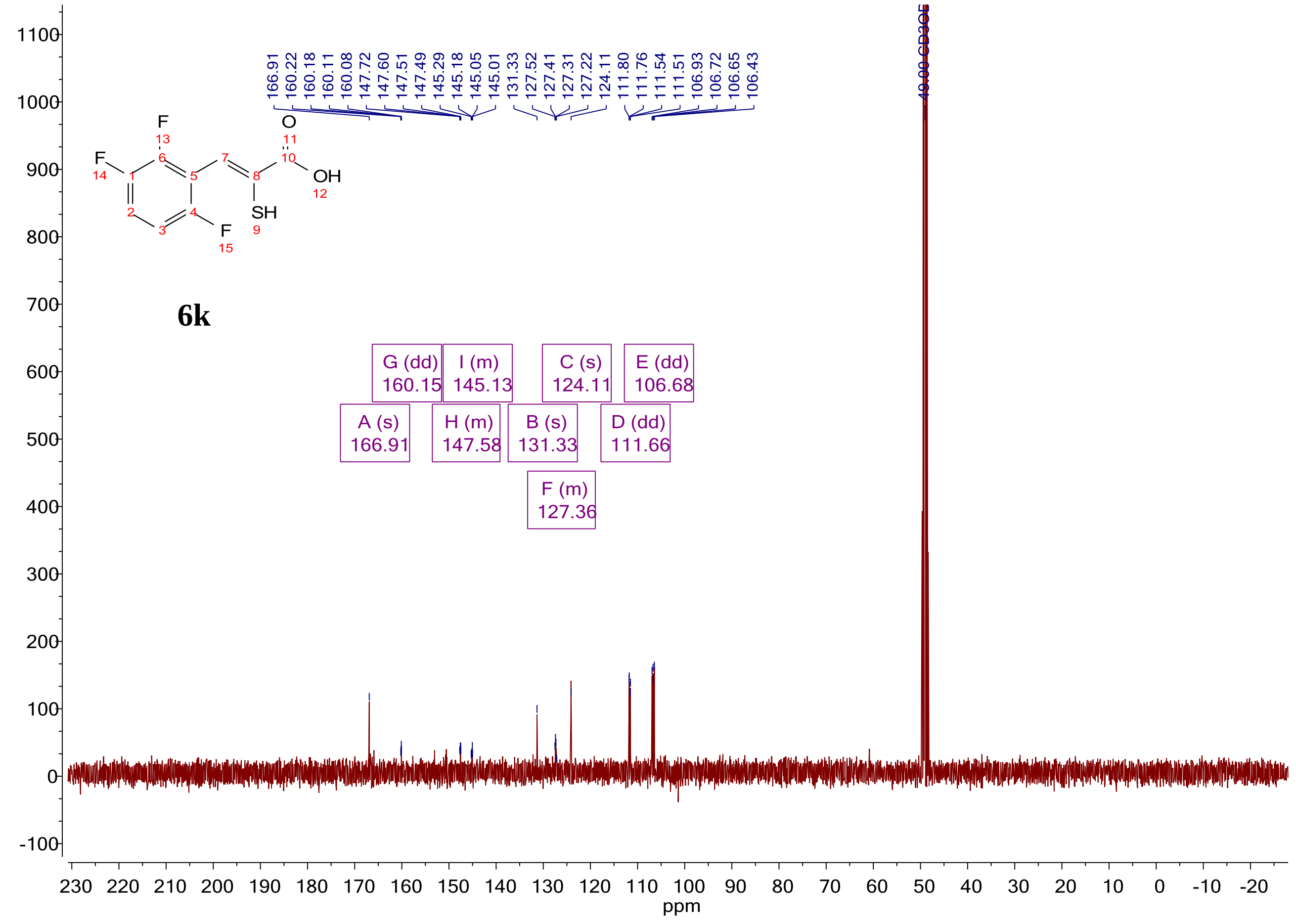

6k
166.91
160.22
160.18
160.11
160.08
147.72
147.60
147.51
147.49
145.29
145.18
145.05
145.01
131.33
127.52
127.41
127.31
127.22
124.11
111.80
111.76
111.54
111.51
106.93
106.72
106.65
106.43
G (dd) 160.15
I (m) 145.13
C (s) 124.11
E (dd) 106.68
A (s) 166.91
H (m) 147.58
B (s) 131.33
D (dd) 111.66
F (m) 127.36
ppm

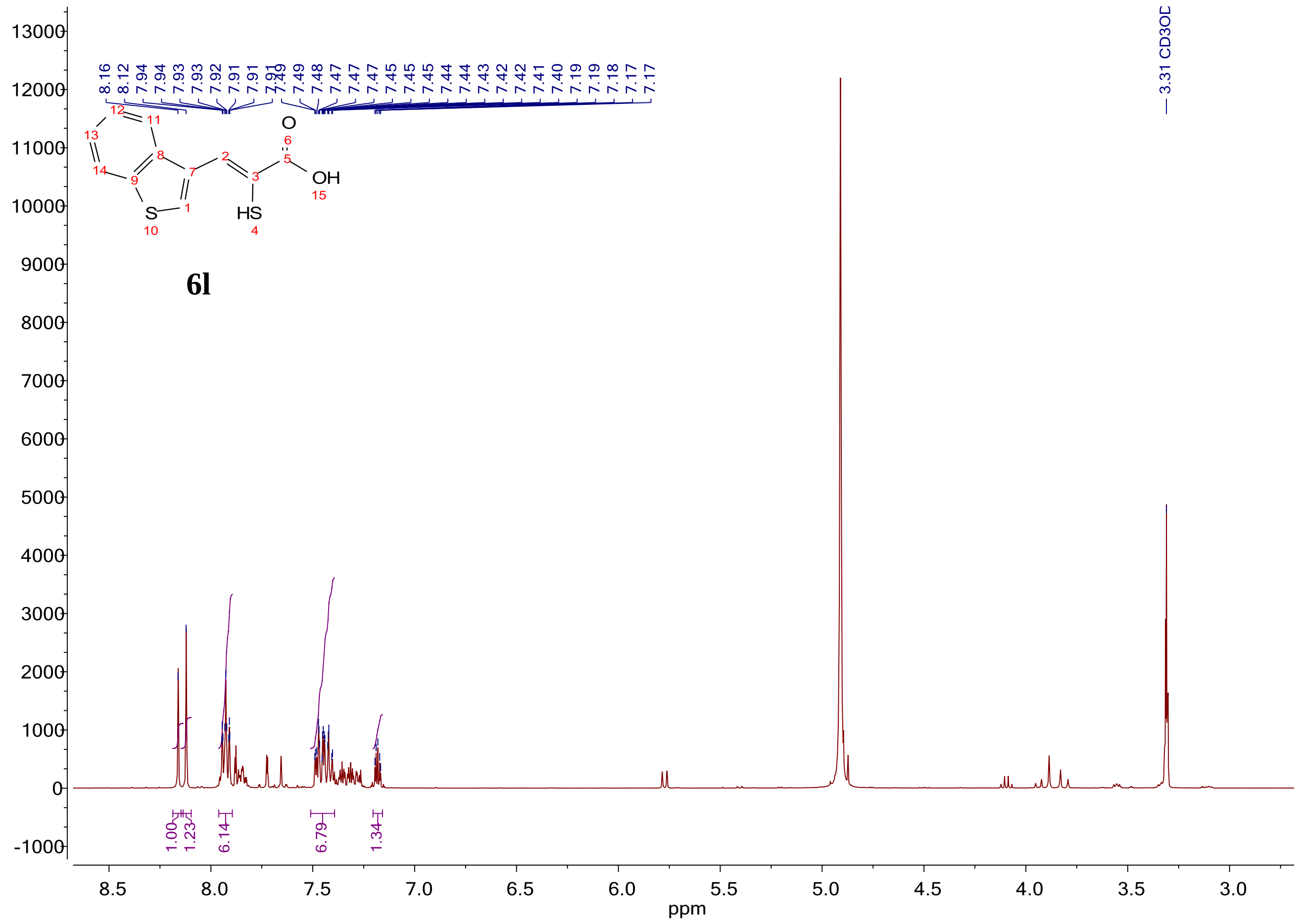

8.16
8.12
7.94
7.94
7.93
7.93
7.92
7.91
7.91
7.49
7.48
7.47
7.47
7.47
7.45
7.45
7.45
7.44
7.44
7.43
7.42
7.42
7.41
7.40
7.19
7.19
7.18
7.17
7.17
3.31 CD3OD
O
OH
HS
S
6l
1.00
1.23
6.14
6.79
1.34
ppm

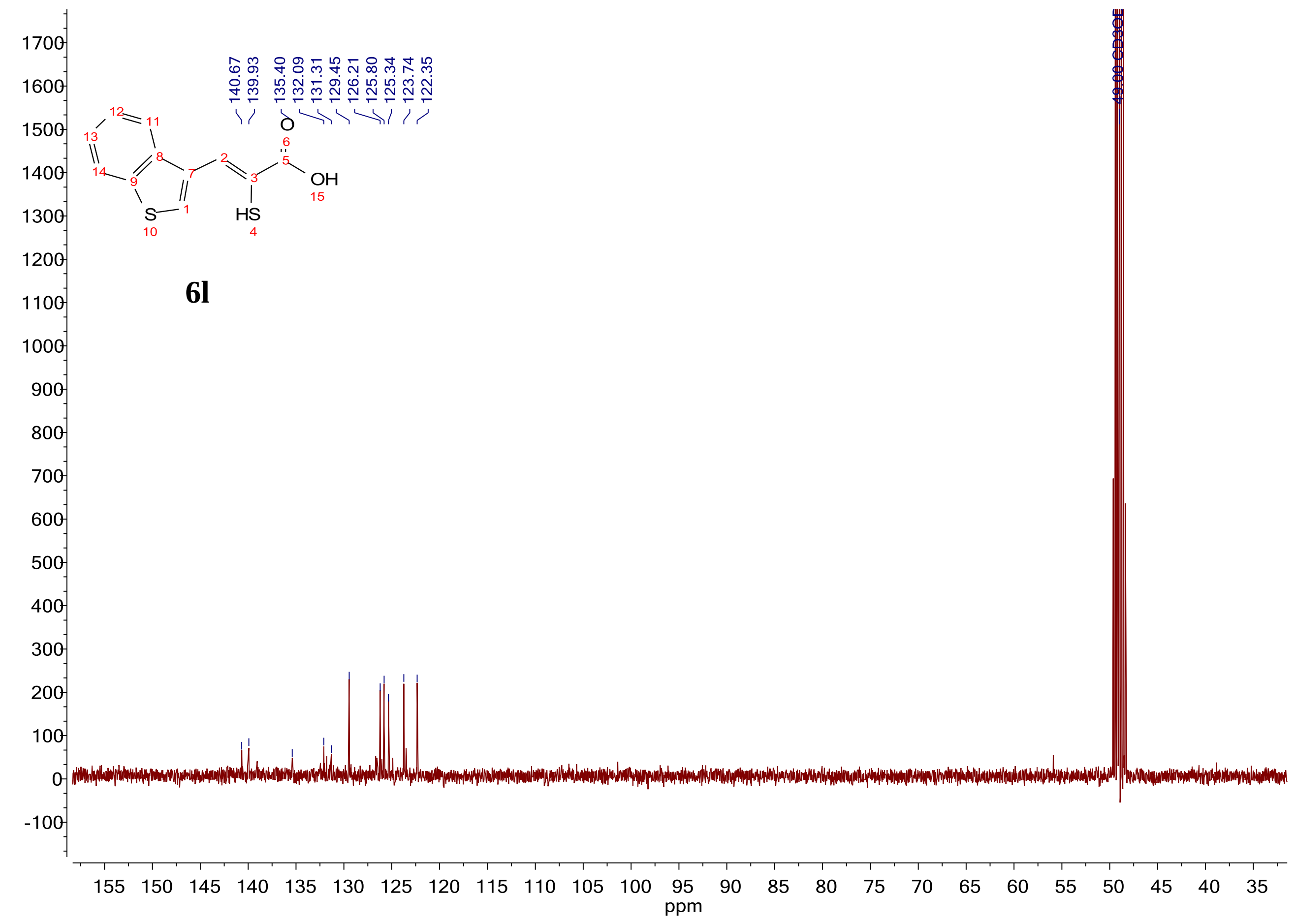

140.67
139.93
135.40
132.09
131.31
129.45
126.21
125.80
125.34
123.74
122.35
49.00 CD3OD
O
OH
HS
S
6l
ppm

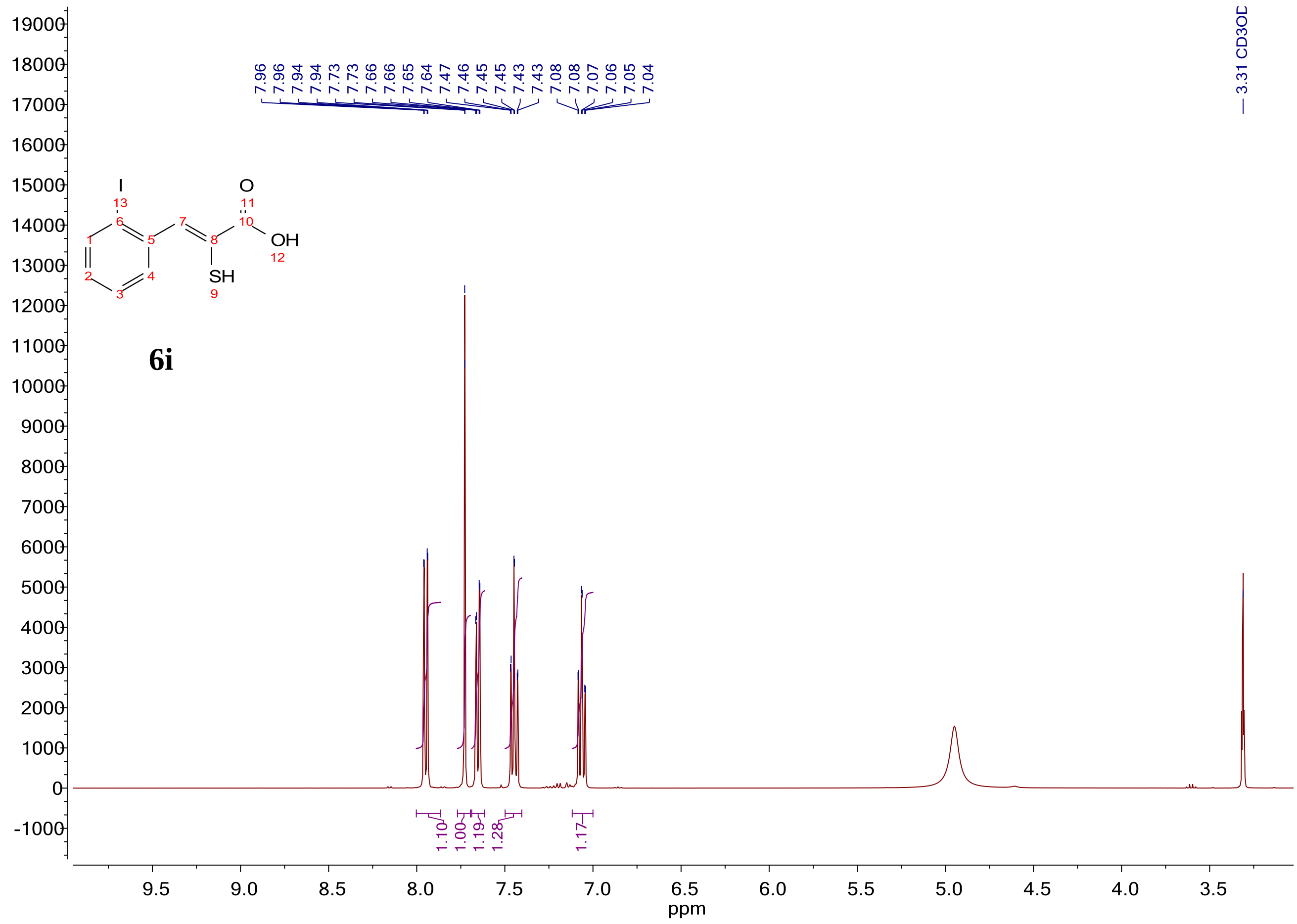

7.96
7.96
7.94
7.94
7.73
7.73
7.66
7.66
7.65
7.64
7.47
7.46
7.45
7.45
7.43
7.43
7.08
7.08
7.07
7.06
7.05
7.04
3.31 CD3OD
6i
1.10
1.00
1.19
1.28
1.17
ppm

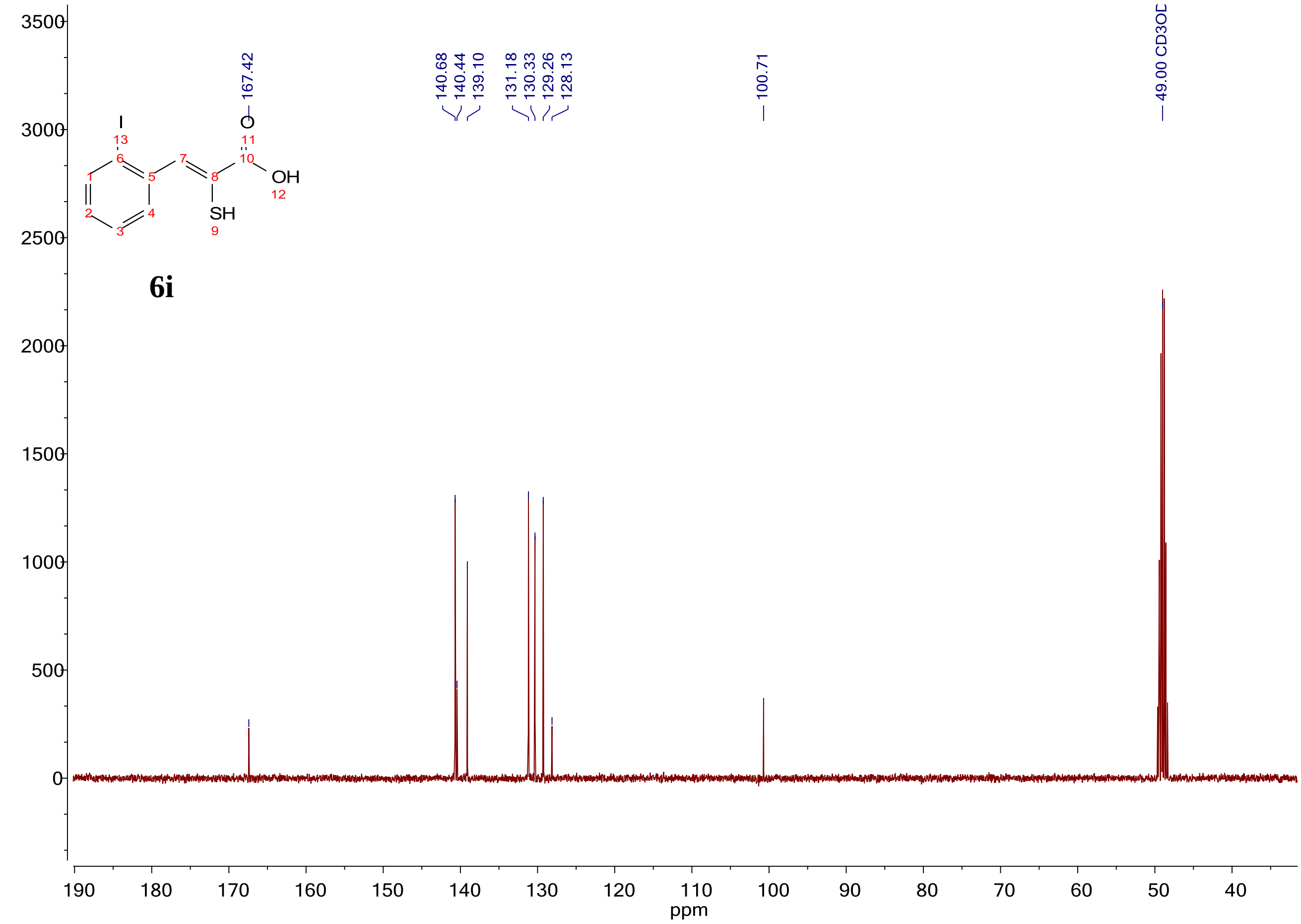

167.42
140.68
140.44
139.10
131.18
130.33
129.26
128.13
100.71
49.00 CD3OD
6i
ppm

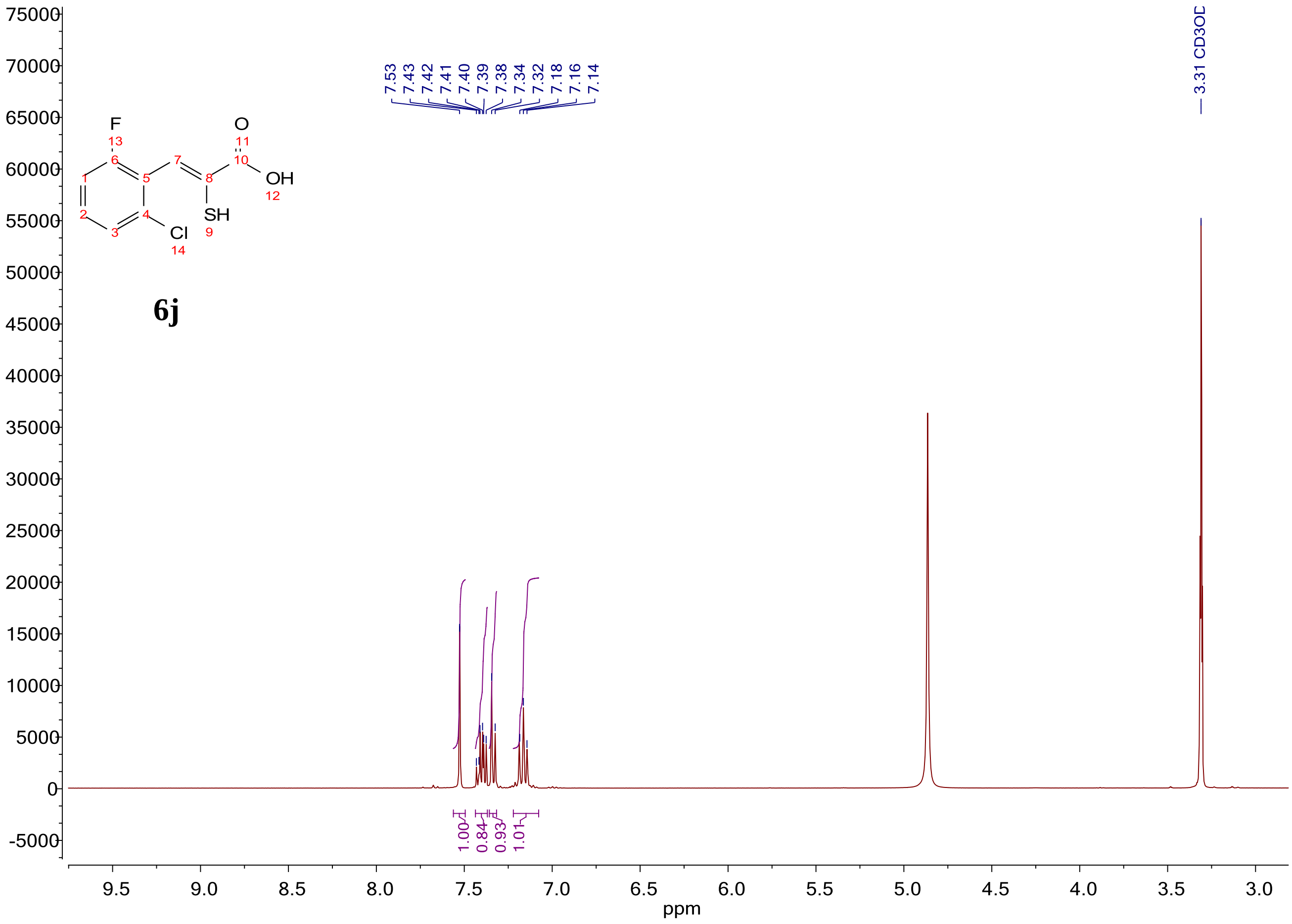
7.53
7.43
7.42
7.41
7.40
7.39
7.38
7.34
7.32
7.18
7.16
7.14
3.31 CD3OD
F
13
6
1
2
3
4
5
Cl
14
7
8
SH
9
10
O
11
OH
12
6j
1.00
0.84
0.93
1.01
ppm

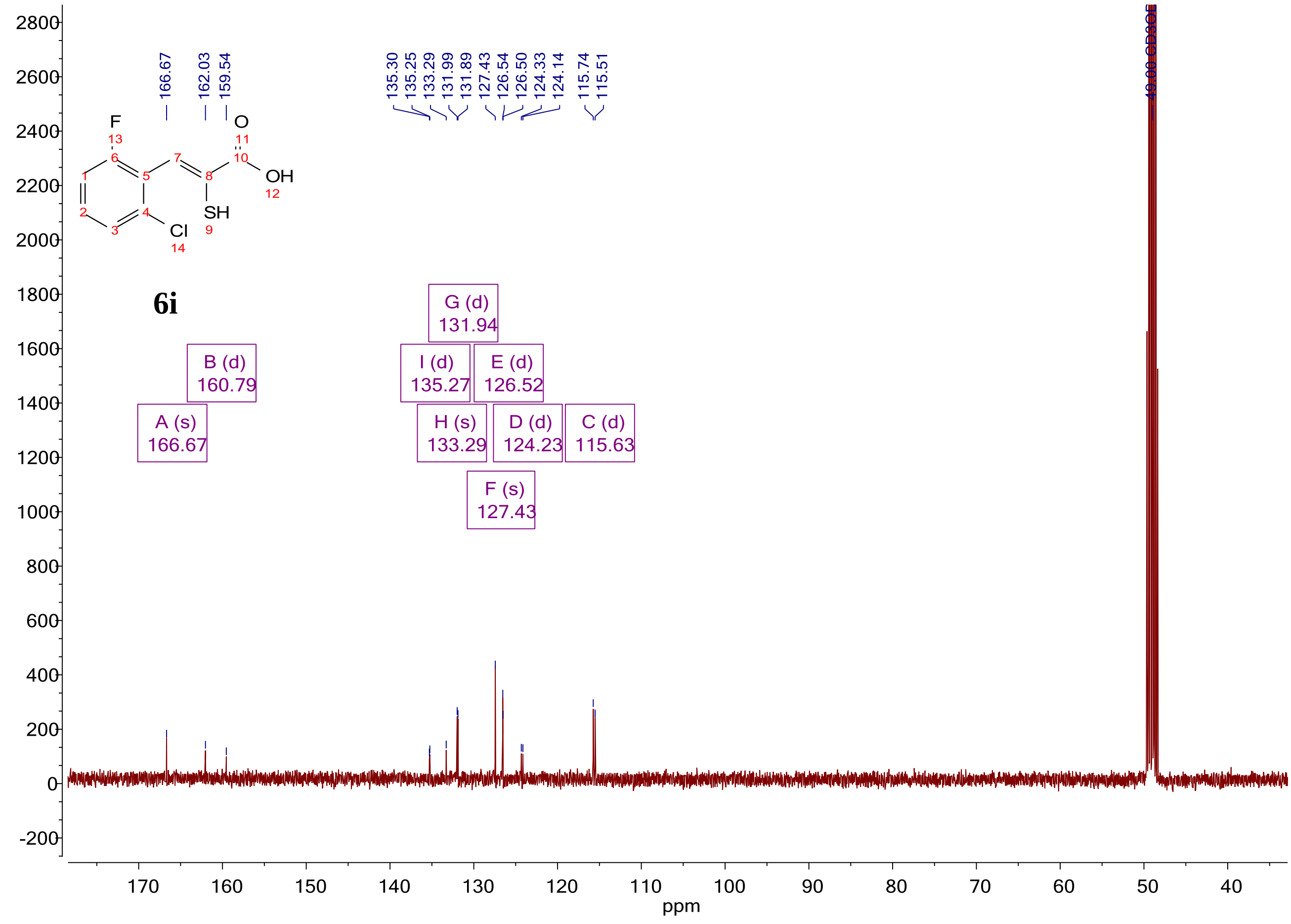
166.67
162.03
159.54
135.30
135.25
133.29
131.99
131.89
127.43
126.54
126.50
124.33
124.14
115.74
115.51
6i
G (d)
131.94
B (d)
160.79
I (d)
135.27
E (d)
126.52
A (s)
166.67
H (s)
133.29
D (d)
124.23
C (d)
115.63
F (s)
127.43
ppm

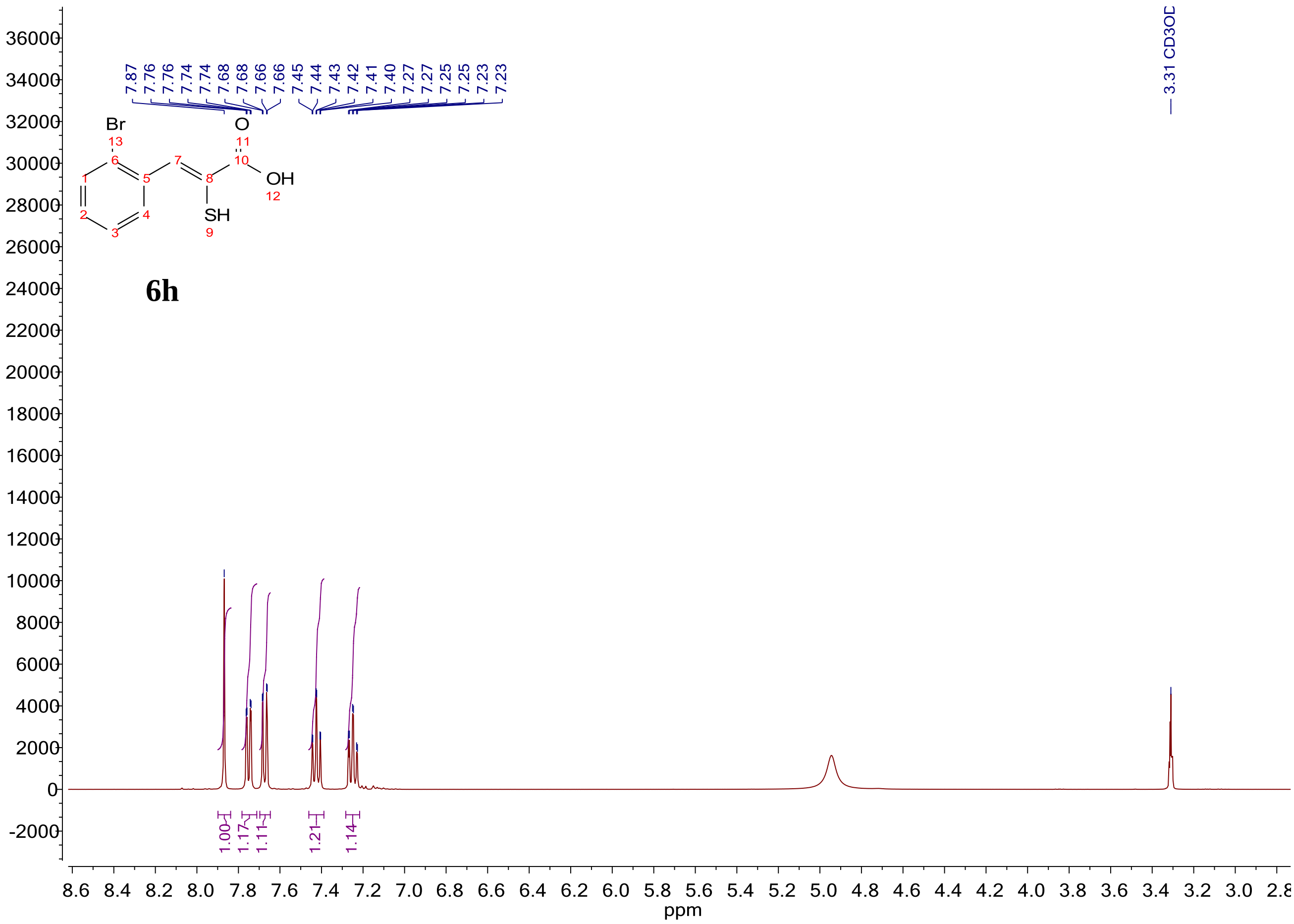
7.87
7.76
7.76
7.74
7.74
7.68
7.68
7.66
7.66
7.45
7.44
7.43
7.42
7.41
7.40
7.27
7.27
7.25
7.25
7.23
7.23
3.31 CD3OD
Br
O
OH
SH
6h
1.00
1.17
1.11
1.21
1.14
ppm

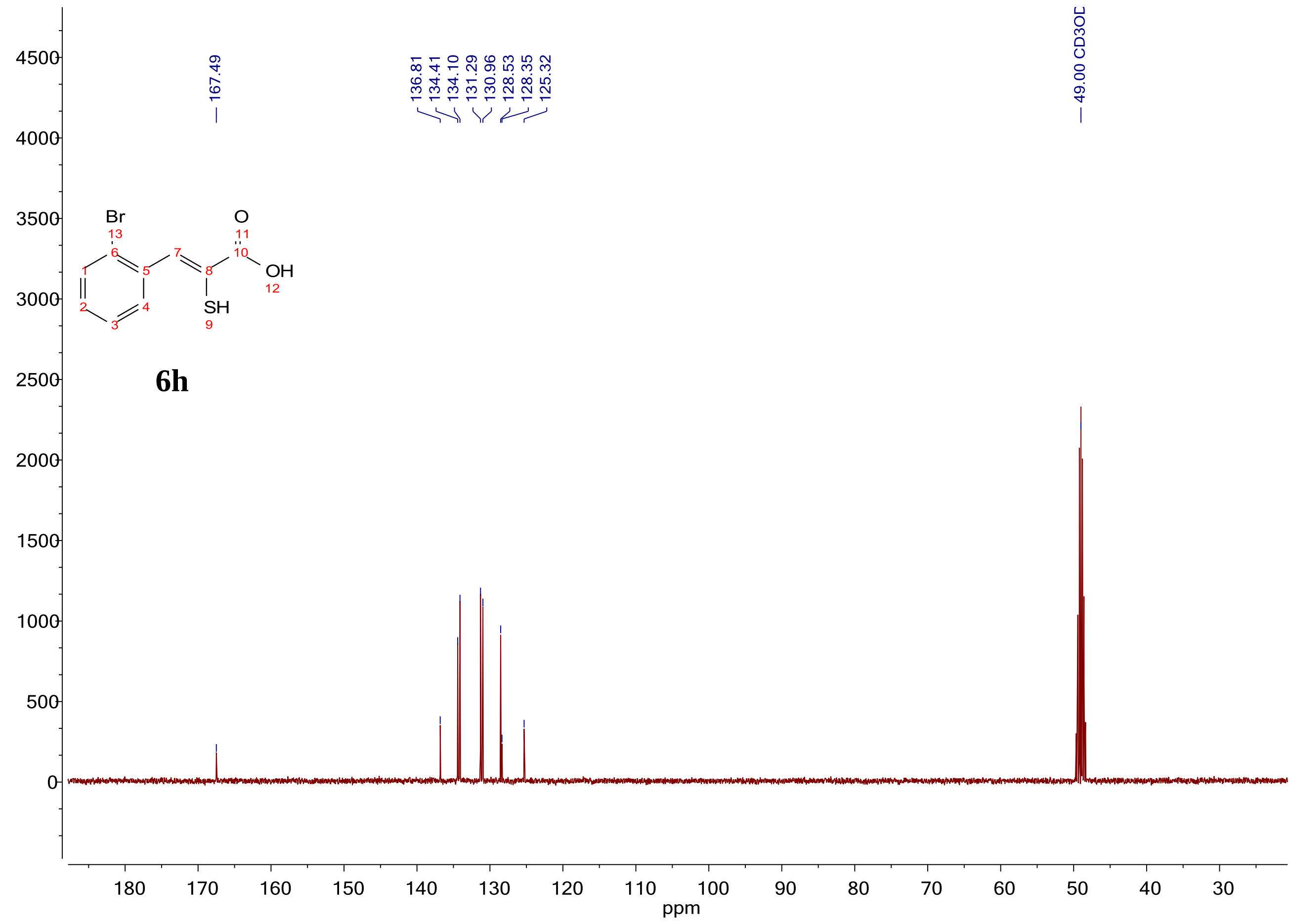
167.49
136.81
134.41
134.10
131.29
130.96
128.53
128.35
125.32
49.00 CD3OD
Br
O
OH
SH
6h
ppm

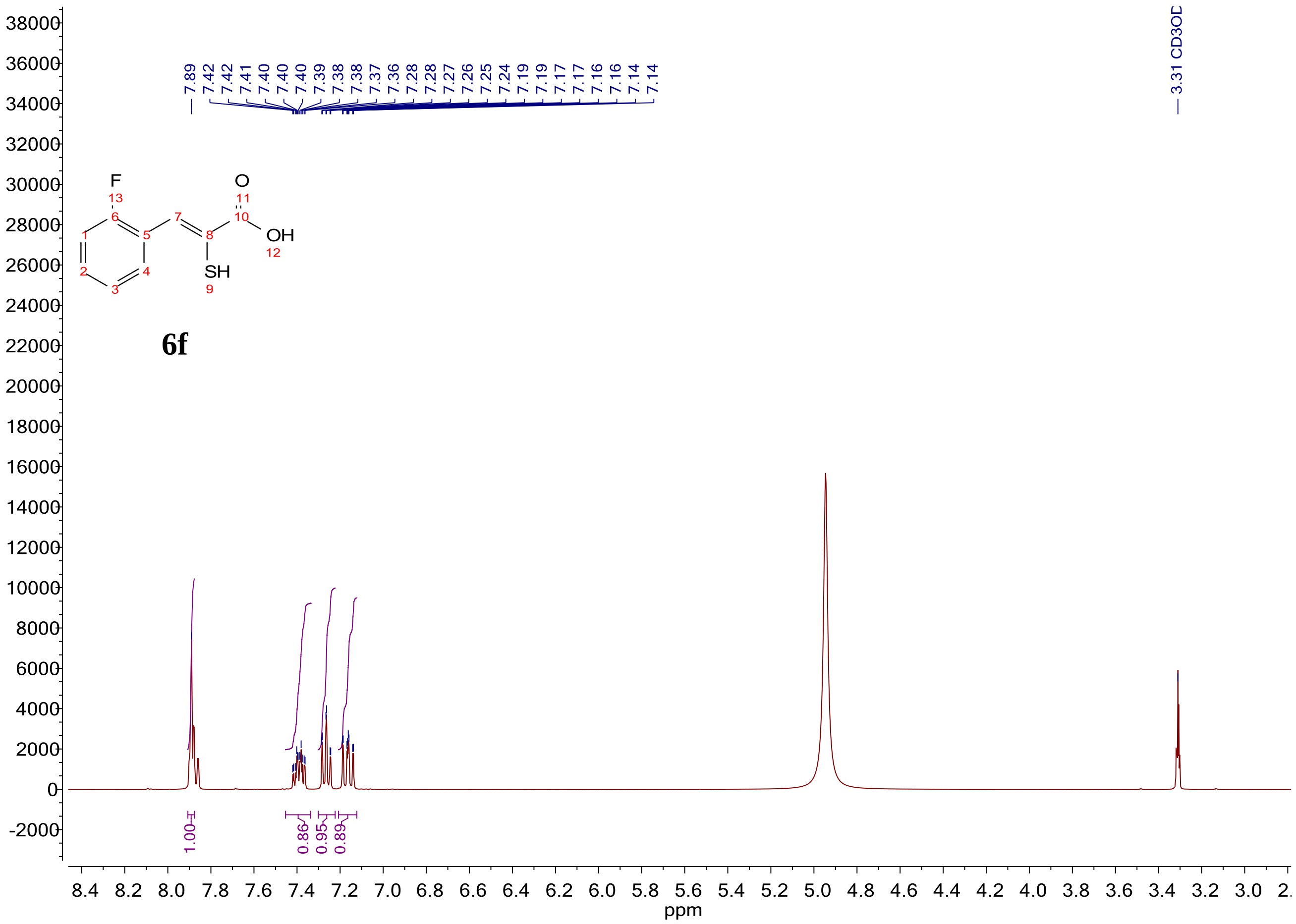
7.89 7.42 7.42 7.41 7.40 7.40 7.40 7.39 7.38 7.38 7.37 7.36 7.28 7.28 7.27 7.26 7.25 7.24 7.19 7.19 7.17 7.17 7.16 7.16 7.14 7.14
3.31 CD3OD
F
O
OH
SH
6f
1.00
0.86
0.95
0.89
ppm

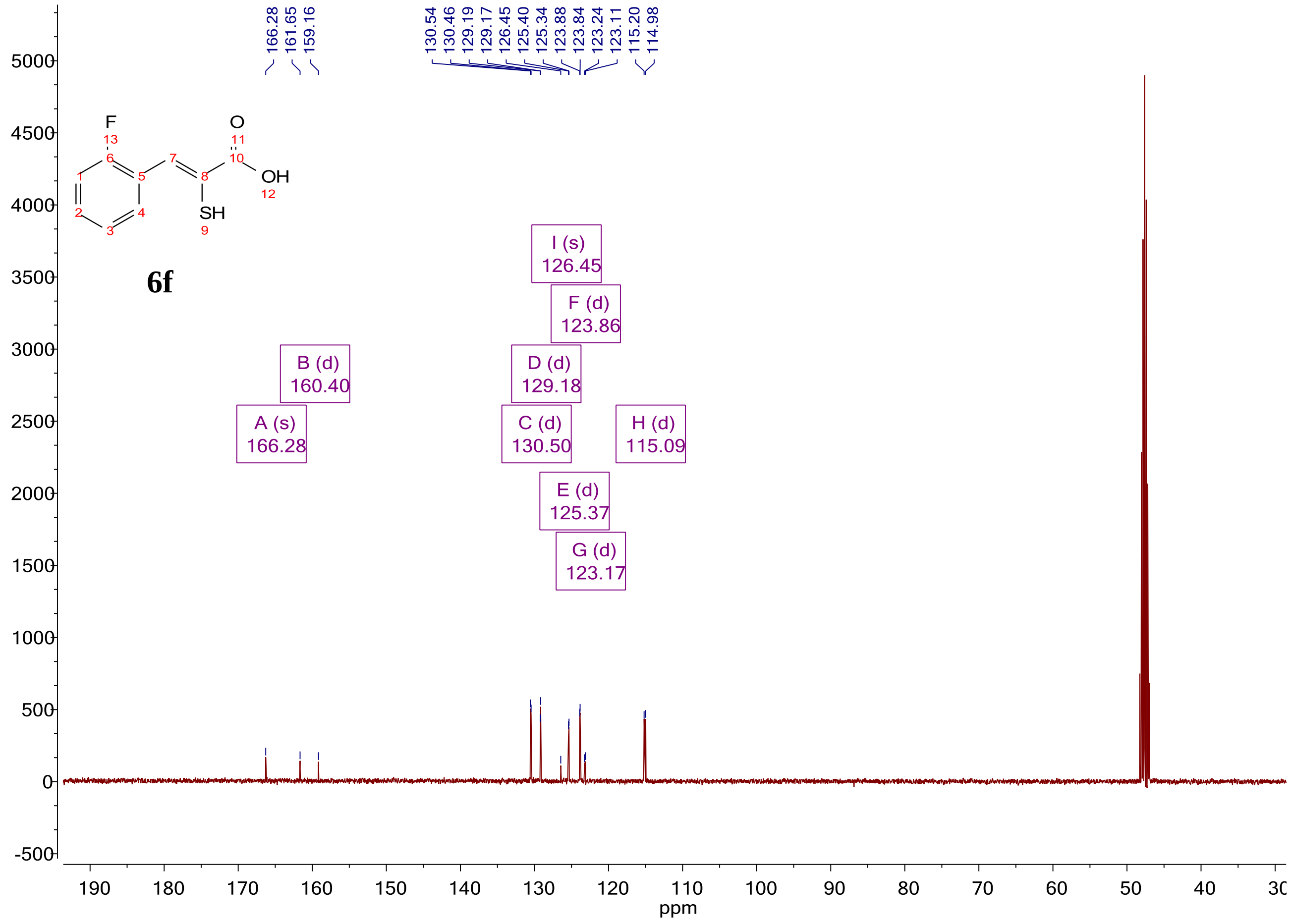
166.28 161.65 159.16
130.54 130.46 129.19 129.17 126.45 125.40 125.34 123.88 123.84 123.24 123.11 115.20 114.98
F
O
OH
SH
6f
I (s)
126.45
F (d)
123.86
B (d)
160.40
D (d)
129.18
A (s)
166.28
C (d)
130.50
H (d)
115.09
E (d)
125.37
G (d)
123.17
ppm

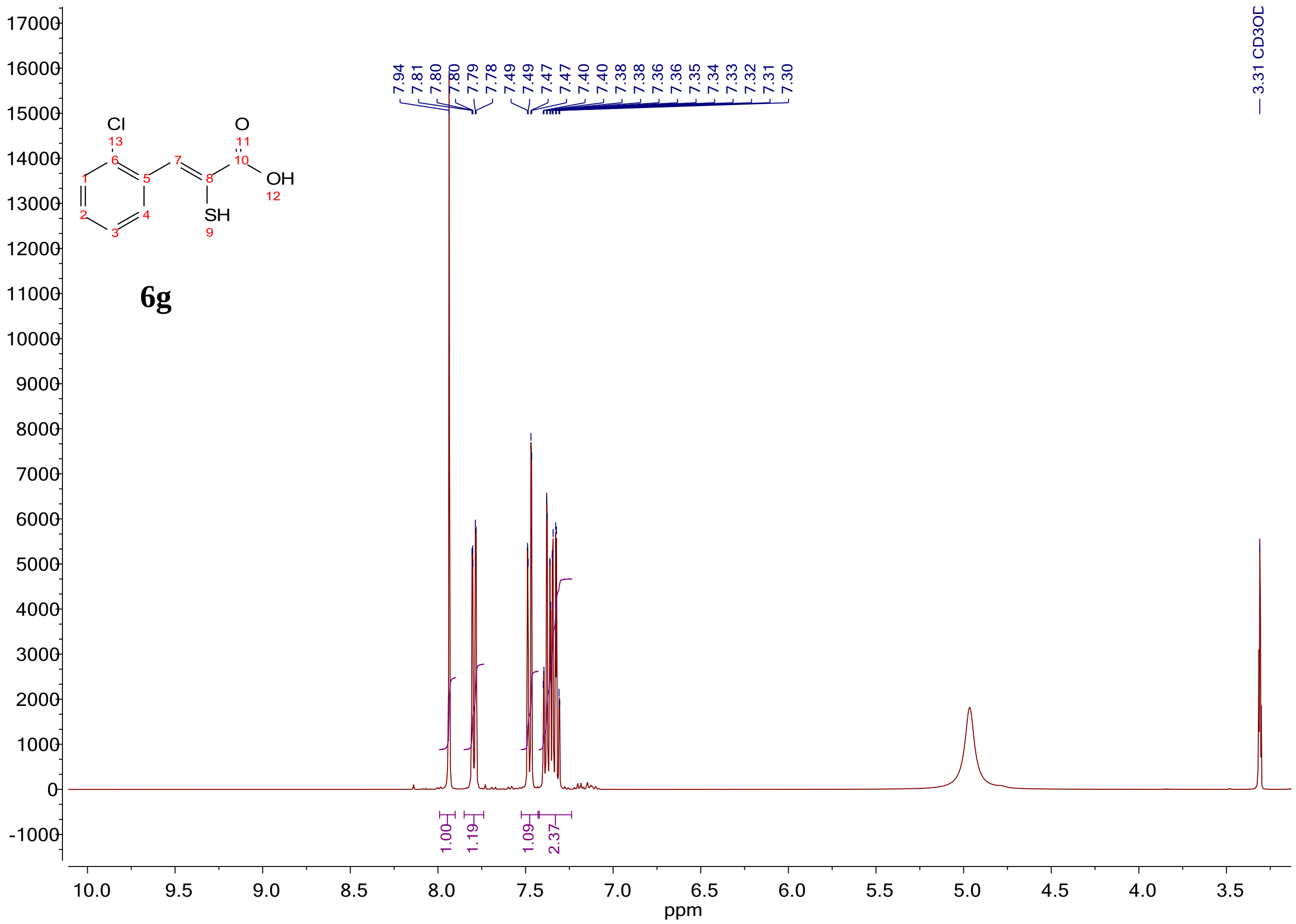
6g
7.94
7.81
7.80
7.80
7.79
7.78
7.49
7.49
7.47
7.47
7.40
7.40
7.38
7.38
7.36
7.36
7.35
7.34
7.33
7.32
7.31
7.30
3.31 CD3OD
1.00
1.19
1.09
2.37
ppm

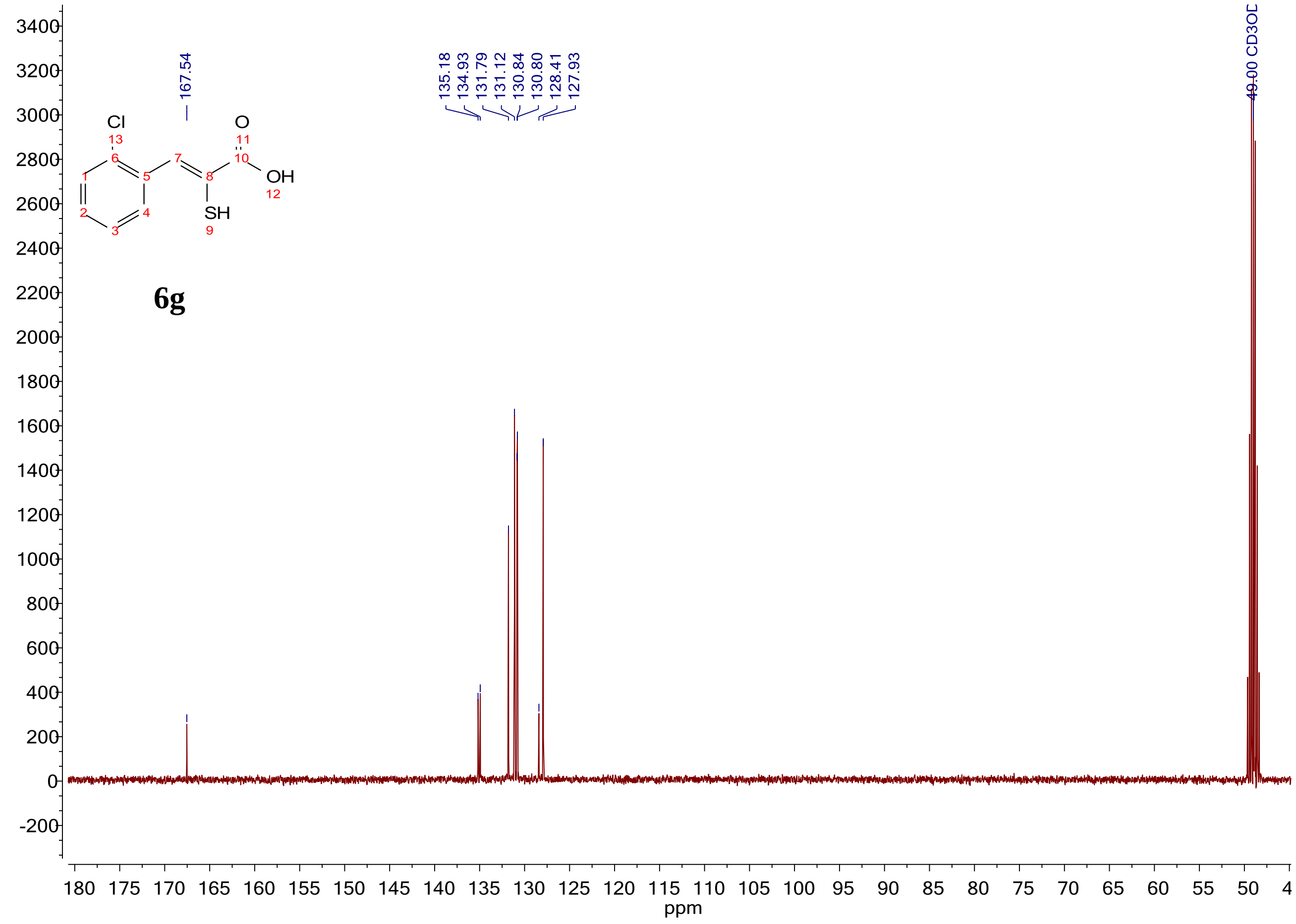
6g
167.54
135.18
134.93
131.79
131.12
130.84
130.80
128.41
127.93
49.00 CD3OD
ppm

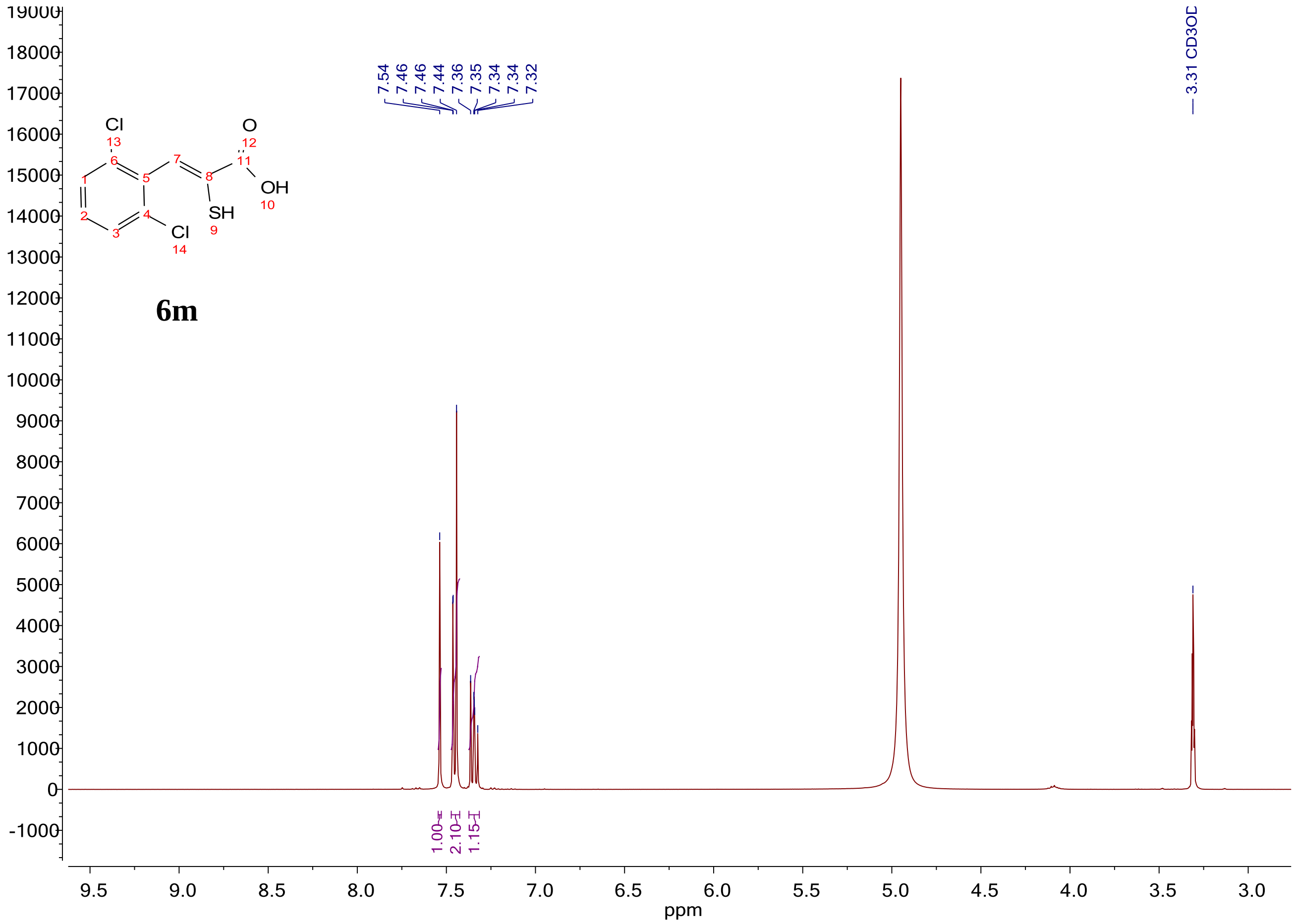
7.54
7.46
7.46
7.44
7.36
7.35
7.34
7.34
7.32
3.31 CD3OD
Cl
13
6
O
12
11
7
5
8
1
OH
10
2
4
SH
9
3
Cl
14
6m
1.00
2.10
1.15
ppm

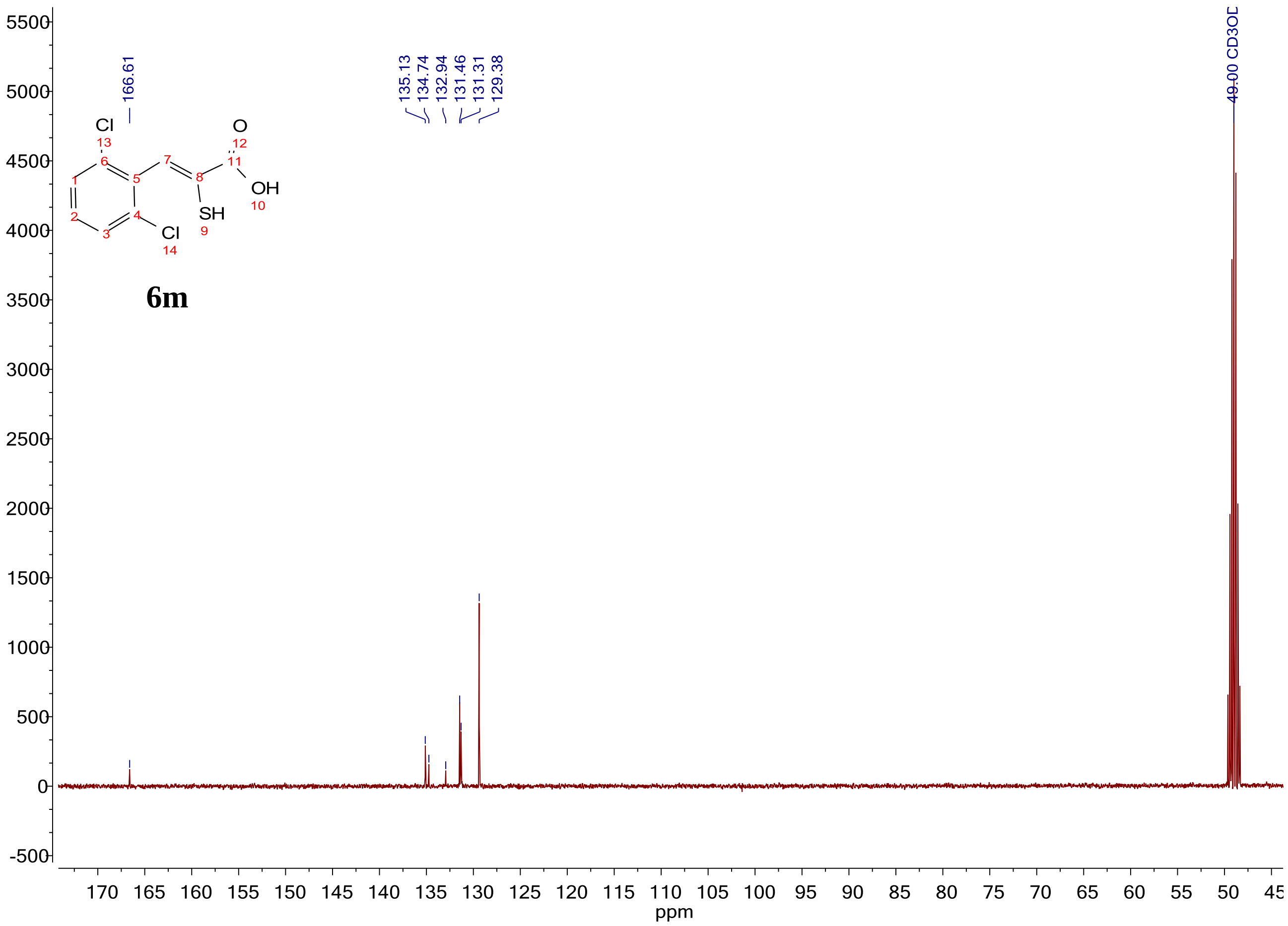
166.61
135.13
134.74
132.94
131.46
131.31
129.38
49.00 CD3OD
Cl
13
6
O
12
11
7
5
8
1
OH
10
2
4
SH
9
3
Cl
14
6m
ppm

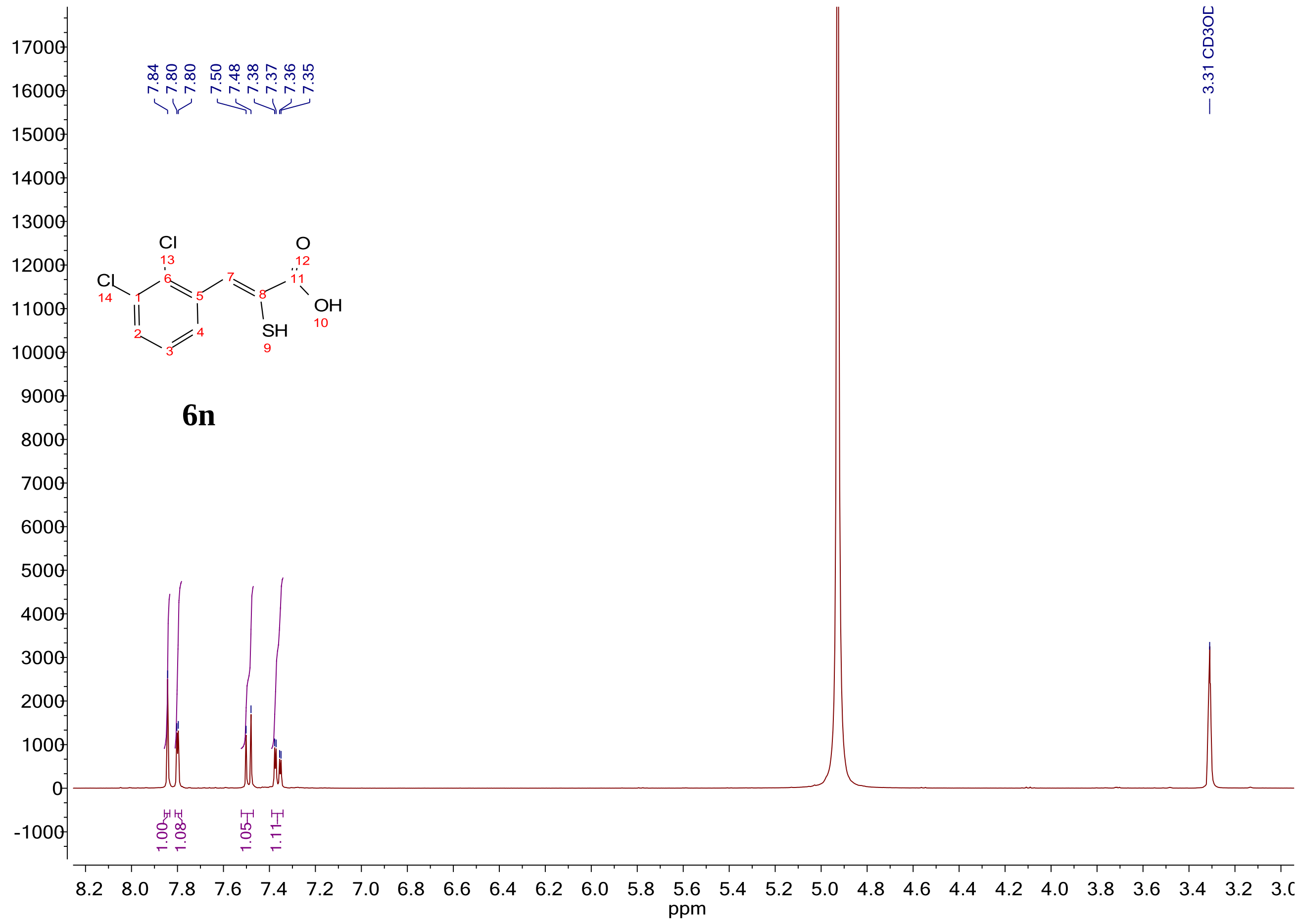
7.84
7.80
7.80
7.50
7.48
7.38
7.37
7.36
7.35
3.31 CD3OD
Cl
13
O
12
Cl
14
6
7
11
1
5
8
OH
10
2
4
SH
9
3
6n
1.00
1.08
1.05
1.11
ppm

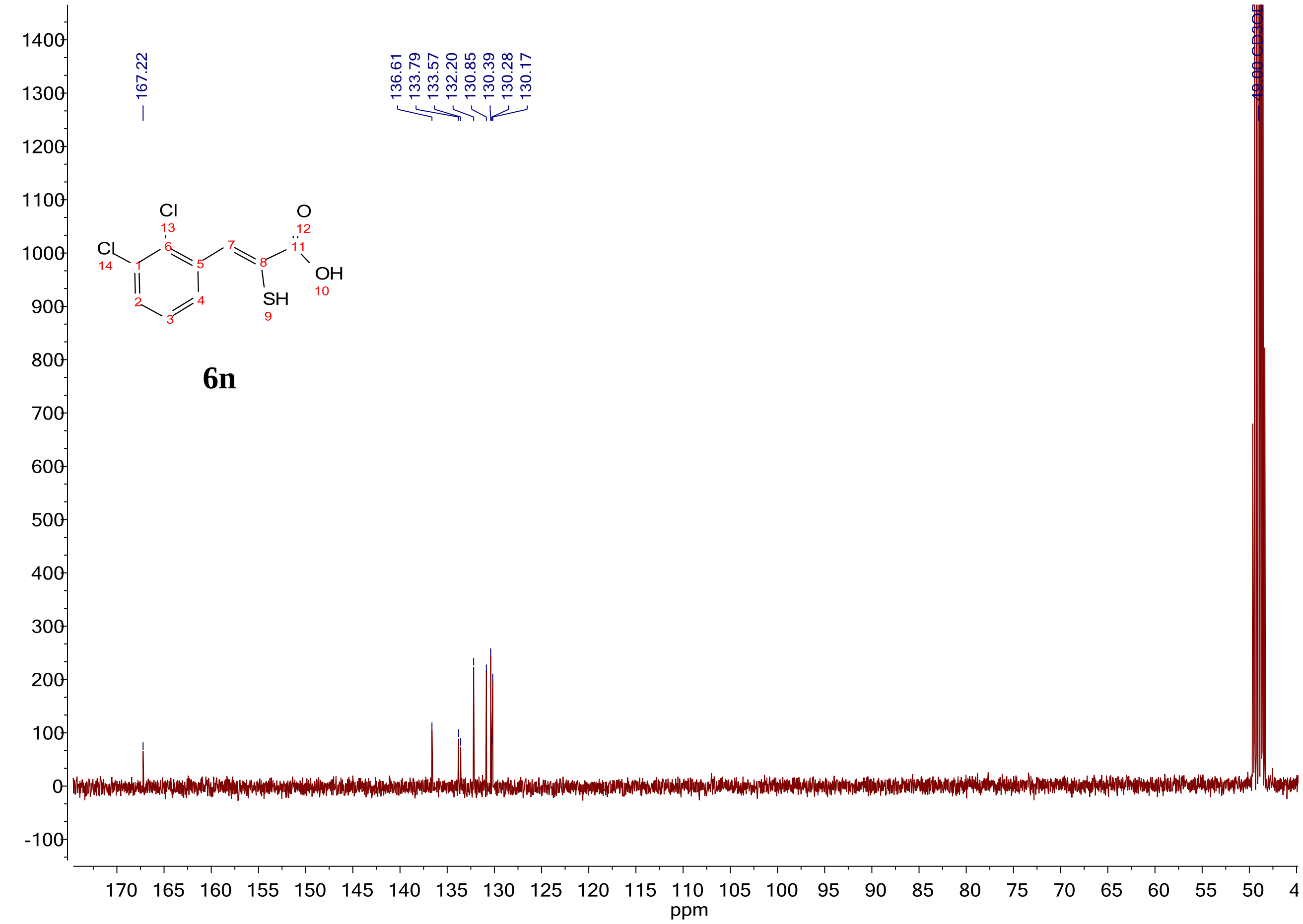
167.22
136.61
133.79
133.57
132.20
130.85
130.39
130.28
130.17
49.00 CD3OD
6n
ppm

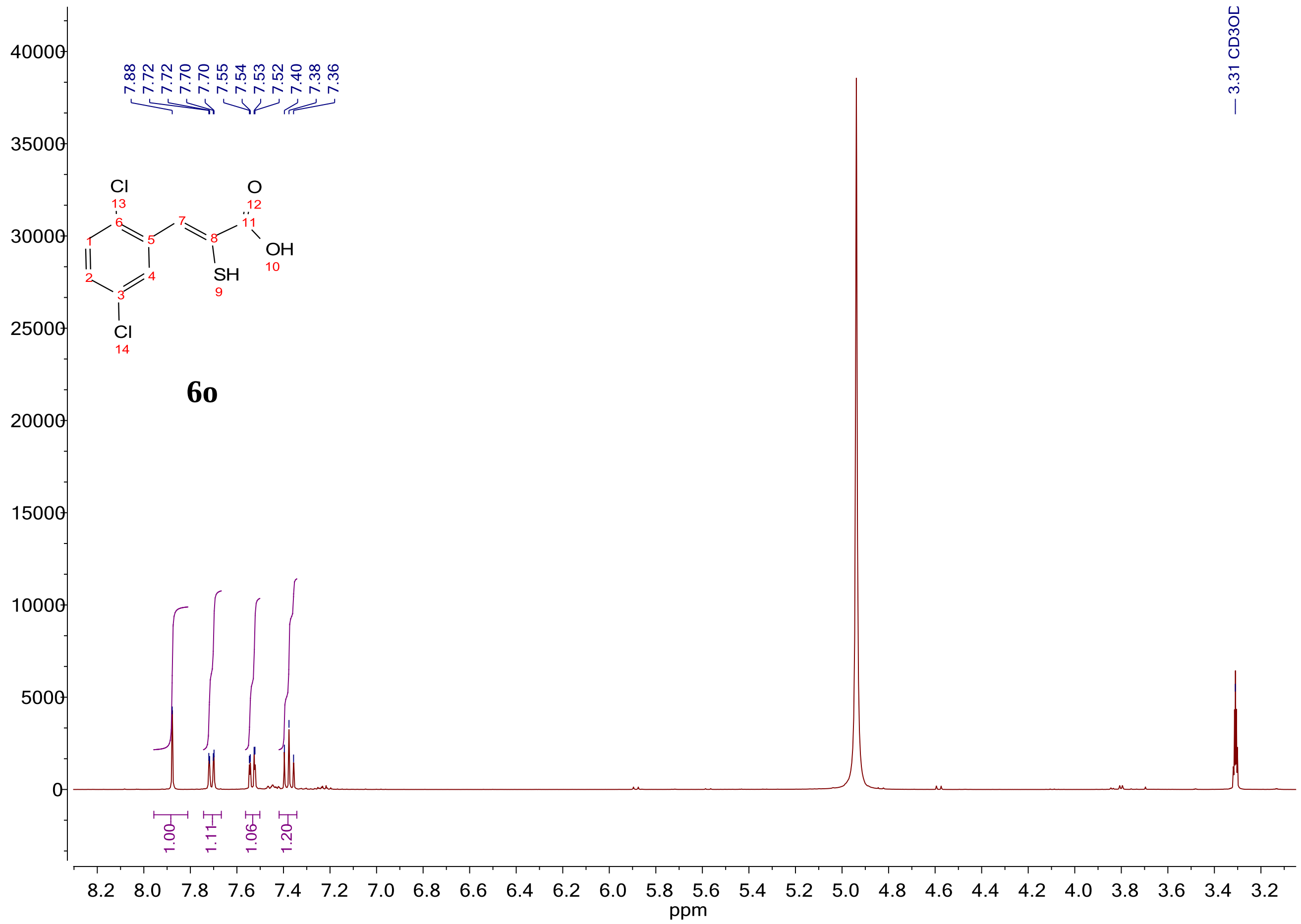
7.88
7.72
7.72
7.70
7.70
7.55
7.54
7.53
7.52
7.40
7.38
7.36
3.31 CD3OD
6o
1.00
1.11
1.06
1.20
ppm

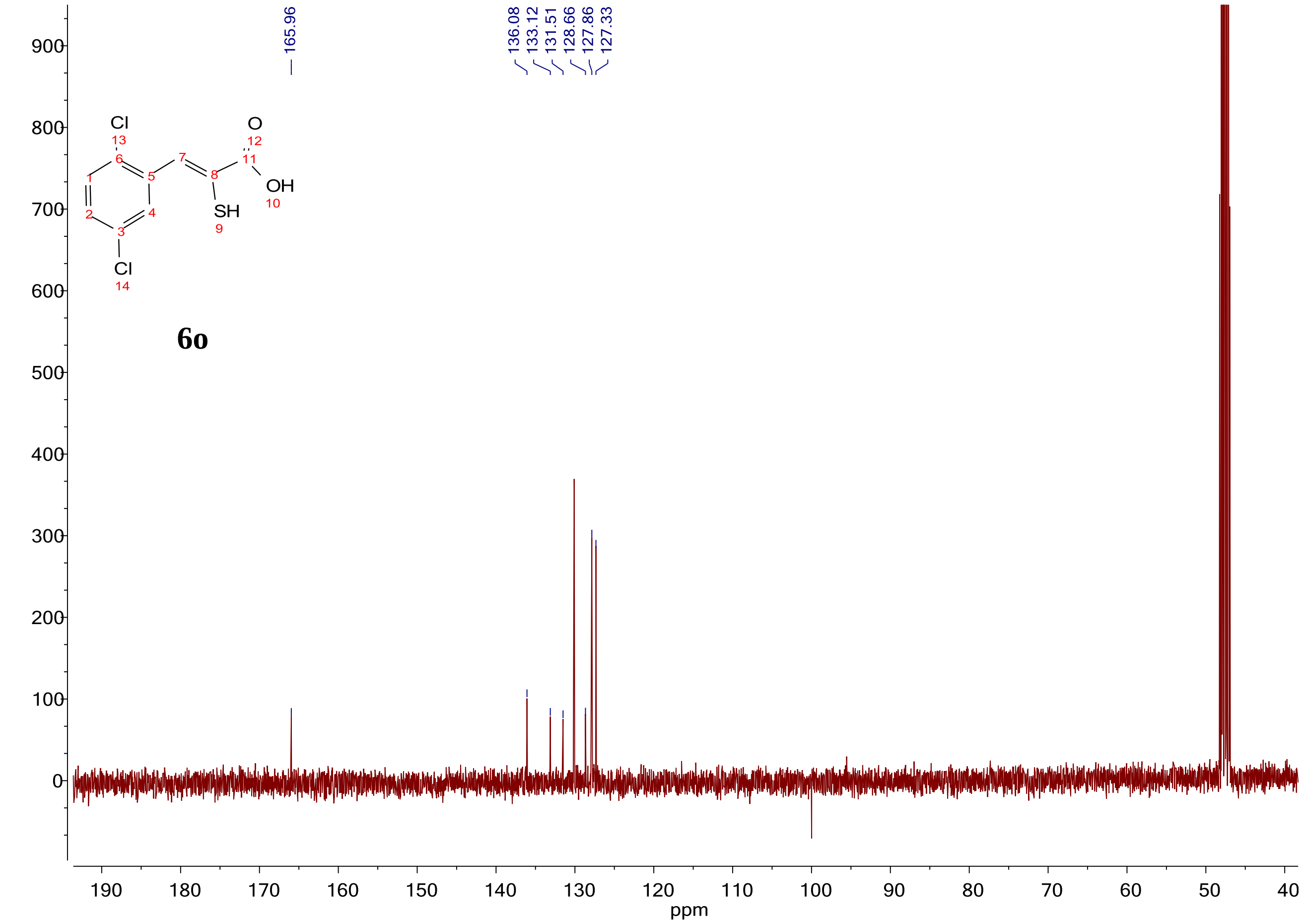
165.96
136.08
133.12
131.51
128.66
127.86
127.33
6o
ppm

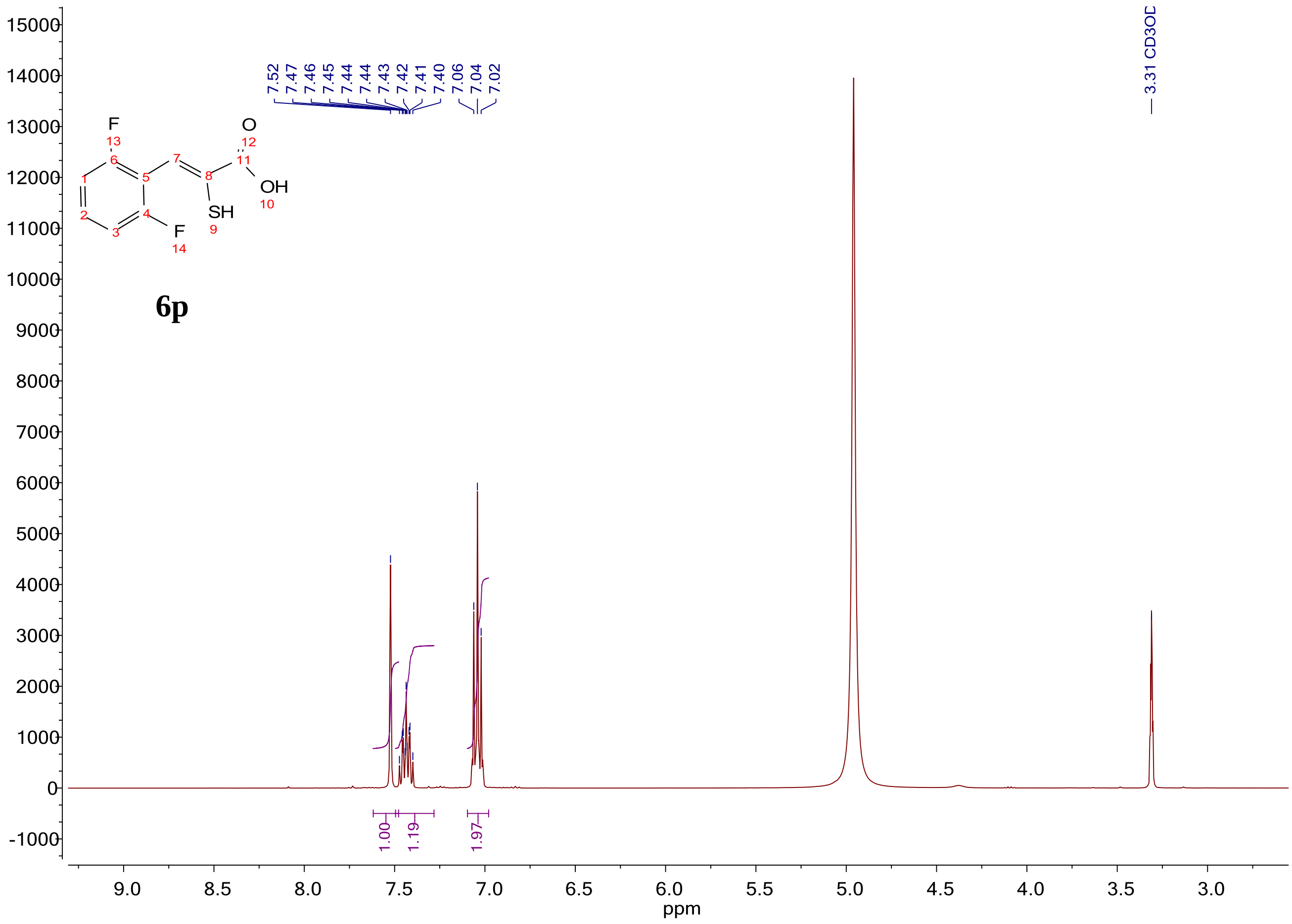
6p
7.52
7.47
7.46
7.45
7.44
7.44
7.43
7.42
7.41
7.40
7.06
7.04
7.02
3.31 CD3OD
1.00
1.19
1.97
ppm

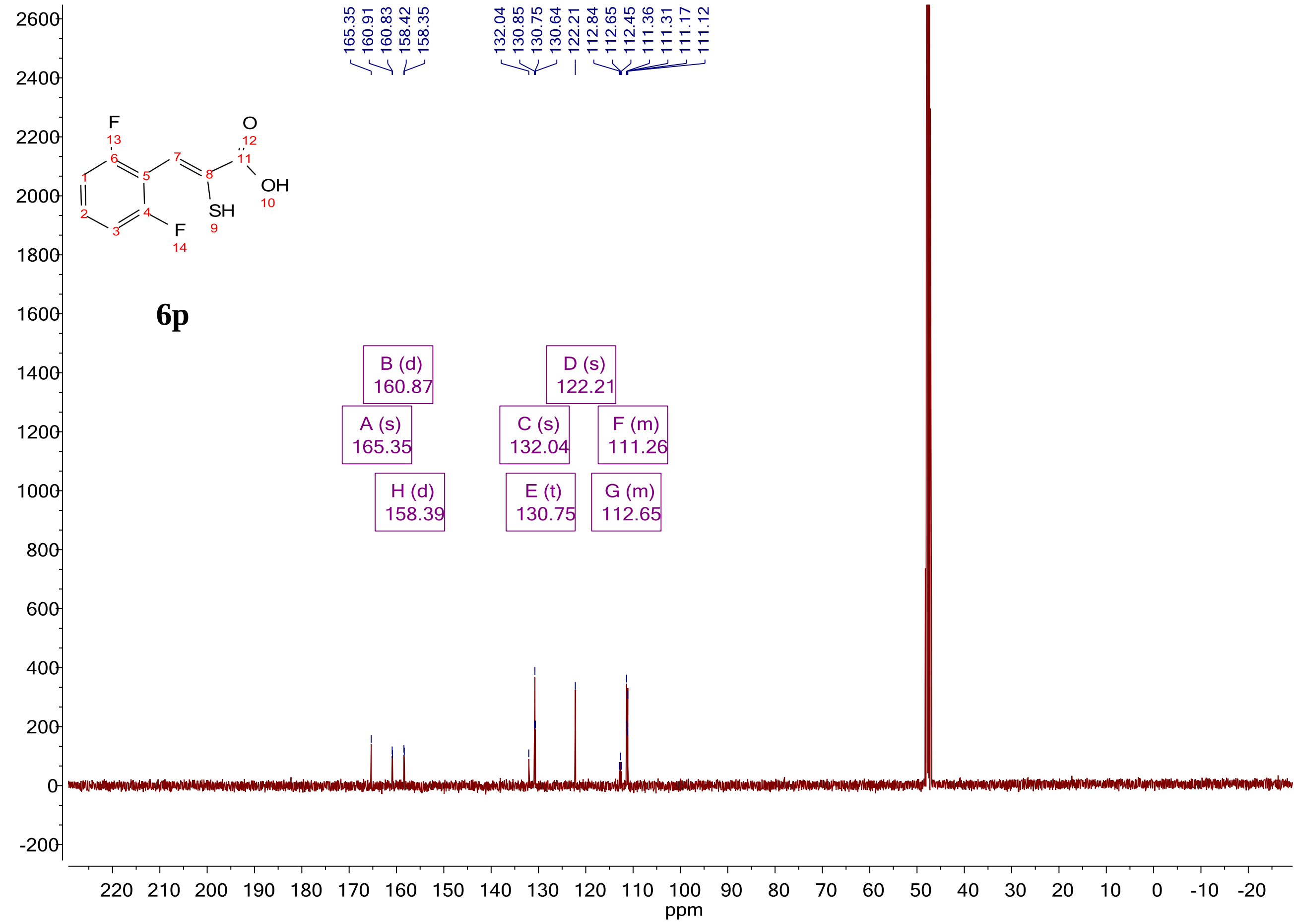
6p
165.35
160.91
160.83
158.42
158.35
132.04
130.85
130.75
130.64
122.21
112.84
112.65
112.45
111.36
111.31
111.17
111.12
A (s) 165.35
B (d) 160.87
H (d) 158.39
C (s) 132.04
E (t) 130.75
D (s) 122.21
F (m) 111.26
G (m) 112.65
ppm

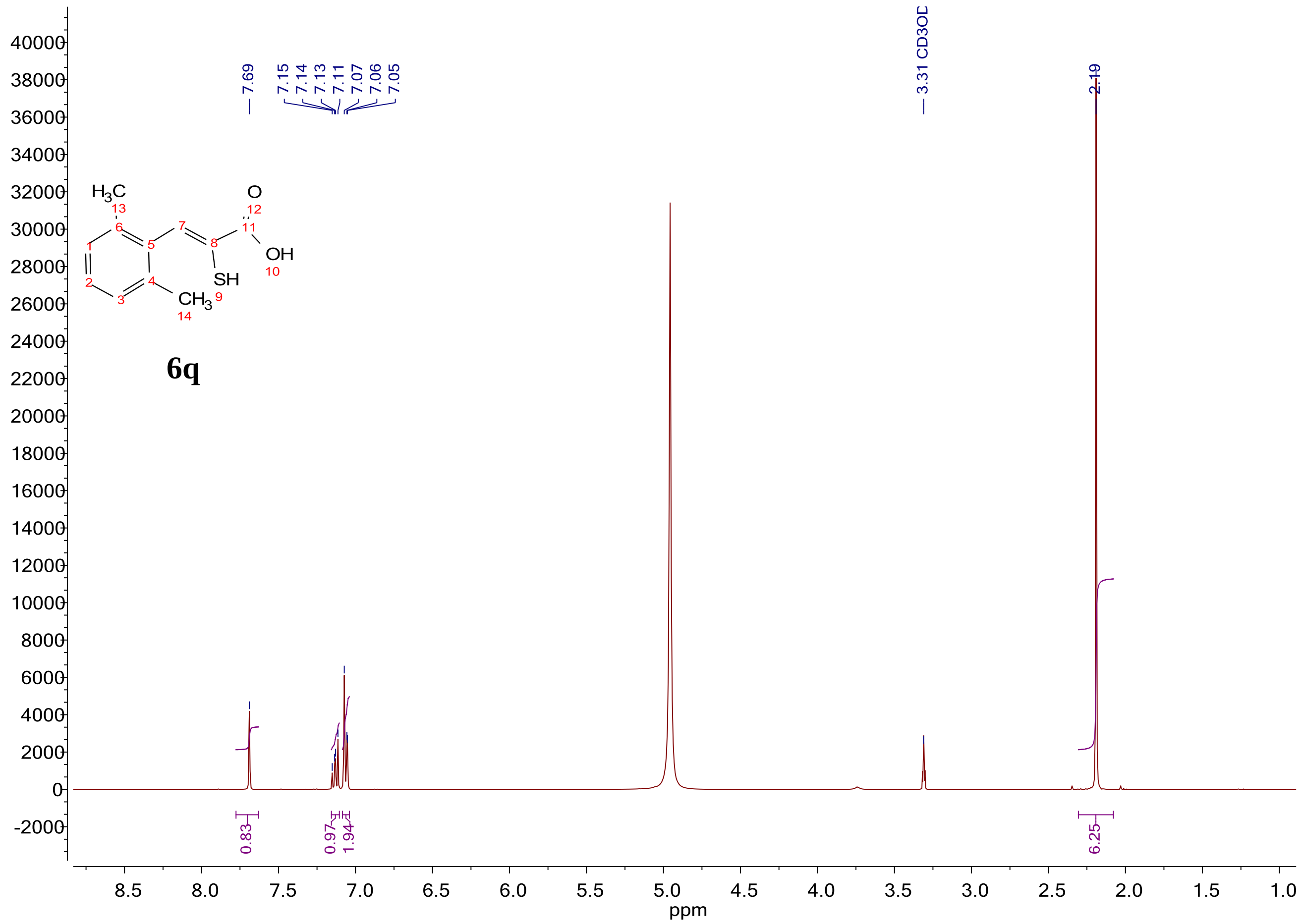
7.69
7.15
7.14
7.13
7.11
7.07
7.06
7.05
3.31 CD3OD
2.19
0.83
0.97
1.94
6.25
6q
ppm

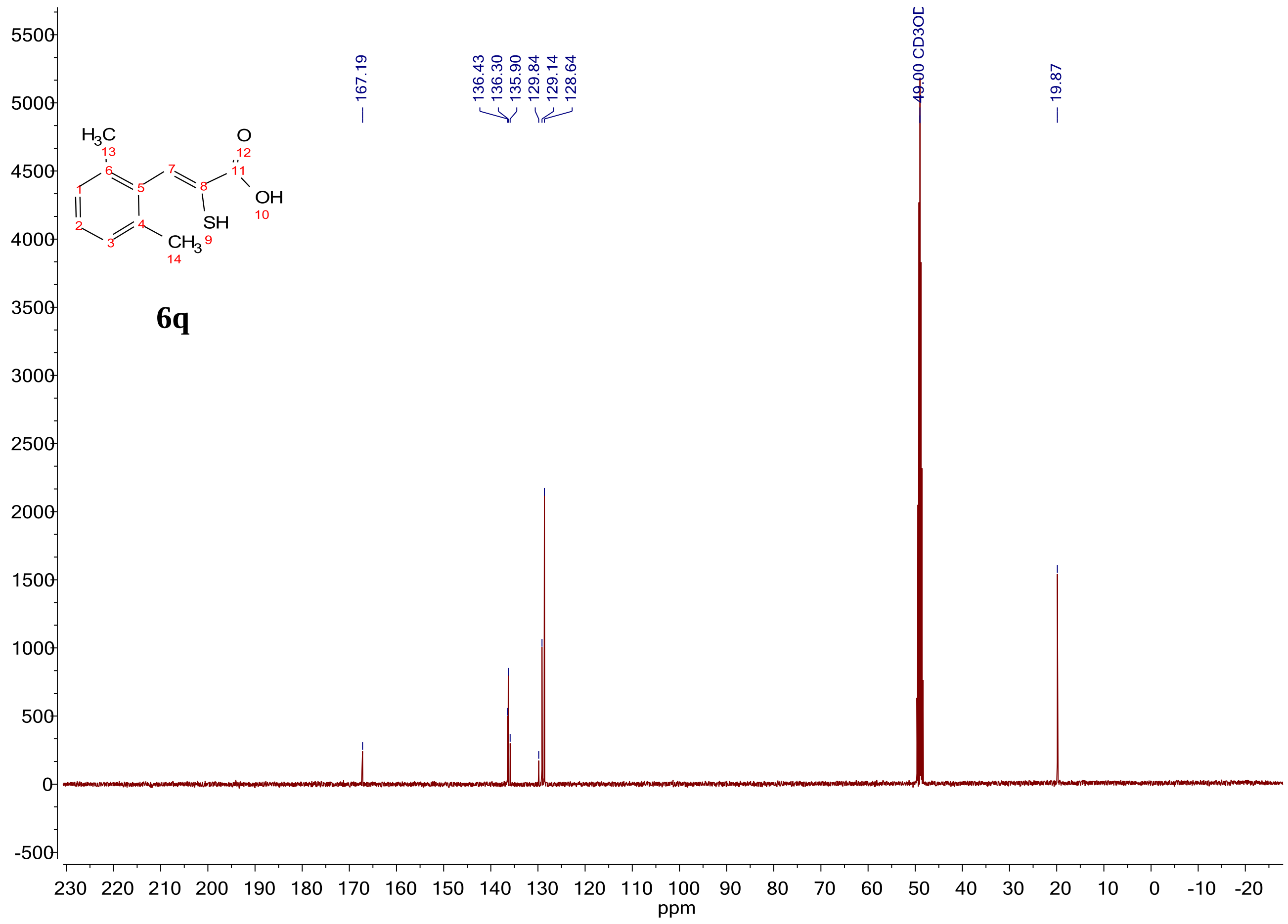
167.19
136.43
136.30
135.90
129.84
129.14
128.64
49.00 CD3OD
19.87
6q
ppm

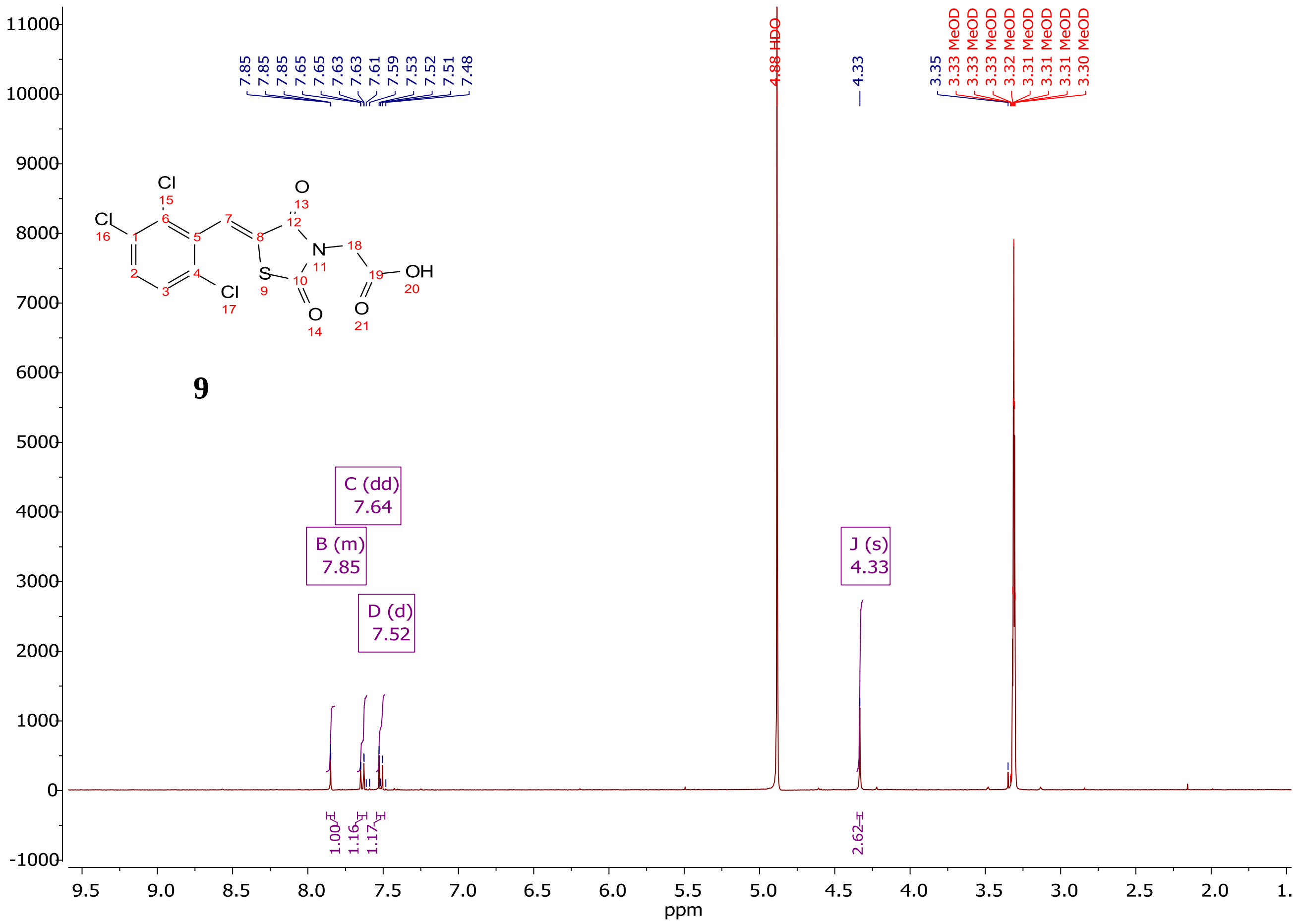
7.85
7.85
7.85
7.65
7.65
7.63
7.63
7.61
7.59
7.53
7.52
7.51
7.48
4.88 HDO
4.33
3.35
3.33 MeOD
3.33 MeOD
3.33 MeOD
3.32 MeOD
3.31 MeOD
3.31 MeOD
3.31 MeOD
3.30 MeOD
9
C (dd)
7.64
B (m)
7.85
J (s)
4.33
D (d)
7.52
1.00
1.16
1.17
2.62
ppm

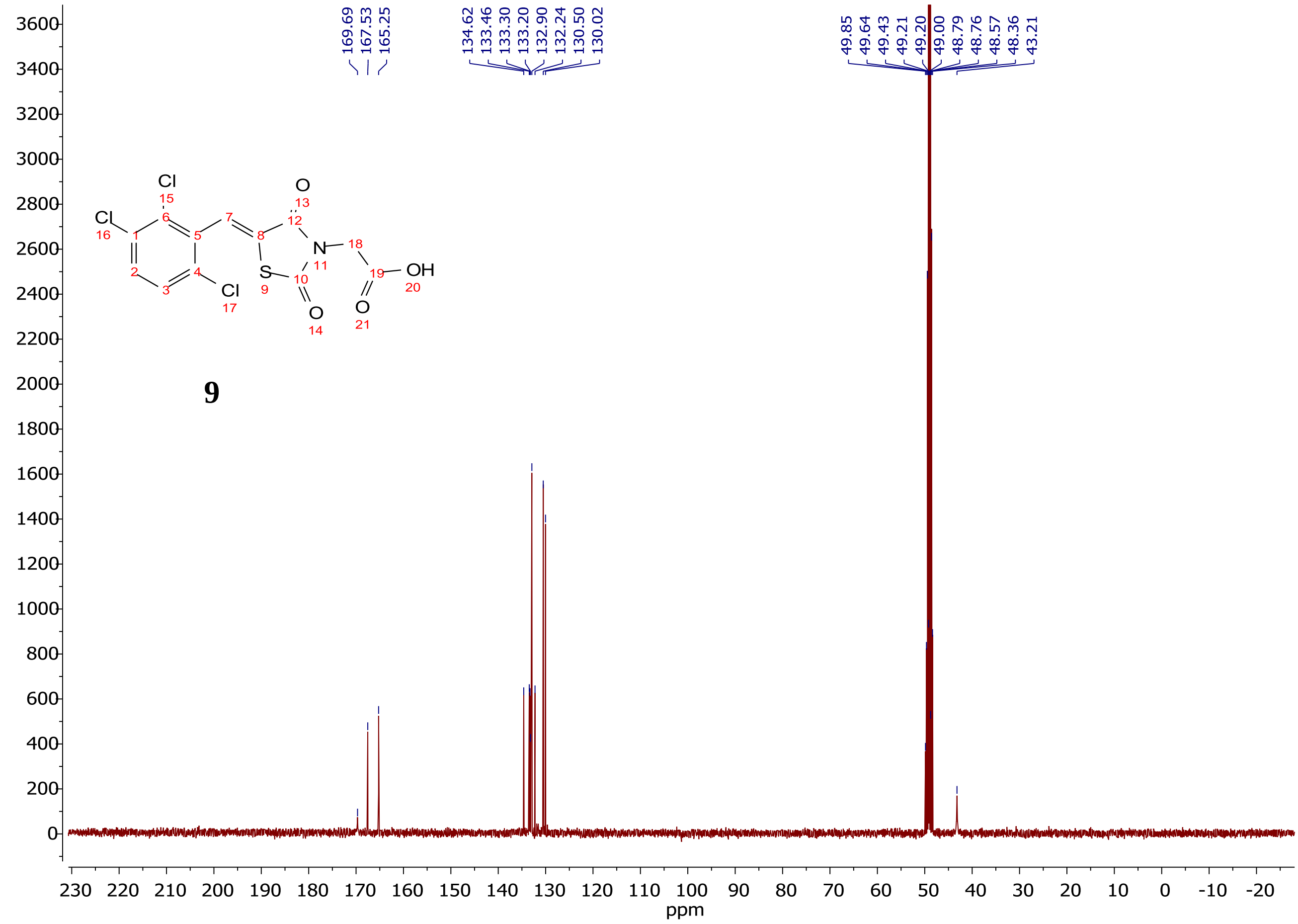
169.69
167.53
165.25
134.62
133.46
133.30
133.20
132.90
132.24
130.50
130.02
49.85
49.64
49.43
49.21
49.20
49.00
48.79
48.76
48.57
48.36
43.21
9
ppm

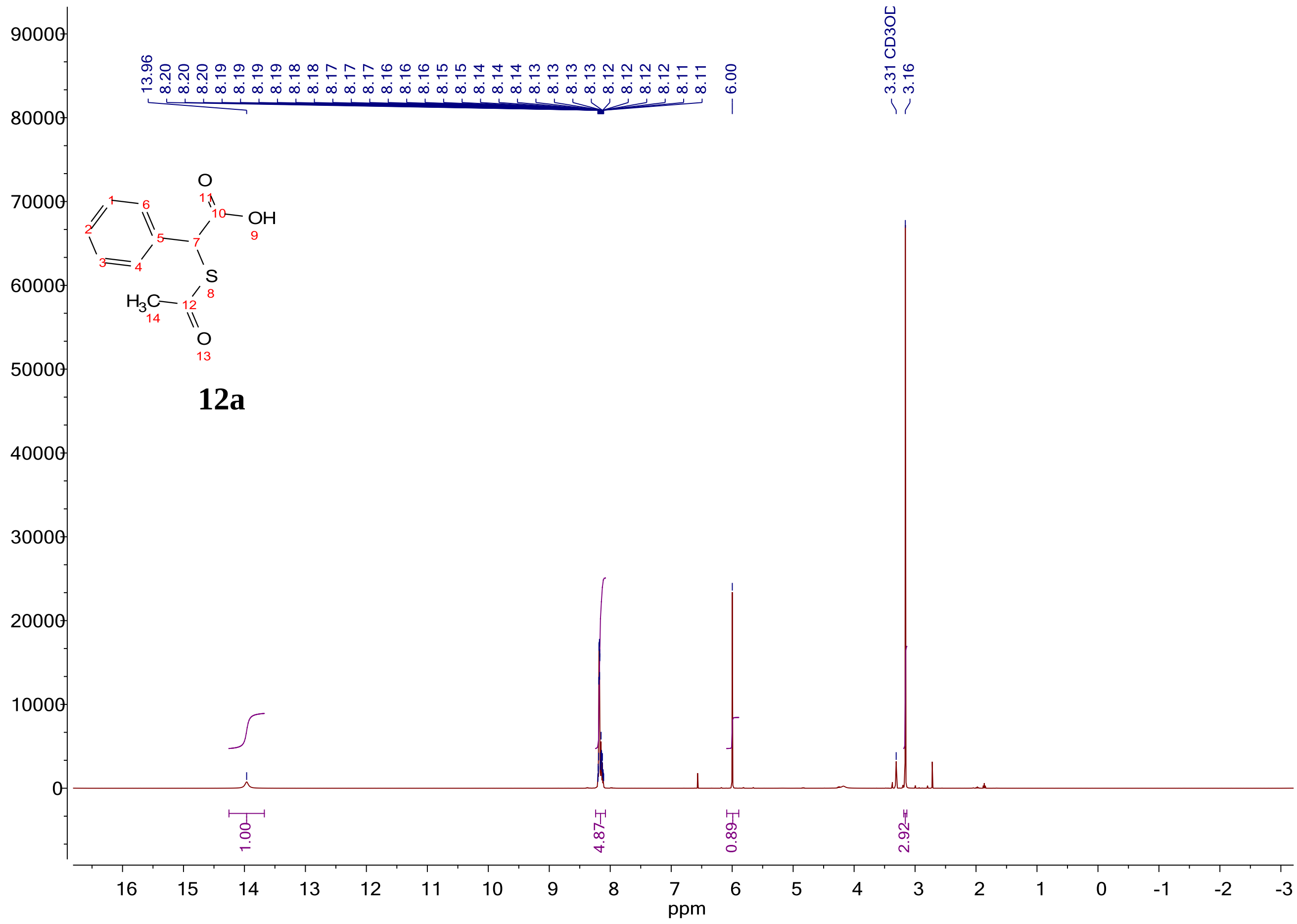

13.96
8.20
8.20
8.20
8.19
8.19
8.19
8.18
8.18
8.17
8.17
8.17
8.16
8.16
8.16
8.15
8.15
8.14
8.14
8.14
8.13
8.13
8.13
8.13
8.12
8.12
8.12
8.12
8.11
8.11
6.00
3.31 CD3OD
3.16
O
OH
S
H_3C
O
12a
1.00
4.87
0.89
2.92
ppm

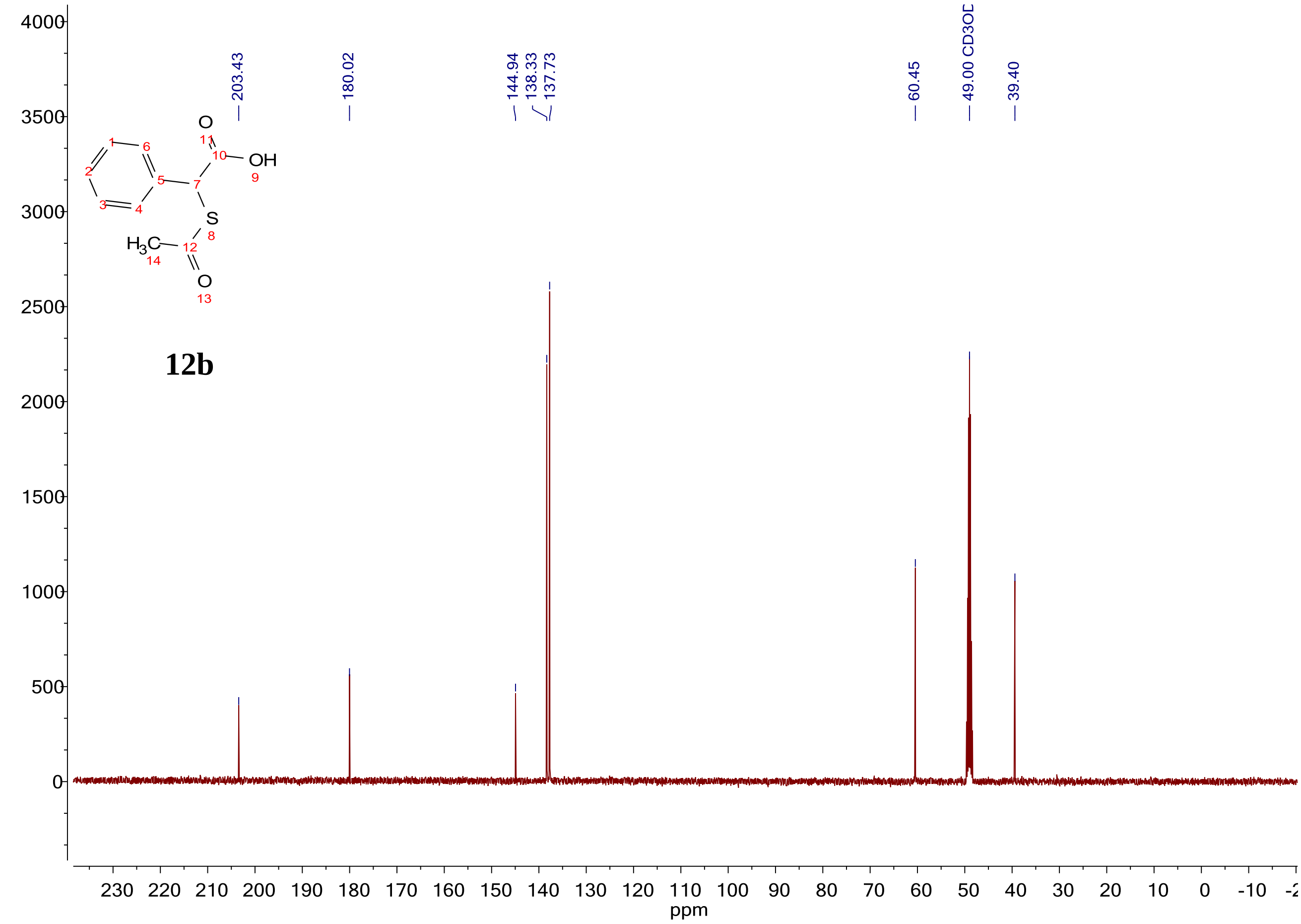

203.43
180.02
144.94
138.33
137.73
60.45
49.00 CD3OD
39.40
O
OH
S
H_3C
O
12b
ppm

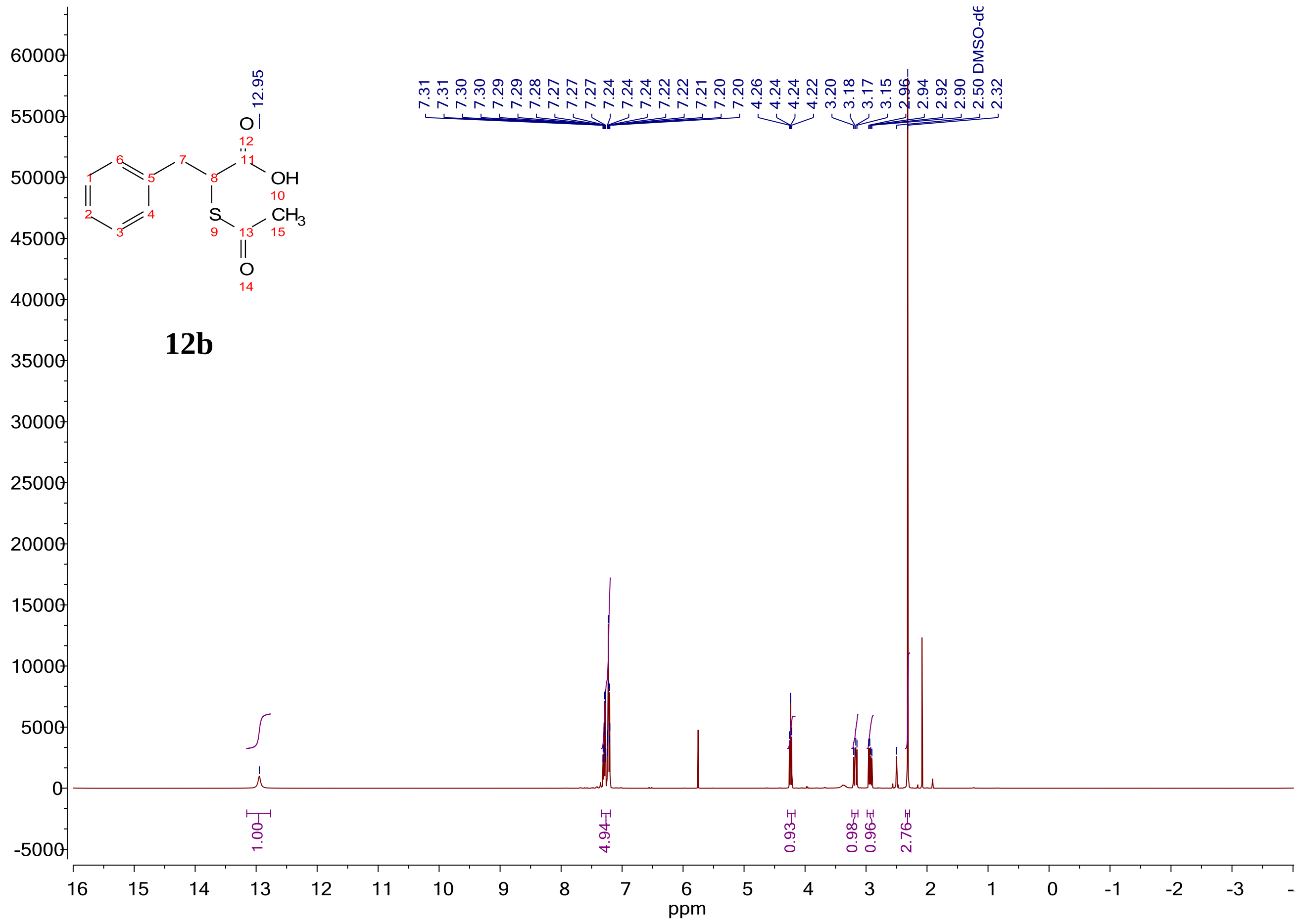

12.95
7.31 7.31 7.30 7.29 7.29 7.28 7.27 7.27 7.27 7.24 7.24 7.24 7.24 7.22 7.22 7.21 7.20 7.20
4.26 4.24 4.24 4.22
3.20 3.18 3.17 3.15 2.96 2.94 2.92 2.90 2.50 DMSO-d6 2.32
12b
1.00
4.94
0.93
0.98
0.96
2.76
ppm

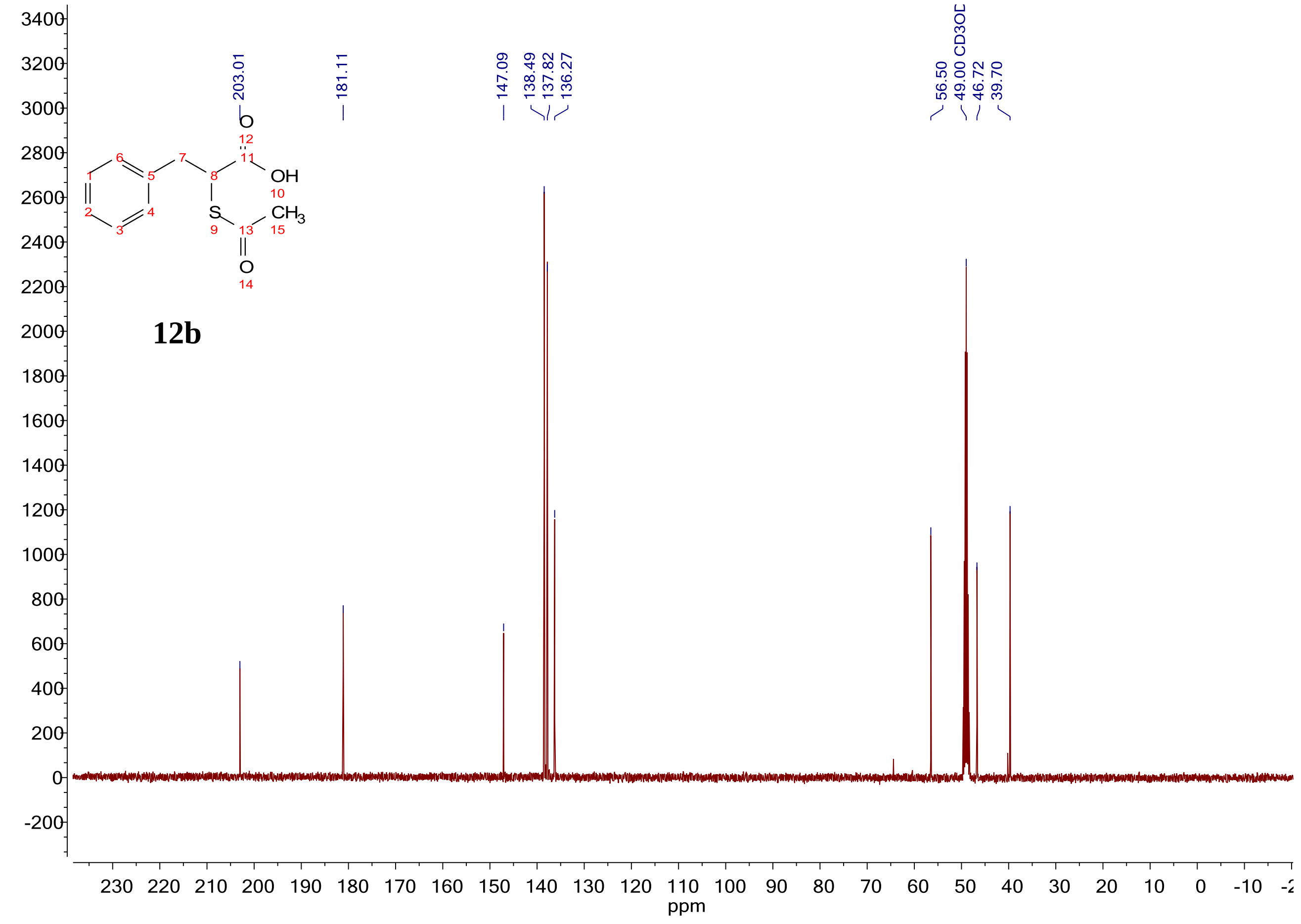

203.01
181.11
147.09 138.49 137.82 136.27
56.50 49.00 CD3OD 46.72 39.70
12b
ppm

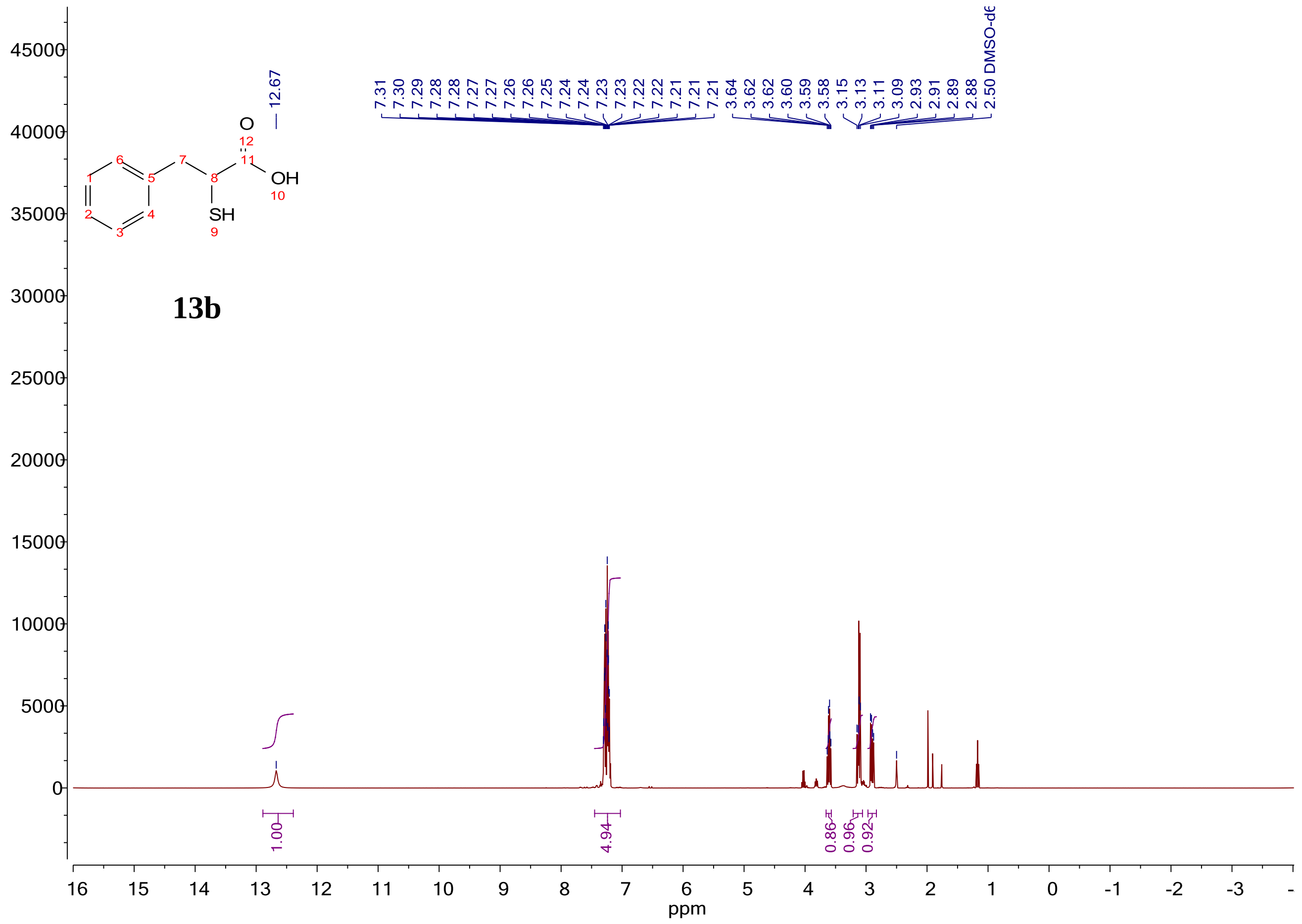

13b

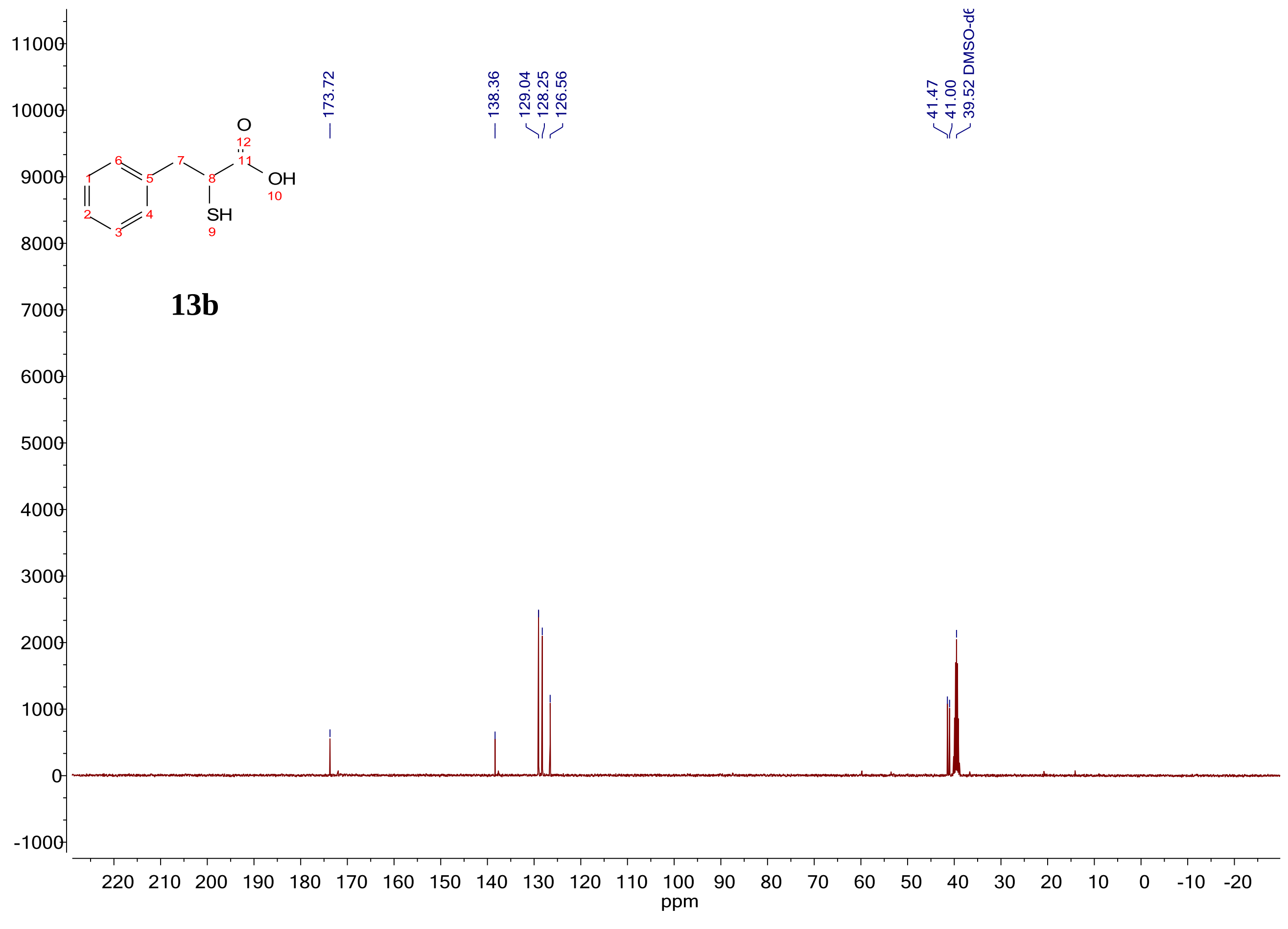

13b

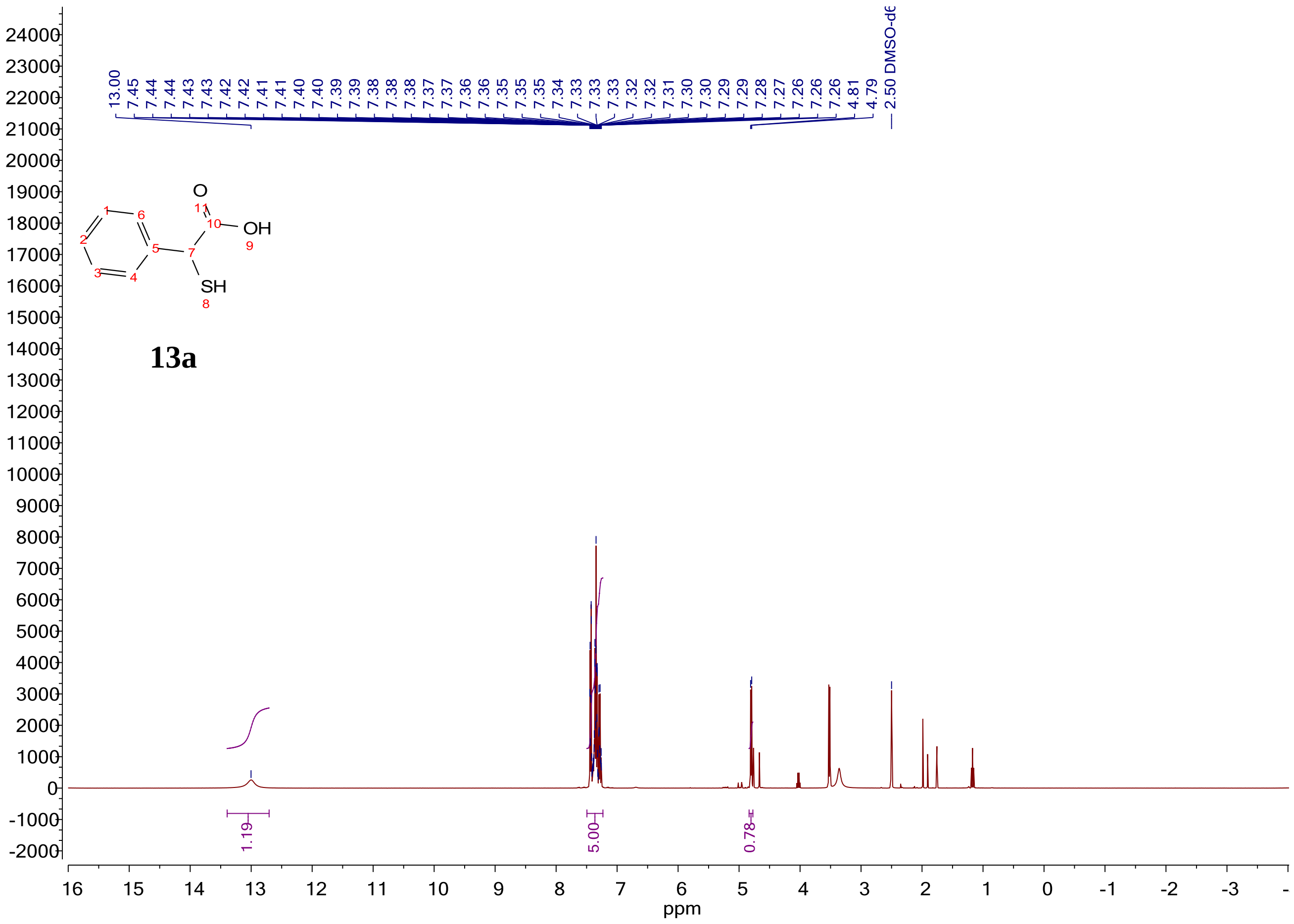

13.00
7.45
7.44
7.44
7.43
7.43
7.42
7.42
7.41
7.41
7.40
7.40
7.39
7.39
7.38
7.38
7.38
7.37
7.37
7.36
7.36
7.35
7.35
7.35
7.34
7.33
7.33
7.33
7.32
7.32
7.31
7.30
7.30
7.29
7.29
7.28
7.27
7.26
7.26
7.26
4.81
4.79
2.50 DMSO-d6
1.19
5.00
0.78
ppm

13a

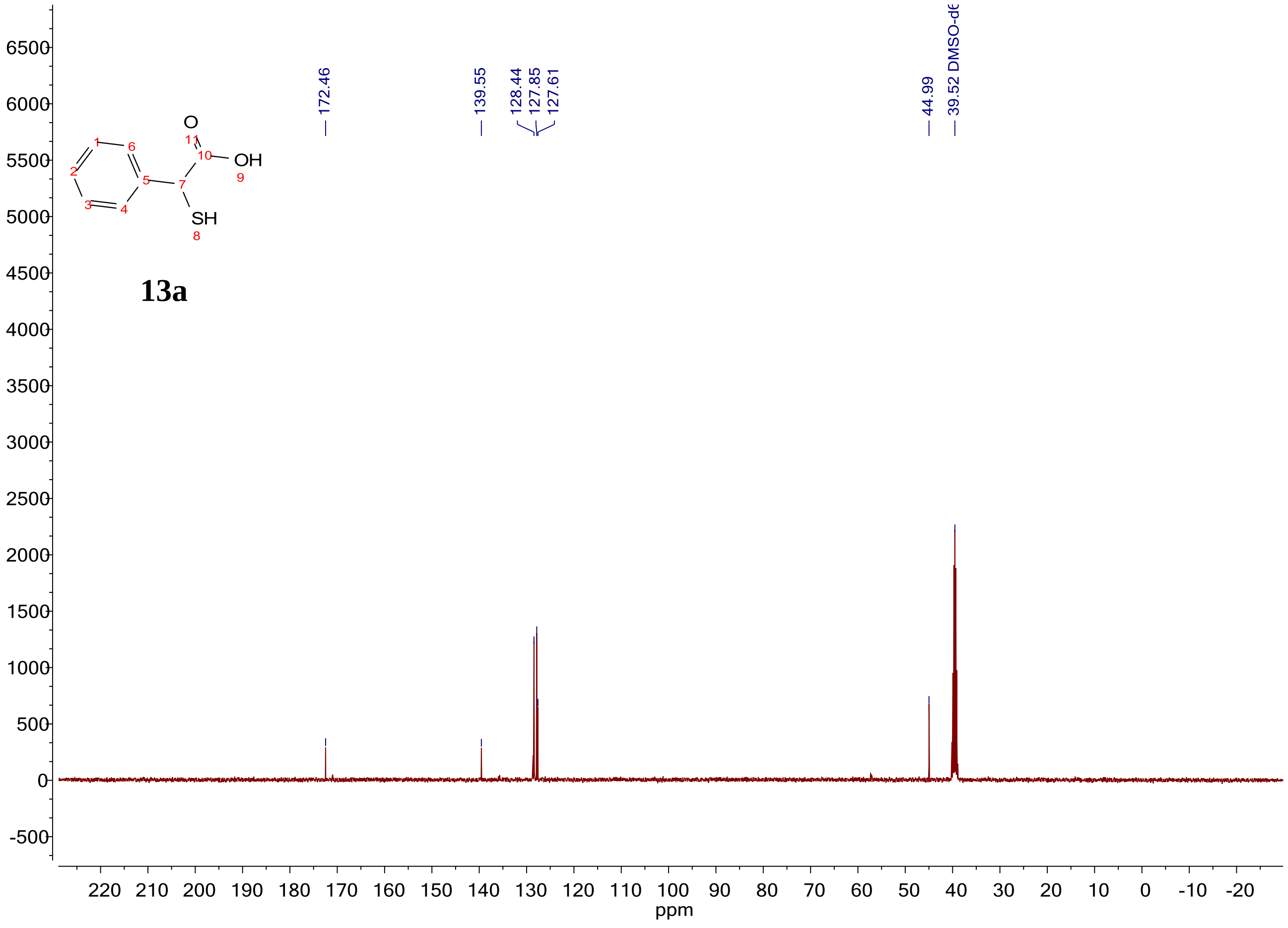

172.46
139.55
128.44
127.85
127.61
44.99
39.52 DMSO-d6
ppm

13a

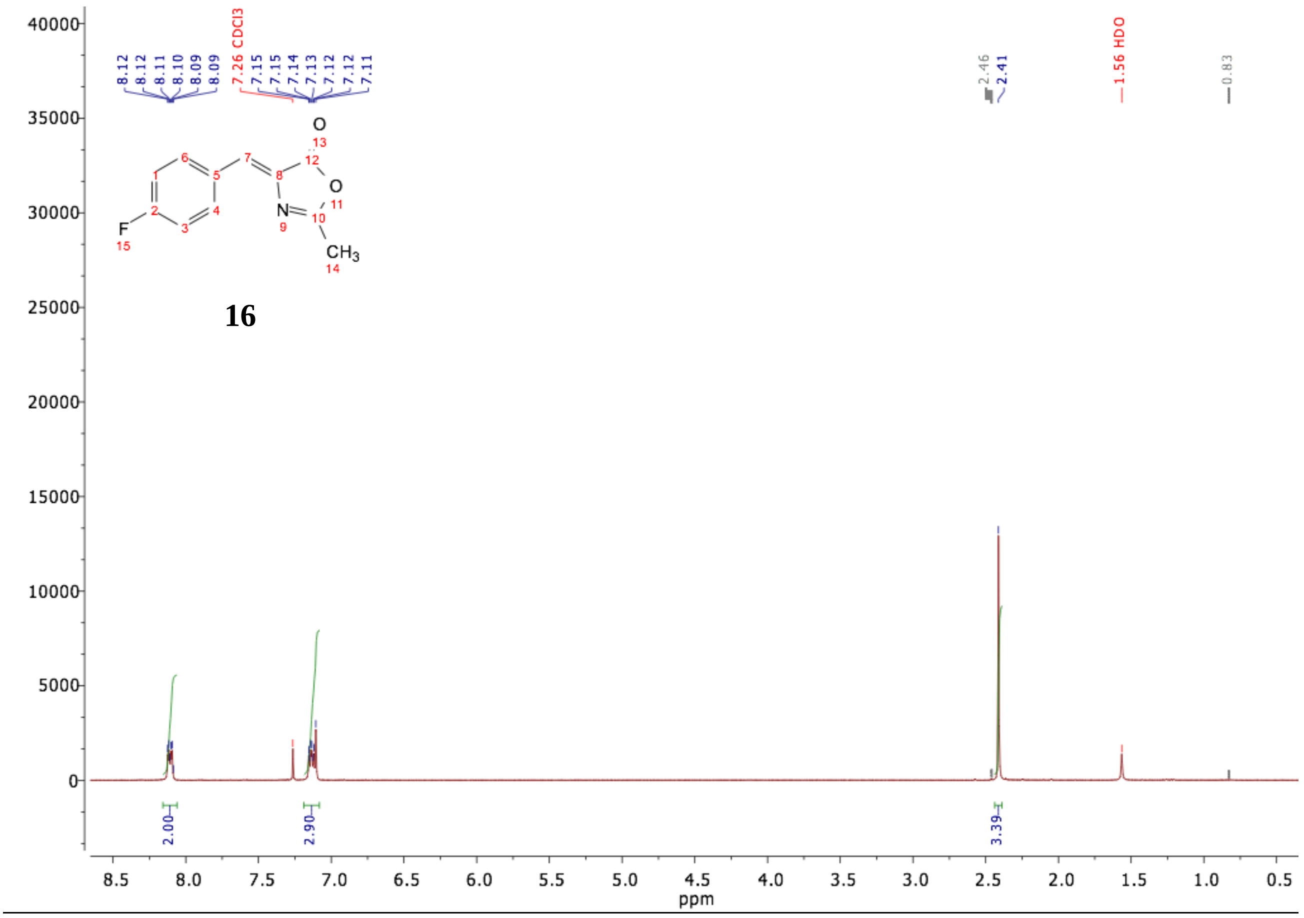
8.12
8.12
8.11
8.10
8.09
8.09
7.26 CDCl3
7.15
7.15
7.14
7.13
7.12
7.12
7.11
2.46
2.41
1.56 HDO
0.83
2.00
2.90
3.39
ppm

16

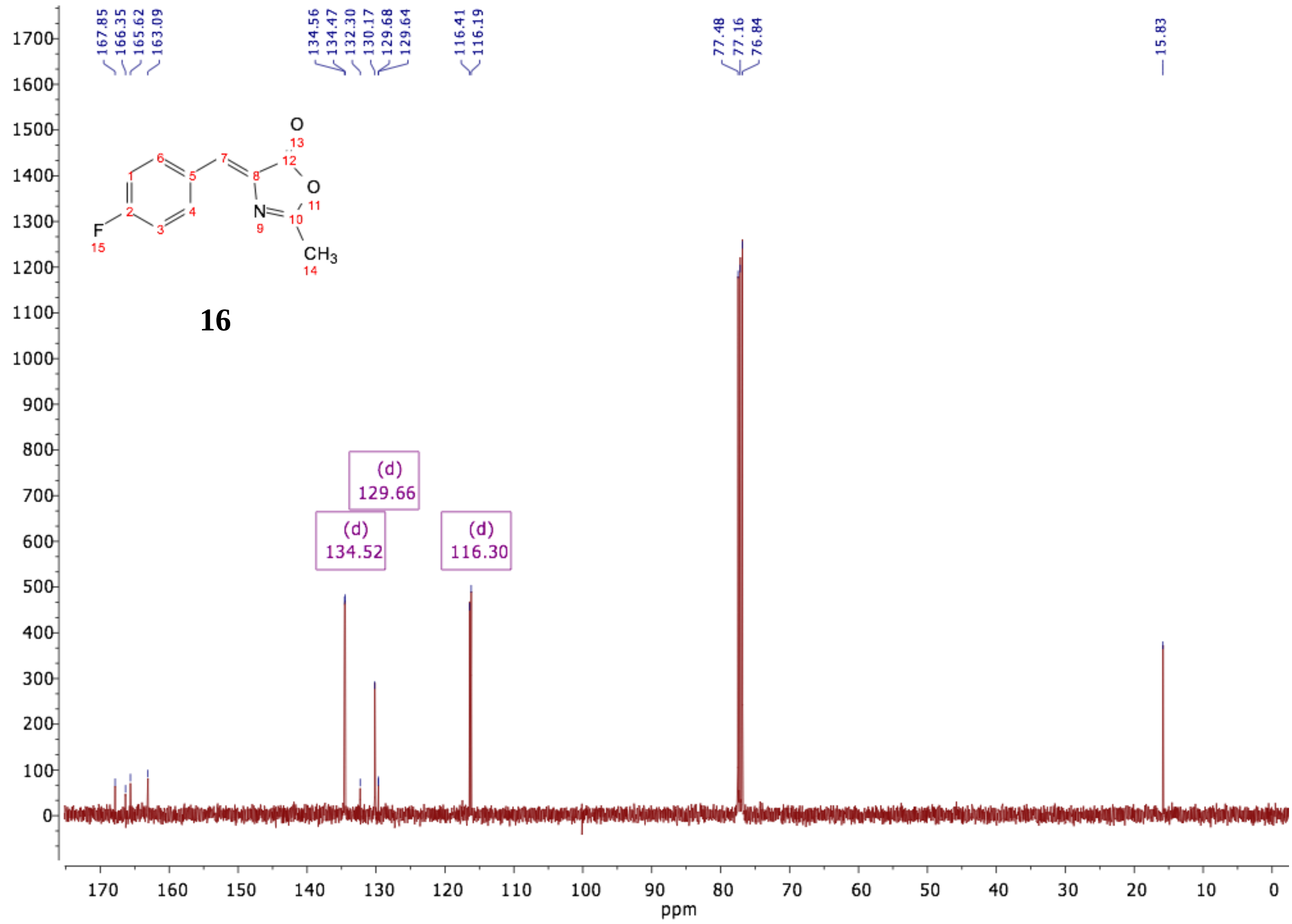
167.85
166.35
165.62
163.09
134.56
134.47
132.30
130.17
129.68
129.64
116.41
116.19
77.48
77.16
76.84
15.83
(d)
129.66
(d)
134.52
(d)
116.30
ppm

16

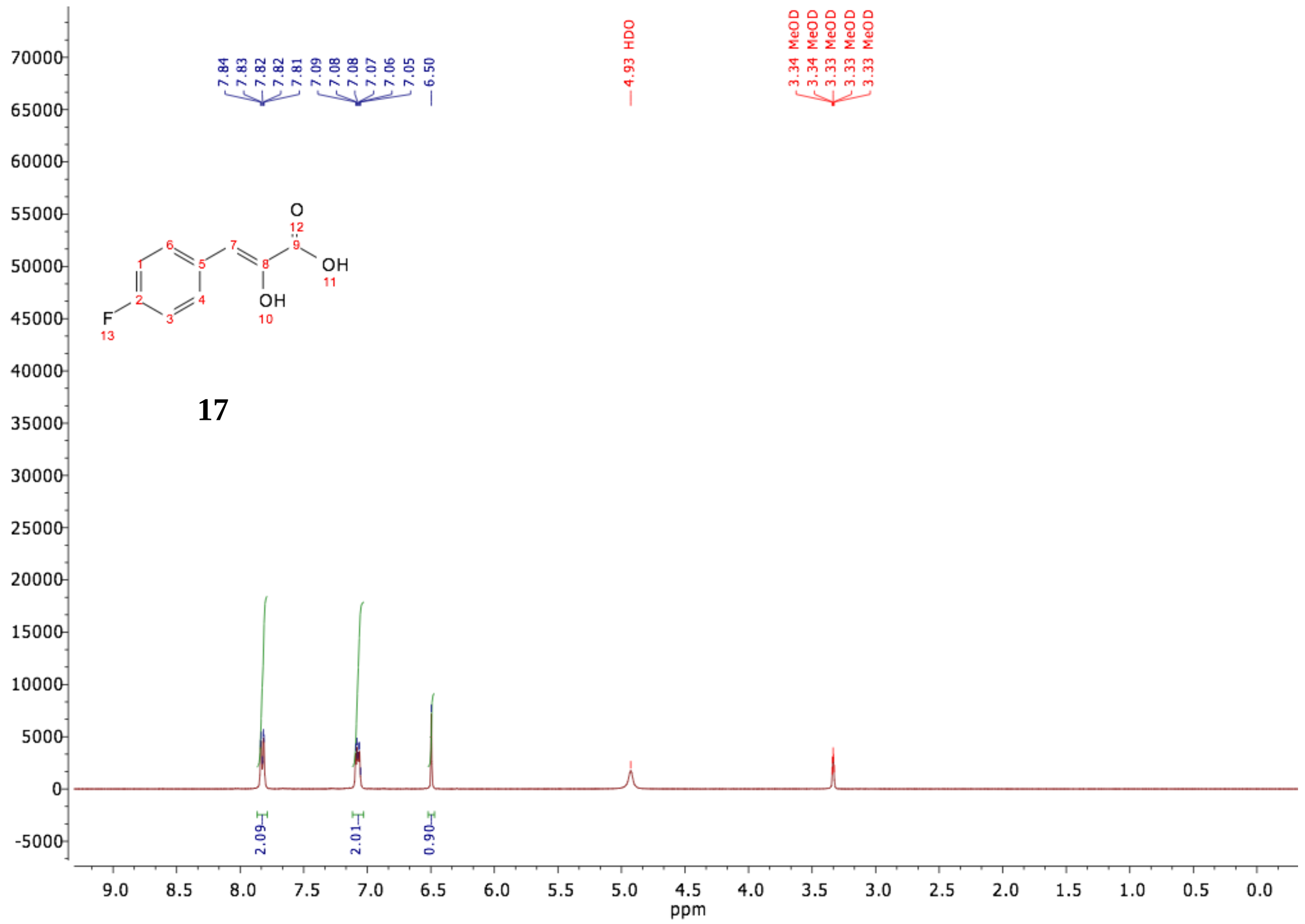
7.84 7.83 7.82 7.82 7.81 7.09 7.08 7.08 7.07 7.06 7.05 6.50
4.93 HDO
3.34 MeOD 3.34 MeOD 3.33 MeOD 3.33 MeOD 3.33 MeOD
17
2.09
2.01
0.90
ppm

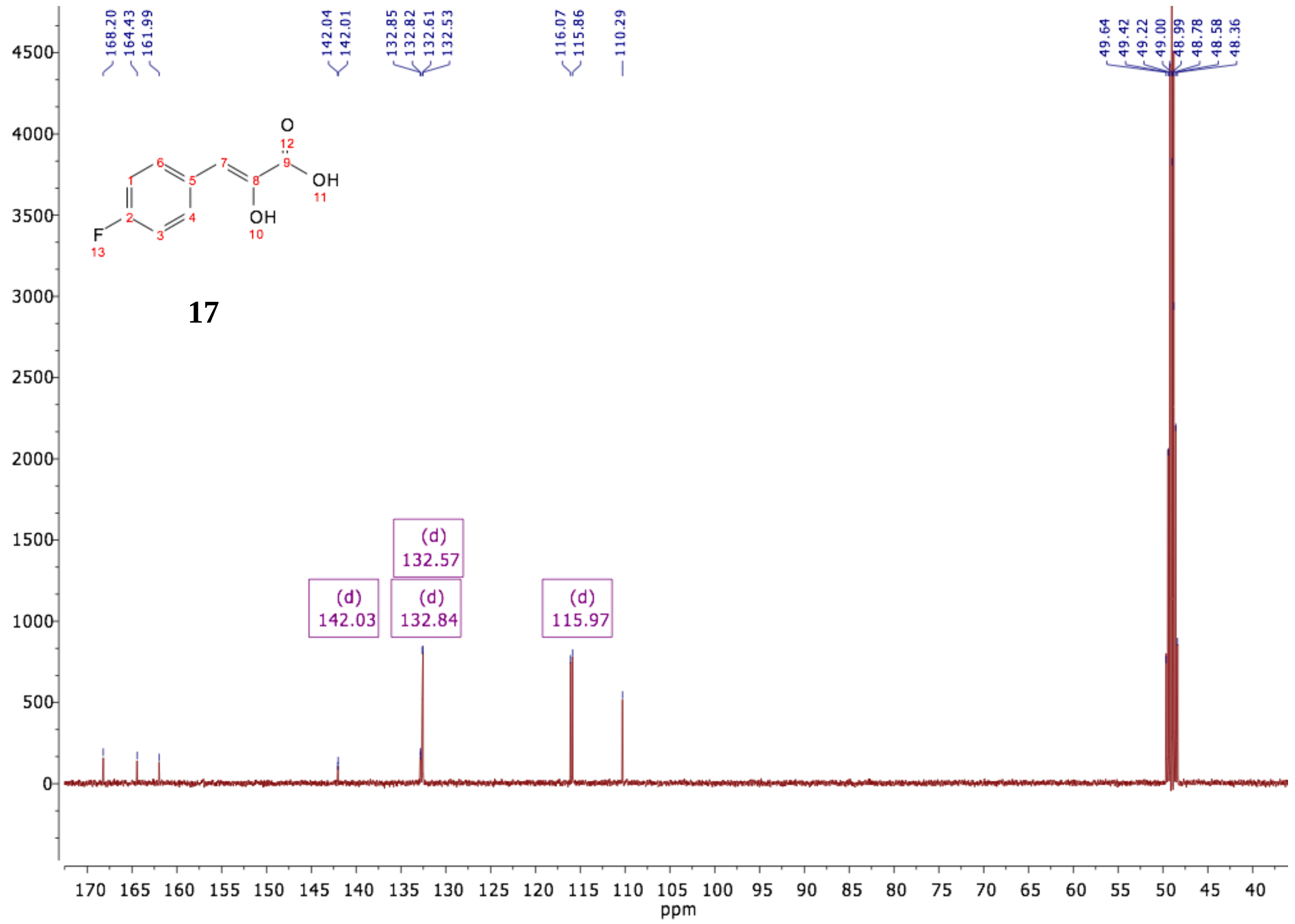
168.20 164.43 161.99
142.04 142.01
132.85 132.82 132.61 132.53
116.07 115.86
110.29
49.64 49.42 49.22 49.00 48.99 48.78 48.58 48.36
17
(d) 132.57
(d) 142.03
(d) 132.84
(d) 115.97
ppm

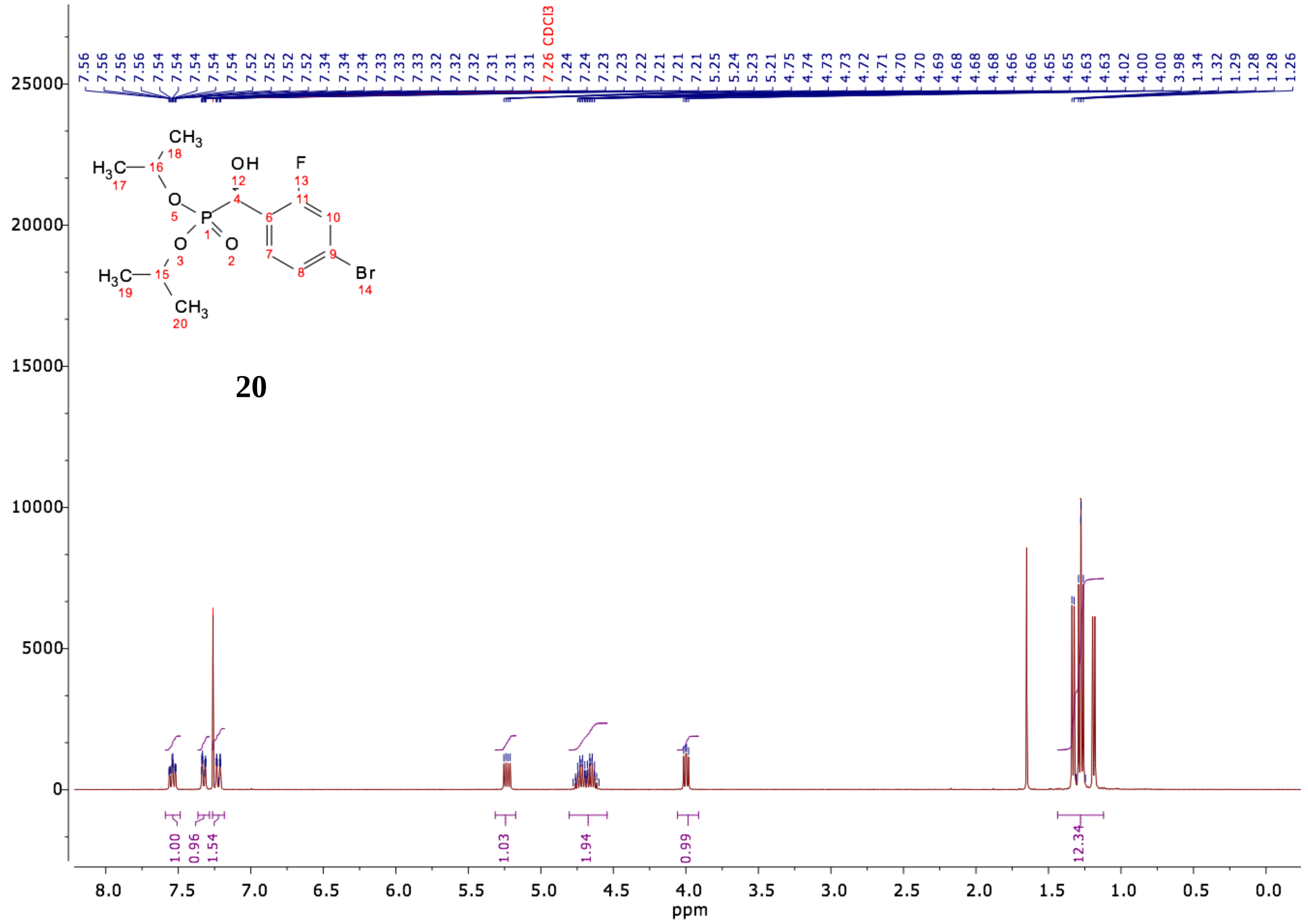
7.56 7.56 7.56 7.54 7.54 7.54 7.54 7.54 7.52 7.52 7.52 7.52 7.34 7.34 7.34 7.33 7.33 7.33 7.32 7.32 7.32 7.31 7.31
7.26 CDCl3
7.24 7.24 7.23 7.23 7.22 7.21 7.21 7.21 5.25 5.24 5.23 5.21 4.75 4.74 4.73 4.73 4.72 4.71 4.70 4.70 4.69 4.68 4.68 4.68 4.66 4.66 4.65 4.65 4.63 4.63 4.02 4.00 4.00 3.98 1.34 1.32 1.29 1.28 1.28 1.26
20
1.00
0.96
1.54
1.03
1.94
0.99
12.34
ppm

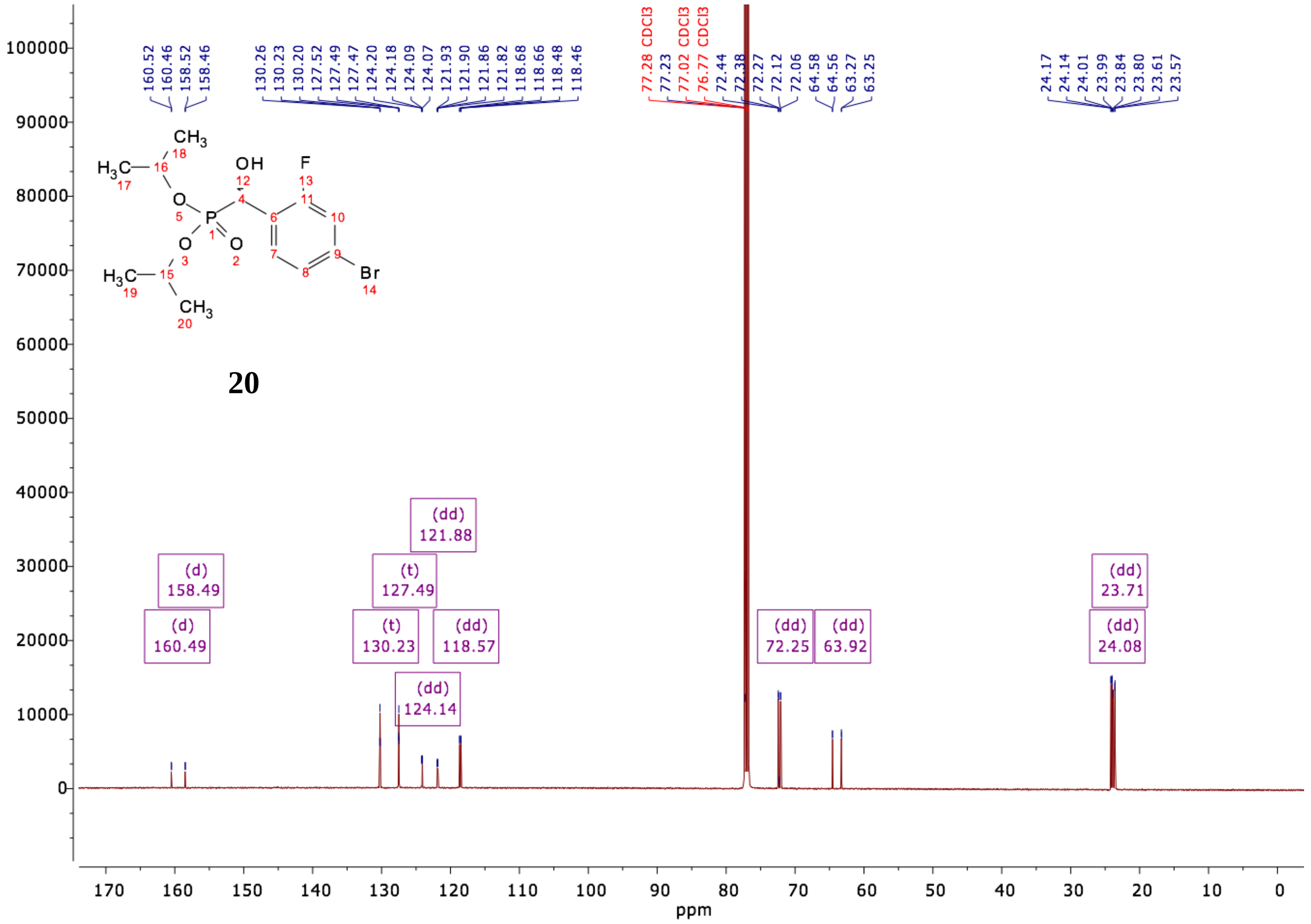
160.52 160.46 158.52 158.46
130.26 130.23 130.20 127.52 127.49 127.47 124.20 124.18 124.09 124.07 121.93 121.90 121.86 121.82 118.68 118.66 118.48 118.46
77.28 CDCl3
77.23
77.02 CDCl3
76.77 CDCl3
72.44 72.38 72.27 72.12 72.06 64.58 64.56 63.27 63.25
24.17 24.14 24.01 23.99 23.84 23.80 23.61 23.57
20
(dd) 121.88
(d) 158.49
(t) 127.49
(dd) 23.71
(d) 160.49
(t) 130.23
(dd) 118.57
(dd) 72.25
(dd) 63.92
(dd) 24.08
(dd) 124.14
ppm

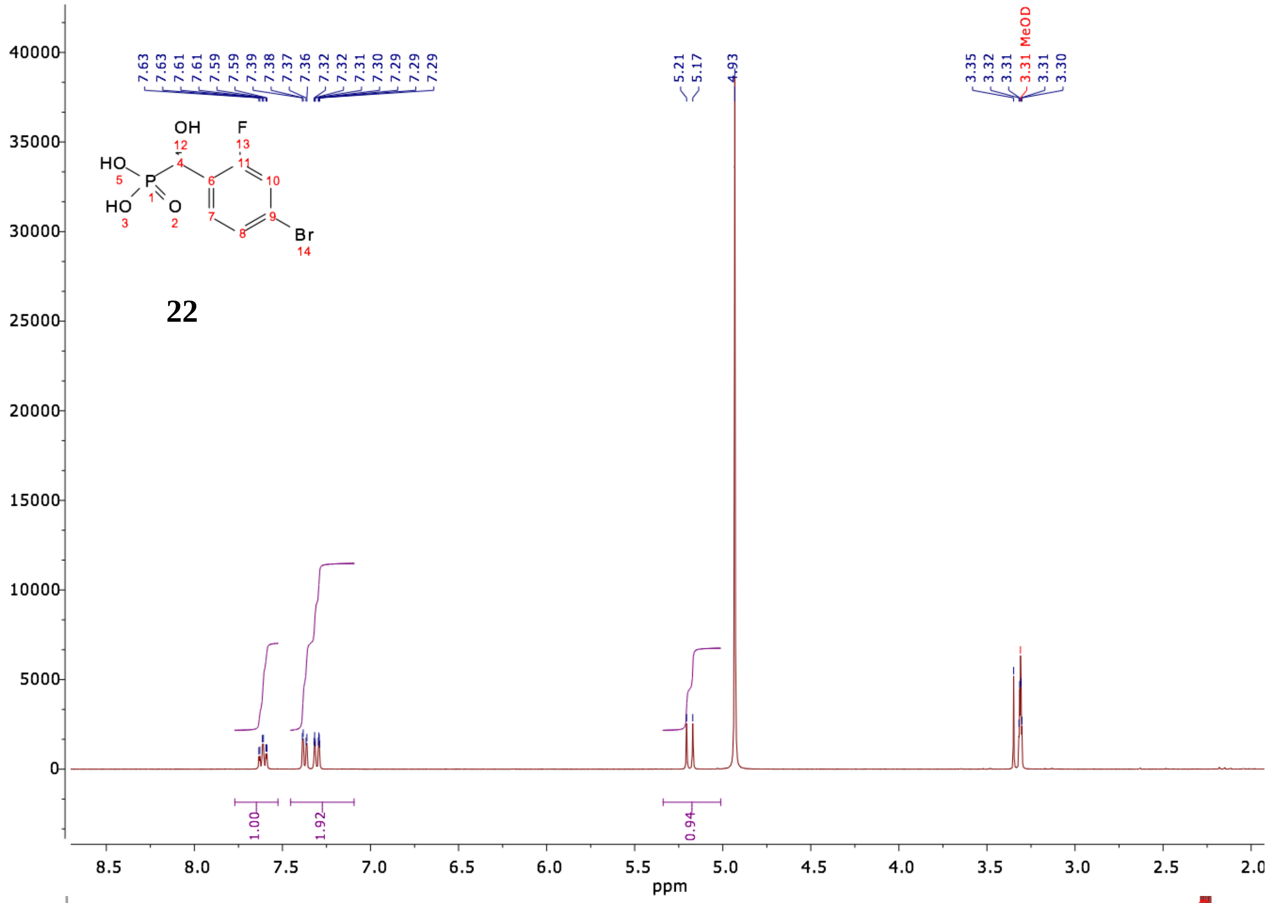
7.63 7.63 7.61 7.61 7.59 7.59 7.39 7.38 7.37 7.36 7.32 7.32 7.31 7.30 7.29 7.29 7.29
5.21 5.17
4.93
3.35 3.32 3.31 3.31 MeOD 3.31 3.30
OH
F
HO
P
HO
O
Br
22
1.00
1.92
0.94
ppm

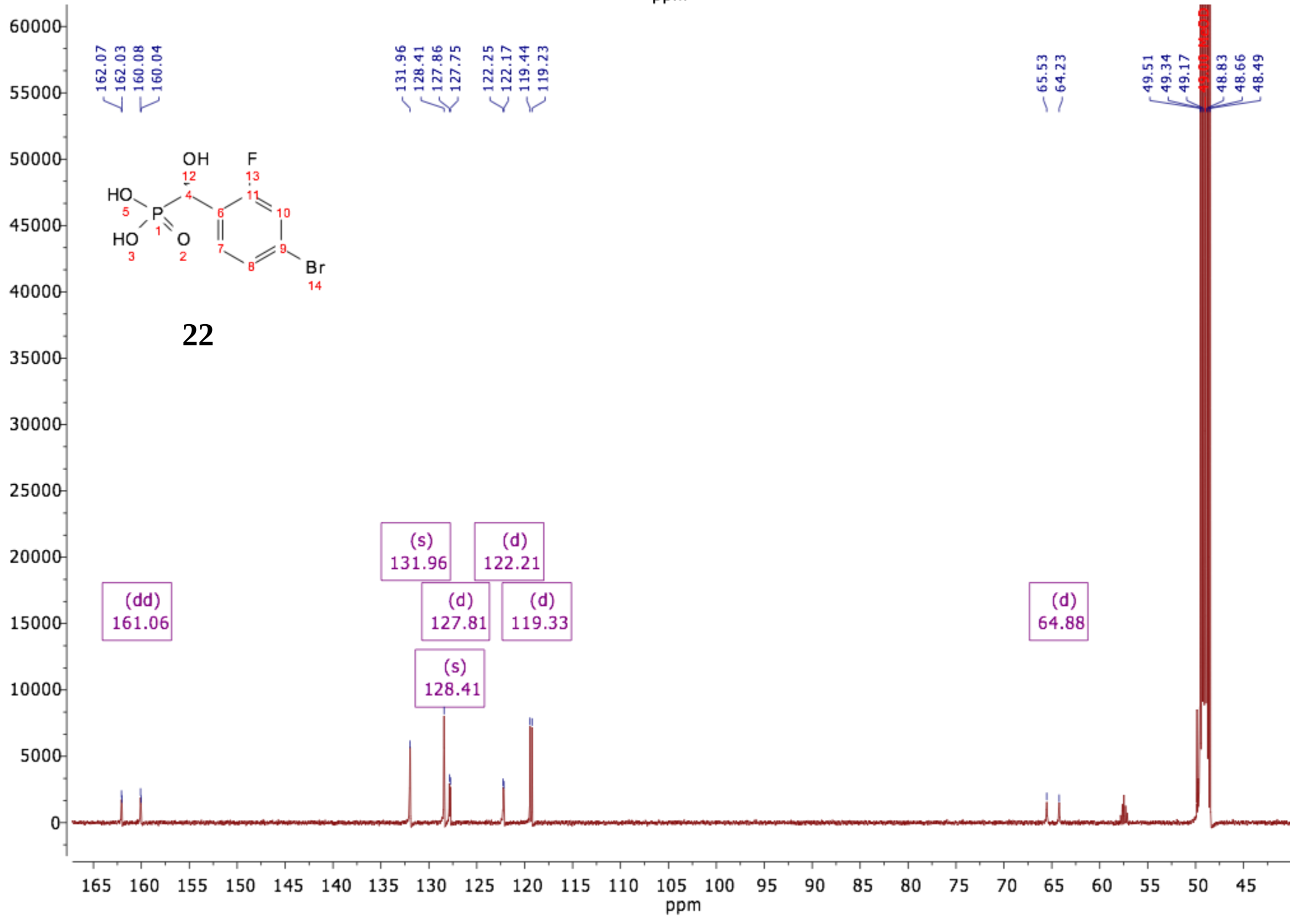
162.07 162.03 160.08 160.04
131.96 128.41 127.86 127.75
122.25 122.17 119.44 119.23
65.53 64.23
49.51 49.34 49.17 MeOD 48.83 48.66 48.49
OH
F
HO
P
HO
O
Br
22
(dd) 161.06
(s) 131.96
(d) 122.21
(d) 127.81
(d) 119.33
(s) 128.41
(d) 64.88
ppm

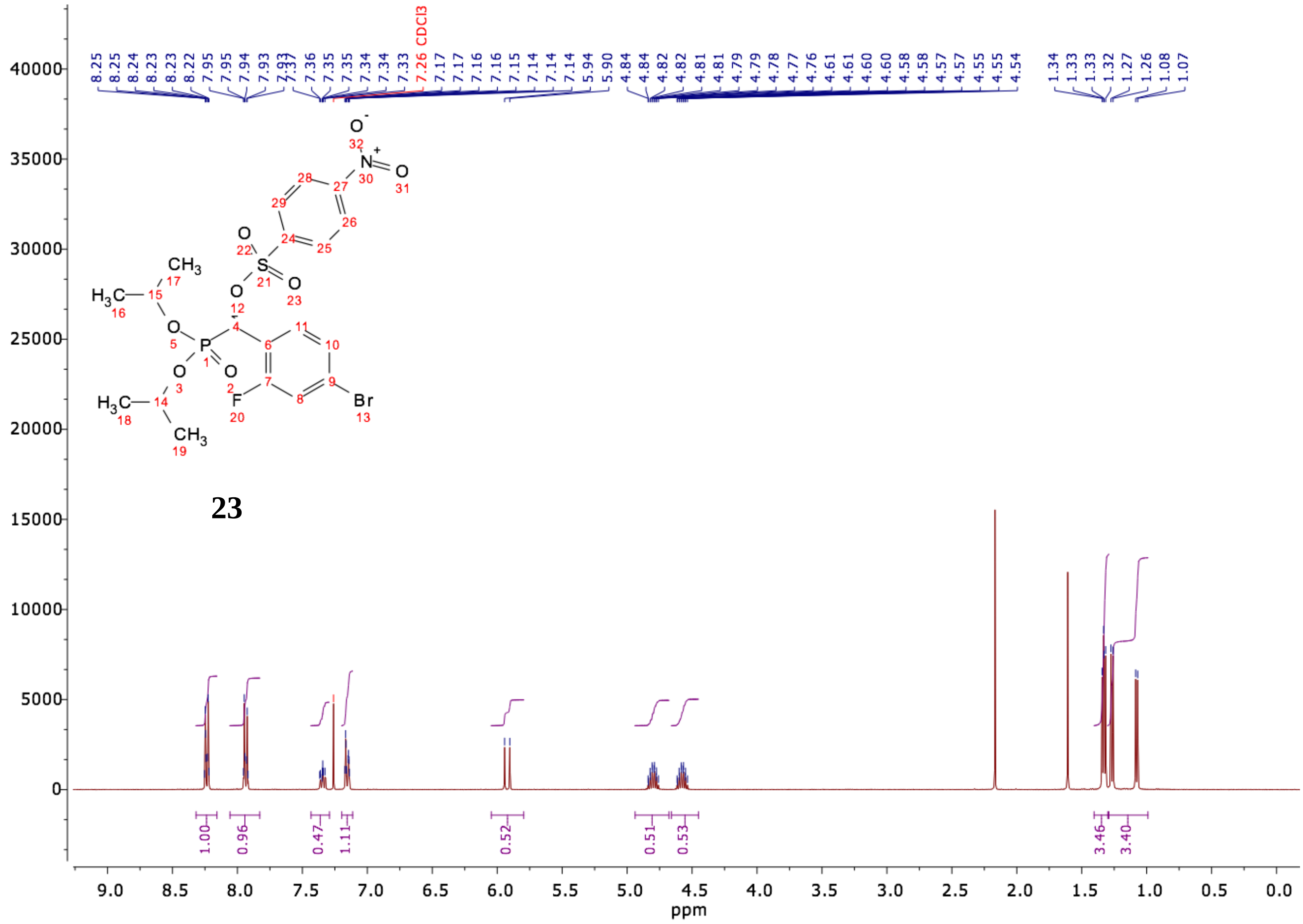

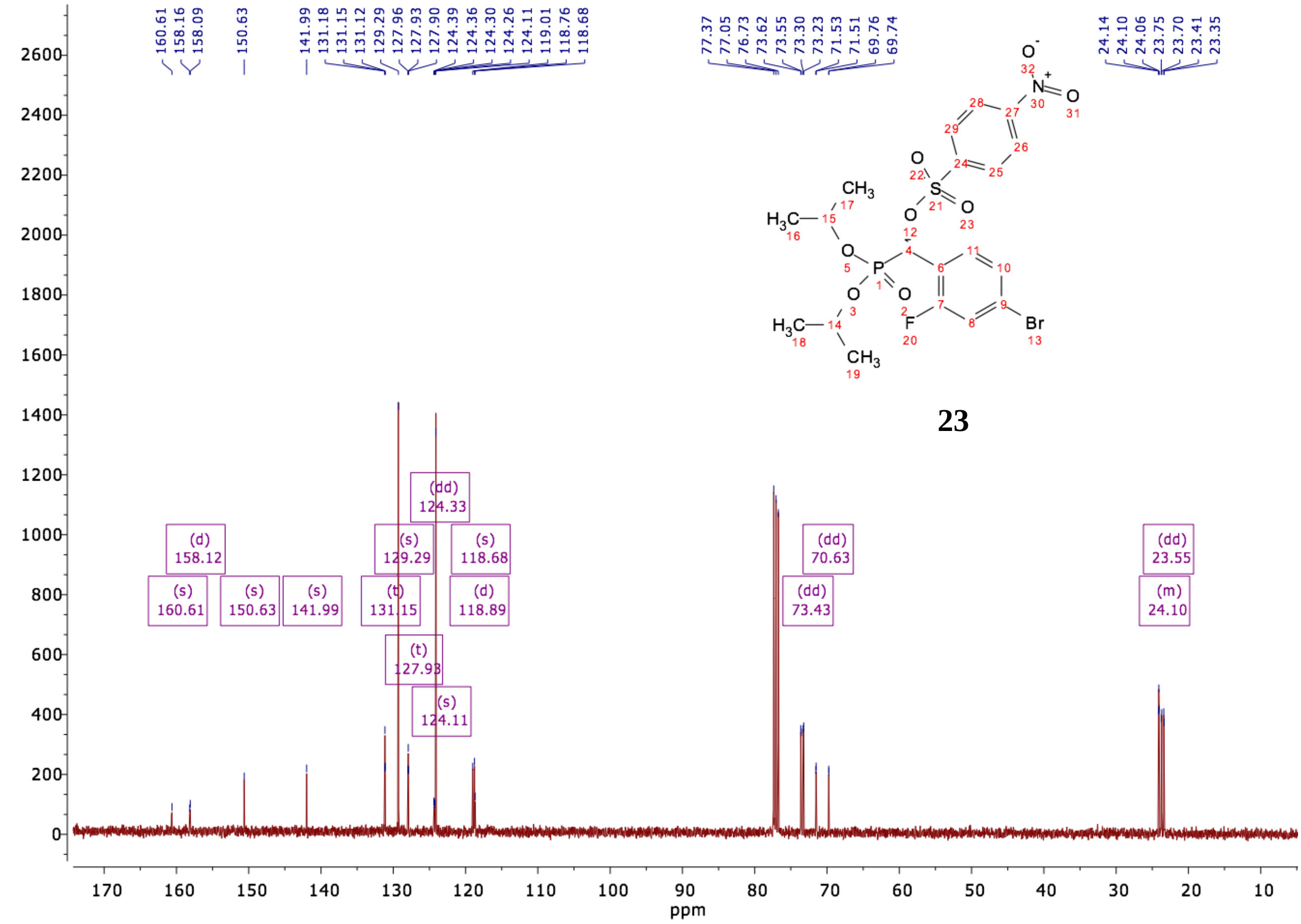

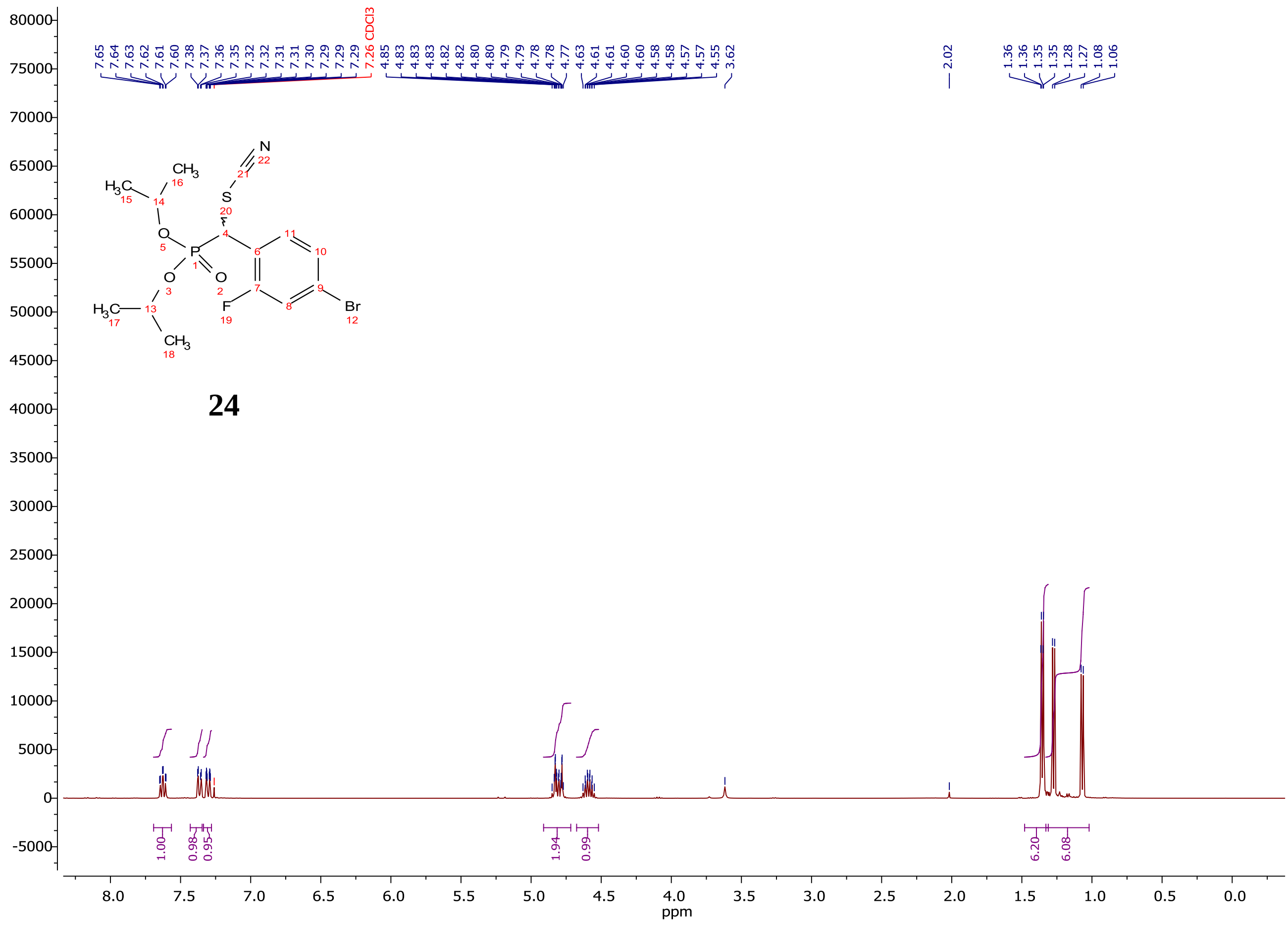

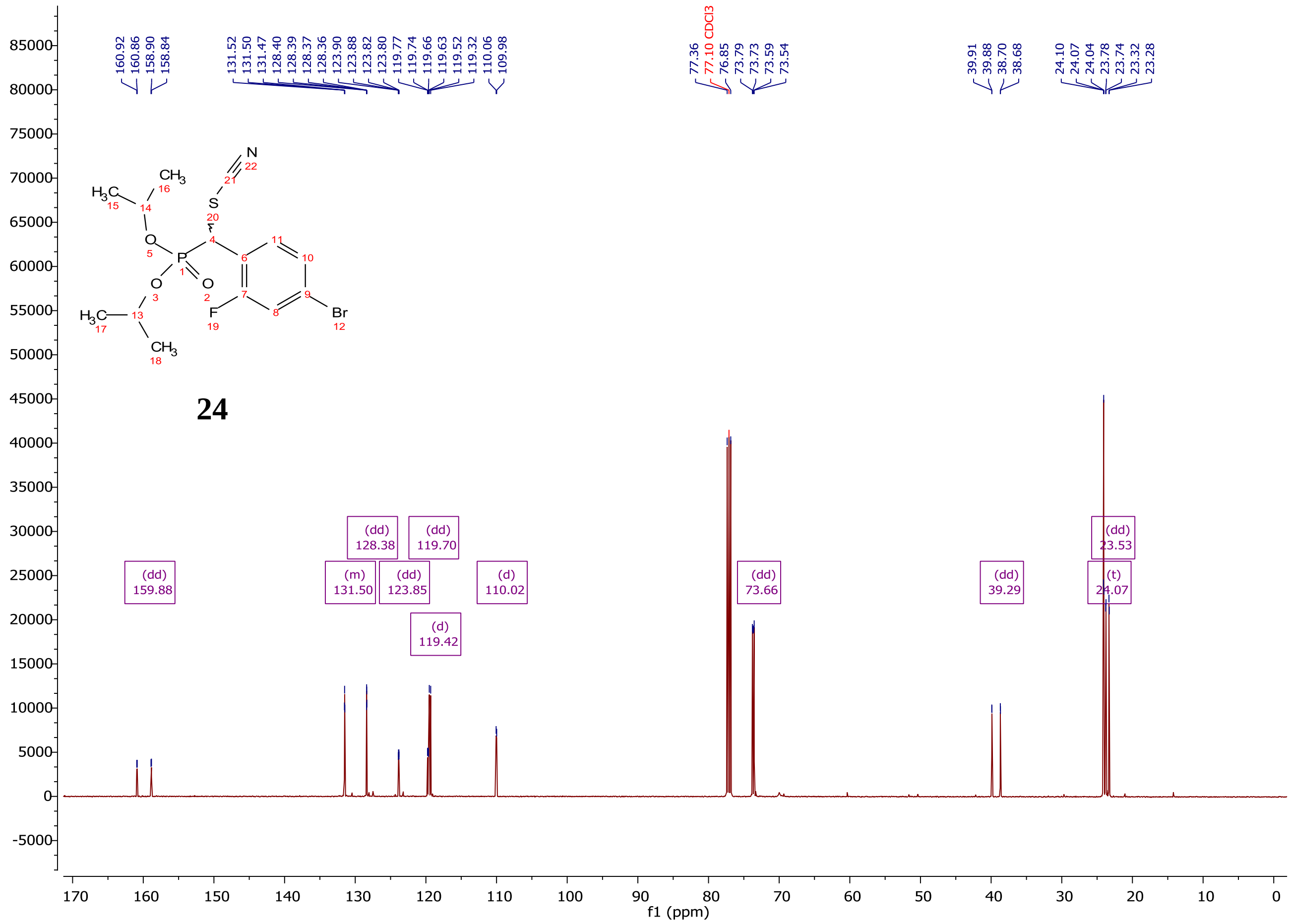

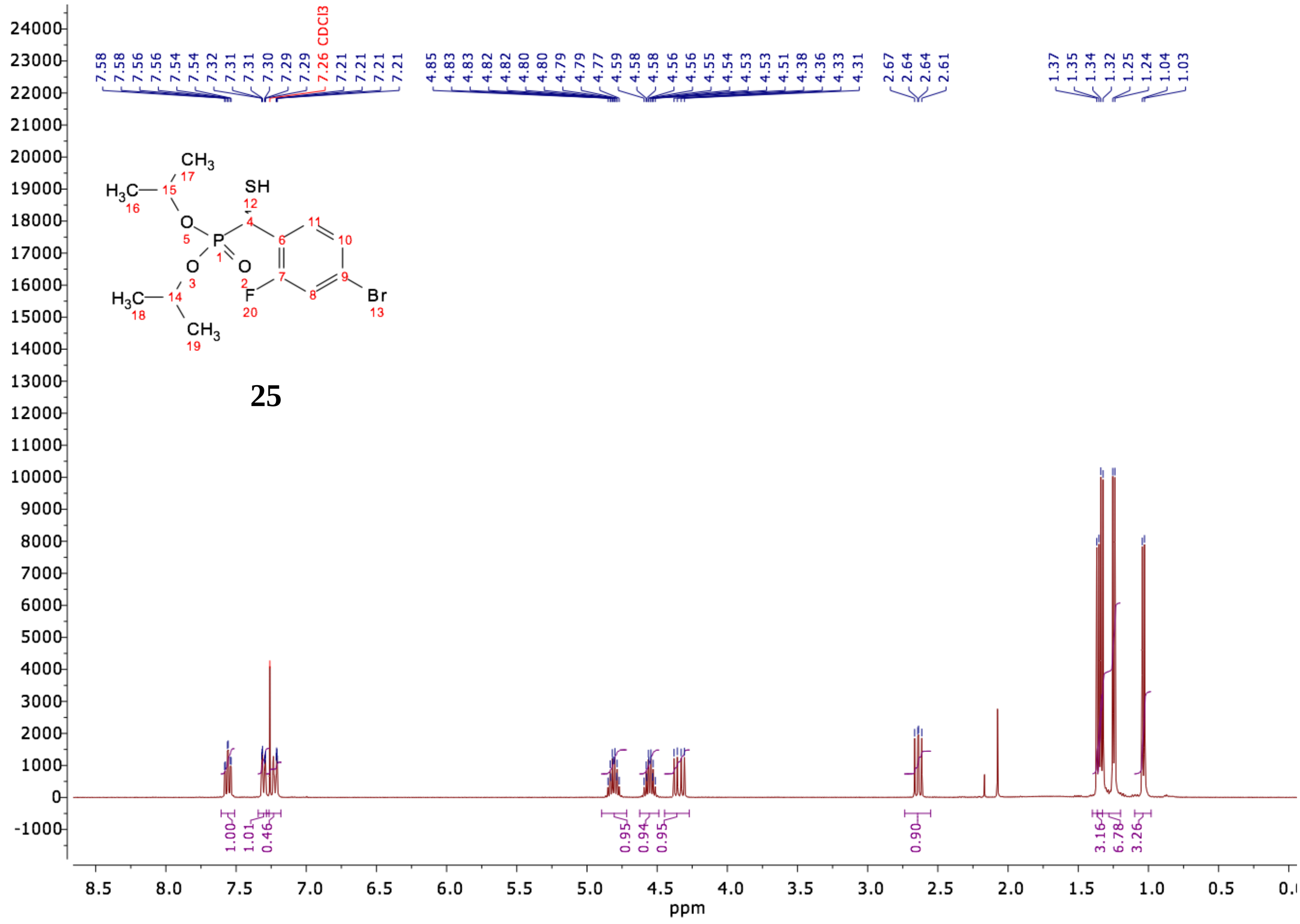

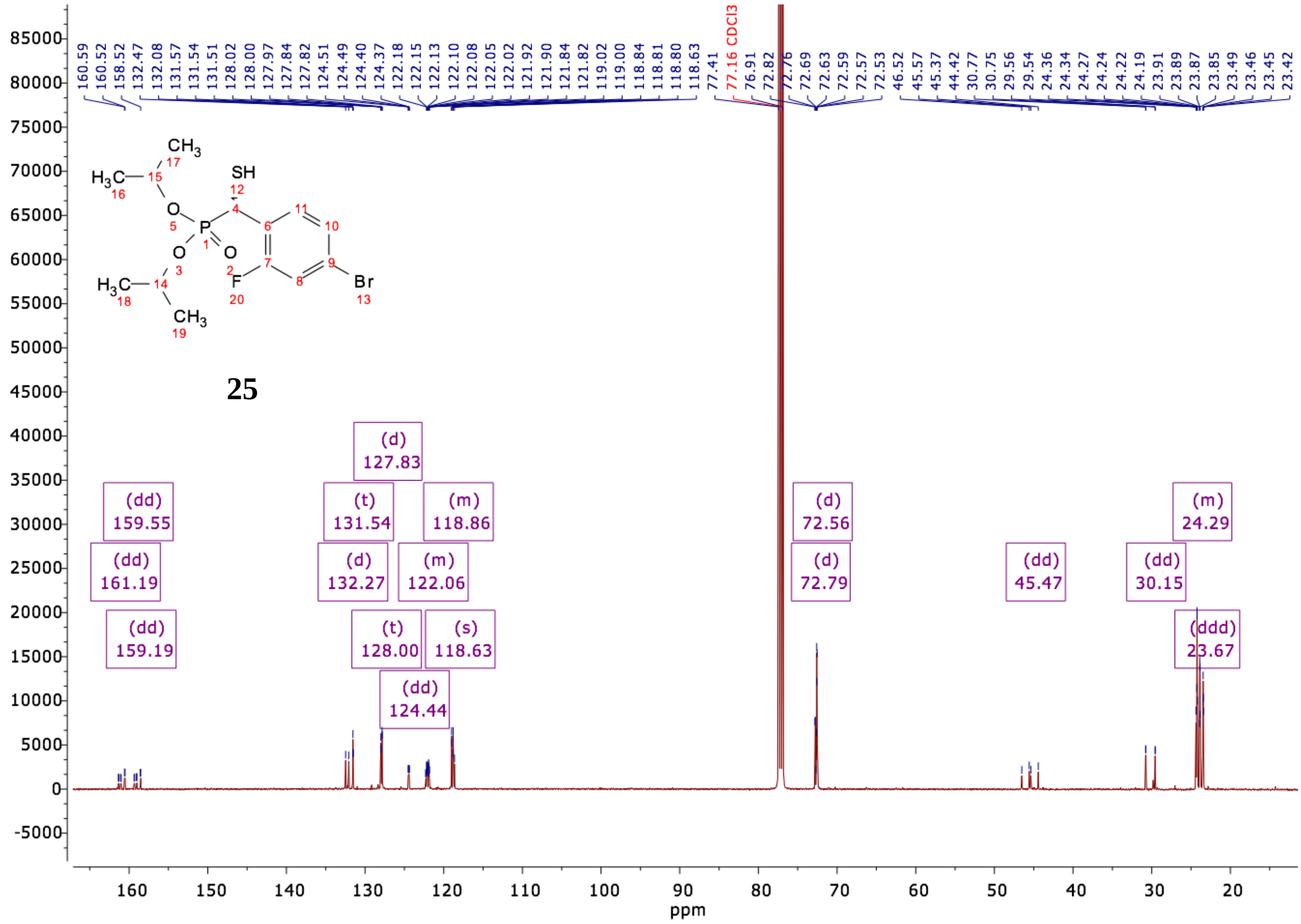

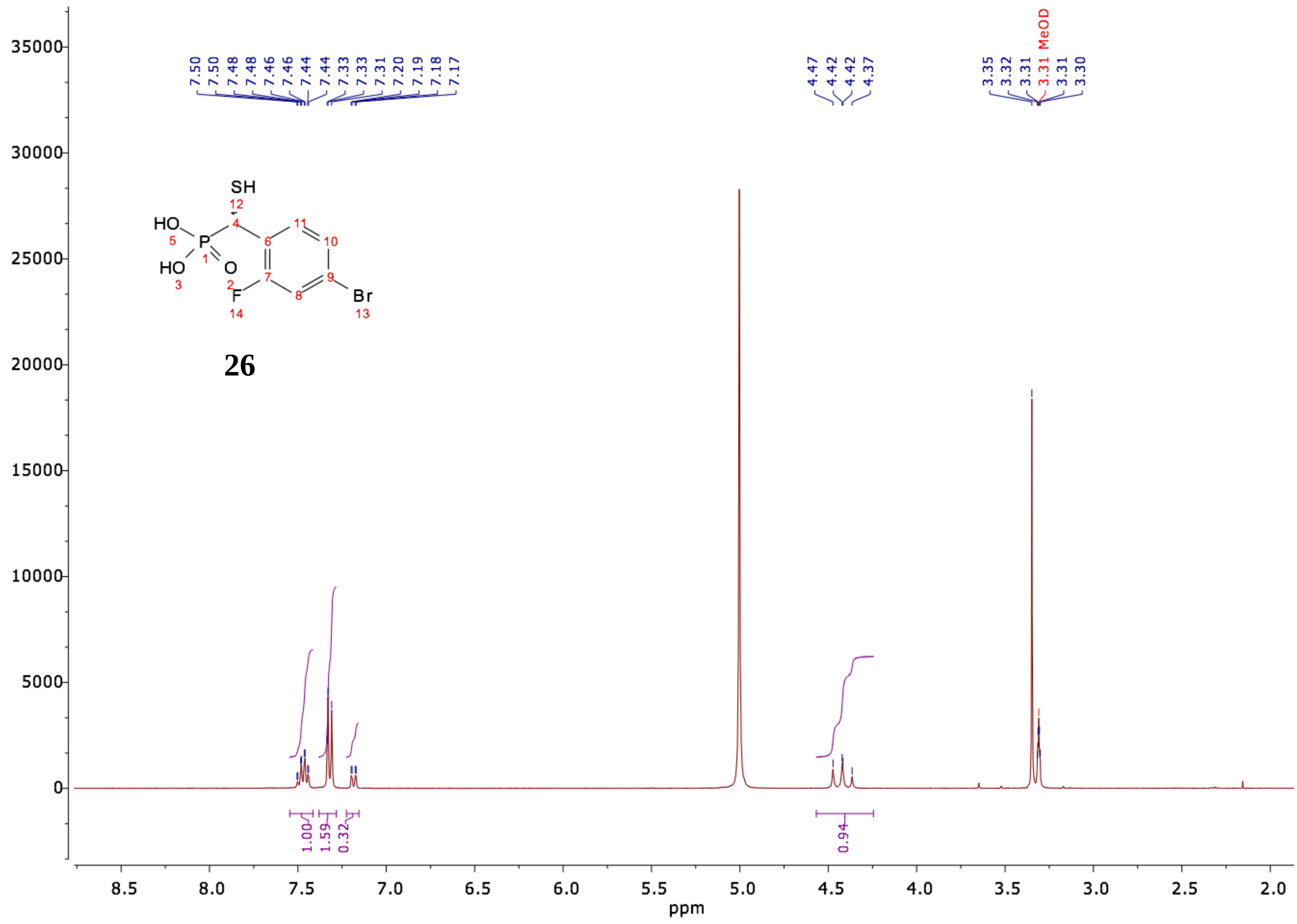
7.50 7.50 7.48 7.48 7.46 7.46 7.44 7.44 7.33 7.33 7.31 7.20 7.19 7.18 7.17
4.47 4.42 4.42 4.37
3.35 3.32 3.31 3.31 MeOD 3.31 3.30
SH
HO
P
HO
O
F
Br
26
1.00
1.59
0.32
0.94
ppm

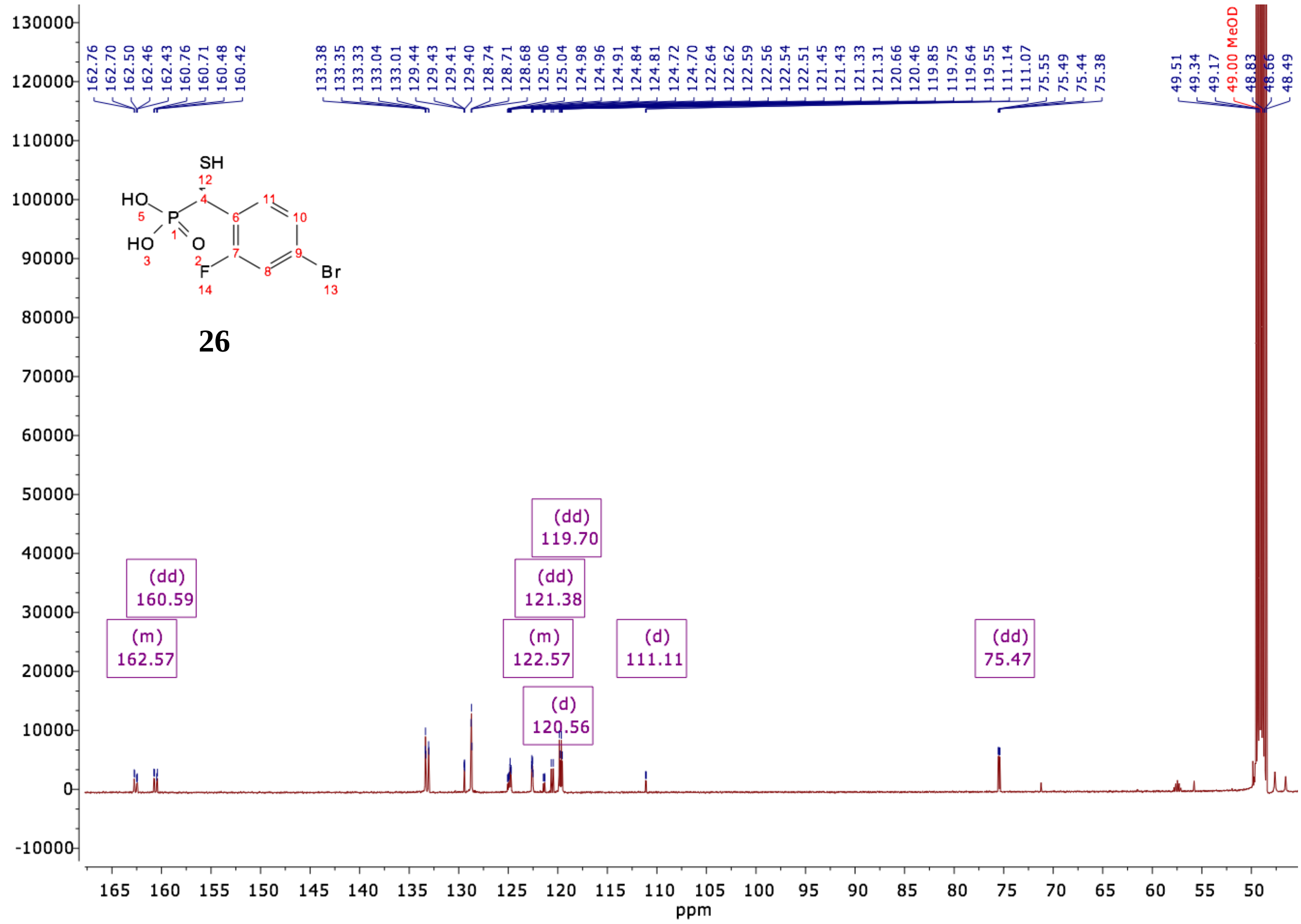
162.76 162.70 162.50 162.46 162.43 160.76 160.71 160.48 160.42
133.38 133.35 133.33 133.04 133.01 129.44 129.43 129.41 129.40 128.74 128.71 128.68 125.06 125.04 124.98 124.96 124.91 124.84 124.81 124.72 124.70 122.64 122.62 122.59 122.56 122.54 122.51 121.45 121.43 121.33 121.31 120.66 120.46 119.85 119.75 119.64 119.55 111.14 111.07 75.55 75.49 75.44 75.38
49.51 49.34 49.17 49.00 MeOD 48.83 48.66 48.49
SH
HO
P
HO
O
F
Br
26
(dd) 160.59
(m) 162.57
(dd) 119.70
(dd) 121.38
(m) 122.57
(d) 111.11
(d) 120.56
(dd) 75.47
ppm

4. Extended biochemical results and tables:

Table S1. Residual activities of metallo-β-lactamases (MBLs) upon inhibition by selected rhodanine/thioenol derivatives at 100 µM.

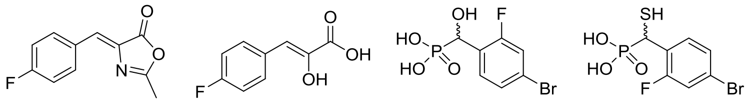

16 **17** **22** **26**

	% Residual activity versus				
**Compound**	**SPM-1**	**BcII**	**IMP-1**	**VIM-2**	**NDM-1**
**16**	87 ± 1	92 ± 2	100	100	97 ± 2
**17**	93 ± 2	90 ± 3	100	100	97 ± 4
**22**	92 ± 5	98 ± 2	100	100	100
**26**	95 ± 6	100	100	100	96 ± 3

BcII, *Bacillus cereus* II MBL; IMP-1, Imipenemase MBL-1; NDM-1,New Delhi MBL-1; SPM-1, São Paulo MBL-1; VIM-2, Verona integron-encoded MBL-2

Figure S1. Residual activities of serine-β-lactamases (SBLs) upon inhibition by rhodanines and enethiols at 200µM.

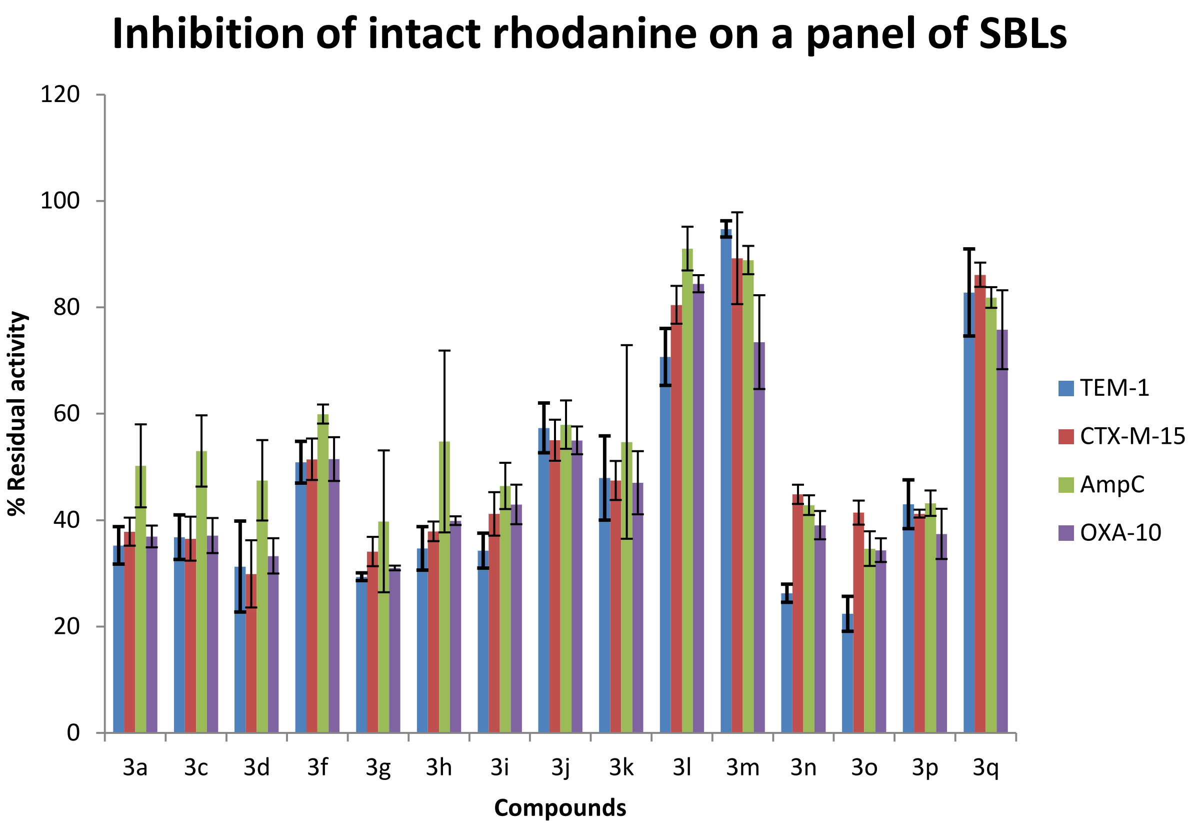

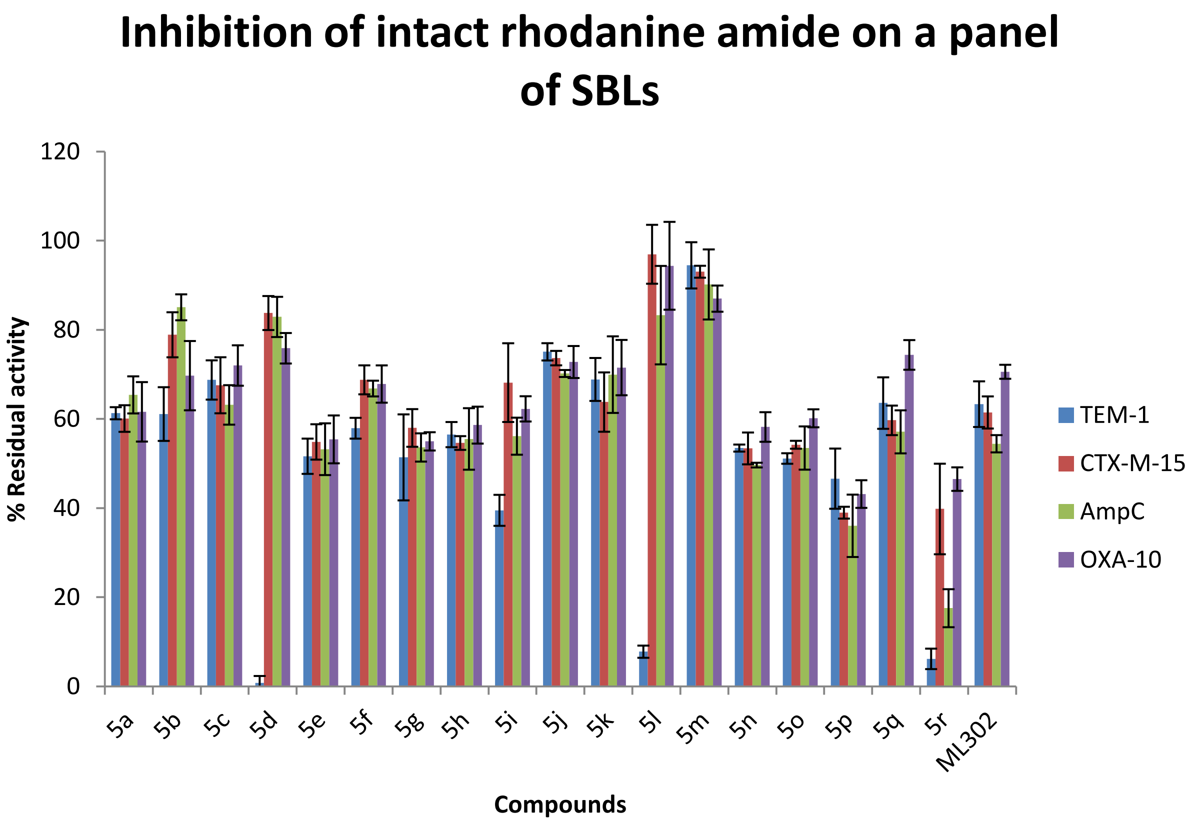

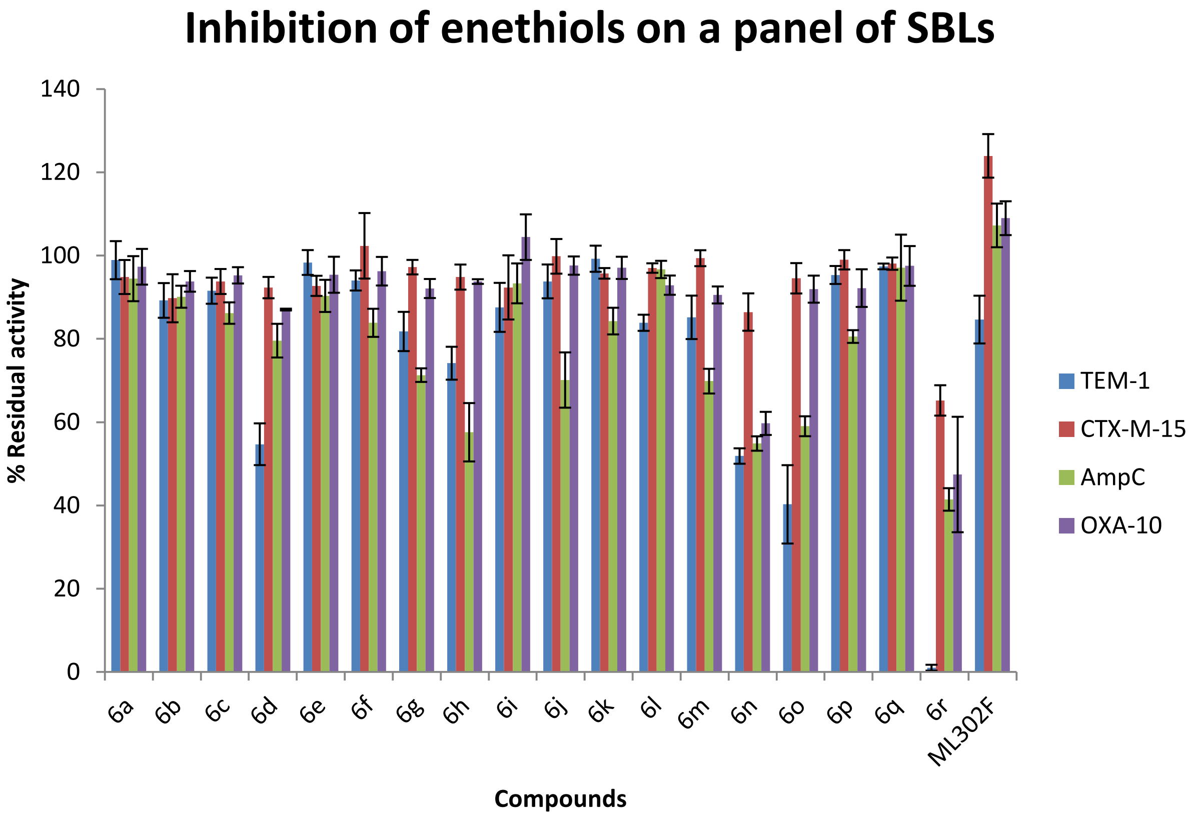

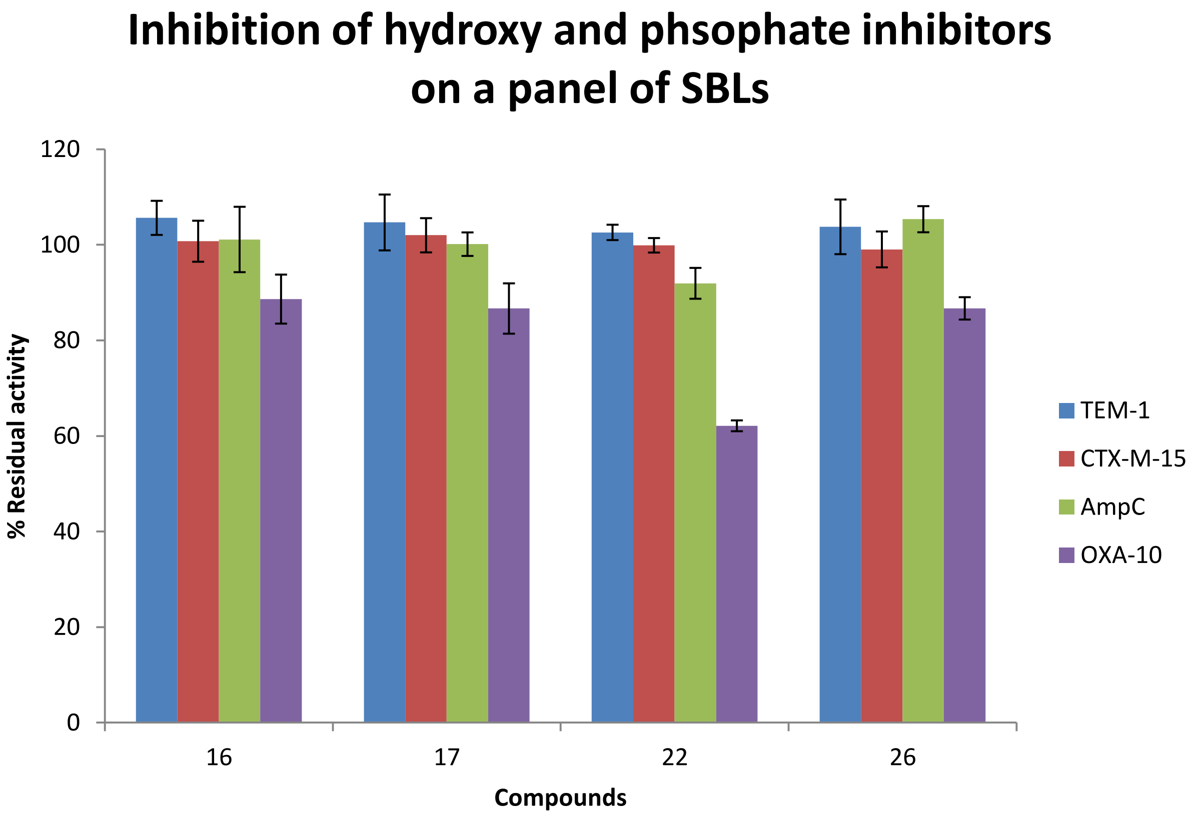

AmpC, Ampicillin resistant β-lactamase class C; CTX-M-15, Cefotaxime hydrolysing β-lactamase from Munich 15; OXA-10, Oxacillinase-10; TEM-1, Temoneira β-lactamase-1

Figure S2: IC_{50} curves for BcII inhibition time courses. Residual activities were calculated from the initial rate of FC5 hydrolysis after incubation of the enzyme with the inhibitor for 10 minutes.

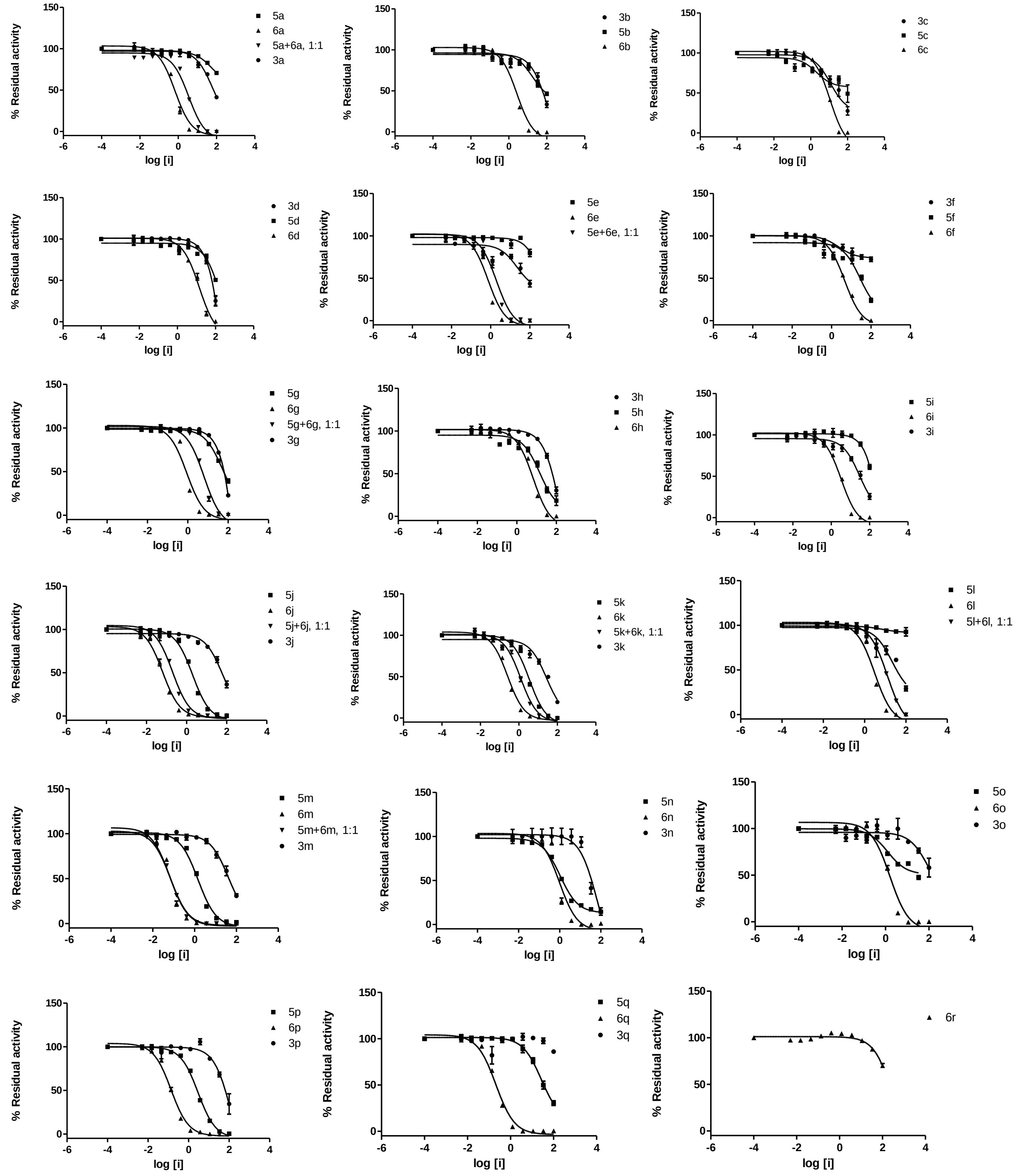

Figure S2 continued

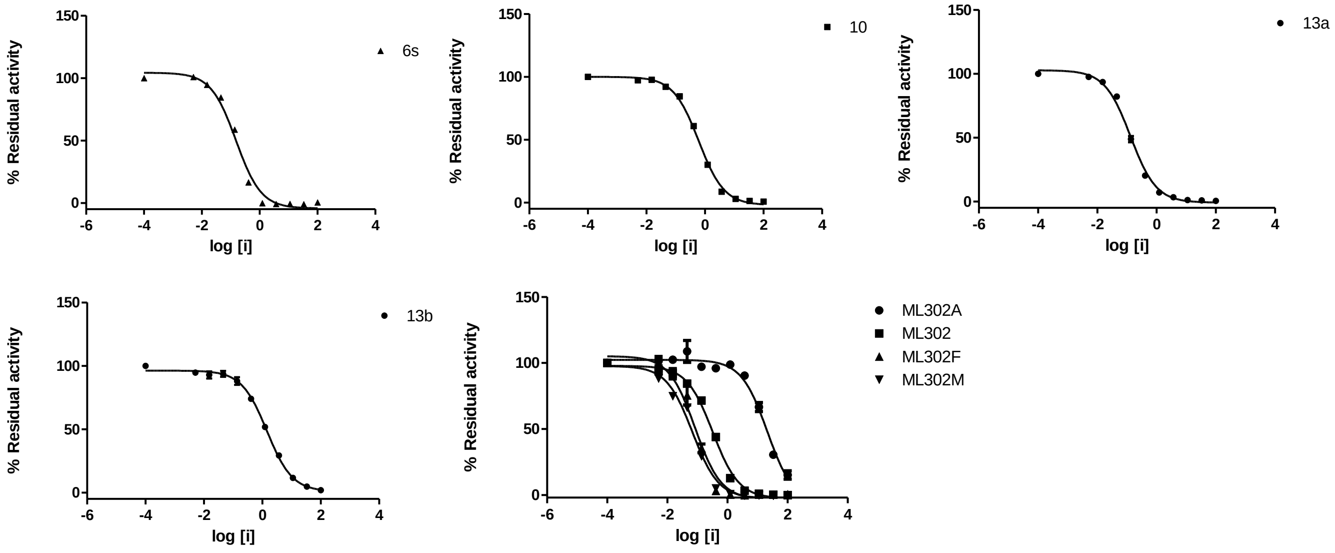

Figure S3: IC_{50} curves for IMP-1 inhibition time courses. Residual activities were calculated from the initial rate of FC5 hydrolysis after incubation of the enzyme with the inhibitor for 10 minutes.

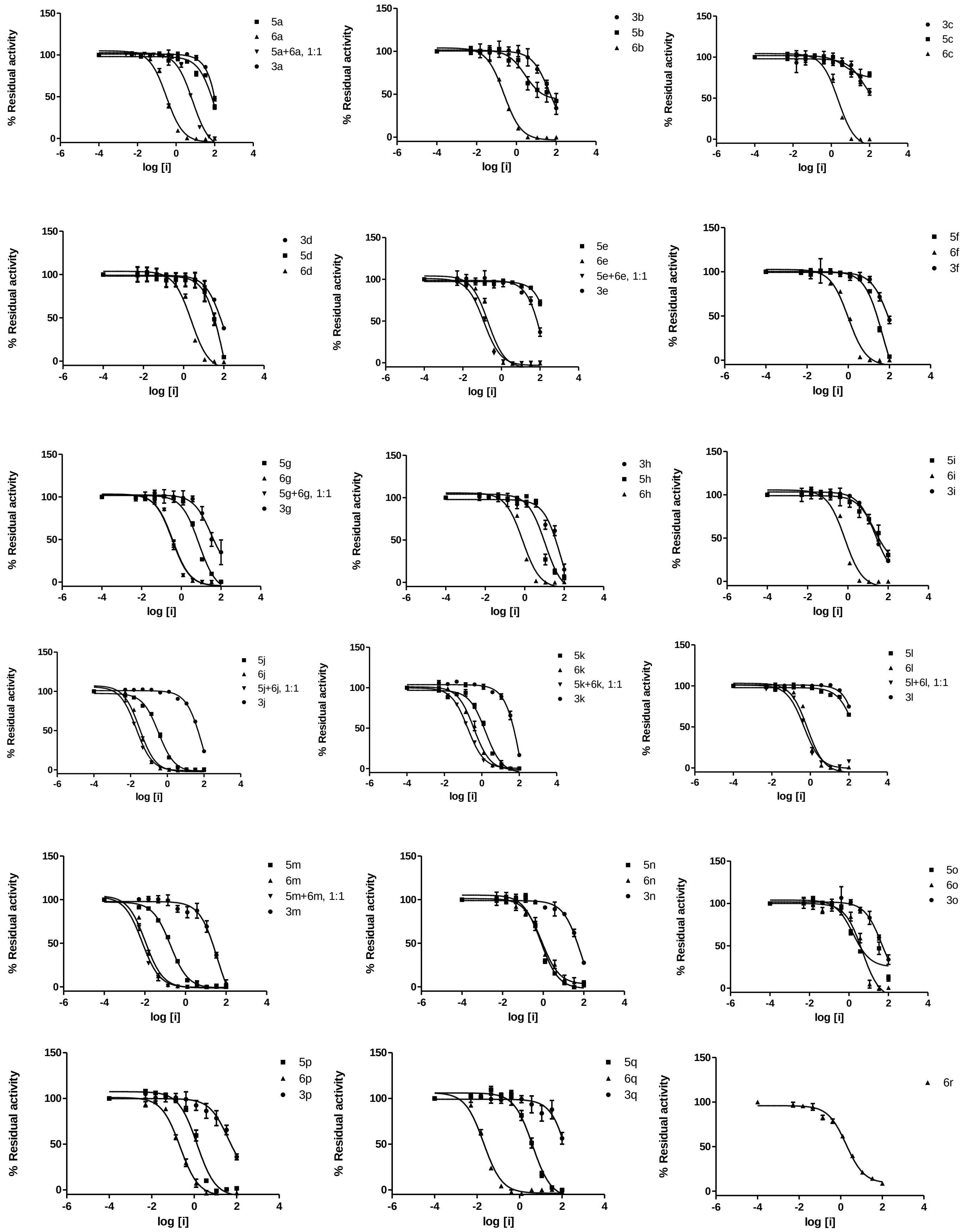

Figure S3 continued

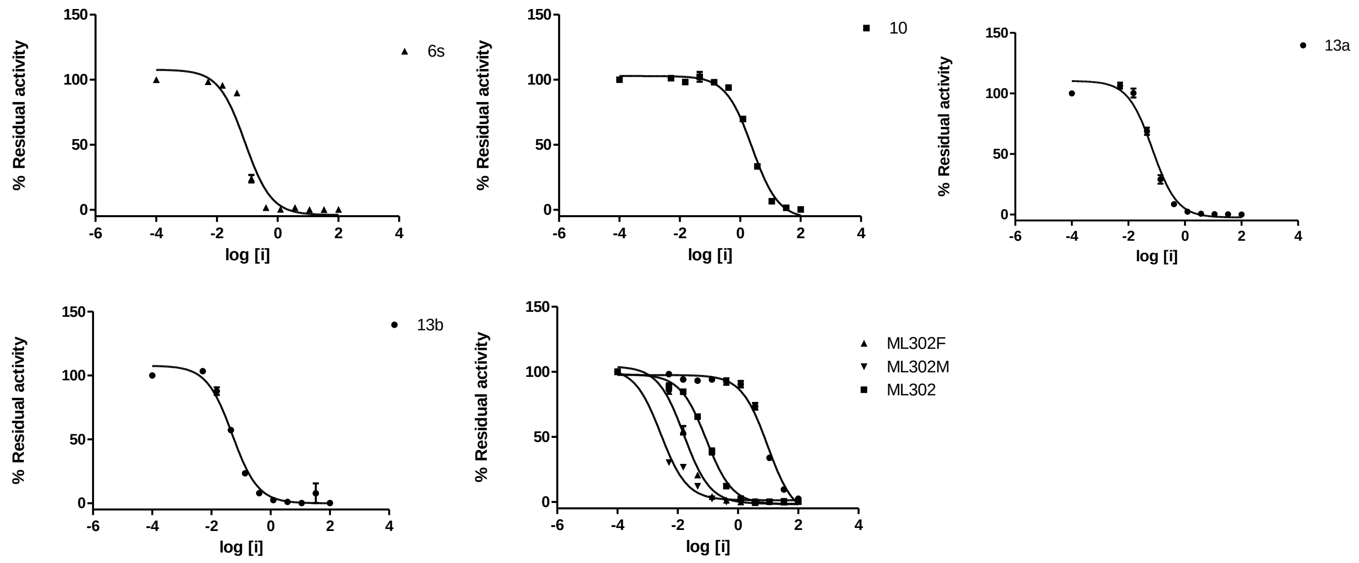

Figure S4: IC_{50} curves for NDM-1 inhibition time courses. Residual activities were calculated from the initial rate of FC5 hydrolysis after incubation of the enzyme with the inhibitor for 10 minutes.

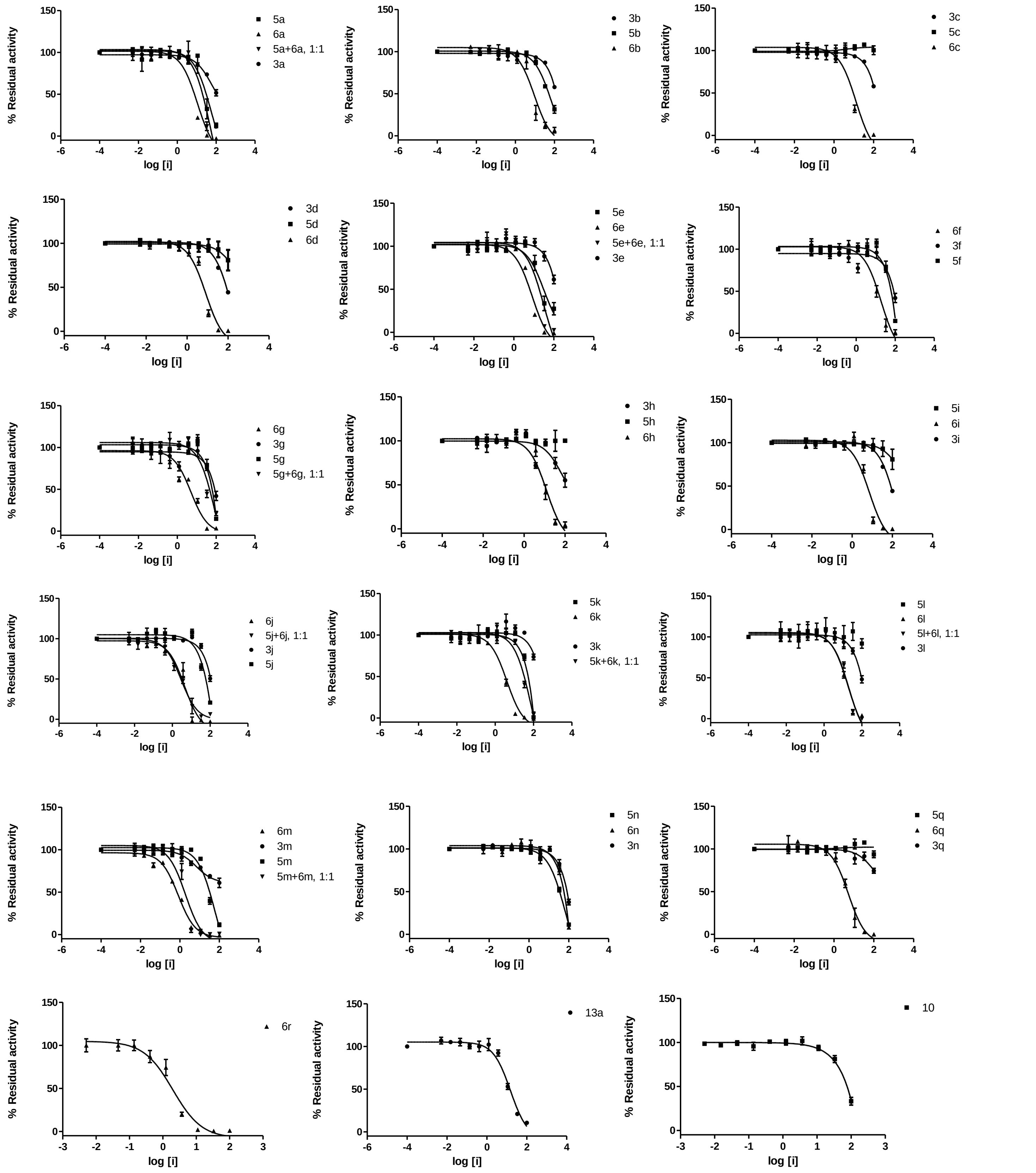

Figure S4 continued

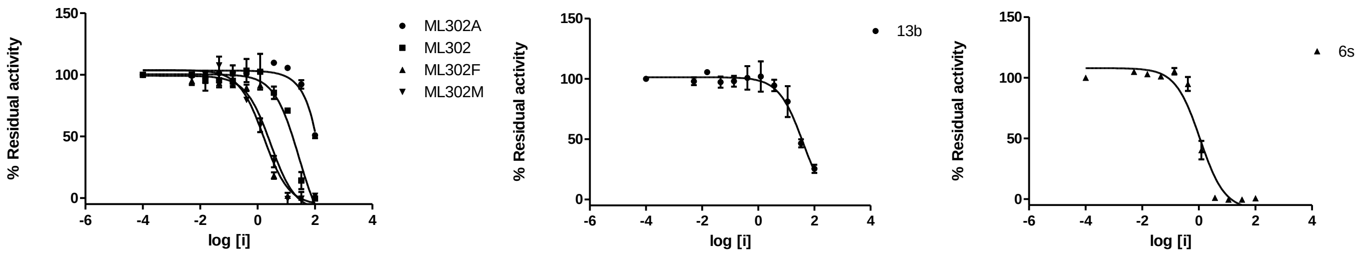

Figure S5: IC_{50} curves for SPM-1 inhibition time courses. Residual activities were calculated from the initial rate of FC5 hydrolysis after incubation of the enzyme with the inhibitor for 10 minutes.

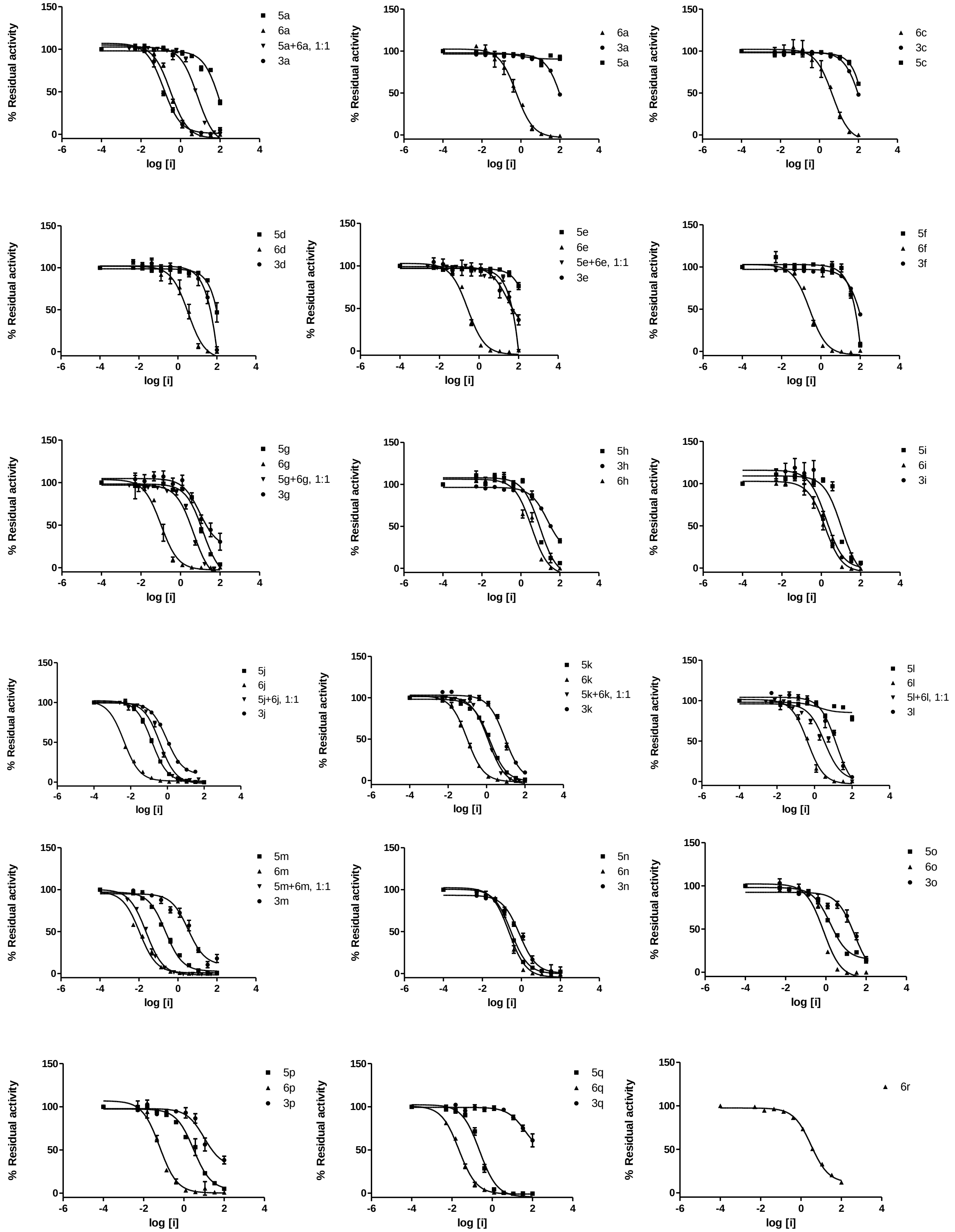

Figure S5 continued

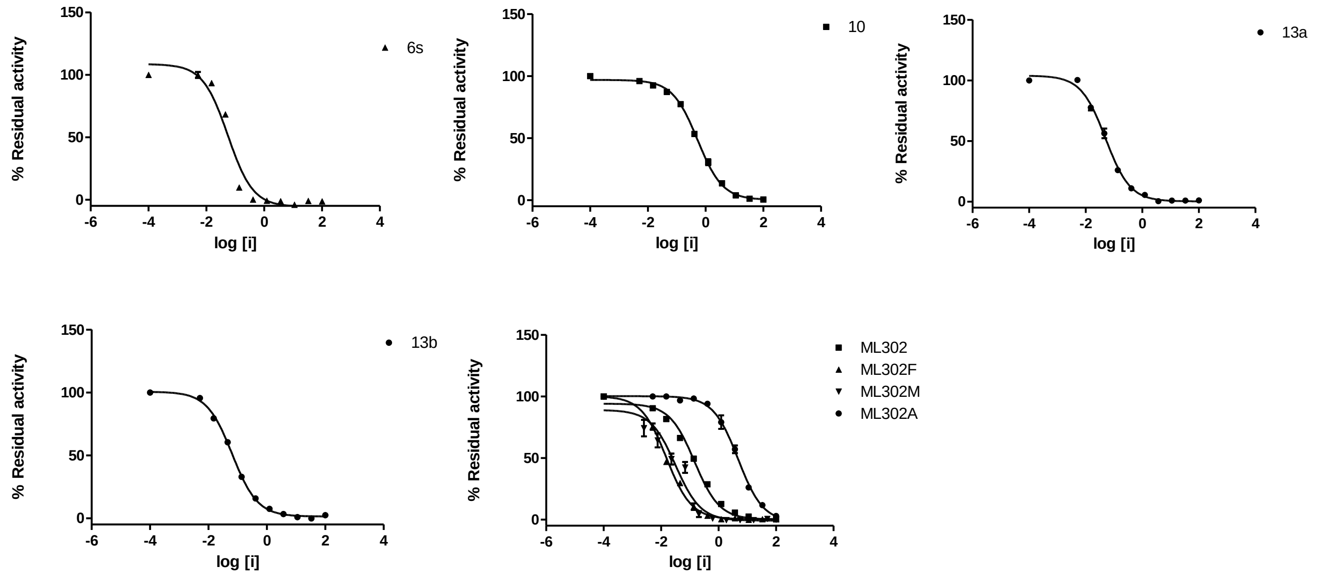

Figure S6: IC_{50} curves for VIM-2 inhibition time courses. Residual activities were calculated from the initial rate of FC5 hydrolysis after incubation of the enzyme with the inhibitor for 10 minutes.

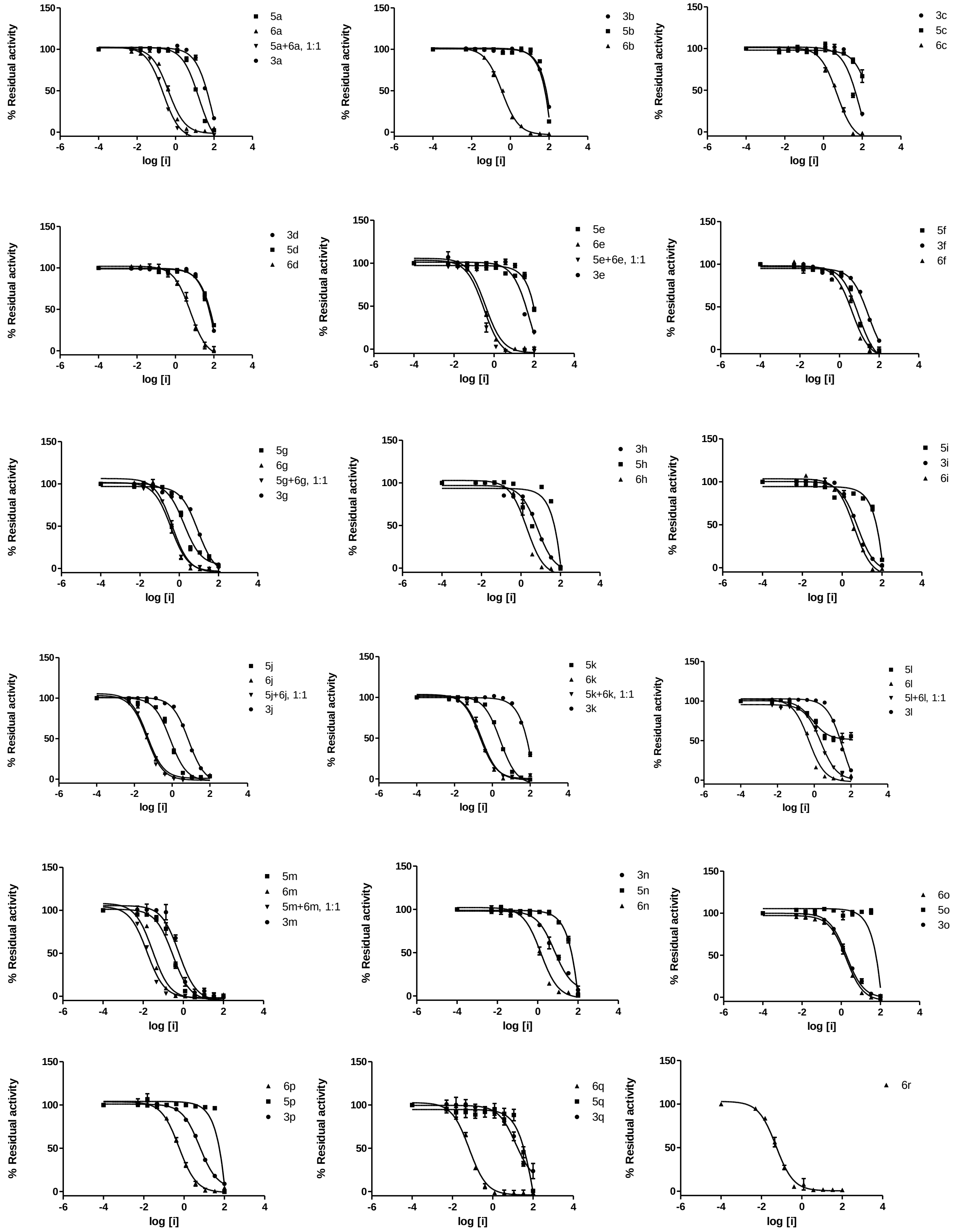

Figure S6 continued

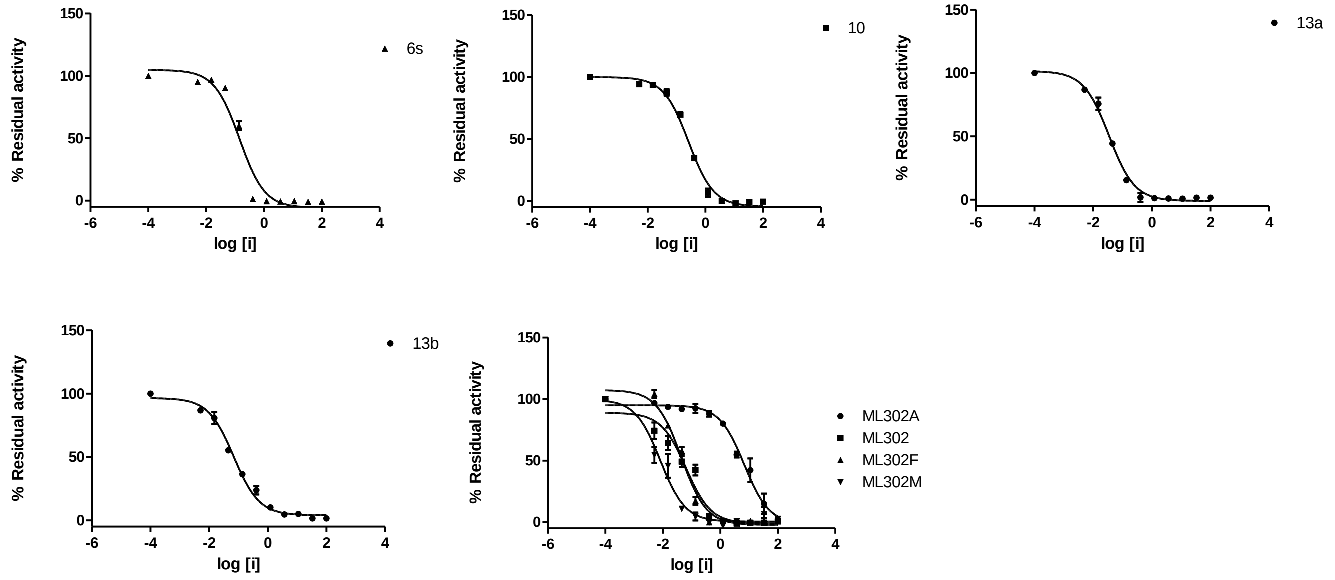

Figure S7. ^{1}H NMR (700MHz) stability test for compound **10**. ***A***, **10** (200 µM) in NH_4HCO_3 (50 mM, pH 7.5 in D_2O) in the absence of NDM-1, ^{1}H NMR time course over 18 hours; ***B***, **10** (200 µM) in NH_4HCO_3 (50 mM, pH 7.5 in D_2O) in the presence of NDM-1 (1 µM), ^{1}H NMR time course over 18 hours; ***C***, **10** (200 µM) in in Tris-d_{11} (50 mM, pH 7.5 in D_2O) in the absence of NDM-1, ^{1}H NMR time course over 18 hours; ***D***, **10** (200 µM) in Tris-d_{11} (50 mM, pH 7.5 in D_2O) and in the presence of NDM-1 (1 µM), ^{1}H NMR time course over 18 hours.

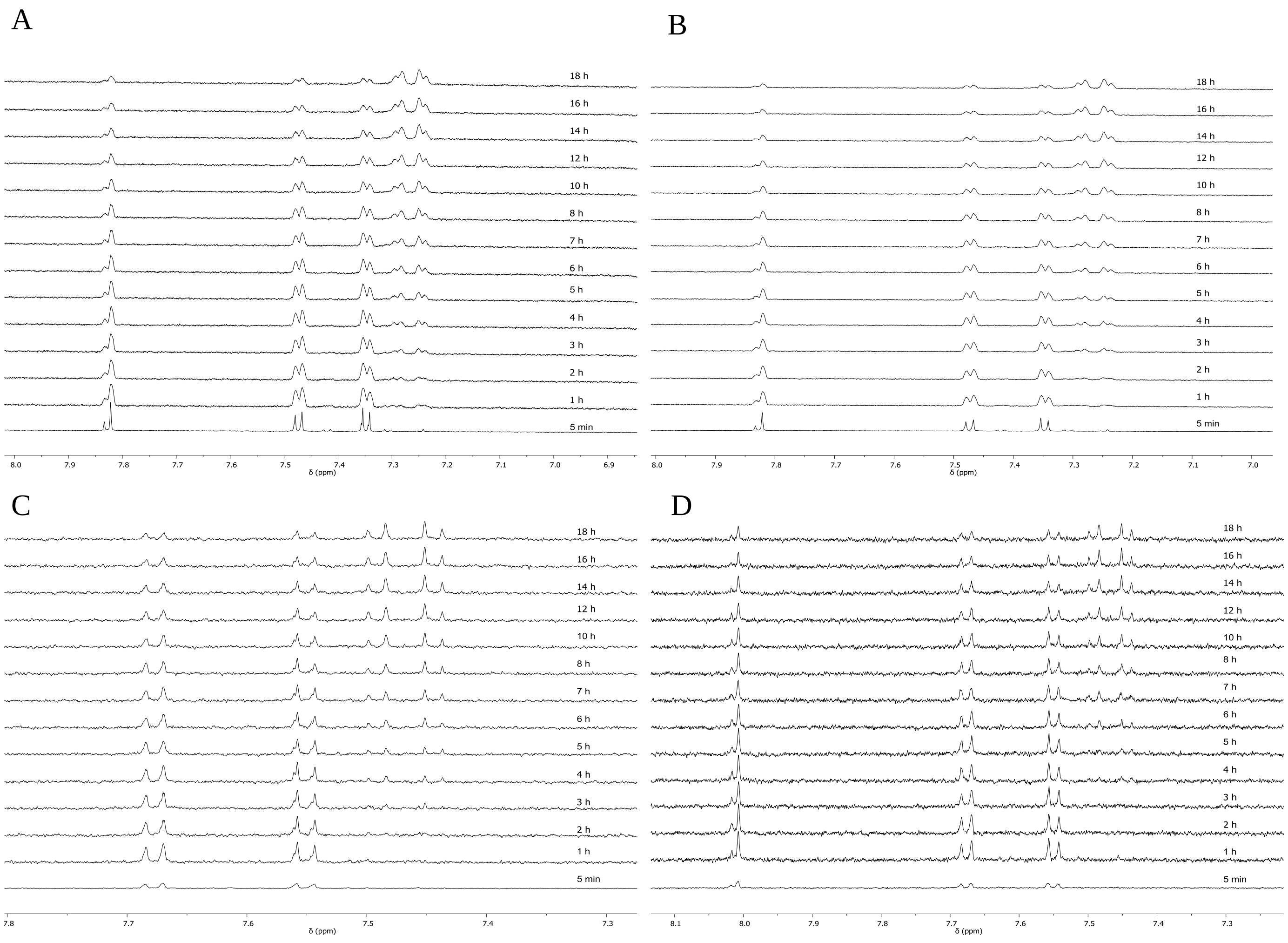

Figure S8. Reaction of compound **10** (200 µM initial concentration) monitored over 18 hours by ^{1}H NMR (600/700MHz) in: (A) in NH_4HCO_3 (50 mM, pH 7.5, D_2O solution) in the absence of NDM-1 (**10** purple diamonds, **10F/ML302F** grey triangles); (B) in NH_4HCO_3 (50 mM, pH 7.5, D_2O solution) in the presence of NDM-1 (1 µM) (**10** pink triangles, **ML302F** blue hexagons); (C) in Tris-d_{11} (50 mM, pH 7.5, D_2O solution) in the absence of NDM-1 (**10** red squares, **10F/ML302F** blue circles); and (D) in Tris-d_{11} (50 mM, pH 7.5, D_2O solution) in the presence of NDM-1 (1 µM) (**10** yellow triangles, **10F/ML302F** green triangles).

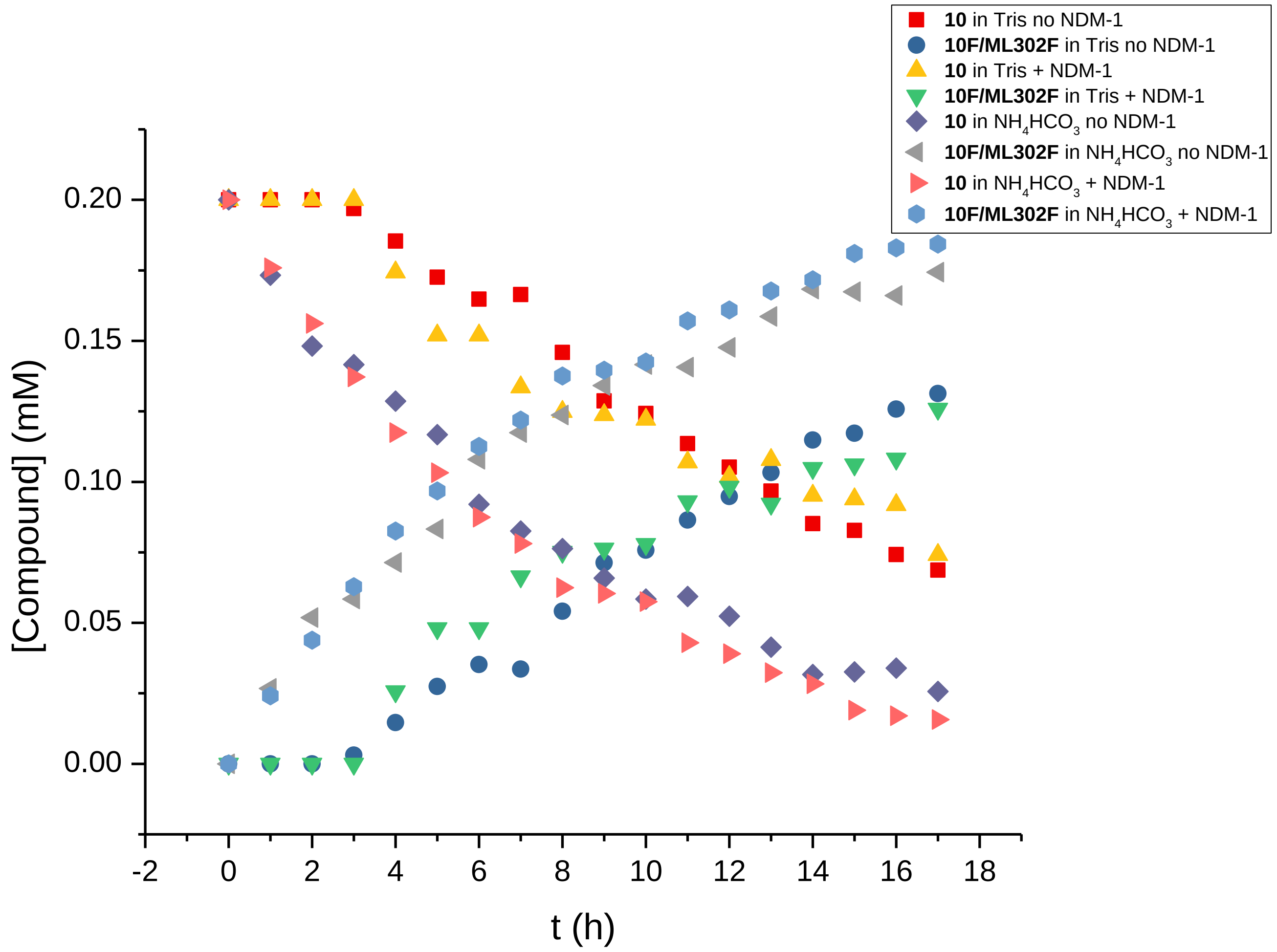

5. Crystallography

Table S2. Data collection and refinement statistics.

Dataset (PDB ID)	*BCII (6c) 305 (5JMX)*	*BCII (6k) 307 (6EUM)*	*BCII (6l) 308 (6EWE)*	*BCII (6s) KDU197 (6F2N)*	*VIM2 ML302F (6EW3)*
Beamline	DLS I03	DLS I02	DLS I03	DLS I03	DLS I04-1
Wavelength	0.97625	0.95000	0.97625	0.97625	0.92819
Resolution range§	68.95 - 1.44 (1.48 - 1.44)	19.53 - 1.18 (1.21 - 1.18)	35.31 - 1.46 (1.50 - 1.46)	26.87 - 1.15 (1.19 - 1.15)	51.27 - 2.14 (2.22 - 2.14)
Space group	$C\ 2_1$	$C\ 2_1$	$C\ 2_1$	$C\ 2_1$	$C\ 2_1$
Unit cell	53.25 61.24 69.05 90 93.09 90	53.18 61.44 70.18 90 93.2 90	53.19 61.01 69.54 90 92.99 90	53.17 61.67 69.68 90 93.132 90	102.48 78.91 67.46 90 130.48 90
Unique reflections§	40059 (2931)	71979 (5168)	38243 (2839)	77778 (7357)	22190 (2217)
Multiplicity§	6.6 (6.4)	16.5 (17)	6.6 (6.3)	6.6 (6.3)	3.4 (3.4)
Completeness (%)§	99.9 (100.0)	97.23 (94.63)	99.2 (98.6)	97.33 (92.15)	98.01 (98.27)
Mean I/σ(I)§	10.1 (1.1)	14.1 (1.1)	9.7 (1.1)	14.2	8.7 (2.1)
Wilson B-factor	19.35	15.48	20.07	11.70	22.50
R_{merge} §	0.083 (1.567)	0.090 (2.490)	0.089 (1.544)	ND	0.112 (0.517)
R_{meas} §	0.104 (1.189)	0.101 (2.642)	0.109 (0.724)	0.069	ND
R_{pim} §	0.041 (0.738)	0.025 (0.631)	0.043 (1.845)	ND	ND
$CC^{1}/_{2}$ §	0.998 (0.487)	0.999 (0.563)	0.998 (0.440)	0.998(0.753)	ND
Reflections used in refinement§	40041 (2906)	71973 (5146)	38231 (2830)	77738 (7345)	22187 (2217)
Reflections used for R_{free} §	2008 (219)	3416 (343)	1938 (232)	2002 (191)	1107 (138)
R_{work} †† §	0.1525 (0.3239)	0.1391 (0.2796)	0.1477 (0.2787)	0.1462 (0.3009)	0.1691 (0.2267)
R_{free} †† §	0.1859 (0.3490)	0.1574 (0.2938)	0.1739 (0.3186)	0.1637 (0.3189)	0.2096 (0.2745)
# non-hydrogen atoms	1929	2120	1941	2126	3933
macromolecules	1698	1838	1738	1756	3612
ligands	38	34	34	30	65
solvent	193	248	169	340	256
Protein residues	219	222	218	221	463
RMS (bonds) ¶	0.012	0.019	0.013	0.019	0.003
RMS (angles) ¶	1.38	1.81	1.53	1.55	0.55
Ramachandran favored (%)	97.17	97.24	97.20	97.21	96.94
Ramachandran allowed (%)	2.83	2.76	2.80	2.79	2.84
Ramachandran outliers (%)	0.00	0.00	0.00	0.00	0.22
Rotamer outliers (%)	1.08	0.99	1.06	1.05	1.57
Clashscore	1.72	2.65	3.11	2.25	1.80
Average B-factor (Å²)	29.13	24.24	29.36	19.72	26.79
Macromolecules (Å²)	27.32	22.46	27.78	17.31	26.31
Ligands (Å²)	41.30	33.32	47.33	27.27	33.08
Solvent (Å²)	42.65	36.22	41.99	31.50	31.95
Number of TLS groups	9	NA	NA	NA	17

§ Parentheses indicate high resolution shell

§§ $R_{merge} = \sum_j \sum_h | I_{hj} - \langle I_h \rangle | / \sum_j \sum_h \langle I_h \rangle \times 100$

††$R_{work} = \sum ||Fobs| - |Fcalc|| / |Fobs| \times 100$

† R_{free}, based on 2-5% of the total reflections

¶ RMS deviation from ideality.

Statistics for the highest-resolution shell are shown in parentheses.

Figure S9. View from a crystal structure of BcII (turquoise) in complex with **6c** (yellow) (PDB ID: 5JMX). Active site residues shown as ball-and-stick with atoms coloured C (white), O (red), N (blue), Zn (grey spheres), water (red spheres). Ligand interactions are indicated with black dashed lines. Ligand mFo-DFc OMIT maps contoured to 3.0 σ are shown as light grey mesh. Bottom figure wall eye stereo.

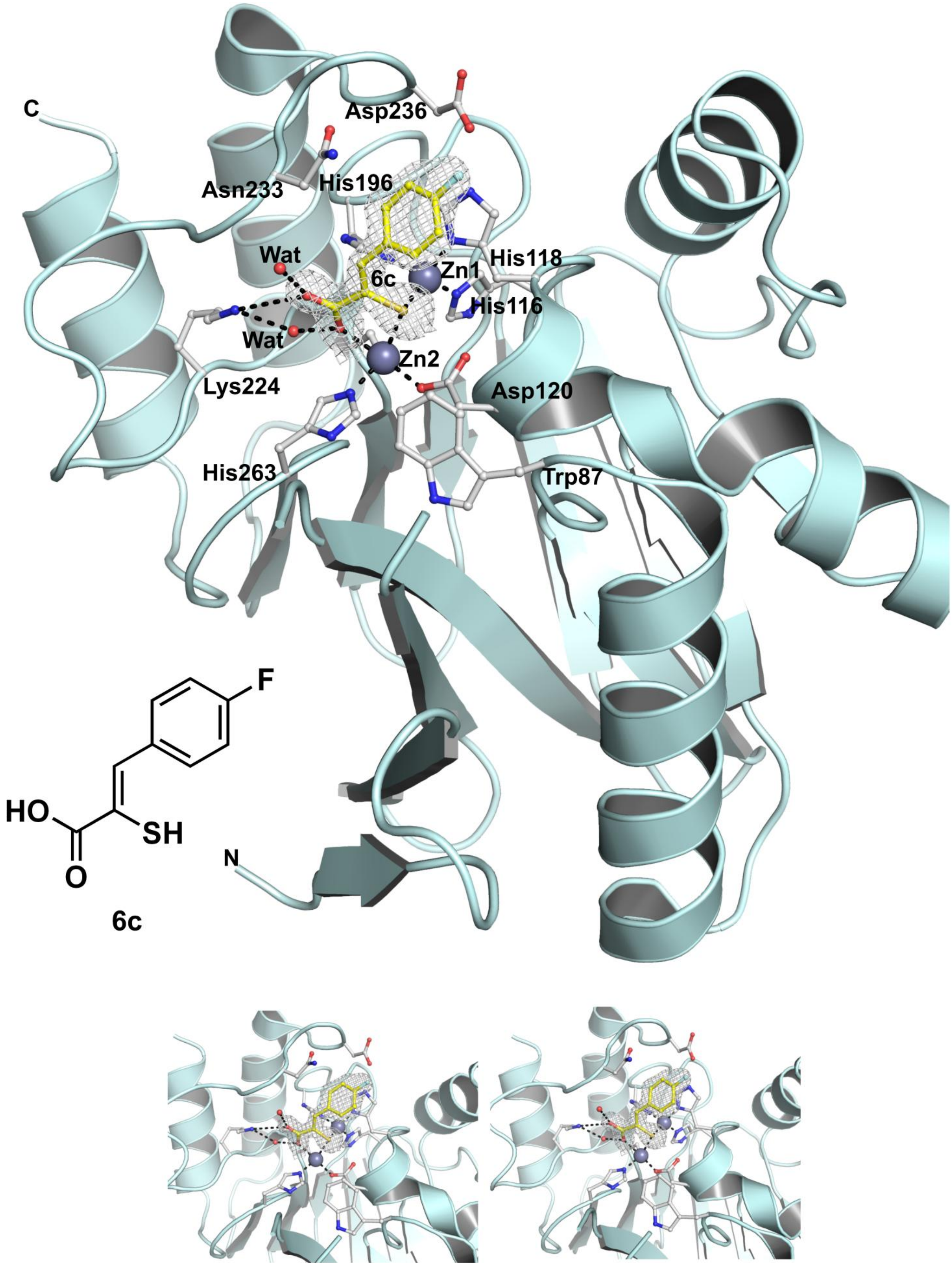

Figure S10. View from a crystal structure of BcII (turquoise) in complex with **6k** (salmon) (PDB ID: 6EUM). Active site residues shown as ball-and-stick with atoms coloured C (white), O (red), N (blue), Zn (grey spheres), water (red spheres). Ligand interactions are indicated with black dashed lines. Ligand mFo-DFc OMIT maps contoured to 3.0 σ are shown as light grey mesh. Bottom figure wall eye stereo.

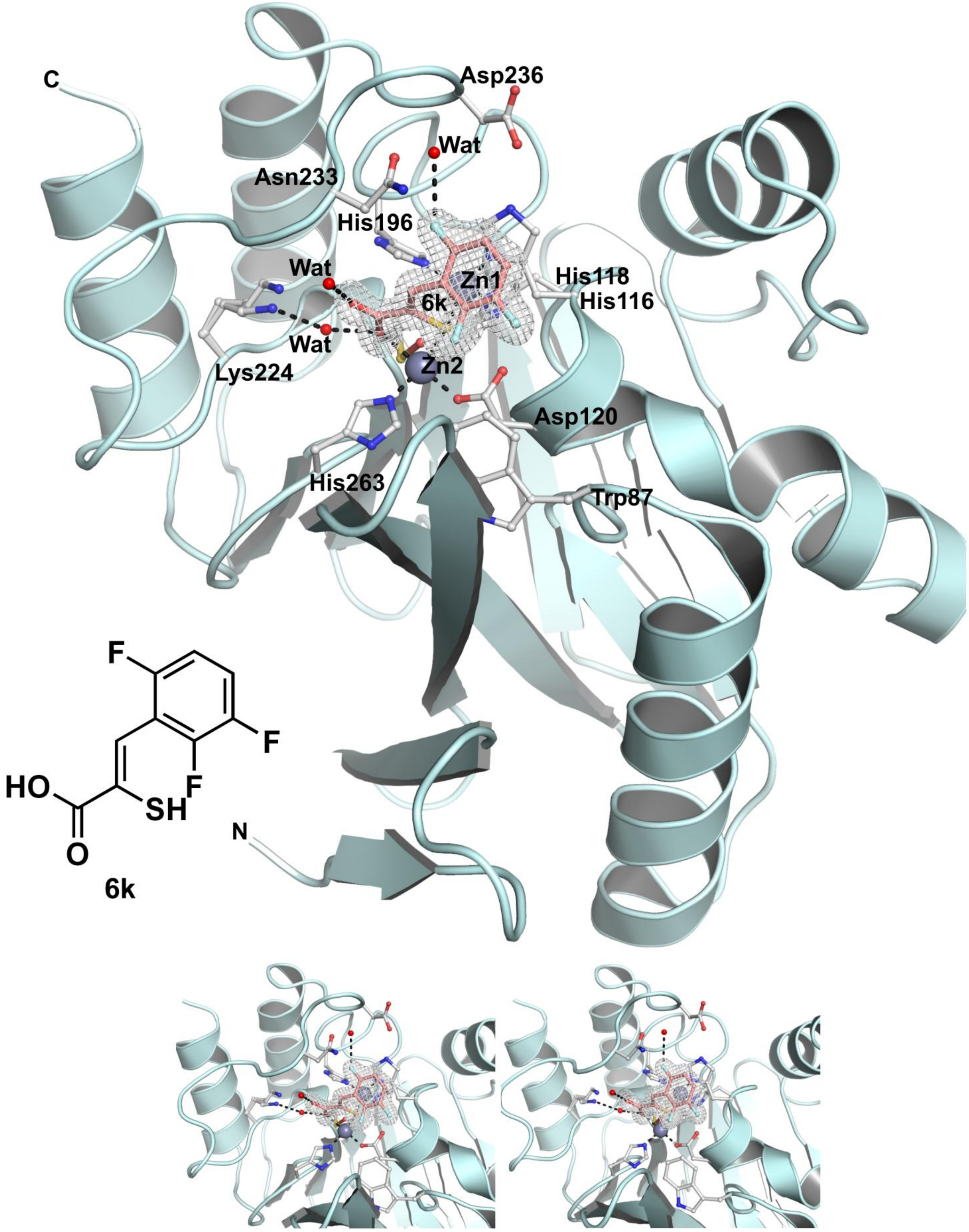

Figure S11. View from a crystal structure of BcII (turquoise) in complex with **6l** (purple) (PDB ID: 6EWE). Active site residues shown as ball-and-stick with atoms coloured C (white), O (red), N (blue), Zn (grey spheres), water (red spheres). Ligand interactions are indicated with black dashed lines. Ligand mFo-DFc OMIT maps contoured to 3.0 σ are shown as light grey mesh. Bottom figure wall eye stereo.

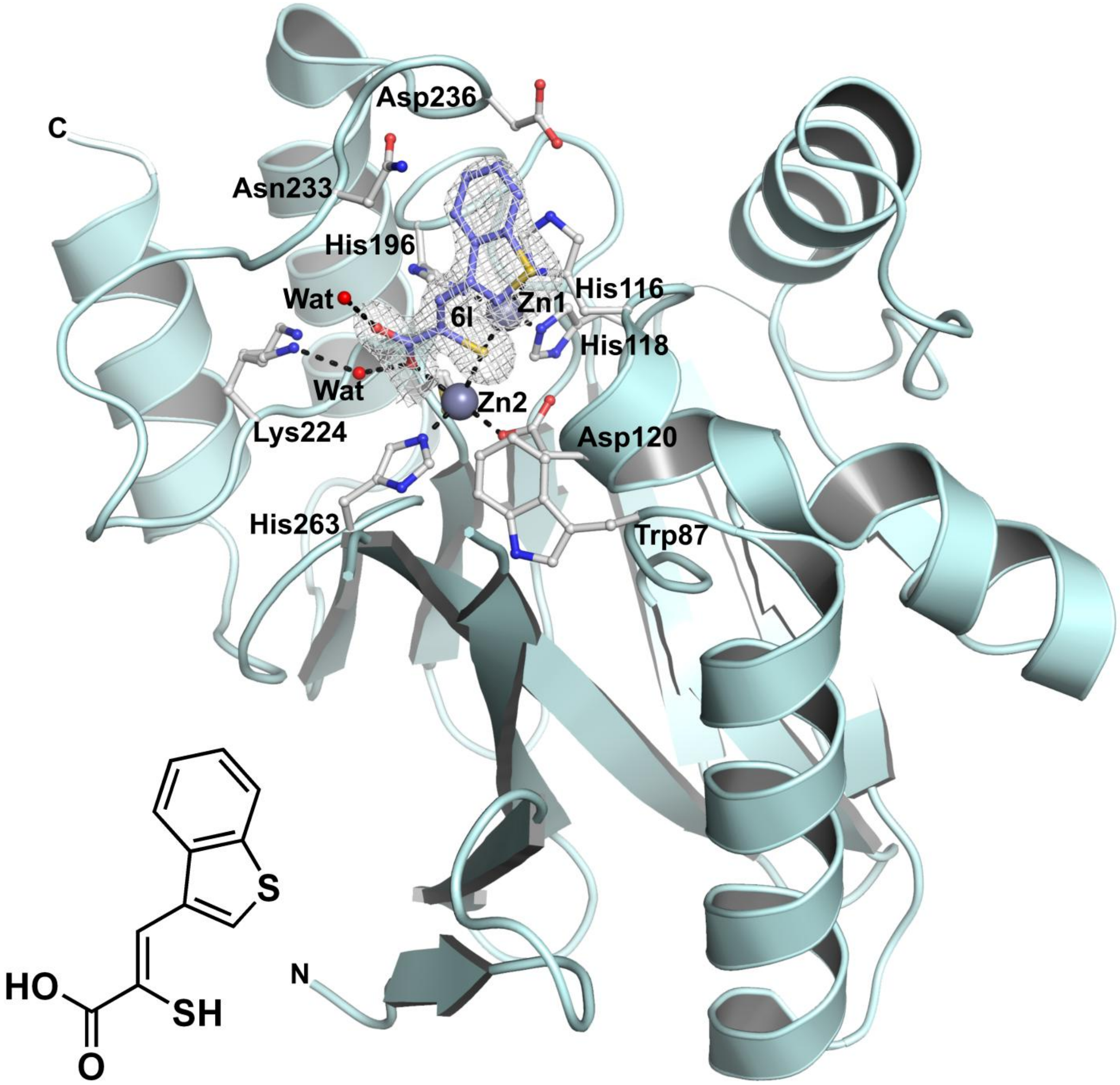

6l

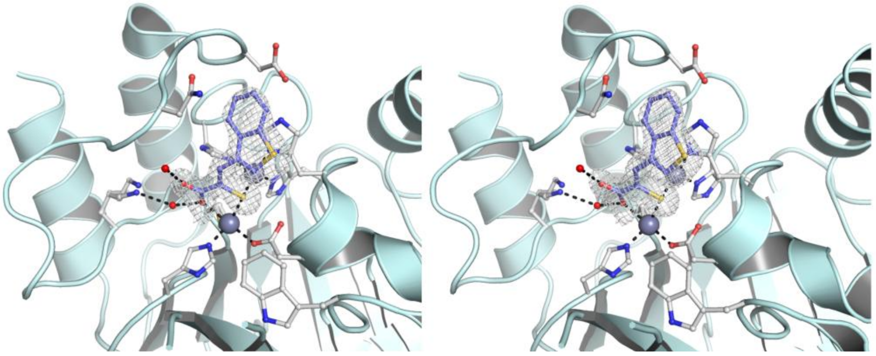

Figure S12. View from a crystal structure of VIM-2 (pink) in complex with **ML302F** (wheat) (PDB ID: 6EW3). Active site residues shown as ball-and-stick with atoms coloured C (white), O (red), N (blue), Zn (grey spheres), water (red spheres). Ligand interactions are indicated with black dashed lines. Ligand mFo-DFc OMIT maps contoured to 3.0 σ are shown as light grey mesh. Bottom figure wall eye stereo.

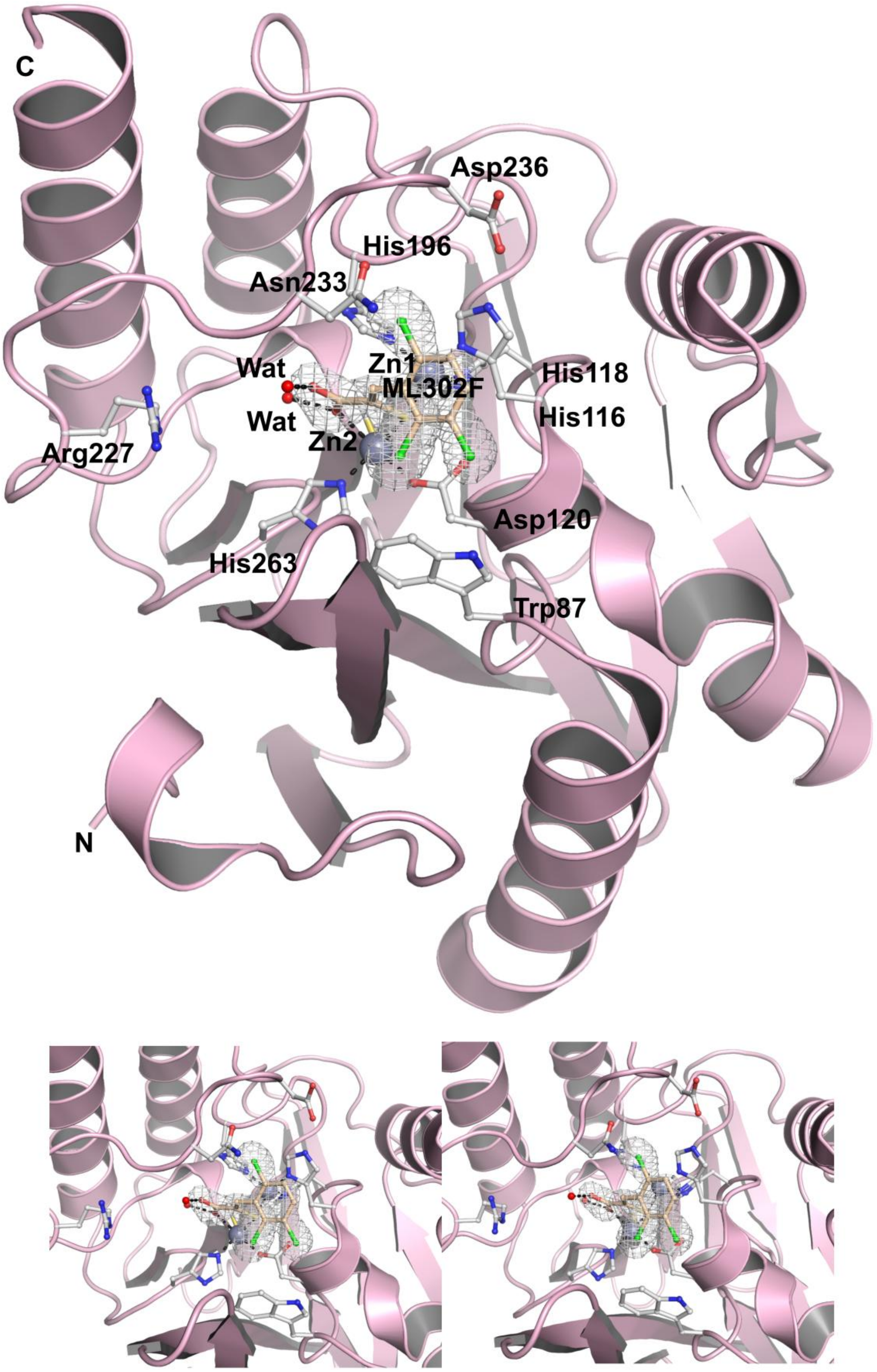

Figure S13. Crystallographic analysis reveals that the binding modes of enethiol carboxylate are similar to those of the MBL β-lactam antibiotic hydrolysis products. **a.** Binding mode of hydrolysed benzylpenicillin (penicillin G, cyan) with NDM-1 (PDB code: 4EYF)[1], **b.** Binding mode of **6s** (green) obtained by co-crystallization of **6s** with BcII (PDB ID:6F2N).

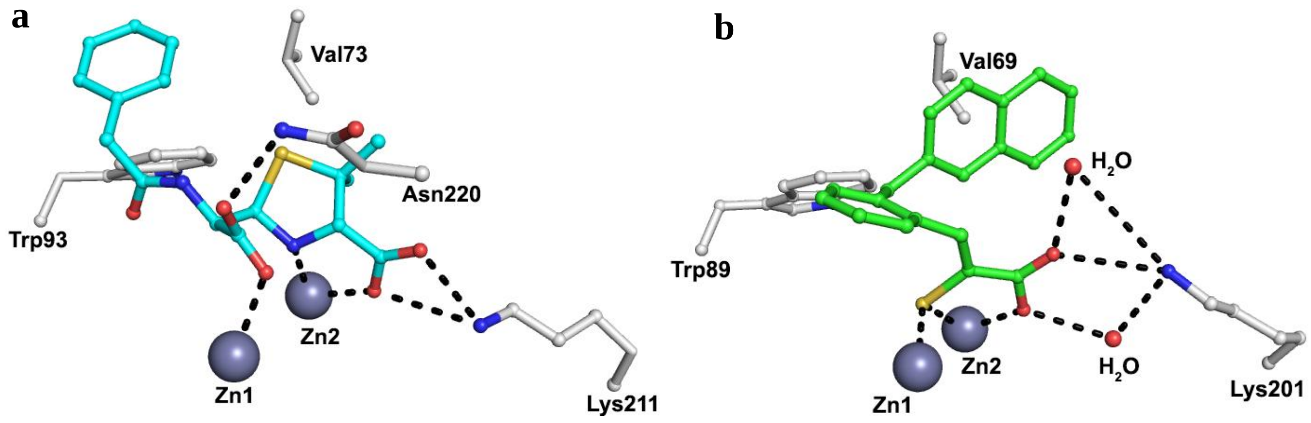

6. Author contributions

Dong Zhang, Marios S. Markoulides, Anna M. Rydzik, Klaus-Daniel Umland and Corentin Bon carried out synthesis. Marios S. Markoulides assisted with supervision.

Jürgen Brem carried out protein purifications.

Jürgen Brem, Michael A. McDonough, Patrick Collins, Samuel T. Cahill, Dmitrijs Stepanovs, and Ahmed El-Hussein carried out crystallography, with supervision by Frank von Delft, Jürgen Brem and Michael A. McDonough.

Assays were carried out by Dong Zhang, David Wang, and Samuel T. Cahill.

NMR experiments were carried out by Jos J. A. G. Kamps, with supervision by Timothy D. W. Claridge.

Christopher J. Schofield conceived the study and supervised the work.

The manuscript was written by Christopher J. Schofield, Michael A. McDonough and Dong Zhang with help from all the authors.

7. References:

(1) King DT, Worrall LJ, Gruninger R, Strynadka NC. *J.Am.Chem.Soc.* 2012, *134*, 11362-11365
